# Azetidines Kill Multidrug-Resistant *Mycobacterium tuberculosis* without Detectable Resistance by Blocking Mycolate Assembly

**DOI:** 10.1021/acs.jmedchem.3c01643

**Published:** 2024-02-08

**Authors:** Yixin Cui, Alice Lanne, Xudan Peng, Edward Browne, Apoorva Bhatt, Nicholas J. Coltman, Philip Craven, Liam R. Cox, Nicholas J. Cundy, Katie Dale, Antonio Feula, Jon Frampton, Martin Fung, Michael Morton, Aaron Goff, Mariwan Salih, Xingfen Lang, Xingjian Li, Chris Moon, Jordan Pascoe, Vanessa Portman, Cara Press, Timothy Schulz-Utermoehl, Suki Lee, Micky D. Tortorella, Zhengchao Tu, Zoe E. Underwood, Changwei Wang, Akina Yoshizawa, Tianyu Zhang, Simon J. Waddell, Joanna Bacon, Luke Alderwick, John S. Fossey, Cleopatra Neagoie

**Affiliations:** † School of Chemistry, University of Birmingham, Edgbaston, Birmingham, West Midlands B15 2TT, U.K.; ‡ Institute of Microbiology and Infection, School of Biosciences, University of Birmingham, Edgbaston, Birmingham, West Midlands B15 2TT, U.K.; § State Key Laboratory of Respiratory Disease, China-New Zealand Joint Laboratory on Biomedicine and Health, Guangzhou Institutes of Biomedicine and Health, Chinese Academy of Science, 190 Kai Yuan Avenue, Science Park, Guangzhou 510530, China; ∥ Sygnature Discovery, The Discovery Building, BioCity, Pennyfoot Street, Nottingham NG1 1GR, U.K.; ⊥ School of Biosciences, University of Birmingham, Edgbaston, Birmingham, West Midlands B15 2TT, U.K.; # College of Medical and Dental Sciences, University of Birmingham, Edgbaston, Birmingham, West Midlands B15 2TT, U.K.; ∇ Centre for Regenerative Medicine and Health, Hong Kong Institute of Science & Innovation, Chinese Academy of Sciences, 15 Science Park West Avenue NT, Hong Kong SAR; ○ ApconiX Ltd, BIOHUB at Alderly Park, Nether Alderly, Cheshire SK10 4TG, U.K.; ◆ Department of Global Health and Infection, Brighton and Sussex Medical School, University of Sussex, Falmer BN1 9PX, U.K.; ¶ TB Research Group, National Infection Service, Public Health England (UKHSA), Manor Farm Road, Porton, Salisbury SP4 0JG, U.K.; & Discovery Sciences, Charles River Laboratories, Chesterford Research Park, Saffron Walden CB10 1XL, U.K.; ● Visiting Scientist, School of Chemistry, University of Birmingham, Edgbaston, Birmingham, West Midlands B15 2TT, U.K.

## Abstract

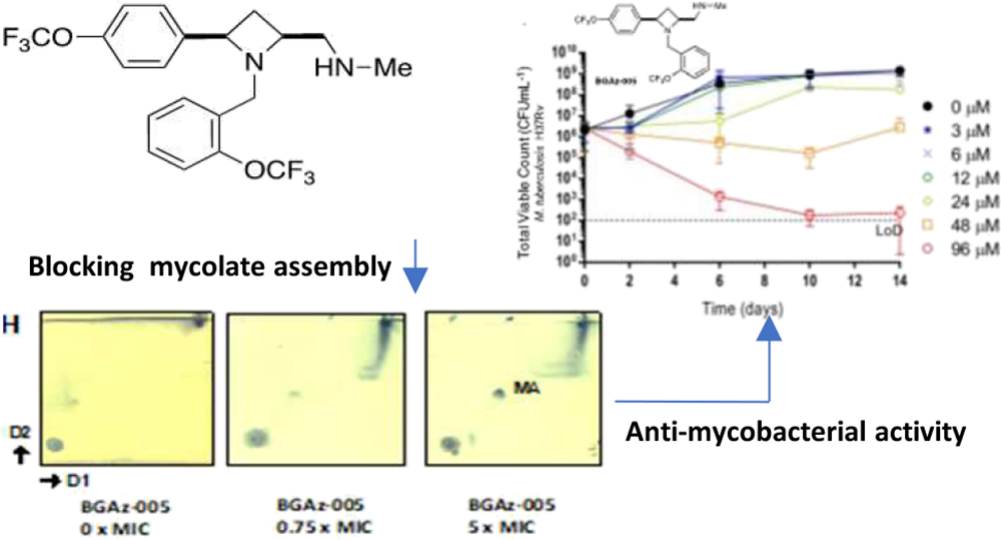

Tuberculosis (TB) is the leading cause of global morbidity and mortality resulting from infectious disease, with over 10.6 million new cases and 1.4 million deaths in 2021. This global emergency is exacerbated by the emergence of multidrug-resistant MDR-TB and extensively drug-resistant XDR-TB; therefore, new drugs and new drug targets are urgently required. From a whole cell phenotypic screen, a series of azetidines derivatives termed BGAz, which elicit potent bactericidal activity with MIC_99_ values <10 μM against drug-sensitive *Mycobacterium tuberculosis* and MDR-TB, were identified. These compounds demonstrate no detectable drug resistance. The mode of action and target deconvolution studies suggest that these compounds inhibit mycobacterial growth by interfering with cell envelope biogenesis, specifically late-stage mycolic acid biosynthesis. Transcriptomic analysis demonstrates that the BGAz compounds tested display a mode of action distinct from the existing mycobacterial cell wall inhibitors. In addition, the compounds tested exhibit toxicological and PK/PD profiles that pave the way for their development as antitubercular chemotherapies.

## Introduction

Tuberculosis (TB) is the principal infectious disease and cause of death worldwide, accounting for 1.4 million deaths in 2021. One-third of the world’s population is currently infected with latent TB, and over 10 million new cases of active TB are recognized per annum.^1,2^ Patients suffering from TB are treated with a cocktail of four drugs over a 6-month period. While cure rates can be as high as 90–95%,^3^ a combination of poor patient compliance and pharmacokinetic variability has led to the emergence of multidrug-resistant (MDR) and extensively drug-resistant (XDR) TB.^4,5^ The alarming increase in MDR-TB (500,000 new cases in 2018),^1^ coupled with the fact that the last novel frontline anti-TB drug, rifampicin, was discovered over 40 years ago,^6^ suggests that development and implementation of new control measures are essential for the future abatement of TB.^7,8^ Herein, the identity and antimycobacterial activity of azetidine derivatives with MIC_99_ values <10 μM against *Mycobacterium tuberculosis* are disclosed. These compounds did not give rise to emerging specific resistance in mycobacterial model organism *Mycobacterium smegmatis* and *Mycobacterium bovis* BCG. The mode of action and target deconvolution studies suggest that mycobacterial growth inhibition is conferred by a hitherto uncharacterized mechanism that arrests late-stage mycolic acid biosynthesis. DMPK and toxicology profiles confirm that the azetidine derivatives identified display relevant and acceptable profiles for translation.

## Results

### Identification and Development of Azetidine Derivatives with Antimycobacterial Activity

A bespoke compound library of novel lead-like small molecules^9^ for activity screening, which displayed a high fraction of sp^3^ (Fsp^3^, an indication of complexity and *3D-character*) atoms,^10−12^ were free from pan-assay interference compounds (PAINS),^13,14^ and are synthetically tractable, allowing for hit-to-lead scaffold elaboration,^15^ were sought. Unrelated synthetic chemistry methodology studies^16^ proved to be an ideal untapped source of such compounds.^17−20^ Compounds were fed into an open-ended antimycobacterial compound screen at the *University of Birmingham Drug Discovery Facility*.^21^ From that, an azetidine derivative **BGAz-001** (Table 1, entry 1), which displayed promising antimycobacterial activity against both *Mycobacterium smegmatis* and *Mycobacterium bovis* BCG, with MICs of 30.5 and 64.5 μM, respectively, was identified, and further azetidine derivatives were synthesized at *Guangzhou Institutes of Biomedical Health* (GIBH).^22^ Preliminary compound screening at 2 and 20 μM in an end point REMA assay to assess antimycobacterial activity, with subsequent secondary MIC determination against BCG and *M. smegmatis* (MIC refers to MIC_99_ unless otherwise stated), was sufficient to rule out the majority of ancillary compounds for further study.^23^ Analogues of the best performing compounds were synthesized, and structure–activity relationship (SAR) investigation was undertaken. Based on the screening results, 16 azetidine derivatives with MIC values against *M. smeg.* strains or *M. bovis* BCG strains lower than 100 μM are listed in Table1. Ten of these molecules had a molecular weight less than 500 Da, and 14 had *c*Log*P* values lower than 7, indicating an overall good potential for further anti-TB drug discovery based on their physicochemical property aspects.

**Table 1:**
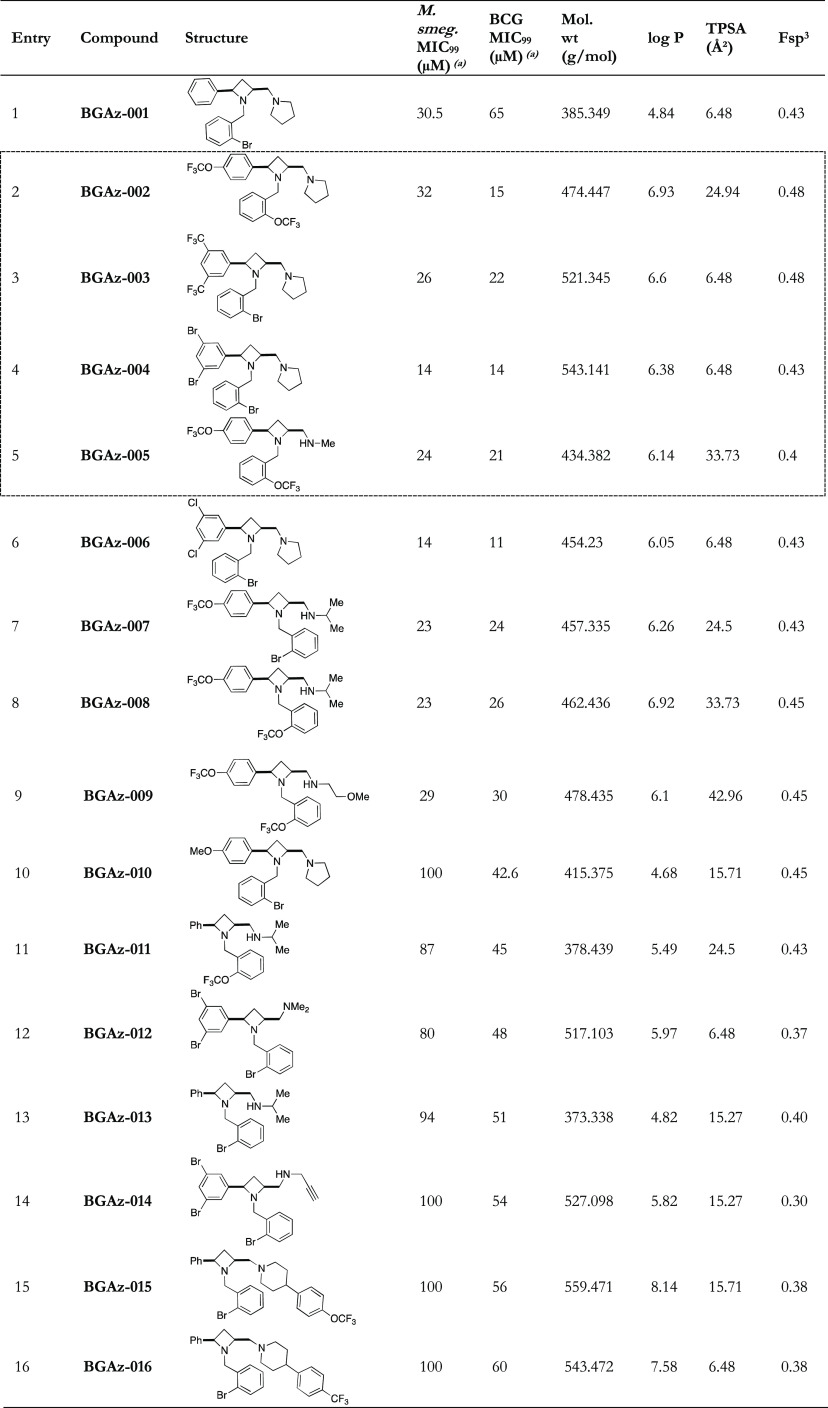
Activity-guided SAR Expansion of Hit Azetidine **BGAz-001-016**^a^^,^^b^

a MIC_99_ as determined by a modified Gompertz function.

b MIC values were determined from three biological replicates using a resazurin end point assay.

**Figure 1:**
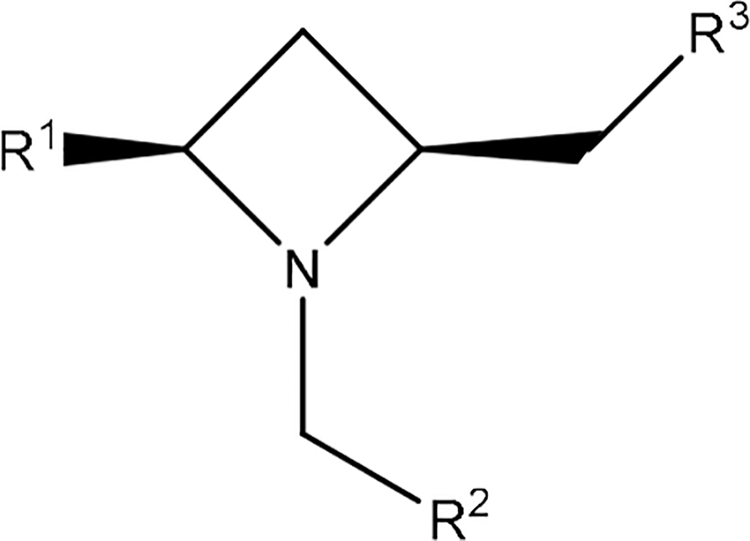
BGAz derivatives synthesized.

Compared with **BGAz-001, R**^**1**^ = phenyl (Figure 1), the compounds containing the electron-withdrawing group-functionalized phenyl ring of R^1^ (**BGAz-003**, **BGAz-004**, and **BGAz-006**) showed greatly increased activities against both *M. bovis* BCG and *M. smeg.* organisms, while **BGAz-002** showed similar activity against *M. smeg.* and better activity against *M. bovis* BCG (Table 1). It clearly appears that the electron-withdrawing group-functionalized phenyl ring of **R**^**1**^ favored inhibition against *M. smeg.* and *M. bovis* BCG compared with analogues decorated with an electron-donating group-functionalized phenyl ring of **R**^**1**^, (**BGAz-010**, Table 1). Introduction of electron-withdrawing groups (−Br, −Cl, −CF_3,_ and -OCF_3_) at the *para* and *meta* positions of the pendant aryl rings **R**^**1**^ resulted in an approximately three- to four-fold increase in activity against *M. bovis* BCG (**BGAz-002, BGAz-003, and BGAz-004**) or six-fold increase (**BGAz-006**) when compared to **BGAz-001.** Among the BGAz derivatives synthesized, active compounds often contained either a bromo substituent or trifluoromethyl ether at the *ortho* position of the azetidine *N*-benzyl group (**R**^**2**^, Figure 1) provided by this methodology. Regarding the **R**^**3**^, the amine pendant group (Figure 1), replacing the pyrrolidine with a methyl-, isopropyl-, or methoxypropyl-amine, while phenyl ring **R**^**1**^ bears an electron-withdrawing group, results in compounds (**BGAz-005**, **BGAz-007**, **BGAz-008**, and **BGAz-009** respectively), which retain antimycobacterial activity, with an approximately three-fold increase in activity against *M. bovis* BCG (**BGAz-005**) or with an approximately two-fold increase against *M. bovis* BCG **(BGAz-007**, **BGAz-008**, and **BGAz-009**). The inhibition against both *M. smeg.* and *M. bovis* BCG was considerably reduced when the pendant amine was dimethylamine and propargylamine, **BGAz-012** and **BGAz-014** (Table 1). Further iterations of BGAz modification included permutations of the pendant amines (isopropylamine), and aryl ring combinations (**R**^**1**^ = phenyl and **R**^**2**^ = trifluoromethoxyphenyl, bromomethyl) resulted in compounds with marginally reduced antimycobacterial activity (**BGAz-011 and BGAz-013**), showing the importance of the electron-withdrawing group-functionalized phenyl ring **R**^**1**^. In addition, the inclusion of piperidine derivatives as pendant amines provided no further enhancement in antimycobacterial activity (**BGAz-015** – **BGAz-016**).

Therefore, four azetidine-analogues (**BGAz-002 – BGAz-005**) with satisfactory activity against model organisms, which were representative of the subclass of chemistry identified, were selected for further evaluation. Compared with **BGAz-007, BGAz-008,** and **BGAz-009**, **BGAz-005** showed similar MIC values against *M. smeg.* but lower MIC values against *M. bovis* BCG. **BGAz-006** with the lowest MIC values was also encouraging for further development; however, due to time limitations, this was not included in this project. Antitubercular activity of azetidine derivatives (BGAz-002–BGAz-005) was observed.

Compounds **BGAz002**–**BGAz005** displayed antitubercular activity against *M. tuberculosis* strains that include reference strains H37Ra::pTYOK and H37Rv, and two clinical isolates *M. tuberculosis* (Beijing/W lineage 1192/015) and *M. tuberculosis* (Beijing 08/00483E) that are drug-sensitive or multidrug-resistant to isoniazid, rifampicin, pyrazinamide, and ethambutol (Table 2).

**Table 2:**
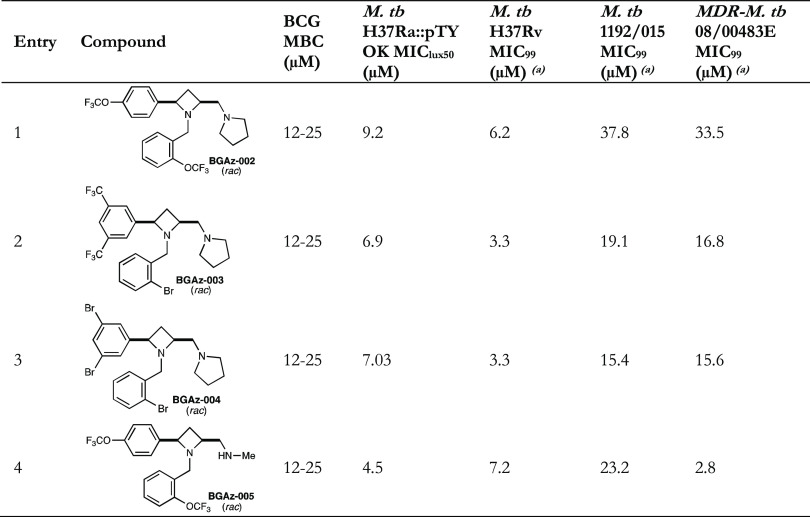
MIC and MBC Values of the **BGAz-002**–**BGAz-005** against Mycobacterial Strains With Different Drug-susceptibility Profiles^a^

a The *M. tb* H37Ra::pTYOK is an auto-luminescent strain of mycobacteria.^24^ The MIC_99_ values of **BGAz-002**–**BGAz-005** against *M. smegmatis* and *M. bovis* BCG, drug-sensitive *M. tuberculosis* H37Rv (reference strain) and *M. tuberculosis* 1192/015 (clinical isolate), and multi-drug-resistant *M. tuberculosis* 08/00483E (clinical isolate resistant to INH, RIF, PZA and EMB). MIC values were determined from three biological replicates using a resazurin end point assay. The minimum bactericidal concentration (MBC) was determined for **BGAz-002**–**BGAz-005** against *M. bovis* BCG.

Compounds **BGAz-002**–**BGAz-005** elicited antitubercular activities ranging from 4.5–9.2 μM MIC_lux50_ using a recently reported autoluminescent avirulent strain of *M. tuberculosis* H37Ra (Table 2, entries 1–4). Both **BGAz-003** and **BGAz-004** inhibit *M. tuberculosis* (H37Rv) at an MIC of 3.3 μM (Table 2, entries 2 and 3), with **BGAz-002** and **BGAz-005** inhibiting growth at MICs of 6.2 and 7.2 μM, respectively (Table 2, entries 1 and 4). The MICs determined for **BGAz-002**–**BGAz-005** are higher in the drug-sensitive *M. tuberculosis* Beijing/W (1192/015) clinical isolate compared to H37Rv. When comparing MICs between drug-sensitive and drug MDR clinical strains, no significant differences were observed for **BGAz-002**–**BGAz-004**, suggesting that the acquisition of mutations conferring front-line drug resistance does not impact the antitubercular activity of these compounds. However, it is noteworthy that **BGAz-005** was tested against *M. tuberculosis* 08/00483E, which is a clinical isolate that was sequenced at PHE (UKHSA) Porton using whole genome sequencing. The strain was confirmed to be resistant to all four frontline drugs (isoniazid, rifampicin, pyrazinamide, and ethambutol), as it has the following mutations, *katG S315, rpoB,* S450, *pncA+*T186, *and embB* M306 V.^25^ BGAz-005 displayed an 8-fold lower MIC (2.8 μM, Table 2, entry 4) in comparison to the drug-sensitive isolate. The absence of cross-resistance of these new anti-TB agents with the current frontline TB drugs is an important consideration in the development of new TB therapies with distinct modes of action.^26^ Evaluation of the minimal bactericidal concentrations (MBCs) for **BGAz-002**–**BGAz-005** against BCG demonstrates that these compounds exhibit bactericidal activity, since both MIC and MBC values overlap (Table 2).

### Physiochemical and Toxicological Properties of **BGAz-002–BGAz-005**

**BGAz-002**–**BGAz-005** were subjected to in vitro DMPK testing; **BGAz-001** was excluded from further study due to its comparatively poor antimycobacterial activity. The poorer kinetic solubility **BGAz-002**–**BGAz-004** (9 to 57 μM) in aqueous buffered solution in comparison to the higher solubility of **BGAz-005** (117 μM) can be attributed to the presence of a secondary versus tertiary amine functionality (Table S3). Metabolic stability of the compounds was evaluated through measuring the intrinsic clearance (CL_int_) by mouse liver microsomes and by liver hepatocytes. Compounds **BGAz-002**–**BGAz004** all exhibited a CL_int._ of >150 μL/min/mg in the microsomal stability assay, indicating a rapid clearance (Table S3, entries 1 to 3), with **BGAz-005** giving the lowest rate of microsomal clearance (36 μL/min/mg, Table S3, entry 4). Experiments were repeated using mouse liver hepatocytes, and all four compounds afforded CL_int_ values of <60 μL/min/mg, indicating good overall metabolic stability (Table S3). Caco-2 permeability assays were conducted to predict both intestinal permeability and drug efflux. Compounds **BGA-002**–**BGAz-004** exhibited poor efflux ratios while **BGAz-005** continued to perform well with an efflux ratio of less than 1.0.

The pharmacokinetic (PK) parameters of **BGAz-001**–**BGAz-005** in a mouse model were investigated by cassette (combined) dosing at 5 mg/kg PO, 1 mg/kg IV, and IP (PK data file: Tables S1–S5). For the oral dosing, at 5 mg/kg, **BGAz-001**, **BGAz-002,** and **BGAz-003** gave peak serum concentrations (*C*_max_) of 54.7, 53.9, and 56.0 μg/L, respectively. **BGAz-004** gave the highest *C*_max_, whereas **BGAz-005** gave the lowest, 87.6 and 43.1 μg/L, respectively (Table 3, entries 2 and 3). The plasma half-life (*T*_1/2_) of **BGAz001** was revealed to be 1.5 h, and those of **BGAz002** and **BGAz003** were 24.9 and 28.0 h, respectively. **BGAz004** has a half-life of 11.4 h, with **BGAz005** having the longest half-life of 35.7 h. **BGAz001–003** was excluded from further study due to poor PK/PD parameters. Mice were dosed multiple times with **BGAz-004** and **BGAz-005** (four and three times, respectively) at 30 mg/kg (PO) in order to provide evidence of compound tolerability and refinement of measured parameters. **BGAz-004** and **BGAz-005** gave *C*_max_ values of 363.0 and 1712.5 μg/L, respectively; *T*_1/2_ values of 8.1 and 80.4 h were calculated, respectively (Table 3, entries 2 and 3). The total body exposure from multiple dosing at 30 mg/kg (PO) of **BGAz-005** (82247.7 ng/mL*h) was significantly greater than **BGAz-004** (5420.99 ng/mL*h), indicating a superior overall pharmacokinetic profile for **BGAz-005** (Table 3, entries 2 and 3). **BGAz-002**, **BGAz-004,** and **BGAz-005** were tested for cytochrome P450 (CYP450) metabolic activity by measuring the inhibition of each of specific enzymes in human liver microsomes. All three compounds exhibited no discernible inhibition of CYP1A2, an enzyme known to metabolize aromatic/heterocyclic amine-containing drugs (Table 3). The CYP2C9 enzyme is a relatively abundant CYP450 in the liver that dominates CYP450-mediated drug oxidation. In this regard, only minimal CYP2C9 inhibition when **BGAz-004** was preincubated for 30 min prior to the addition of NADPH to initiate catalysis was observed. Known to metabolize a wide range of drug molecules, CYP2C19 is an essential member of the CYP450 superfamily as it contributes ∼16% of total hepatic content in humans. **BGAz-002** and **BGAz-005** displayed only negligible inhibition of this enzyme, **BGAz-004** displayed strong inhibitory activity when preincubated for 30 min prior to initiation of catalysis (Table 3). CYP2D6 is widely implicated in the metabolism of drugs that contain amine functional groups, such as monoamine oxidase inhibitors and serotonin reuptake inhibitors. CYP2D6 is responsible for the second highest number of drugs metabolized by the CYP450s, as demonstrated by the significant inhibition of this enzyme by **BGAz-002**, **BGAz-004,** and **BGAz-005**. All three compounds were evaluated for mitochondrial dysfunction by measuring IC_50_ values against HepG2 cells cultured in media containing either glucose or galactose, which serves to direct cellular metabolic activity toward glycolysis or oxidative phosphorylation, respectively. While **BGAz-004** exhibited a negligible effect, both **BGAz-002** and **BGAz-005** demonstrated cytotoxicity with IC_50_ values of 38 and 21 μM, respectively (Table 3). To enhance cellular susceptibility to mitochondrial toxicants, assays were repeated in the presence of galactose, which resulted in Glu/Gal ratios of <1, confirming no mitochondrial toxicity. Compounds **BGAz-002**, **BGAz-004,** and **BGAz-005** were assayed for hERG inhibition using *IonWorks* patch clamp electrophysiology. An eight-point concentration–response curve was generated from a three-fold serial dilution of a top compound concentration of 167 μM. Compound **BGAz-002** was the best performing compound, displaying a hERG liability IC_50_ of 173 μM, (i.e., inhibition of less than 50% at the top 167 μM test concentration). In comparison, compounds **BGAz-004** and **BGAz-005** performed significantly worse with hERG liability IC_50_ of 25 and 12.7 μM, respectively. Overall, the BGAz compounds investigated in this preliminary study display an encouraging toxicological and PK/PD profile to enable further exploration and development toward clinics.

**Table 3:**
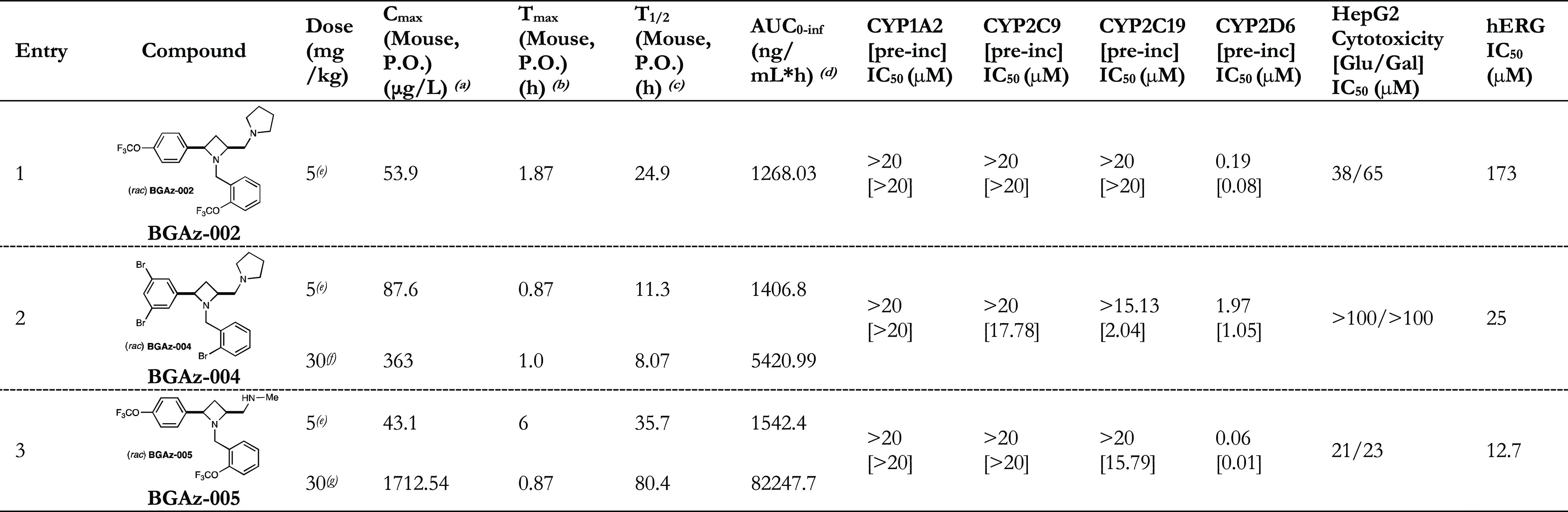
Pharmacokinetic Profiles, CYP450 Activities, Mitochondrial Dysfunction, and hERG Liabilities of **BGAz-002**, **BGAz-004,** and **BGAz-005**

a Measures maximum peak serum concentrations of the drug.

b The time of first occurrence of *C*_max_.

c The terminal half-life of the drug.

d The area under the plasma drug concentration–time curve to infinite time.

e As a result of cassette dosing of **BGAz-001–005;** see the *PK Data* file in the Supporting Information.

f Multiple daily dosing 4 × 30 mg/kg; see *the PK Data* file in the Supporting Information.

g Multiple daily dosing 3 × 30 mg/kg; see *the PK Data* file in the Supporting Information.

### BGAz Compounds Kill *M. tuberculosis* with Bactericidal Activity

**Figure 2:**
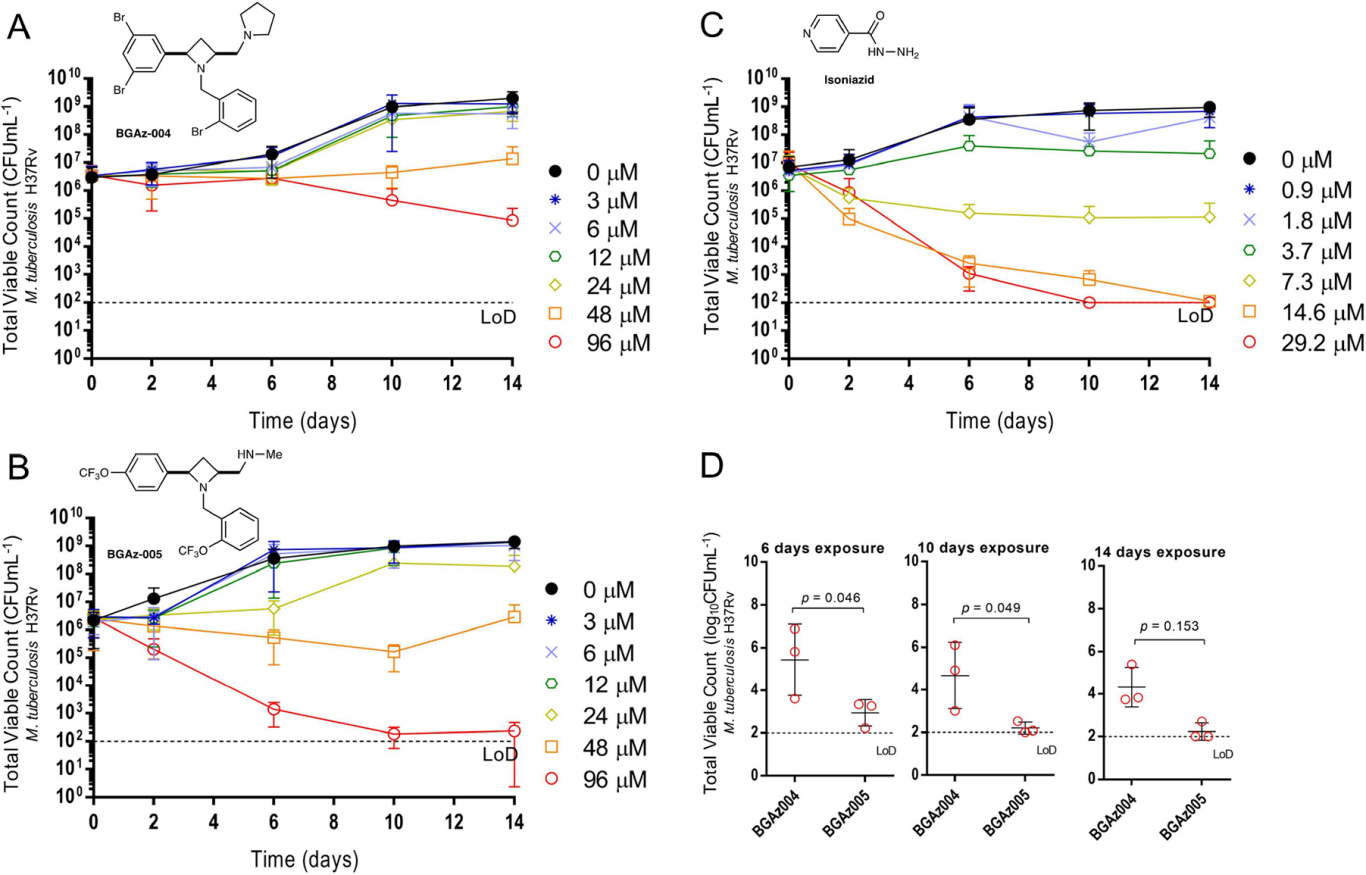
Assessment of bactericidal activity of **BGAz-004** and **BGAz-005** against *M. tuberculosis* H37Rv. Average total viable counts (CFU mL^–1^) of *M. tuberculosis* cultures exposed to either **BGAz-004** (Panel A) or **BGAz-005** (Panel B) at concentrations: 0 μM (0.1% DMSO) (circle, closed), 3, 6, 12, 24, 48, and 96 μM or isoniazid (Panel C) at concentrations 0 μM (0.1% DMSO), 0.9, 1.8, 3.7, 7.3, 14.6, and 29.2 μM over a 14-day time-course. Samples were taken after 0, 2, 6, 10, and 14 days of antibiotic exposure, serially diluted, and plated by the method of Miles et al.^27^ Statistical comparisons were performed at 6, 10, and 14 days of antibiotic exposure at 96 μM **BGAz-004** and **BGAz-005** using factorial ANOVA and posthoc Tukey’s honestly significant difference test (Panel D). Data represent three biological repeats ± standard deviation.

The bactericidal activity of **BGAz-004** and **BGAz-005** against *M. tuberculosis* H37Rv was further assessed by exposing the bacilli to a range of concentrations of **BGAz004** and **BGAz-005** over a time-course of 14 days and total viable counts (CFU mL^–1^) enumerated on solid medium. Both **BGAz-004** and **BGAz-005** were active against *M. tuberculosis* H37Rv (Figure 2, panels A and B), with **BGAz-005** demonstrating statistically significantly greater early bactericidal and concentration-dependent activity than **BGAz-004** at day 6 (*P* = 0.046) and day 10 (*P* = 0.049) (Figure 2D). Exposure of *M. tuberculosis* H37Rv to **BGAz-005** resulted in a greater bactericidal effect with more pronounced activity earlier in the time-course, with a reduction of 3.28 ± 1.00 log_10_ CFU mL^–1^ after 6 days of exposure and reduction of 3.99 + 0.60 log_10_ CFU mL^–1^ after 14 days’ exposure, at a concentration of 96 μM (Figure 2B). Equivalent activity was not observed by **BGAz-004** early in the time-course and showed delayed activity at all concentrations, only achieving a decrease in 0.79 ± 2.20 log_10_ CFU mL^–1^ by day six and 1.90 ± 1.28 log_10_ CFU mL^–1^ reduction after 14 days at 96 μM (Figure 2A). The profile for **BGA**_**Z**_**-004** is commensurate with antibiotics that exhibit bacteriostatic activity at lower concentrations. Isoniazid demonstrated a higher rate of bactericidal activity compared to both **BGAz-004** and **BGAz-005** by achieving a reduction of >4.52 ± 0.60 log_10_ CFU mL^–1^ to a limit of detection (100 CFU mL^–1^), by day 10, at a lower concentration of 29 μM (Figure 2C).

**Figure 3:**
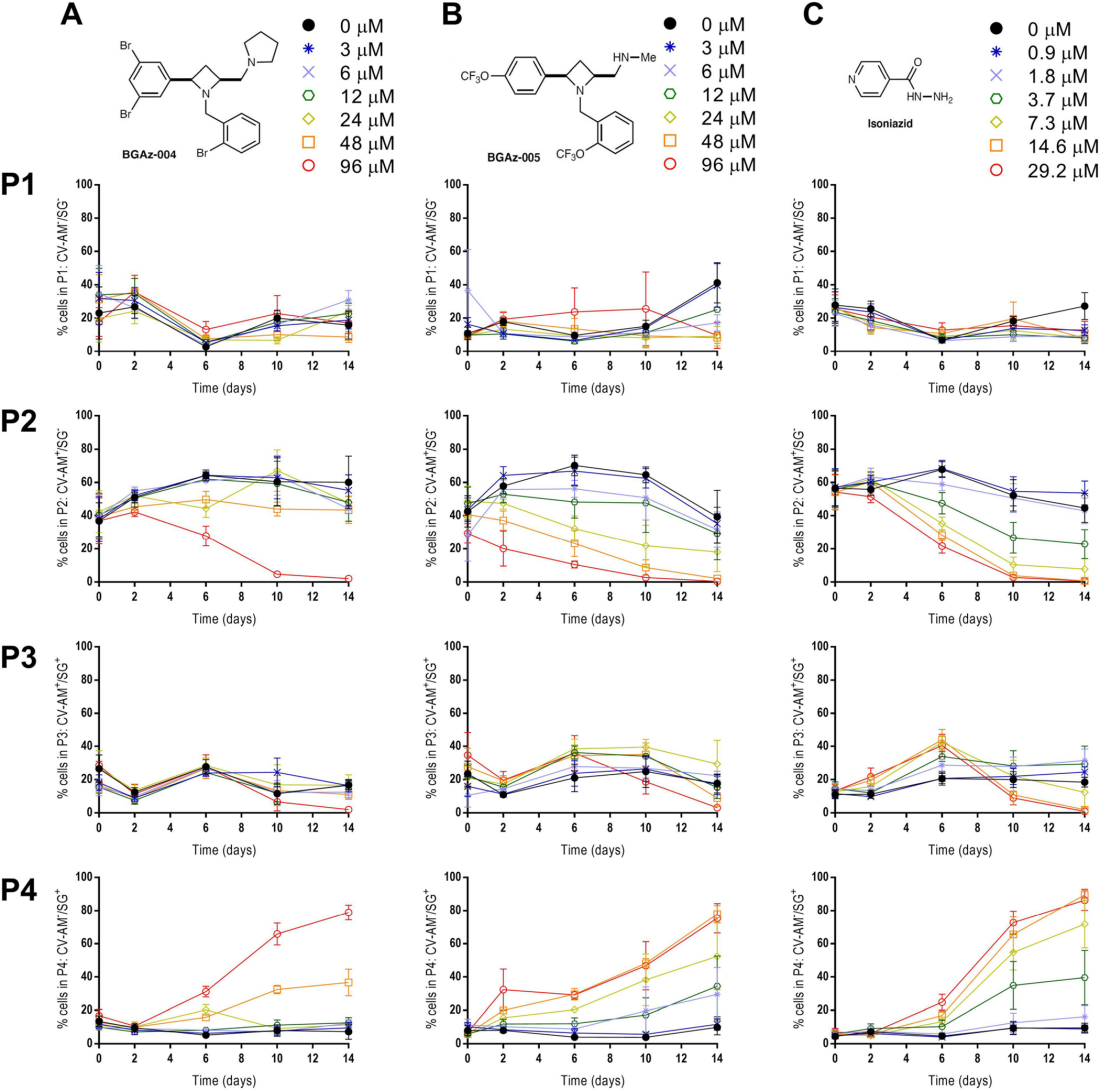
Assessment of bactericidal activity of **BGAz-004** and **BGAz-005** against *M. tuberculosis* H37Rv. Quantitation of Calcien-Violet-AM (CV-AM) and Sytox-green (SG) fluorescence of *M. tuberculosis* H37Rv, using flow cytometry, after exposure to **BGAz-004** (column A) and **BGAz-005** (column B) at concentrations: 0 μM (0.1% DMSO), 3, 6, 12, 24, 48, and 96 μM or (column C) isoniazid at concentrations 0 μM (0.1% DMSO), 0.9, 1.8, 3.7, 7.3, 14.6, and 29.2 μM over a 14-day time-course. The percentages of the population that are unstained or stained with each dye (or both dyes) are represented in four gates (rows P1–P4). Row P1: unstained population (CV-AM^–^/SG^–^); row P2: CV-stained population (CV-AM^+^/SG^–^); row P3: dual-stained population (CV-AM^+^/SG^+^); and row P4: SG-stained population (CV-AM^–^/SG^+^). Data represent three biological repeats ± standard deviation. Statistical comparisons were made using factorial ANOVA and *posthoc* Tukey’s honestly significant difference test.

In addition to the enumeration of viable bacilli on agar, the activity of **BGAz-004** and **BGAz-005** was determined using flow cytometry. This approach allows for a direct assessment of whether BGAz compounds are able to kill *M. tuberculosis* in a dose-dependent manner and whether the killing profile was similar between these compounds and to that observed for isoniazid, which would provide insights about their mode of action.^28^ Culture samples were taken at each time-point and dual-stained using Calcein Violet with an acetoxy-methyl ester group (CV-AM) that is a correlate of metabolic activity, and Sytox Green (SG) that enables measurement of cell-wall permeability (a proxy for cell death). Single bacilli were identified by forward scattered light area and height using flow cytometry analyses. Gated single cells were further differentiated based on the presence and absence of CV-AM and SG staining using a quadrant gating approach. The percentages of the population that are unstained or stained with each dye (or both dyes) are represented in four gates P1–P4 (P1: CV-AM^–^/SG^–^, P2: CV-AM^+^/SG^–^, P3: CV-AM^+^/SG^+^, P4: CV-AM^–^/SG^+^) (Figure 3). The CV-AM staining profiles (metabolic activity) for these compounds were reflective of the total viable counts (Figure); the decrease in CV-AM staining over the time-course at 96 μM for **BGAz-005** was statistically significant after day 6 (*P* = 0.043), day 10 (*P* = 0.08), and day 14 (*P* = 0.011) compared to the decrease in the CV-AM staining for **BGAz-004**, at the same concentration (Figure 3A,B; P2). A similar difference in activity was observed at 48 μM, (*P* = 0.067, 0.065, 0.066 for days 6, 10, and 14, respectively). The SG-staining profiles showed that both compounds possessed equivalent killing activity at high concentrations of 96 μM (Figure 3A,B; P4); however, **BGAz-005** shows higher levels of kill at days 6 and 14 with a lower concentration of 48 μM (*P* = 0.027 and 0.068, respectively). Both **BGAz-004** and **BGAz-005** show similar staining profiles to isoniazid (Figure 3C), which targets the mycobacterial cell wall.^28^

### **BGAz-004** and **BGAz-005** Inhibit the Incorporation of Mycobacterial Cell Wall Precursors and Display No Detectable Resistance

**Figure 4:**
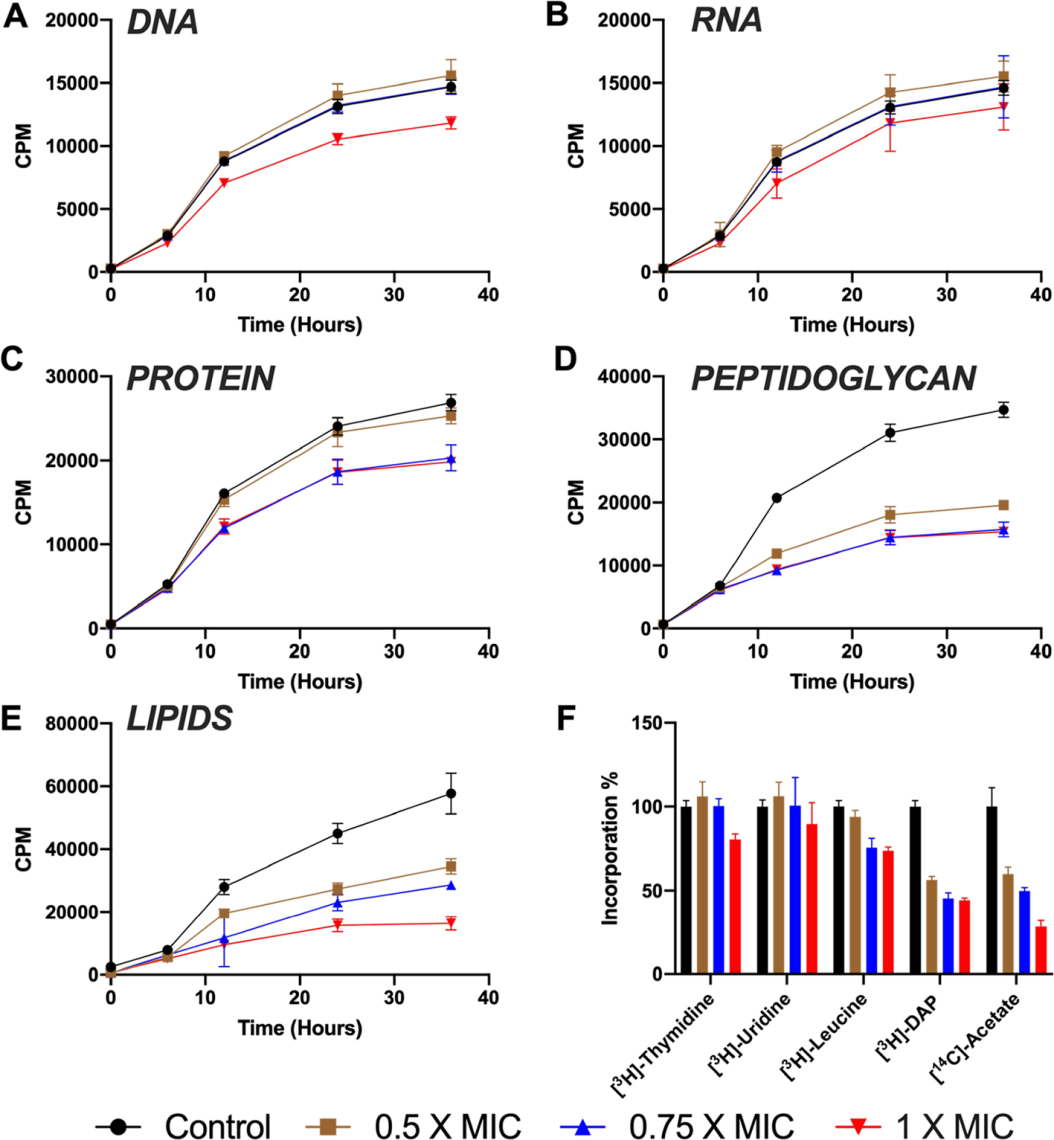
Effect of **BGAz-005** on the incorporation of radiolabeled precursors into the major cellular macromolecules of *M. smegmatis*. The incorporation of (A) [*methyl-*^3^H]thymidine (for DNA), (B) [5,6-^3^H]uridine (for RNA), (C) l-[4,5-^3^H]leucine (for protein), (D) [^3^H]*meso-*diaminopimelic acid (for peptidoglycan), and (E) [^14^C]acetic acid (for lipids) was measured over a period of 36 h. The percentage of incorporation measured at 36 h is represented in panel F. Each plot and error bars represent the average of three independent experiments.

Whole genome sequencing (WGS) of laboratory-generated mutants that are resistant to TB drugs is a widely used approach to determine the mode of action of novel antibacterial compounds.^29−31^ Multiple attempts (>5 biological repeats) to generate drug-resistant mutants of **BGAz-002–BGAz-005** in *M. smegmatis* and *M. bovis* BCG (including a strain of BCG devoid of *recG* which has a higher mutational frequency)^32^ were unsuccessful, implying an undetectably low frequency of resistance for these compounds (Supporting Information). This advantageous property is a double-edged sword. The discovery of the BGAz series as novel antitubercular compounds with low frequencies of resistance is attractive in terms of drug development, especially in the context of MDR-TB; however, the inability to generate resistant mutants against the most active compounds suggests that this series of compounds may elicit pleiotropic activity or have nonspecific modes of action, or nonprotein target(s). Therefore, in order to investigate the mode of action of **BGAz-005**, the most active of the compounds tested against mycobacteria, biosynthetic inhibition of five major macromolecular pathways was evaluated by measuring the incorporation of selected radiolabeled precursors during microbial cell culture. The addition of **BGAz-005** up to a concentration of 0.75 × MIC had almost no effect on the incorporation of [^3^H]-thymidine, [^3^H]-uridine, and [^3^H]-leucine with only a moderate 20% reduction of incorporation at 1 × MIC, suggesting that **BGAz-005** does not directly inhibit DNA, RNA, or protein biosynthesis (Figure 4). In contrast, **BGAz-005** decreased the incorporation of both [^3^H]-DAP and [^14^C]-acetic acid from 6 h postlabeling and, at 0.5× and 1 × MIC, caused a titratable decrease in [^14^C]-acetic acid incorporation, exerting a ∼50 and ∼75% loss of lipid biosynthesis, respectively (Figure 4). These data suggest that the **BGAz-005** acts by inhibiting aspects of mycobacterial cell envelope biosynthesis.

### **BGAz-005** Dysregulates the Expression of Cell Envelope Biosynthetic Genes

**Figure 5:**
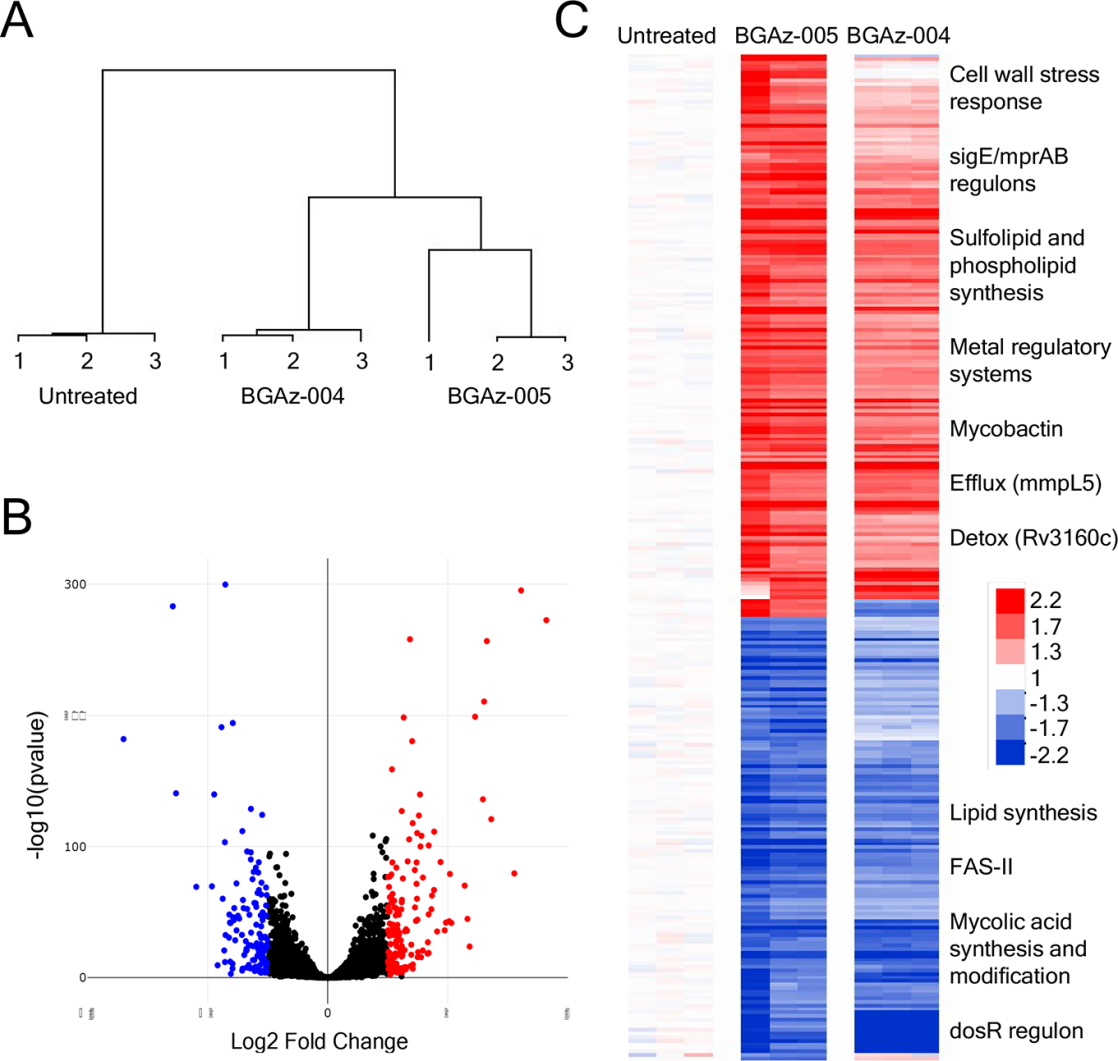
Transcriptional response to **BGAz-005** exposure demonstrating inhibition of mycobacterial cell envelope biosynthesis. (A) Cluster diagram of all genes showing similarity of biological replicates and separation of drug-treated from carrier control samples. (B) Volcano plot of *M. bovis* BCG response to **BGAz-005**, highlighting genes significantly differentially expressed. (C) Heatmap of 286 gene **BGAz-005** signature relative to carrier control. Conditions as columns, genes as rows; red coloring highlighting induced genes and blue representing repressed genes. The **BGAz-004** signature is clustered alongside, indicating a similar mode of drug action.

To explore the mode of action of **BGAz-005** using an unsupervised approach, the *M. bovis* BCG transcriptional response to drug exposure was profiled by RNaseq A signature consisting of 160 induced and 126 repressed genes was identified after 8 h’ exposure to 1 × MIC **BGAz-005**. The response was comprised of three principal features, namely, inhibition of cell wall biosynthesis, dysregulation of metal homeostasis, and disruption of the respiratory chain (Figure 5). The inhibition of cell wall synthesis was evidenced by induction of key regulators of cell wall stress *sigE* and *mprAB*, alongside significant upregulation of their regulons (hypergeometric p value of *sigE* regulon enrichment 2.47 × 10^–17^;^33^*mprAB* hgp 5.29 × 10^–11^).^34^ In contrast to isoniazid and ethambutol where FasII genes are induced by drug exposure, FasII genes (*hadA*, *fabG1*, *inhA*, *acpM*) alongside mycolic acid synthesis and modification genes (*mmaA2*, *mmaA3*, *fbpA*, *fbpB*, *fbpD*, and *desA2*) were repressed by **BGAz-005** treatment, indicating a different mechanism of **BGAz-005** drug action to cell wall inhibitors currently in use. The functional category (I.H) lipid biosynthesis was significantly repressed by **BGAz-005** (hgp 6.52 × 10^–5^), and mycolyl-arabinogalactan-peptidoglycan complex biosynthesis was the top pathway dysregulated by **BGAz-005** (pathway perturbation score of 3.4).^35,36^ Genes involved in the synthesis of alternative cell wall factors, sulfolipids (*mmpL8*, *papA1*, and *pks2*) and the oleic acid stearoyl-CoA desaturases that produce phospholipids (*desA3_1*, *desA3_2*, and *BCG_3260c/Rv3230c*), were induced.^37^ A series of metal-responsive regulatory systems were upregulated by **BGAz-005** (*cmtR*, *zur*, *ideR*, and *tcrYX*) as well as genes encoding the lipid-bound siderophore mycobactin (*mbtB*, *mbtC*, and *mbtD*), representing disruption of metal control systems, likely impacted by loss of cell-wall structure. Induction of redox-inducible *clgR* in combination with repression of the *dosR* regulon (hgp 6.40 × 10^–9^) reflected the impact of **BGAz-005** on the respiratory chain.^38^ However, unlike many drugs that affect respiration, no differential expression of energy metabolism systems (*nuoA-N*, *qcrA-C*, *ctaC-E*, *cydA-D*, and *narG-J*) was observed.^39,40^ Systems implicated in the efflux (*mmpL5*, *mmpS5, BCG_0727*/*Rv0678*, and *BCG_0728c*/*Rv0679c*) or detoxification (*BCG_3184c*/*Rv3160c*, *BCG_3185c*/*Rv3161c*, and *BCG_3186c*/*Rv3162c*) of antimicrobial drugs were also induced by **BGAz-005**.^41^ Significantly, the efflux pump *efpA*, highly induced by cell wall targeting drugs isoniazid, ethambutol, and benzothiazinone, was not induced by **BGAz-005** exposure.

Mapping the drug-responsive gene clusters identified by Boshoff and co-workers revealed significant enrichment of GC-27 and GC-82 representing cell wall inhibition,^42^ alongside GC-39 (*dosR* regulon) and GC-108 (iron scavenging). The most similar drug signatures were the phenothiazines, chlorpromazine, and thioridazine (hgp 2.83 × 10^–14^), disrupting the cell wall and electron transfer chain,^43^ alongside analogues of ethambutol (hgp 4.98 × 10^–13^)^44^ and benzothiazinone (hgp 8.00 × 10^–8^) targeting arabinose biosynthesis in the mycobacterial cell wall.^45^ Thus, **BGAz-004** and **BGAz-005** elicit a transcriptomic response representing major abrogation of normal cell envelope function.

### **BGAz-004** and **BGAz-005** Significantly Alter Mycobacterial Cell Envelope Composition

**Figure 6:**
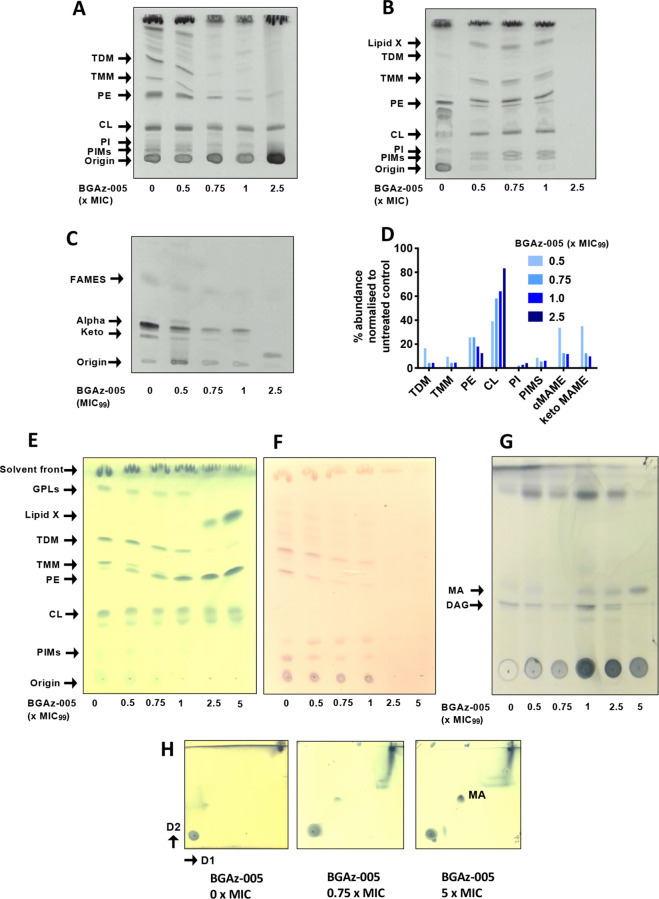
BCG cell envelope lipid analysis upon exposure to **BGAz-005**. BCG were cultured in 7H9 broth and exposed to increasing concentrations of **BGAz-005**. Lipids were selectively labeled with [^14^C]-acetic acid for 12 h, and cell envelope lipids were selectively removed by solvent extraction, separated by TLC (chloroform/methanol/water, 80:20:2, v/v/v), and visualized by autoradiography. (A) Equal volumes of lipids loaded adjusted for BCG growth; (B) equal counts of lipids (25,000 cpm) loaded; (C) mycolic acid methyl ester (MAME) analysis of cell-wall bound mycolates released by 5% TBAH and separated by TLC (petroleum ether/acetone, 95:5, v/v); (D) quantification of BCG lipids from panels A–C by densitometry. *M. smegmatis* cell envelope lipid analysis upon exposure to **BGAz-005**. (E) *M. smegmatis* were cultured in 7H9 broth, exposed to increasing concentrations of **BGAz-005** for 6 h and the cell envelope lipids selectively removed by solvent extraction. Equal volumes of lipid adjusted by bacterial growth were separated by TLC (chloroform/methanol/water, 80:20:2, v/v/v) and stained with MPA or (F) alpha-naphthol. (G) Equal volumes of lipid adjusted by bacterial growth were separated by TLC (hexane/diethyl ether/acetic acid), 70:30:1, v/v/v and stained with MPA. (H) Equal volumes of lipid adjusted by bacterial growth were separated by 2D-TLC (direction 1 chloroform/methanol 96:4, v/v, direction 2 toluene/acetone 80:20, v/v) and stained with MPA.

The results of transcriptomic profiling and whole cell phenotyping support the hypothesis that **BGAz-004** and **BGAz-005** inhibit aspects of cell envelope biosynthesis. To further investigate the mechanism by which mycobacterial cell envelope lipid composition is affected, actively growing cultures of BCG were exposed to increasing concentrations of **BGAz-005** followed by metabolic labeling using [^14^C]-acetic acid. Autoradiographs of cell envelope lipids separated by thin layer chromatography (TLC) revealed that treatment of BCG with **BGAz-005** at 0.5 × MIC caused a significant reduction in trehalose monomycolate (TMM) and trehalose dimycolate (TDM) and a complete loss of TMM and TDM at concentrations beyond the MIC (Figure 6A). The formation of cytoplasmic membrane phospholipids (PIMs and CL) remains unaffected (Figure 6A). The analysis of lipids loaded and separated by TLCs that had been normalized for total lipids extracted revealed an altered lipid profile, highlighting the accumulation of an unidentified lipid species that resolves to a relatively high Rf (Lipid species X, Figure 6B). The analysis of mycolic acid methyl esters (MAMES) reveals that both alpha and keto mycolates bound to the cell wall arabinogalactan (AG) are gradually depleted, as BCG is exposed to increasing concentrations of **BGAz-005** during active cell culture (Figure 6C). Quantification of the relative abundance of each lipid species highlights the significant depletion of mycolates (either conjugated to trehalose in the form of TMM/TDM or AG) when **BGAz-005** is used at a half MIC, while other lipids including PI and PIMS remain largely unaffected (Figure 6D). **BGAz-004** affects mycobacterial cell envelope lipid biosynthesis in an almost identical manner (Figure S3). The immediate and specific arrest in the biosynthesis of TMM and TDM, and as a result, loss of esterified mycolates to AG, strongly supports the hypothesis that **BGAz-004** and **BGAz-005** inhibit mycobacteria by targeting mycolate biosynthesis.

To investigate the effect of **BGAz-005** on mycobacterial cell envelope composition and to identify the composition of Lipid-X, actively growing cultures of *M. smegmatis* were exposed to a range of **BGAz-005** concentrations, which resulted in a titratable-dependent reduction in the formation of TMM and TDM as observed by staining with MPA and α-naphthol (Figure 6E/F), consistent with [^14^C]-labeling experiments performed when **BGAz-005** was exposed to BCG (Figure 6). *M. smegmatis* exposed to the highest concentration of **BGAz-005** resulted in a significant increase in the relative abundance of free mycolic acid (MA) within the cell envelope (Figure 6G); **BGAz004** affects mycobacterial cell envelope lipid biosynthesis in an almost identical manner (Figure S4). The gradual reduction of TMM and TDM abundance in *M. smegmatis* and *M. bovis* BCG is a distinct observable phenotype that occurs upon exposure to **BGAz-005** (Figures 6 and S3 and S4). The separation of solvent-extractable lipids by two-dimensional TLC provides further confirmatory evidence that the increasing abundance of Lipid-X can be directly attributed to free MA.^46^ This large increase in free mycolic acid, paralleled with the loss of TMM, TDM, and arabinogalactan-linked mycolates, illustrates that **BGAz-005** (and **BGAz-004**) compounds kill mycobacteria by arresting the final stages of mycolate biosynthesis.

### **BGAz-004** and **BGAz-005** Target Late-Stage Mycolic Acid Biosynthesis Enzymes

**Figure 7:**
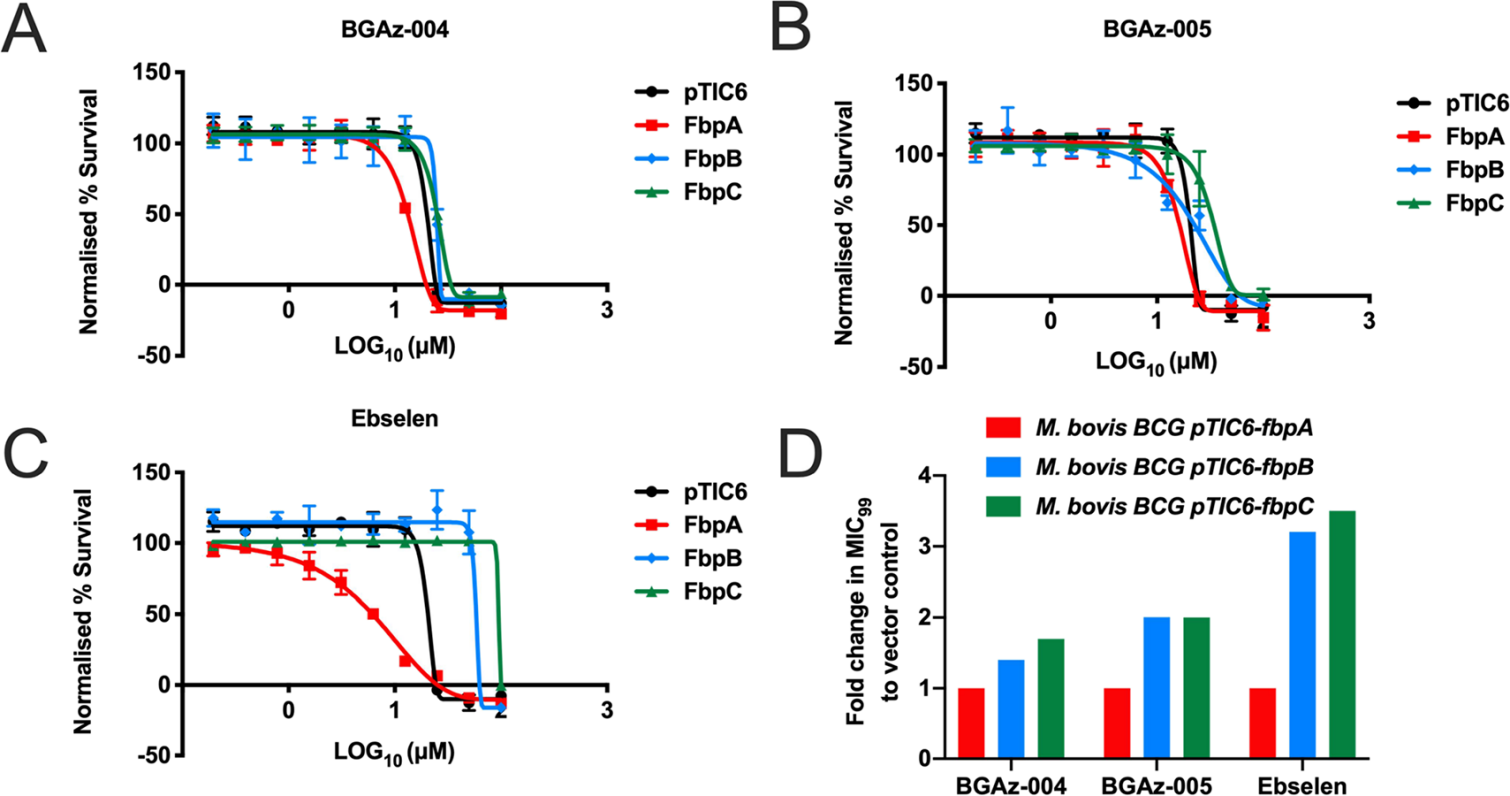
Assessing the MIC shift of **BGAz-004** and **BGAz-005** against the AG85 complex. MIC values of **BGAz-004**, **BGAz-005,** and Ebselen were determined against BCG harboring overexpression vectors and compared to empty vector controls (pTIC6) in order to identify a shift in MIC against fbpA (A), fbpB (B), and fbpC (C). Fold change in MIC shift (D). The MIC_99_ was calculated using an end point resazurin assay and the Gomperz equation for MIC determination (GraphPad Prism). Data are of triplicate repeats.

The inhibition of mycolate incorporation into the mycobacterial cell wall, supported by transcriptomic profiling, suggests that **BGAz-004** and **BGAz-005** act by targeting late-stage mycolate biosynthesis (Figures 5 and 6). The accumulation of free mycolic acid upon exposure to **BGAz-004** and **BGAz-005** at the highest concentrations suggests that mycolates are being formed but not deposited into the cell wall (Figures 6 and S4). Pks13, MmpL3, and the Ag85 complex (FbpA, FbpB, and FbpC) represent a selection of putative enzyme targets of mycolate biosynthesis that could be inhibited by **BGAz-004** and **BGAz-005**. Pks13 catalyzes the last condensation reaction in mycolate biosynthesis, condensing two fatty acids to form mycolic acids.^47^ It also plays a role in TMM formation through acylation of trehalose.^48^ Although essential in mycobacteria, *Corynebacterium glutamicum* can survive without mycolates,^47^ and so a Pks13 deletion mutant of *C. glutamicum* was utilized in this study (Supporting Information). **BGAz-005** inhibits corynemycolic acid biosynthesis in *C. glutamicum* in a manner similar to mycobacteria (Figure S6). BGAz**-002**–**BGAz-005** retained similar levels of activity in *C. glutamicum*Δ*pks13* as in the wild-type strain, implying that Pks13 is not the target (Table S2). MmpL3 is the essential membrane transporter responsible for translocating TMM across the cytoplasmic membrane.^46,49^ Treatment of *M. bovis* BCG harboring an MmpL3 overexpression vector (pMV261A-*mmpL3*)^50^ with **BGAz-002**–**BGAz-005** resulted in no shift in the MIC_99_ compared to the empty vector (pMV261A) control (Table S2). This negates MmpL3 as a potential target of the active BGAz compounds in this study, as overexpression of *mmpL3* should result in an increase in MIC as a result of increased copy number and target abundance. The Ag85 complex consists of three essential enzymes with mycolyltransferase activity, responsible for the formation of TMM, TDM, and the covalent attachment of mycolic acids to arabinogalactan.^51^ BCG harboring the plasmids pTIC6-*fbpA*, pTIC6-*fbpB*, and pTIC6-*fbpC* overexpressing Ag85A, Ag85B and Ag85C, respectively, were treated with **BGAz-004** and **BGAz-005**. A statistically significant increase in MIC was seen with FbpB and FbpC, but not FbpA (Figure 7). A two-fold increase in MIC was seen for both **BGAz-004** and **BGAz-005** upon overexpression of FbpB and FbpC, compared to the three-fold increase seen with known covalent inhibitor Ebselen.^52^ No shift in MIC was seen for FbpA upon Ebselen treatment. Furthermore, *M. smegmatis* cultured in the presence of Ebselen also resulted in a significant increase of free mycolic acid (MA) within the cell envelope and a concomitant loss of TMM and TDM (Figure S5), mirroring the lipid profiles induced by **BGAz-004** and **BGAz-005**. Collectively, these findings point toward the antigen 85 enzymes as a possible target of the active BGAz compounds of this study.

## Discussion and Conclusions

The emergence of multidrug-resistant TB means that new drugs to treat this disease are desperately required. Any new therapy should meet a number of parameters: it should be effective against MDR-TB; it should be rapidly bactericidal; it should show a novel mechanism of action; and it should possess ADME properties suitable for once-a-day oral dosing and coadministration with the current TB therapies and anti-HIV agents.^53^**BGAz-002**–**BGAz-005** display potent inhibitory activity against different mycobacterial species (including virulent and avirulent *M. tuberculosis* reference strains), as well as drug-sensitive and drug-resistant (resistant to isoniazid, rifampicin, pyrazinamide, and ethambutol) clinical isolates of *M. tuberculosis*. It is promising that **BGAz-002**–**BGAz-005** retain similar levels of activity between drug-sensitive and drug-resistant clinical isolates of *Mtb*; not only do these compounds target MDR-TB, but this also suggests that there is no cross-resistance with the current frontline drugs, indicative of a distinct mode of action. In addition to targeting an MDR strain of *Mtb*, the active BGAz compounds tested display no detectable resistance against mycobacteria in the laboratory, suggesting that the development of clinical resistance to this class of compounds will be slow to occur. Similarly, teixobactin is a cyclic undecapeptide antibiotic that elicits bactericidal activity toward clinically relevant Gram-positive pathogens, also displaying an undetectable frequency of resistance. Teixobactin has a unique mode of action; by binding simultaneously to the cell-wall biosynthetic precursors lipid II and Lipid III, this antibiotic inhibits the biosynthesis of peptidoglycan and cell-wall teichoic acids, respectively.^54^

For each of **BGAz-002**–**BGAz-005**, the MBC is within four-fold of the MIC_99_, demonstrating their bactericidal nature in BCG. Further assessment in *M. tuberculosis* by time-kill viable counts and flow cytometry confirmed the bactericidal activity of **BGAz-004** and **BGAz-005**, with **BGAz-005** being significantly bactericidal after 6 days. The significantly earlier bactericidal activity of **BGAz-005** compared to **BGAz-004** could be attributed to the increased kinetic solubility of **BGAz-005**, or it could suggest that **BGAz-005** has additional targets. While neither **BGAz-004** nor **BGAz-005** displayed bactericidal activity as rapidly as the current frontline drug isoniazid,^55^ the slower killing induced by the BGAz compounds tested may prove advantageous in preventing bacterial regrowth.^56^ Previous studies have shown that the rapid, early bactericidal activity of isoniazid results in bacterial regrowth, compared to no regrowth seen with the slower-acting bactericidal drugs rifampicin and pyrazinamide.^56^ The longer-acting bactericidal activity of the BGAz compounds tested, combined with the absence of any detectable generation of resistance, suggests that they may be superior to isoniazid, as the potential for tolerance and resistance is very low.

The comprehensive mode of action studies supports the hypothesis that the BGAz compounds target mycobacterial cell wall biosynthesis. Radiolabeled precursor incorporation studies demonstrate that **BGAz-005** specifically arrests peptidoglycan and lipid biosynthesis, and transcriptome signatures of **BGAz-004-** and **BGAz-005**-treated *M. bovis* BCG reveal significant alterations in cell wall biosynthetic genes. The mycobacterial cell wall is a well-validated and commonly occurring target among antitubercular drugs, including frontline drugs isoniazid^57^ and ethambutol,^58^ as well as ethionamide,^57^ SQ109,^59^ and d-cylcoserine.^60^ While the BGAz compounds tested in this study also inhibit cell wall biosynthesis, they do so without displaying target redundancy against current front-line drugs, such as isoniazid. Transcriptomic analysis revealed marked differences between the **BGAz-004**, **BGAz-005**, isoniazid, and ethambutol, specifically the downregulation of mycolic acid synthesis genes of the FasII system and mycolic acid synthesis and modification genes, and the lack of induction of the *efpA* efflux pump. Compared to other genome-wide transcriptional studies of antitubercular drugs,^61^ the observed differences induced by BGAz compounds suggest that they possess a unique mode of action compared to current chemotherapeutic agents.

To further probe the mechanisms by which the BGAz compounds perturb the mycobacterial cell envelope, the lipid profiles of mycobacteria exposed to increasing concentrations of **BGAz-005** were examined. A specific and rapid depletion in TMM and TDM was seen upon BGAz treatment of both *M. smegmatis* and *M. bovis* BCG, while other lipids such as cardiolipin and PIMs remained constant. The effects are more pronounced in BCG due to its increased sensitivity of radiolabeling, but in both instances, there is an almost complete arrest in TMM and TDM production by 1 × MIC of **BGAz-005**. Analysis of the cell-wall bound mycolates revealed a concurrent depletion in MAMEs. The specific loss in mycolates, both noncovalently (TMM and TDM) and covalently (MAMEs) associated, indicates that the BGAz compounds tested target mycolic acid biosynthesis. Mycolates are an essential component of the mycobacterial cell envelope and are targeted by the current drugs isoniazid and ethionamide.^62^ The BGAz compounds tested target the same biosynthetic pathway as isoniazid corroborates with the flow cytometry analysis, where the staining profiles of **BGAz-004** and **BGAz-005** were like those of isoniazid, suggesting a similar target. Further cell envelope analysis revealed a pronounced increase in free mycolic acid correlating with increasing concentration of **BGAz-005**. Typically, the relative abundance of free MA in the cell envelope of planktonically cultured mycobacteria is extremely low. However, previous studies have demonstrated that free MA levels increase significantly when mycobacteria are cultured as pellicle biofilms^63^ or as nonreplicating populations induced by gradual nutrient starvation.^64^ This simultaneous depletion of mycolates conjugated to trehalose and AG, alongside an accumulation of free mycolic acid, implies that the mycolates are being synthesized (demonstrated by the accumulation of free mycolate) but are not incorporated into the cell wall (demonstrated by the loss of TMM, TDM, and MAMEs). Thus, unlike isoniazid, the BGAz compounds tested target late-stage mycolic acid biosynthesis and have a mode of action distinct from that of isoniazid and Ethionamide, which target the early stages of mycolate production.^57^ There are several enzyme candidates involved in the latter stages of mycolic acid biosynthesis and incorporation into the cell envelope. Specifically, these encode the polyketide synthase (Pks13) responsible for the last condensation reaction in mycolate biosynthesis,^47^ the essential membrane transporter responsible for translocating TMM across the cytoplasmic membrane (MmpL3),^46,49^ and the mycolyltransferase responsible for the formation of TMM, TDM, and the covalent attachment of mycolic acids to arabinogalactan by the antigen 85 complex enzymes (FbpA, FbpB, and FbpC).^51^ Target engagement overexpression studies ruled out MmpL3 as a BGAz target, while experiments conducted in *C. glutamicum* demonstrate that Pks13 is equally not inhibited by these compounds. Guided by evidence obtained from transcriptomic profiling and cell envelope lipid analysis, further target engagement overexpression studies revealed that FbpB and FpbC afforded moderate protection to BCG exposed to **BGAz-004** or **BGAz-005**. Previous studies have identified FbpA, FbpB, and FpbC as druggable targets for the development of new antitubercular agents;^52,65−67^ however, many of these agents display unfavorable toxicological and PK/PD profiles. In this regard, the BGAz compound series demonstrates an encouraging overall toxicological profile with good absorption, low mitochondrial toxicity, and rapid clearance from hepatocytes. To reduce the potential of compound-related risk factors, a selection of mechanistic screening assays was utilized to identify hazardous and undesirable chemistry in this study. A critical example of such compound liabilities is the blocking of the hERG potassium channel. The hERG channel is a voltage-gated potassium channel that is expressed in a variety of human tissues such as the brain, thymus, adrenal gland, retina, and cardiac tissue. Any significant blocking of hERG channels by potential drug candidates could have serious off-target effects due to the dysregulation of action potential repolarisation. Furthermore, **BGAz-005** displays a remarkable pharmacokinetic profile in mice, with a blood plasma half-life exceeding 3 days that exceeds the *C*_max_ required to reach a therapeutic dose for MDR-TB. Overall, the results presented here demonstrate that the BGAz compound series represents promising novel antitubercular agents for further development. In addition to this publication, we have patented these molecules.^85^ The present invention relates to azetidine compounds and their uses. In particular, the invention relates to 1,2,4-substituted azetidine compounds and their use as antibacterial agents. This patent, we believe, gives us comprehensive intellectual property protection for our primary chemical modification.

## Experimental Section

### Chemistry at GIBH

All commercially available solvents and reagents were purchased and used without further purification. All reactions were monitored by thin layer chromatography (TLC) with silica gel-coated plates and were visualized under UV light at 254 nm or by potassium permanganate solution staining followed by heating. ^1^H NMR spectra were acquired via a Bruker AVIII300 or AVIII400 at 300 or 400 MHz, respectively, at room temperature (21 to 28 °C). ^13^C NMR spectra were recorded via a Bruker AVIII400 or AVIII500 at 101 or 126 MHz, respectively, at room temperature. ^19^F NMR spectra were recorded via a Bruker AVIII500 at 471 MHz at room temperature. Chemical shifts (δ) are reported in parts per million relatives to residual solvent for ^1^H and ^13^C NMR spectroscopy. The NMR spectral data collected thus were processed using the MestReNova-12.0.3 software package. Coupling constants (*J*) are reported in Hertz (Hz). Multiplicities of the signals are abbreviated as singlet (s), doublet (d), triplet (t), quartet (q), septet (sept), multiplet (m), and broad (br). Mass spectra were obtained on an API 2000 electrospray mass spectrometer. Infrared spectra were recorded at room temperature using a Bruker Tensor 27 FT-IR spectrometer using KBr pellets. Column chromatography purification was done using Silica Gel 200–300. Thin layer chromatography (TLC) was performed using aluminum-backed, F254-coated analytical TLC plates, which were visualized under UV light at 254 nm or by staining phosphomolybdic acid (in ethanol), followed by heating. Analytical HPLC was performed with an Agilent 1200 Series system using a Waters Acquity UPLC BEH 250 × 4.6 mm, C18, 5 μm column (solvent: MeOH-water +0.1% NH_3_H_2_O; gradient: 1 mL min^–1^; *T* = 25 °C) and UV detection at 210 nm wavelength. Purities for compounds ***BGAz-001–BGAz-005*** were >95%.

### Synthesis of **BGAz-001–BGAz-005**

Novel racemic 2,4-*cis*-amino-azetidine derivatives (**BGAz-001**–**BGAz-005**) were prepared and purified according to procedures reported for the synthesis of related compounds; for details, see Scheme S1.^17−20^ Briefly, commercially available aldehydes (**S1**) and amines (**S2**) were dissolved in methanol and heated at reflux to afford the corresponding imines (**S3**).^68−70^ Imines (**S3**) were isolated and subsequently reacted with in situ prepared allyl zinc reagent to afford homoallyl amine derivatives thereof (**S4**). The homoallyl amine derivatives (**S4**) thus obtained were dissolved in acetonitrile and treated with iodine (3 equiv) and sodium bicarbonate (5 equiv) at temperatures not exceeding 20 °C, resulting in cyclization to the corresponding 2-iodomethyl azetidine derivatives (**S5**). Displacement of iodine in derivatives **S5** by the appropriate primary or secondary amines delivered the BGAz series of compounds in four linear steps.

### Bacterial Strains and Growth Conditions

*M. smegmatismc*^2^(2)155 was cultured at 37 °C, 180 rpm in Middlebrook 7H9 media supplemented with 0.05% Tween-80 or grown on LB agar. *M. bovis* BCG (strainTice) was cultured at 37 °C and 5% CO_2_, static, in Middlebrook 7H9 media supplemented with 0.05% Tween-80 and 10% (v/v) BBL Middlebrook OADC enrichment or grown on Middlebrook 7H11 agar supplemented with 10% (v/v) BBL Middlebrook OADC enrichment.

### Determination of MIC and MBC

The minimum inhibitory concentration (MIC_99_) was determined in 96-well flat bottom, black polystyrene microtiter plates (Greiner) in a final volume of 200 μL. Compounds were two-fold serially diluted in neat DMSO and added to the microtiter plate at a final concentration of 1% DMSO. DMSO (1% in 7H9) was used as a negative control and rifampicin as a positive. The inoculum was standardized at OD600 0.05 in Middlebrook 7H9 medium and added to the plate, which was then incubated without shaking at 37 °C for 24 h (*M. smegmatis*)^84^ or 7 days (*M. bovis* BCG). Following incubation, 42 μL of resazurin (0.02% v/v in dH2O) was added to each well and incubated for a further 2 h (*M. smegmatis*) or 24 h (*M. bovis* BCG). Fluorescence was measured (Polar star omega plate reader ex 544 nm, em 590 nm), and the data were normalized using equation one. The concentration of the drug required to inhibit cell growth by 99% was calculated by nonlinear regression (Gomperz equation for MIC determination, GraphPad Prism).
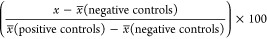
1

To determine the minimum bactericidal concentration (MBC), *M. smegmatis* and *M. bovis* BCG were grown in the presence of a two-fold serial dilution of the compound as mentioned above. After a 24-h (*M. smegmatis*) or 7-day (*M. bovis* BCG) incubation, the cells were pelleted and washed with phosphate buffered saline (PBS) pH 7.2. The washed cells were plated onto agar, devoid of compound, and incubated for 4 days (*M. smegmatis*) or 21 to 28 days *M. bovis* (BCG). The MBC was defined as the lowest concentration of compound, for which there was a 99% decrease of live bacteria compared with the inoculated amount.

### Determination of MIC_lux50_ against Autoluminescent M.tb H37Ra

AlRa^71^ (*Mtb* H37Ra::pTYOK) was homogenized with sterile glass beads in a 50 mL tube containing Middlebook 7H9 medium (5 mL) plus 0.05% Tween 80, 10% v/v oleic acid albumin dextrose catalase (OADC) supplement (7H9-OADC-Tw). When OD_600_ reached 0.3–0.5, relative light unit (RLU) counts were determined by placing culture (200 μL) on the detection hole of the luminometer. When the RLU reached 2 million/mL, the activities of compounds were assessed over a range of 3-fold increasing from 0.000001 to 10 μg/mL prepared in 25 μL AlRa broth culture (RLU diluted to 2000–4000/25 μL) grown in 7H9 broth without Tween 80. DMSO was used as negative control and isoniazid (INH, 10, 1, and 0.1 μg/mL) and rifampicin (RIF, 10, 1, and 0.1 μg/mL) were used as positive controls. RLU counts were determined four times daily (daily, i.e., days 0, 1, 2, and 3). The MIC_lux50_ was defined as determined as the lowest concentration that can inhibit >50% RLUs compared with that from the untreated controls on day 3.^72^

### Determination of MIC_99_ for Clinical M.tb Strains

The previously described resazurin microtiter assay (REMA) plate method was used.^73,74^ Compounds were two-fold serially diluted in CAMR Mycobacterium Medium MOD2 (CMM MOD2).^75^ Individual wells, in a 96-well plate, were inoculated with 1 × 10^6^ CFU mL^–1^ bacilli and incubated for 7 days at 37 °C with agitation (200 rpm). Following this, resazurin solution was added to wells (0.02% (w/v) in PBS pH 7.4, supplemented with 5% Tween 80).^76^ The 96-well plates were incubated at room temperature for 6 h. The OD_570nm_ of each well was recorded using a Tecan Sunrise plate reader. The minimum inhibitory concentration (MIC_99_) was calculated using a modified Gompertz function.^77^ The optical density measurements for each drug concentration were compared to vehicle control to determine the percentage reduction in bacterial optical density.

### Physiochemical and Toxicological Analysis

#### Kinetic Solubility

Compounds were solubilized (10 mM in DMSO) and diluted in PBS (pH 7.4) into a seven-point curve (0.2–100 μM) and incubated for 5 min at 25 °C with shaking (final DMSO concentration 1%). The turbidimetry was assessed at each of the seven concentrations using UV spectrophotometry at 620 nm, and the Log*S* was converted into the estimated solubility (*S*) using the equation *S* = 10^Log*S*^. Nicardipine hydrochloride was used as a control compound. All experiments were performed in triplicate.

#### Mouse PPB

Compounds were solubilized (10 mM in DMSO), and rapid equilibrium dialysis (RED) was used to measure the percentage binding to mouse plasma protein of the BGAz compounds at a final concentration of 5 μM. The BGAz compound was incubated in 100% mouse plasma and dialyzed against buffer in a RED device for 4 h at 37 °C in a 5% CO_2_ incubator, with continuous shaking at 200 rpm. Samples were matrix-matched and analyzed by LC-MS/MS against a six-point standard curve prepared with 100% plasma. All experiments were performed in triplicate.

#### Mouse Microsomal Clearance

Compounds were solubilized (10 mM in DMSO). 1 μM of BGAz compound was incubated with 0.5 mg/mL mouse microsomes in the presence or absence of the Phase 1 cofactor NADPH (1 mM) at 37 °C for 0, 5, 10, 15, and 30 min. The disappearance of the BGAz compound was assessed by LC-MS/MS. All experiments were performed with two replicates per compound and were validated by the inclusion of up to three species-specific control compounds. Data output consists of mean intrinsic clearance (CL_int_) and half-life (*t*_1/2_) measurements.

#### Mouse Hepatocyte Clearance

Compounds were solubilized (10 mM in DMSO). 1 μM of BGAz compound was incubated with 0.5 × 10^6^ cells/mL mouse hepatocytes at 37 °C for 0, 10, 20, 30, 45, and 60 min. The disappearance of the BGAz compound in the presence and absence of hepatocytes was assessed using LC-MS/MS. All experiments were performed with two replicates per compound and were validated by the inclusion of up to three species-specific control compounds. Data output consists of mean intrinsic clearance (CL_int_) and half-life (*t*_1/2_) measurements.

#### Caco-2 and Efflux

Compounds were solubilized (10 mM in DMSO), and the CacoReady Kit from ReadyCell S.L. (Barcelona, Spain) was used to determine compound permeability. Differentiated and polarized Caco-2 cells (21-day system) were plated on a 96-transwell permeable system as a single monolayer to allow for automated high throughput screening of compounds, and 10 μM BGAz compound was added to the system in HBSS buffer (pH 7.4) and incubated for 2 h at 37 °C in a CO_2_ incubator. Lucifer yellow was used as a cell monolayer integrity marker. Drug transport was assessed in both directions [apical to basolateral (A-B) and basolateral to apical (B-A)] across the cell monolayer. The buffer used for the assay does not include HEPES, so as to minimize the inhibitory effect on uptake transporters.^78^ The BGAz compound concentrations were quantified using a calibration curve following analysis by LC-MS/MS, and the apparent permeability coefficient (Papp) and efflux ratio of the compound across the monolayer were calculated. The efflux ratio is used as an indicator of active efflux. The permeability coefficient (*P*_app_) was calculated from the following equation:
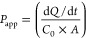
2where d*Q*/d*t* is the amount of compound in the basal (A-B) or apical (B-A) compartment as a function of time (nmol/s). *C*_0_ is the initial concentration in the donor (apical or basal) compartment (Mean of *T* = 0) (nmol/mL) and *A* is the area of the transwell (cm^2^).

The efflux ratio was then calculated as
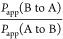
3

All experiments were performed in triplicate, and the MDR1 efflux markers Digoxin, quinidine, and propranolol were used as positive controls.

#### Pharmacokinetic Studies

Methods for combined single dosing (**BGAz001**–**005**) and dosing of single compounds multiple times (**BGAz004** and **BGAz005**) are described in detail in the Supporting Information (PK Data).

#### Cytochrome P450 Activities

Compounds were solubilized to 10 mM in DMSO. The BGAz compounds were incubated at concentrations of 0.003, 0.009, 0.03, 0.08, 0.25, 0.74, 2.2, 6.7, and 20 μM with CYP1A2, CYP2C9, CYP2C19, and CYP2D6 at 37 °C in the presence of the drug-like probe substrate HLM the Phase 1 cofactor NADPH (1 mM). The formation of metabolites of the drug-like probe substrates in the absence and presence of the BGAz compound was monitored by LC-MS/MS, and the IC_50_ value was determined. All assays had two replicates per compound and included a positive control inhibitor.

#### HepG2 Mitochondrial Dysfunction

Compounds were solubilized to 30 mM in DMSO. The BGAz compounds were added to the HepG2 cell model in a 96-well microplate in half log dilutions from 100–0.0003 μM (final DMSO concentration 0.3%) using both glucose (DMEM consisting of 25 mM glucose) and galactose (DMEM consisting of 10 mM galactose) media. The compounds were incubated with the cell line for 24 h at 37 °C in a humidified CO_2_ tissue culture incubator, followed by cell viability staining with MTT (3-(4,5-dimethylthiazol-2-yl)-2.5-diphenyltetrazolium bromide) conversion to the Formazan product, determined by absorbance measurement. The cell viability IC_50_ was determined in HepG2 glucose and metabolism-modified HepG2 galactose, and the fold change difference between the Glu/Gal IC_50_ was determined. All experiments were performed in duplicate with the mitochondrial toxicity controls rotenone and Antimycin A and the cytotoxin control tamoxifen.

#### hERG Cardiotoxicity Function

Compounds were solubilized to 30 mM in DMSO before dilution in PBS to 300 mM. A further 3-fold on-board dilution resulted in a final top BGAz compound concentration of 100 mM. Eight-point concentration–response curves were generated using 3.16-fold serial dilutions from the top test concentration. Electrophysiological recordings were made from a Chinese Hamster Ovary cell line stably expressing the full length hERG channel. Single cell ionic currents were measured in the perforated patch clamp configuration (100 μg mL^–1^ amphotericin) at room temperature (21–23 °C) using an IonWorks Quattro instrument (Molecular Devices). The internal solution contained (mM) 140 KCl, 1 MgCl_2_, 1 EGTA, and 20 HEPES and was buffered to pH 7.3. The external solution [PBS contained (mM): 138 NaCl, 2.7 KCl, 0.9 CaCl_2_, 0.5 MgCl_2_, 8 Na_2_HPO_4_, and 1.5 KH_2_PO_4_ buffered to pH 7.4]. Cells were clamped at a holding potential of −70 mV for 30 s and then stepped to +40 mV for one second. This was followed by a hyperpolarising step of 1 s to −30 mV to evoke the hERG tail current. Currents were measured from the tail step and referenced to the holding current. Compounds were then incubated for 3–4 min prior to a second measurement of the hERG signal using an identical pulsetrain.

### Mycobacterial Time Kill Experiments

**BGAz-004**, **BGAz-005,** and isoniazid were 2-fold serially diluted in 100 μL of CMM MOD2 medium from 96–3 μM, 96–3 μM, and 29.2–0.9 μM; vehicle control (0.1% DMSO) was included in all experiments. Individual wells of a 96-well microtiter plate were inoculated to a starting bacterial titer of 1 × 10^6^ CFU mL^–1^. Microtiter plates were incubated for 0, 2, 6, 10, and 14 days at 37 °C with agitation (200 rpm). The bacterial titer at each time point was enumerated via outgrowth of bacilli on solid media for 3 weeks at 37 °C via a method adapted from Miles and Misra,^27^ where triplicate 20 μL spots of bacterial culture are spotted onto Middlebrook 7H10 agar for each 10-fold serial dilution. Statistical analyses of data were performed using a factorial ANOVA and posthoc Tukey’s honestly significant difference test.

### Bacterial Staining and Flow Cytometry Analyses

The method reported by Hendon-Dunn et al.^28^ was used to analyze stained cell populations by flow cytometry. 100 μL of *M. tuberculosis* H37Rv from each antibiotic incubation, at each time point, was transferred to a microtiter plate in quadruplicate and incubated in the dark for 1 h at 37 °C with either no dye added, 20 μM calcein violet (CV-AM), 20 μM sytox green (SG), or both 20 μM CV-AM and 20 μM SG. These incubations were then spun by centrifugation, and the supernatant was removed. The cells were fixed with 4% formaldehyde (v/v in water) for 1 h. The stained bacteria were examined using a Cytoflex S (Beckman Coulter) flow cytometer. Lasers with excitatory wavelengths of 488 and 405 nm were used. SG fluorescence emission (excitation and emission, 488 and 523 nm, respectively) was detected in the FIT-C channel (530/40 BP), and CV-AM fluorescence (excitation and emission, 400 and 452 nm, respectively) was detected in the PB-450 channel (450/50 BP). A quadrant gating strategy was used;^28^ briefly, 10,000 single-cell events were gated upon using a two-parameter dot plot of forward scatter height versus forward scatter area. From gated single cells, the percentages of the total cell population residing in each polygonal population gate (P1: CV-AM^–^/SG^–^, P2: CV-AM^+^/SG^–^, P3: CV-AM^+^/SG^+^, P4: CV-AM^–^/SG^+^) were obtained. Statistical analysis of data was performed using a Student’s *t-*test.

### Inhibition of Macromolecular Synthesis

Inhibition of macromolecular biosynthesis was assayed by measuring the incorporation of radiolabeled precursors into DNA, RNA, protein, peptidoglycan, and fatty acids in the 10% trichloroacetic acid (TCA) extracts of cells exposed to azetidines. *M. smegmatis* were cultured in 7H9 media supplemented with 0.05% Tween-80 and grown to an OD_600_ of 0.4. Cultures (5 mL) were then transferred into sterile glass tubes and preincubated with 0× , 0.5× , 0.75× and 1 × MIC_99_ azetidines for 1 h at 37 °C with shaking. After preincubation, 10 μL of 500 nCi/μL [*methyl-*^3^H]thymidine, 500 nCi/μL [5,6-^3^H]uridine, 500 nCi/μL l-[4,5-^3^H]leucine, 500 nCi/μL [^3^H]*meso-*diaminopimelic acid, and 500 nCi/μL [^14^C]acetic acid were added to cultures to measure synthesis of DNA, RNA, protein, peptidoglycan and lipids, respectively. All cultures were incubated for 36 h, and 100 μL samples were sacrificed at 6, 12, 24, and 36 h time points by the addition of 50 μL 30%TCA/70% ethanol in Eppendorf tubes. Tubes were incubated at room temperature for 60 min to allow for precipitation of macromolecular material. Samples were individually vacuum filtered using 0.025 μm membrane filters (VSWP01300, MF-Millipore) that were prewashed with 500 μL 70% ethanol. Samples were washed with 3 × 500 μL 5% TCA, followed by 2 × 95% ethanol. Filter papers were dried and combined with 5 mL of scintillation fluid before measuring radioactive counts.

### Transcriptomic Profiling by RNA-seq Analysis

*M. bovis* BCG was cultured to an OD_600_ of 0.4 before exposure to 1 × MIC_99_ concentrations of **BGAz-004** or **BGAz-005** for 8 h in three biological replicates and then compared to carrier control-treated bacilli. Cells were pelleted, flash-frozen in liquid nitrogen, and stored at −80 °C. Pellets were resuspended in lysozyme (600 μL, 5 mg/mL) and β-mercaptoethanol (7 μL/mL) in TE buffer and lysed by bead beating at 6 m/min (1 × 45 s). Samples were subjected to further bead beating (3 × 45 s), following the addition of (60 μL, 10% SDS). Sodium acetate pH 5.2 (3 M, 60 μL) and acidified phenol pH 4.2 (726 μL) were added, and the tubes were mixed well by inversion. Samples were incubated at 65 °C for 5 min and centrifuged for 5 min at 18,000 × *g*. The upper aqueous phase was transferred to a fresh tube, and an equal volume of acid phenol pH 4.2 was added and mixed well by inversion. Following heating (65 °C for 2 min) and centrifugation (5 min at 18,000 × *g*), the upper aqueous phase was once more transferred to a fresh tube, and an equal volume of chloroform:isoamyl alcohol (24:1 v/v) was added. The sample was mixed well by inversion and centrifuged at 18,000 × *g* for 5 min. The upper aqueous phase was transferred to a fresh tube, and a 1/10 volume of sodium acetate (3 M, pH 5.2) and three volumes of 100% ethanol were added. Samples were incubated at −20 °C overnight, centrifuged for 10 min (4 °C, 14,000 × *g*), and the supernatant removed. Ethanol (70% in water, 500 μL) was added to the pellet and centrifuged for 10 min (4 °C, 14,000 × *g*). The supernatant was removed, the pellet air-dried, and the extracted RNA resuspended in of RNase-free dH_2_O (40 μL). DNase treatment was performed using the TURBO DNA-*free* kit (Invitrogen). Briefly, a 0.1 × volume of 10 × TURBO DNase buffer was added to the RNA, along with TURBO DNase enzyme (1 μL of enzyme stock). The sample was incubated for 30 min at 37 °C, an additional TURBO DNase enzyme (1 μL of enzyme) was added, and the sample was incubated for another 30 min. DNase inactivation reagent (0.2 volumes) was added, and the sample was incubated at room temperature for 5 min. Following centrifugation at 10,000 × *g* for 1.5 min, the supernatant containing the RNA was transferred to a fresh tube and stored at −80 °C. The purified RNA was quantified, depleted of rRNA, and library-prepped before sequencing by Illumina HiSeq (150 × 2 paired end) by Genewiz Ltd. Adapter sequences and poor-quality reads were removed using Trimmomatic v.0.36, before mapping to the *Mycobacterium bovis* BCG Pasteur 1173P2 genome using Bowtie2 aligner v.2.2.6. Gene expression was quantified using FeatureCounts from the Subread package v.1.5.2. Differentially expressed genes were identified with the DESeq2 R package, normalized by the RLE method, and using the Wald test with Benjamini and Hochberg multiple testing correction. Genes with an adjusted *p*-value <0.05 and log2 fold change >1 were considered to be differentially expressed. Significantly enriched signatures, with updated genome annotation,^79,80^ were identified using the hypergeometric function comparing to published drug responses^41,42^ or mapped to metabolic pathways.^35,36^ Genes significantly differentially expressed in response to **BGAz-004** and **BGAz-005** are detailed in Supporting Information. Fully annotated RNA-seq data will be deposited in ArrayExpress; the accession number will be provided.

### Radioisotope Labeling of Lipids and Analysis

Cells were grown to an OD600 0.5, treated with compound, and grown for 6 h (*M. smegmatis*) or overnight (*M. bovis* BCG). For radio labeling experiments, 1 μCi mL^–1 14^C acetic acid was then added, followed by a 16-h incubation. Cells were harvested and extracted using chloroform:methanol:water (10:10:3, v/v/v, 2 mL) for 2 h at 50 °C. Following centrifugation, the organic extracts were combined with chloroform and water (1.75 and 0.75 mL respectively). The lower organic phase containing associated lipids was recovered, washed twice with chloroform:methanol:water (3:47:48, v/v/v, 2 mL), and dried with nitrogen gas. Samples were resuspended in chloroform:methanol (2:1, v/v, 200 μL), and OD-adjusted volumes were subjected to thin-layer chromatography (TLC) analysis. Cell wall-associated lipids were visualized by either heating TLC plates after treatment with molybdophosphoric acid (MPA) in ethanol (5% w/v) or alpha-naphthol in ethanol (5% w/v), or by autoradiography by exposure to Kodak BioMax MR film.

Cell wall-bound lipids from the delipidated extracts from the abovementioned extraction were released by the addition of a solution of tetra-butyl ammonium hydroxide (TBAH) (5% m/v, 2 mL), followed by a 16-h incubation at 100 °C. Water (2 mL), dichloromethane (4 mL), and iodomethane (200 μL) were then added and mixed thoroughly for 30 min. The organic phase was recovered, following centrifugation, and washed with water (3 × 4 mL), dried, and resuspended in diethyl ether (4 mL). After sonication and centrifugation, the supernatant was dried and resuspended in dichloromethane. Equivalent aliquots of the samples were subjected to TLC in petroleum ether:acetone (95:5, v/v) and visualized by MPA and heat or autoradiography.

### Target Gene Overexpression Studies

The constructs pMV261_*mmpL3* and pVV16_*trpAB*,^50,81^ including the empty pMV261 and pVV16 vectors, were electroporated into *M. bovis* BCG as previously described, and the MIC_99_ determined as described above. The constructs pTIC6_*fbpA,* pTIC6_*fbpB,* and pTIC6_*fbpC* were synthesized by GenScript Ltd. by inserting the coding regions of the *M. tuberculosis* H37Rv genes into the vector pTIC6, which encodes kanamycin selection. The constructs and empty pTIC6 vector were electroporated into *M. bovis* BCG. Following induction of gene expression with 50 ng/mL of anhydrotetracycline for 24 h, the MIC_99_ was determined as described above.

## Supporting Information Available

Supplementary PK data (PDF).
A detailed description of author contributions, general methods, chemical and biological general procedures, synthetic chemistry protocols for the synthesis of **BGAz001**–**BGAz0016**, NMR spectra, details of mass spectrometry analysis, additional corresponding references,^82,83^ and molecular formula strings (ZIP).

## Abbreviations used

AG: Arabinogalactan
Ag85: antigen 85
BGAz: azetidine derivate
MmpL3: mycobacterial membrane protein Large 3
PKs13: polyketide synthase 13
TDM: trehalose dimycolate
TMM: trehalose monomycolate
WGS: whole genome sequencing.

## References

[ref1] www.who.int/gho/tb/en/. World Health Organisation: Global Health Observatory (GHO) data www.who.int/gho/tb/en/.

[ref2] Global tuberculosis report 2022; World Health Organization: Geneva, 2022, Licence: CC BY-NC-SA 3.0 IGO.

[ref3] Combs D. L.; O’Brien R. J.; Geiter L. J. USPHS Tuberculosis Short-Course Chemotherapy Trial 21: Effectiveness, Toxicity, and Acceptability: The Report of Final Results. Ann. Int. Med. 1990, 112, 397–406. 10.7326/0003-4819-76-3-112-6-397. 2155569

[ref4] Shah N. S.; Wright A.; Bai G. H.; Barrera L.; Boulahbal F.; Martin-Casabona N.; Drobniewski F.; Gilpin C.; Havelkova M.; Lepe R.; Lumb R.; Metchock B.; Portaels F.; Rodrigues M. F.; Rusch-Gerdes S.; Van Deun A.; Vincent V.; Laserson K.; Wells C.; Cegielski J. P. Worldwide emergence of extensively drug-resistant tuberculosis. Emerg. Infect. Dis. 2007, 13, 380–7. 10.3201/eid1303.061400. 17552090 PMC2725916

[ref5] Sharma S. K.; Mohan A. Multidrug-Resistant Tuberculosis: A Menace That Threatens To Destabilize Tuberculosis Control. Chest 2006, 130, 261–272. 10.1016/S0012-3692(15)50981-1. 16840411

[ref6] Zumla A.; Nahid P.; Cole S. T. Advances in the development of new tuberculosis drugs and treatment regimens. Nat. Rev. Drug Discovery 2013, 12, 388–404. 10.1038/nrd4001. 23629506

[ref7] Ma Z.; Lienhardt C.; McIlleron H.; Nunn A. J.; Wang X. Global tuberculosis drug development pipeline: the need and the reality. Lancet 2010, 375, 2100–2109. 10.1016/S0140-6736(10)60359-9. 20488518

[ref8] Sharma A.; De Rosa M.; Singla N.; Singh G.; Barnwal R. P.; Pandey A. Tuberculosis: An Overview of the Immunogenic Response, Disease Progression, and Medicinal Chemistry Efforts in the Last Decade toward the Development of Potential Drugs for Extensively Drug-Resistant Tuberculosis Strains. J. Med. Chem. 2021, 64, 4359–4395. 10.1021/acs.jmedchem.0c01833. 33826327

[ref9] Raymer B.; Bhattacharya S. K. Lead-like Drugs: A Perspective. J. Med. Chem. 2018, 61, 10375–10384. 10.1021/acs.jmedchem.8b00407. 30052440

[ref10] Lovering F. Escape from Flatland 2: complexity and promiscuity. MedChemComm 2013, 4, 515–519. 10.1039/c2md20347b.

[ref11] Lovering F.; Bikker J.; Humblet C. Escape from Flatland: Increasing Saturation as an Approach to Improving Clinical Success. J. Med. Chem. 2009, 52, 6752–6756. 10.1021/jm901241e. 19827778

[ref12] Yang Y.; Chen H.; Nilsson I.; Muresan S.; Engkvist O. Investigation of the Relationship between Topology and Selectivity for Druglike Molecules. J. Med. Chem. 2010, 53, 7709–7714. 10.1021/jm1008456. 20942392

[ref13] Baell J. B.; Holloway G. A. New Substructure Filters for Removal of Pan Assay Interference Compounds (PAINS) from Screening Libraries and for Their Exclusion in Bioassays. J. Med. Chem. 2010, 53, 2719–2740. 10.1021/jm901137j. 20131845

[ref14] Baell J. B.; Nissink J. W. M. Seven Year Itch: Pan-Assay Interference Compounds (PAINS) in 2017—Utility and Limitations. ACS Chem. Biol. 2018, 13, 36–44. 10.1021/acschembio.7b00903. 29202222 PMC5778390

[ref15] Colomer I.; Empson C. J.; Craven P.; Owen Z.; Doveston R. G.; Churcher I.; Marsden S. P.; Nelson A. A divergent synthetic approach to diverse molecular scaffolds: assessment of lead-likeness using LLAMA, an open-access computational tool. Chem. Commun. 2016, 52, 7209–7212. 10.1039/C6CC03244C. 27145833

[ref16] Synthetic chemistry methodology projects within the School of Chemistry at the University of Birmingham initially provided ∼ 200 compounds suitable for biological screening.

[ref17] Feula A.; Dhillon S. S.; Byravan R.; Sangha M.; Ebanks R.; Hama Salih M. A.; Spencer N.; Male L.; Magyary I.; Deng W.-P.; Müller F.; Fossey J. S. Synthesis of azetidines and pyrrolidines *via* iodocyclisation of homoallyl amines and exploration of activity in a zebrafish embryo assay. Org. Biomol. Chem. 2013, 11, 5083–5093. 10.1039/c3ob41007b. 23824110

[ref18] Feula A.; Male L.; Fossey J. S. Diastereoselective preparation of azetidines and pyrrolidines. Org. Lett. 2010, 12, 5044–5047. 10.1021/ol102215e. 20879778

[ref19] Yoshizawa A.; Feula A.; Leach A. G.; Male L.; Fossey J. S. Palladium and Platinum 2,4-*cis*-amino Azetidine and Related Complexes. Front. Chem. 2018, 6, 211 10.3389/fchem.2018.00211. 29977888 PMC6021532

[ref20] Yoshizawa A.; Feula A.; Male L.; Leach A. G.; Fossey J. S. Rigid and concave, 2,4-*cis*-substituted azetidine derivatives: A platform for asymmetric catalysis. Sci. Rep. 2018, 8, 6541 10.1038/s41598-018-24784-3. 29695806 PMC5916886

[ref21] www.birmingham.ac.uk/facilities/bddf. Birmingham Drug Discovery Facility. www.birmingham.ac.uk/facilities/bddf.

[ref22] The synthesis and preliminary screening data of ∼ 100 azetidine derivatives that were less active, and not investigated further, will be reported elsewhere.

[ref23] Palomino J.-C.; Martin A.; Camacho M.; Guerra H.; Swings J.; Portaels F. Resazurin Microtiter Assay Plate: Simple and Inexpensive Method for Detection of Drug Resistance in *Mycobacterium tuberculosis*. Antimicrob. Agents Chemother. 2002, 46, 2720 10.1128/AAC.46.8.2720-2722.2002. 12121966 PMC127336

[ref24] Yang F.; Njire M. M.; Liu J.; Wu T.; Wang B.; Liu T.; Cao Y.; Liu Z.; Wan J.; Tu Z.; Tan Y.; Tan S.; Zhang T. Engineering more stable, selectable marker-free autoluminescent mycobacteria by one step. PLoS One 2015, 10, e0119341 10.1371/journal.pone.0119341. 25760622 PMC4356594

[ref25] Qin L.; Wang J.; Lu J.; Yang H.; Zheng R.; Liu Z.; Huang X.; Feng Y.; Hu Z.; Ge B. A deletion in the RD105 region confers resistance to multiple drugs in *Mycobacterium tuberculosis*. BMC Biol. 2019, 17, 7 10.1186/s12915-019-0628-6. 30683096 PMC6347829

[ref26] Hoagland D. T.; Liu J.; Lee R. B.; Lee R. E. New agents for the treatment of drug-resistant *Mycobacterium tuberculosis*. Adv. Drug Delivery Rev. 2016, 102, 55–72. 10.1016/j.addr.2016.04.026. PMC4903924 27151308

[ref27] Miles A. A.; Misra S. S.; Irwin J. O. The estimation of the bactericidal power of the blood. Epidemiol. Infect. 1938, 38, 732–749. 10.1017/S002217240001158X. PMC2199673 20475467

[ref28] Hendon-Dunn C. L.; Doris K. S.; Thomas S. R.; Allnutt J. C.; Marriott A. A. N.; Hatch K. A.; Watson R. J.; Bottley G.; Marsh P. D.; Taylor S. C.; Bacon J. A Flow Cytometry Method for Rapidly Assessing *Mycobacterium tuberculosis* Responses to Antibiotics with Different Modes of Action. Antimicrob. Agents Chemother. 2016, 60, 3869 10.1128/AAC.02712-15. 26902767 PMC4914659

[ref29] Abrahams K. A.; Cox J. A. G.; Spivey V. L.; Loman N. J.; Pallen M. J.; Constantinidou C.; Fernandez R.; Alemparte C.; Remuinan M. J.; Barros D.; Ballell L.; Besra G. S. Identification of Novel Imidazo[1,2-*a*]pyridine Inhibitors Targeting *M. tuberculosis* QcrB. PLoS One 2012, 7, e52951 10.1371/journal.pone.0052951. 23300833 PMC3534098

[ref30] Abrahams K. A.; Chung C.-W.; Ghidelli-Disse S.; Rullas J.; Rebollo-López M. J.; Gurcha S. S.; Cox J. A. G.; Mendoza A.; Jiménez-Navarro E.; Martínez-Martínez M. S.; Neu M.; Shillings A.; Homes P.; Argyrou A.; Casanueva R.; Loman N. J.; Moynihan P. J.; Lelièvre J.; Selenski C.; Axtman M.; Kremer L.; Bantscheff M.; Angulo-Barturen I.; Izquierdo M. C.; Cammack N. C.; Drewes G.; Ballell L.; Barros D.; Besra G. S.; Bates R. H. Identification of KasA as the cellular target of an anti-tubercular scaffold. Nat. Commun. 2016, 7, 12581 10.1038/ncomms12581. 27581223 PMC5025758

[ref31] Andries K.; Verhasselt P.; Guillemont J.; Göhlmann H. W. H.; Neefs J.-M.; Winkler H.; Van Gestel J.; Timmerman P.; Zhu M.; Lee E.; Williams P.; de Chaffoy D.; Huitric E.; Hoffner S.; Cambau E.; Truffot-Pernot C.; Lounis N.; Jarlier V. A Diarylquinoline Drug Active on the ATP Synthase of *Mycobacterium tuberculosis*. Science 2005, 307, 223 10.1126/science.1106753. 15591164

[ref32] Batt S. M.; Cacho Izquierdo M.; Castro Pichel J.; Stubbs C. J.; Vela-Glez Del Peral L.; Pérez-Herrán E.; Dhar N.; Mouzon B.; Rees M.; Hutchinson J. P.; Young R. J.; McKinney J. D.; Barros Aguirre D.; Ballell L.; Besra G. S.; Argyrou A. Whole Cell Target Engagement Identifies Novel Inhibitors of *Mycobacterium tuberculosis* Decaprenylphosphoryl-β-d-ribose Oxidase. ACS Infec. Dis. 2015, 1, 615–626. 10.1021/acsinfecdis.5b00065. 27623058

[ref33] Manganelli R.; Voskuil M. I.; Schoolnik G. K.; Smith I. The *Mycobacterium tuberculosis* ECF sigma factor sigmaE: role in global gene expression and survival in macrophages. Mol. Microbiol. 2001, 41, 423–37. 10.1046/j.1365-2958.2001.02525.x. 11489128

[ref34] Balazsi G.; Heath A. P.; Shi L.; Gennaro M. L. The temporal response of the *Mycobacterium tuberculosis* gene regulatory network during growth arrest. Mol. Syst. Biol. 2008, 4, 225 10.1038/msb.2008.63. 18985025 PMC2600667

[ref35] Karp P. D.; Billington R.; Caspi R.; Fulcher C. A.; Latendresse M.; Kothari A.; Keseler I. M.; Krummenacker M.; Midford P. E.; Ong Q.; Ong W. K.; Paley S. M.; Subhraveti P. The BioCyc collection of microbial genomes and metabolic pathways. Brief. Bioinform. 2019, 20, 1085–1093. 10.1093/bib/bbx085. 29447345 PMC6781571

[ref36] Caspi R.; Billington R.; Ferrer L.; Foerster H.; Fulcher C. A.; Keseler I. M.; Kothari A.; Krummenacker M.; Latendresse M.; Mueller L. A.; Ong Q.; Paley S.; Subhraveti P.; Weaver D. S.; Karp P. D. The MetaCyc database of metabolic pathways and enzymes and the BioCyc collection of pathway/genome databases. Nucleic Acids Res. 2016, 44, D471–80. 10.1093/nar/gkv1164. 26527732 PMC4702838

[ref37] Chang Y.; Fox B. G. Identification of Rv3230c as the NADPH oxidoreductase of a two-protein DesA3 acyl-CoA desaturase in *Mycobacterium tuberculosis* H37Rv. Biochemistry 2006, 45, 13476–86. 10.1021/bi0615285. 17087501 PMC2547085

[ref38] McGillivray A.; Golden N. A.; Gautam U. S.; Mehra S.; Kaushal D. The *Mycobacterium tuberculosis* Rv2745c plays an important role in responding to redox stress. PLoS One 2014, 9, e93604 10.1371/journal.pone.0093604. 24705585 PMC3976341

[ref39] Hards K.; Robson J. R.; Berney M.; Shaw L.; Bald D.; Koul A.; Andries K.; Cook G. M. Bactericidal mode of action of bedaquiline. J. Antimicrob. Chemother. 2015, 70, 2028–2037. 10.1093/jac/dkv054. 25754998

[ref40] Mishra S.; Shukla P.; Bhaskar A.; Anand K.; Baloni P.; Jha R. K.; Mohan A.; Rajmani R. S.; Nagaraja V.; Chandra N.; Singh A. Efficacy of β-lactam/β-lactamase inhibitor combination is linked to WhiB4-mediated changes in redox physiology of *Mycobacterium tuberculosis*. eLife 2017, 6, e25624 10.7554/eLife.25624. 28548640 PMC5473688

[ref41] Waddell S. J.; Stabler R. A.; Laing K.; Kremer L.; Reynolds R. C.; Besra G. S. The use of microarray analysis to determine the gene expression profiles of *Mycobacterium tuberculosis* in response to anti-bacterial compounds. Tuberculosis 2004, 84, 263–74. 10.1016/j.tube.2003.12.005. 15207496 PMC7016511

[ref42] Boshoff H. I.; Myers T. G.; Copp B. R.; McNeil M. R.; Wilson M. A.; Barry C. E. 3rd The transcriptional responses of *Mycobacterium tuberculosis* to inhibitors of metabolism: Novel insights into drug mechanisms of action. J. Biol. Chem. 2004, 279, 40174–84. 10.1074/jbc.M406796200. 15247240

[ref43] Amaral L.; Viveiros M. Thioridazine: A Non-Antibiotic Drug Highly Effective, in Combination with First Line Anti-Tuberculosis Drugs, against Any Form of Antibiotic Resistance of *Mycobacterium tuberculosis* Due to Its Multi-Mechanisms of Action. Antibiotics 2017, 6, 3 10.3390/antibiotics6010003. 28098814 PMC5372983

[ref44] Lee R. E.; Protopopova M.; Crooks E.; Slayden R. A.; Terrot M.; Barry C. E. 3rd Combinatorial lead optimization of [1,2]-diamines based on ethambutol as potential antituberculosis preclinical candidates. J. Comb. Chem. 2003, 5, 172–87. 10.1021/cc020071p. 12625709

[ref45] Makarov V.; Manina G.; Mikusova K.; Möllmann U.; Ryabova O.; Saint-Joanis B.; Dhar N.; Pasca M. R.; Buroni S.; Lucarelli A. P.; Milano A.; De Rossi E.; Belanova M.; Bobovska A.; Dianiskova P.; Kordulakova J.; Sala C.; Fullam E.; Schneider P.; McKinney J. D.; Brodin P.; Christophe T.; Waddell S.; Butcher P.; Albrethsen J.; Rosenkrands I.; Brosch R.; Nandi V.; Bharath S.; Gaonkar S.; Shandil R. K.; Balasubramanian V.; Balganesh T.; Tyagi S.; Grosset J.; Riccardi G.; Cole S. T. Benzothiazinones Kill *Mycobacterium tuberculosis* by Blocking Arabinan Synthesis. Science 2009, 324, 801 10.1126/science.1171583. 19299584 PMC3128490

[ref46] Xu Z.; Meshcheryakov V. A.; Poce G.; Chng S.-S. MmpL3 is the flippase for mycolic acids in mycobacteria. Proc. Natl. Acad. Sci. U.S.A. 2017, 114, 7993 10.1073/pnas.1700062114. 28698380 PMC5544280

[ref47] Portevin D.; de Sousa D.; Auria C.; Houssin C.; Grimaldi C.; Chami M.; Daffé M.; Guilhot C. A polyketide synthase catalyzes the last condensation step of mycolic acid biosynthesis in mycobacteria and related organisms. Proc. Natl. Acad. Sci. U. S. A. 2004, 101, 314 10.1073/pnas.0305439101. 14695899 PMC314182

[ref48] Gavalda S.; Bardou F.; Laval F.; Bon C.; Malaga W.; Chalut C.; Guilhot C.; Mourey L.; Daffé M.; Quémard A. The Polyketide Synthase Pks13 Catalyzes a Novel Mechanism of Lipid Transfer in Mycobacteria. Chem. Biol. 2014, 21, 1660–1669. 10.1016/j.chembiol.2014.10.011. 25467124

[ref49] Su C.-C.; Klenotic P. A.; Bolla J. R.; Purdy G. E.; Robinson C. V.; Yu E. W. MmpL3 is a lipid transporter that binds trehalose monomycolate and phosphatidylethanolamine. Proc. Natl. Acad. Sci. U.S.A. 2019, 116, 11241 10.1073/pnas.1901346116. 31113875 PMC6561238

[ref50] Cox J. A. G.; Abrahams K. A.; Alemparte C.; Ghidelli-Disse S.; Rullas J.; Angulo-Barturen I.; Singh A.; Gurcha S. S.; Nataraj V.; Bethell S.; Remuiñán M. J.; Encinas L.; Jervis P. J.; Cammack N. C.; Bhatt A.; Kruse U.; Bantscheff M.; Fütterer K.; Barros D.; Ballell L.; Drewes G.; Besra G. S. THPP target assignment reveals EchA6 as an essential fatty acid shuttle in mycobacteria. Nat. Microbiol. 2016, 1, 15006 10.1038/nmicrobiol.2015.6. 27571973

[ref51] Belisle J. T.; Vissa V. D.; Sievert T.; Takayama K.; Brennan P. J.; Besra G. S. Role of the Major Antigen of *Mycobacterium tuberculosis* in Cell Wall Biogenesis. Science 1997, 276, 1420 10.1126/science.276.5317.1420. 9162010

[ref52] Favrot L.; Grzegorzewicz A. E.; Lajiness D. H.; Marvin R. K.; Boucau J.; Isailovic D.; Jackson M.; Ronning D. R. Mechanism of inhibition of *Mycobacterium tuberculosis* antigen 85 by ebselen. Nat. Commun. 2013, 4, 2748 10.1038/ncomms3748. 24193546 PMC4049535

[ref53] Mdluli K.; Kaneko T.; Upton A. The tuberculosis drug discovery and development pipeline and emerging drug targets. Cold Spring Harb. Perspect. Med. 2015, 5, a021154 10.1101/cshperspect.a021154. 25635061 PMC4448709

[ref54] Ling L. L.; Schneider T.; Peoples A. J.; Spoering A. L.; Engels I.; Conlon B. P.; Mueller A.; Schäberle T. F.; Hughes D. E.; Epstein S.; Jones M.; Lazarides L.; Steadman V. A.; Cohen D. R.; Felix C. R.; Fetterman K. A.; Millett W. P.; Nitti A. G.; Zullo A. M.; Chen C.; Lewis K. A new antibiotic kills pathogens without detectable resistance. Nature 2015, 517, 455–459. 10.1038/nature14098. 25561178 PMC7414797

[ref55] Isoniazid was included as a positive control (0.9–29.2 μM), and comparison, with it being an antibiotic that targets the cell wall and has rapid bactericidal activity.

[ref56] Hendon-Dunn C. L.; Pertinez H.; Marriott A. A. N.; Hatch K. A.; Allnutt J. C.; Davies G.; Bacon J. Regrowth of *Mycobacterium tuberculosis* Populations Exposed to Antibiotic Combinations Is Due to the Presence of Isoniazid and Not Bacterial Growth Rate. Antimicrob. Agents Chemother. 2019, 63, e00570-19 10.1128/AAC.00570-19. 31527023 PMC6879242

[ref57] Banerjee A.; Dubnau E.; Quemard A.; Balasubramanian V.; Um K. S.; Wilson T.; Collins D.; de Lisle G.; Jacobs W. R. inhA, a gene encoding a target for isoniazid and ethionamide in *Mycobacterium tuberculosis*. Science 1994, 263, 227 10.1126/science.8284673. 8284673

[ref58] Takayama K.; Kilburn J. O. Inhibition of synthesis of arabinogalactan by ethambutol in *Mycobacterium smegmatis*. Antimicrob. Agents Chemother. 1989, 33, 1493 10.1128/AAC.33.9.1493. 2817850 PMC172689

[ref59] Tahlan K.; Wilson R.; Kastrinsky D. B.; Arora K.; Nair V.; Fischer E.; Barnes S. W.; Walker J. R.; Alland D.; Barry C. E.; Boshoff H. I. SQ109 Targets MmpL3, a Membrane Transporter of Trehalose Monomycolate Involved in Mycolic Acid Donation to the Cell Wall Core of *Mycobacterium tuberculosis*. Antimicrob. Agents Chemother. 2012, 56, 1797 10.1128/AAC.05708-11. 22252828 PMC3318387

[ref60] Prosser G. A.; de Carvalho L. P. S. Kinetic mechanism and inhibition of *Mycobacterium tuberculosis* D-alanine:D-alanine ligase by the antibiotic D-cycloserine. FEBS Journal 2013, 280, 1150–1166. 10.1111/febs.12108. 23286234

[ref61] Briffotaux J.; Liu S.; Gicquel B. Genome-Wide Transcriptional Responses of *Mycobacterium* to Antibiotics. Front. Microbiol. 2019, 10, 249 10.3389/fmicb.2019.00249. 30842759 PMC6391361

[ref62] Johnsson K.; King D. S.; Schultz P. G. Studies on the Mechanism of Action of Isoniazid and Ethionamide in the Chemotherapy of Tuberculosis. J. Am. Chem. Soc. 1995, 117, 5009–5010. 10.1021/ja00122a038.

[ref63] Milanes C. L.; Pernalete N.; Starosta R.; Perez-Gonzalez M.; Paz-Martinez V.; Bellorin-Font E. Altered response of adenylate cyclase to parathyroid hormone during compensatory renal growth. Kidney Int. 1989, 36, 802–9. 10.1038/ki.1989.265. 2615189

[ref64] Bacon J.; Alderwick L. J.; Allnutt J. A.; Gabasova E.; Watson R.; Hatch K. A.; Clark S. O.; Jeeves R. E.; Marriott A.; Rayner E.; Tolley H.; Pearson G.; Hall G.; Besra G. S.; Wernisch L.; Williams A.; Marsh P. D. Non-replicating *Mycobacterium tuberculosis* elicits a reduced infectivity profile with corresponding modifications to the cell wall and extracellular matrix. PLoS One 2014, 9, e87329 10.1371/journal.pone.0087329. 24516549 PMC3916317

[ref65] Rose J. D.; Maddry J. A.; Comber R. N.; Suling W. J.; Wilson L. N.; Reynolds R. C. Synthesis and biological evaluation of trehalose analogs as potential inhibitors of mycobacterial cell wall biosynthesis. Carbohydr. Res. 2002, 337, 105–120. 10.1016/S0008-6215(01)00288-9. 11814442

[ref66] Barry C. S.; Backus K. M.; Barry C. E.; Davis B. G. ESI-MS Assay of *M. tuberculosis* Cell Wall Antigen 85 Enzymes Permits Substrate Profiling and Design of a Mechanism-Based Inhibitor. J. Am. Chem. Soc. 2011, 133, 13232–13235. 10.1021/ja204249p. 21776980 PMC11042539

[ref67] Viljoen A.; Richard M.; Nguyen P. C.; Fourquet P.; Camoin L.; Paudal R. R.; Gnawali G. R.; Spilling C. D.; Cavalier J.-F.; Canaan S.; Blaise M.; Kremer L. Cyclipostins and cyclophostin analogs inhibit the antigen 85C from *Mycobacterium tuberculosis* both *in vitro* and *in vivo*. J. Biol. Chem. 2018, 293, 2755–2769. 10.1074/jbc.RA117.000760. 29301937 PMC5827452

[ref68] Crespo M.; Martinez M.; de Pablo E. Activation volumes for intramolecular oxidative C–X (X = H, F, Cl or Br) addition to platinum(II) imine complexes as a proof of the intimate mechanism. J. Chem. Soc., Dalton Trans. 1997, 1231–1236. 10.1039/a606872c.

[ref69] Clark P. W.; Dyke S. F.; Smith G.; Kennard C. H. L. The cyclopalladation of benzylidenebenzylamines. J. Organomet. Chem. 1987, 330, 447–460. 10.1016/S0022-328X(00)99057-0.

[ref70] Anderson C. M.; Crespo M.; Kfoury N.; Weinstein M. A.; Tanski J. M. Regioselective C–H Activation Preceded by C_sp_2–C_sp_3 Reductive Elimination from Cyclometalated Platinum(IV) Complexes. Organometallics 2013, 32, 4199–4207. 10.1021/om400398g.

[ref71] Zhang T.; Li S.-Y.; Nuermberger E. L. Autoluminescent *Mycobacterium tuberculosis* for Rapid, Real-Time, Non-Invasive Assessment of Drug and Vaccine Efficacy. PLoS One 2012, 7, e29774 10.1371/journal.pone.0029774. 22253776 PMC3256174

[ref72] Liu Y.; Gao Y.; Liu J.; Tan Y.; Liu Z.; Chhotaray C.; Jiang H.; Lu Z.; Chiwala G.; Wang S.; Makafe G.; Islam M. M.; Hameed H. M. A.; Cai X.; Wang C.; Li X.; Tan S.; Zhang T. The compound TB47 is highly bactericidal against *Mycobacterium ulcerans* in a Buruli ulcer mouse model. Nat. Commun. 2019, 10, 524 10.1038/s41467-019-08464-y. 30705268 PMC6355801

[ref73] Nateche F.; Martin A.; Baraka S.; Palomino J. C.; Khaled S.; Portaels F. Application of the resazurin microtitre assay for detection of multidrug resistance in *Mycobacterium tuberculosis* in Algiers. J. Med. Microbiol. 2006, 55, 857–860. 10.1099/jmm.0.46513-0. 16772411

[ref74] Mosaei H.; Molodtsov V.; Kepplinger B.; Harbottle J.; Moon C. W.; Jeeves R. E.; Ceccaroni L.; Shin Y.; Morton-Laing S.; Marrs E. C. L.; Wills C.; Clegg W.; Yuzenkova Y.; Perry J. D.; Bacon J.; Errington J.; Allenby N. E. E.; Hall M. J.; Murakami K. S.; Zenkin N. Mode of Action of Kanglemycin A, an Ansamycin Natural Product that Is Active against Rifampicin-Resistant *Mycobacterium tuberculosis*. Mol. Cell 2018, 72, 263–274.e5. 10.1016/j.molcel.2018.08.028. 30244835 PMC6202310

[ref75] James B. W.; Williams A.; Marsh P. D. The physiology and pathogenicity of *Mycobacterium tuberculosis* grown under controlled conditions in a defined medium. J. Appl. Microbiol. 2000, 88, 669–77. 10.1046/j.1365-2672.2000.01020.x. 10792526

[ref76] Lambert R. J. W. Susceptibility testing: inoculum size dependency of inhibition using the Colworth MIC technique. J. Appl. Microbiol. 2000, 89, 275–279. 10.1046/j.1365-2672.2000.01105.x. 10971759

[ref77] Lambert R. J.; Pearson J. Susceptibility testing: accurate and reproducible minimum inhibitory concentration (MIC) and non-inhibitory concentration (NIC) values. J. Appl. Microbiol. 2000, 88, 784–90. 10.1046/j.1365-2672.2000.01017.x. 10792538

[ref78] Luo S.; Pal D.; Shah S. J.; Kwatra D.; Paturi K. D.; Mitra A. K. Effect of HEPES Buffer on the Uptake and Transport of P-Glycoprotein Substrates and Large Neutral Amino Acids. Mol. Pharmaceutics 2010, 7, 412–420. 10.1021/mp900193e. PMC2849840 20163160

[ref79] Borgers K.; Ou J. Y.; Zheng P. X.; Tiels P.; Van Hecke A.; Plets E.; Michielsen G.; Festjens N.; Callewaert N.; Lin Y. C. Reference genome and comparative genome analysis for the WHO reference strain for *Mycobacterium bovis* BCG Danish, the present tuberculosis vaccine. BMC Genomics 2019, 20, 561 10.1186/s12864-019-5909-5. 31286858 PMC6615170

[ref80] Kapopoulou A.; Lew J. M.; Cole S. T. The MycoBrowser portal: A comprehensive and manually annotated resource for mycobacterial genomes. Tuberculosis 2011, 91, 8–13. 10.1016/j.tube.2010.09.006. 20980200

[ref81] Abrahams K. A.; Cox J. A. G.; Fütterer K.; Rullas J.; Ortega-Muro F.; Loman N. J.; Moynihan P. J.; Pérez-Herrán E.; Jiménez E.; Esquivias J.; Barros D.; Ballell L.; Alemparte C.; Besra G. S. Inhibiting mycobacterial tryptophan synthase by targeting the inter-subunit interface. Sci. Rep. 2017, 7, 9430 10.1038/s41598-017-09642-y. 28842600 PMC5573416

[ref82] Briffotaux J.; Huang W.; Wang X.; Gicquel B. MmpS5/MmpL5 as an efflux pump in *Mycobacterium* species. Tuberculosis 2017, 107, 13–19. 10.1016/j.tube.2017.08.001. 29050760

[ref83] Abrahams K. A.; Cox J. A. G.; Spivey V. L.; Loman N. J.; Pallen M. J.; Constantinidou C.; Fernández R.; Alemparte C.; Remuiñán M. J.; Barros D.; Ballell L.; Besra G. S. Identification of Novel Imidazo[1,2-a]pyridine Inhibitors Targeting *M. tuberculosis* QcrB. PLoS One 2012, 7, e52951 10.1371/journal.pone.0052951. 23300833 PMC3534098

[ref84] Gupta R. S.; Lo B.; Son J. Phylogenomics and Comparative Genomic Studies Robustly Support Division of the Genus Mycobacterium into an Emended Genus Mycobacterium and Four Novel Genera. Front. Microbiol. 2018, 9, 67 10.3389/fmicb.2018.00067. 29497402 PMC5819568

[ref85] Neagoie C. D.; Peng X.; Tortorella M. D.; Fossey J. S.; Alderwick L. J.; Feula A.; Yoshizawa A.. Preparation of antibacterial compounds: WO2020206594 A12020, 10–15.

