# Supplementary material for: Azetidines Kill Multidrug-Resistant Mycobacterium tuberculosis without Detectable Resistance by Blocking Mycolate Assembly
Source: J Med Chem. 2024 Feb 8;67(4):2529–48. doi: 10.1021/acs.jmedchem.3c01643 (PMC10895678; doi:10.1021/acs.jmedchem.3c01643)
Supplement: Supplementary file 1 — jm3c01643_si_001.pdf [file jm3c01643_si_001.pdf]

Supporting Information

Azetidines kill multidrug resistant *Mycobacterium tuberculosis* without detectable resistance by blocking mycolate assembly.

Yixin Cui [1†], Alice Lanne [2†], Xudan Peng [3], Edward Browne [4], Apoorva Bhatt [2], Nicholas J. Coltman [5], Philip Craven [1], Liam R. Cox [1], Nicholas J. Cundy [1], Katie Dale [2], Antonio Feula [1], Jon Frampton [6], Martin Fung [7], Michael Morton [8], Aaron Goff [9], Mariwan Salih [1], Xingfen Lang [3], Xingjian Li [1,3], Chris Moon [10], Jordan Pascoe [10], Vanessa Portman [4], Cara Press [2], Timothy Schulz-Utermoehl [4], Suki Lee [7], Micky D. Tortorella [3,7], Zhengchao Tu [3], Zoe E. Underwood [10], Changwei Wang [3], Akina Yoshizawa [1], Tianyu Zhang [3], Simon J Waddell [9], Joanna Bacon [10], Luke Alderwick [2,11,*], John S. Fossey [1,*,†], Cleopatra Neagoie [3,7,12,*]

[1] *School of Chemistry, University of Birmingham, Edgbaston, Birmingham, West Midlands, B15 2TT, UK*

[2] *Institute of Microbiology and Infection, School of Biosciences, University of Birmingham, Edgbaston, Birmingham, West Midlands, B15 2TT, UK*

[3] *State Key Laboratory of Respiratory Disease, China-New Zealand Joint Laboratory on Biomedicine and Health,Guangzhou Institutes of Biomedicine and Health, Chinese Academy of Science, 190 Kai Yuan Avenue, Science Park, Guangzhou, 510530, China*

[4] *Sygnature Discovery, The Discovery Building, BioCity, Pennyfoot Street, Nottingham, NG1 1GR, UK*

[5] *School of Biosciences, University of Birmingham, Edgbaston, Birmingham, West Midlands, B15 2TT, UK*

[6] *College of Medical and Dental Sciences, University of Birmingham, Edgbaston, Birmingham, West Midlands, B15 2TT, UK*

[7] *Centre for Regenerative Medicine and Health, Hong Kong Institute of Science and Innovation, Chinese Academy of Sciences, Hong Kong SAR*

[8] *ApconiX Ltd, BIOHUB at Alderly Park, Nether Alderly, Cheshire, SK10 4TG, UK*

[9] *Department of Global Health and Infection, Brighton and Sussex Medical School, University of Sussex, Falmer, BN1 9PX, UK*

[10] *TB Research Group, National Infection Service, Public Health England (UKHSA), Manor Farm Road, Porton, Salisbury SP4 0JG, UK*

[11] *Discovery Sciences, Charles River Laboratories, Chesterford Research Park, CB10 1XL, UK*

[12] *Visiting Scientist, School of Chemistry, University of Birmingham, Edgbaston, Birmingham, West Midlands, B15 2TT, UK*

CN and LA share joint last authorship together with JSF, †; YC and AL share joint first authorship.* ***Corresponding authors. Email:* *cleopatra.neagoie@crmh-cas.org.hk; luke.alderwick@crl.com*

Table of Contents

Detailed author contributions ***S2***
Further general biological details ***S3***
General chemical synthesis aspects ***S3***
Chemistry at UoB ***S3***
General synthetic chemistry procedures ***S4***
Microbiology ***S12***
Extraction of mycobacterial genomic DNA and whole genome sequencing ***S12***
Transcriptomic supporting data ***S13***
BGAz-004 significantly alters mycobacterial cell envelope composition ***S15***
BGAz-005 targets late-stage mycolic acid biosynthesis in C. glutamicum ***S18***
NMR Spectrums ***S20***
Mass Spectrometry BGAz001-005 .... ***S45***
HPLC analysis of BGAz001–BGAz005 .... ***S51***
References ***S56***

Detailed author contributions

Contributions in author order: YC applied and optimised developed methodology for the synthesis of optimised active azetidine derivatives, effectively transferred knowledge across the team *via* secondment visit and co-wrote aspects; **AL** examined anti-TB activity, determined MIC values, probed target/mechanism and elucidated aspects of the mode of action, and wrote sections of the manuscript; **XP** contributed to developing methodology applied to the synthesis of azetidine derivatives screened; **EB** conducted and analysed DMPK experiments and data; **AB** examined biological activity, contributed to interpreting findings; **NJCo** contributed and suggested aspects of biological screening around ADME, gave comment and revision of manuscript; **PC** contributed to synthetic target selection and suggested experiments; **LRC** responsible for delivery of some synthetic aspects, suggested experiments; **NJCu** applied developed methodology to synthesise active azetidine derivatives; **KD** examined biological activity and determined MIC values; **AF** uncovered enabling methodology for the synthesis azetidine derivatives and established protocols underpinning this study; **JF** contribution to the conception of the work, project management and revision of the manuscript; **MF** examined biological activity, contributed to interpreting findings; **MM** devised and conducted hERG liability in vitro screening; **AG** undertook transcriptomic analysis; **MS** applied developed methodology to synthesise active azetidine derivatives; **XLang** contributed to developing methodology applied to the synthesis of azetidine derivatives screened; **XLi** applied developed methodology to synthesise active azetidine derivatives and effectively transferred knowledge across the team *via* secondment visit; **CM** evaluated activity of azetidine derivatives against MDR-TB and determined MIC values; **JP** evaluated activity of azetidine derivatives against MDR-TB and determined MIC values; **VP** conducted and analysed DMPK experiments and data; **CP** examined biological activity and determined MIC values; **TS-U** conducted and analysed DMPK experiments and data; **SL** examined biological activity, contributed to interpreting findings; **MDT** oversight of aspects of the project and commented on progress and decision points; **ZT** biological investigation of activity of a selection of compounds using a fluorescence-based assay; **ZEU** evaluated activity of azetidine derivatives against MDR-TB and determined MIC values; **CW** biological investigation of activity of a selection of compounds using a fluorescence-based assay; **AY** Synthesised first biologically active compound identified as active in this study. Applied and optimised developed methodology for the synthesis of azetidine derivatives and effectively transferred knowledge across the team *via* secondment visit; **TZ** biological investigation of activity of a selection of compounds using a fluorescence-based assay; **SJW** led transcriptomics and co-wrote aspects of the manuscript; **JB** led on evaluation of activity of azetidine derivatives against MDR-TB; **LA** led biology aspects, suggested critical experiments, interpreted key findings, supervised aspects of the research, co-wrote aspects of the manuscript; **JSF** led chemistry aspects, suggested critical experiments,

interpreted key findings, supervised aspects of the research and co-wrote aspects of the manuscript; **CN** led the medicinal chemistry aspects, contributed to interpreting findings, supervised aspects of the research and co-wrote aspects of the manuscript;

Further general biological details

C. glutamicum (ATCC 13032) wild type and *C. glutamicum△pks13* were cultured at 30 °C, 180 rpm in brain heart infusion (BHI) media. (MIC_{99}) was determined in 96-well flat bottom, black polystyrene microtiter plates (Greiner) in a final volume of 200 µL. Compounds were two-fold serially diluted in neat DMSO and added to the microtiter plate at a final concentration of 1% DMSO. DMSO (1% in 7H9) was used as a positive control and rifampicin as a negative. The inoculum was standardised at OD_{600} 0.05 in BHI medium and added to the plate, which was then incubated without shaking at 30 °C for 24 hours. Following incubation, 42 µL of resazurin (0.02% v/v in dH_2O) was added to each well and incubated for a further two hours. Fluorescence was measured (Polarstar Omega plate reader ex 544 nm, em 590 nm) and the data normalized. The concentration of drug required to inhibit cell growth by 99% was calculated by non-linear regression (Gomperz equation for MIC determination, GraphPad Prism). For lipid analysis of *C. glutamicum* (ATCC 13032) wild type and *C. glutamicum△pks13,* cells were harvested and extracted using chloroform:methanol:water (10:10:3, v/v/v, 2 mL) for two hours at 50 °C. Following centrifugation, the organic extracts were combined with chloroform and water (1.75 mL and 0.75 mL respectively). The lower organic phase containing associated lipids was recovered, washed twice with chloroform:methanol:water (3:47:48, v/v/v, 2 mL) and dried with N_2. Samples were resuspended in chloroform:methanol (2:1, v/v, 200 µL) and OD adjusted volumes were subjected to thin-layer chromatography (TLC) analysis. Cell wall associated lipids were visualised by either heating TLC plates after treatment with molybdophosphoric acid (MPA) in ethanol (5% w/v) or alpha-naphthol in ethanol (5% w/v).

General chemical synthesis aspects

Chemistry at UoB

All commercially available solvents and reagents were purchased and used without further purification. **NMR spectroscopy:** ^{1}H NMR spectrums were acquired *via* a Bruker AVIII300 or AVIII400 at 300 or 400 MHz respectively at room temperature (21 to 28 °C). ^{13}C NMR spectrums were recorded *via* a Bruker AVIII400 or AVIII500 at 101 MHz or 126 MHz respectively at room temperature, the JMOD pulse sequence was used in some cases to assist assignment with CH_3 and CH designated and reported as (+) and C_q and CH_2 designated and reported as (-). ^{19}F NMR spectrums were recorded *via* a Bruker AVIII400 at 377 MHz at room temperature. Chemical shifts (δ) are reported in parts per million relative to residual

solvent chloroform (7.26 ppm in $CDCl_3$) or tetramethylsilane (TMS, 0.00 ppm) internal standards for ^{1}H, relative to residual solvent for ^{13}C NMR spectroscopy and are indirectly referenced to $CFCl_3$ at 0.00ppm for ^{19}F NMR spectroscopy. Coupling constants (*J*) are reported in Hertz (Hz). Multiplicities of the signals were abbreviated as singlet (s), doublet (d), triplet (t), quartet (q), septet (sept), multiplet (m) and broad (br). The NMR spectral data collected thus were processed using the MestReNova-12.0.3 software package. Mass spectra were obtained on a Waters LCT Time-of-Flight (TOF) Mass Spectrometer or a Waters GCT Premier Time-of-Flight Mass Spectrometer (TOF MS), using the ESI+ technique. Infrared spectra were recorded at room temperature nest with the ATR technique using a PerkinElmer 100FT-IR spectrometer. Flash column chromatography was performed using Teledyne ISCO CombiFlash Rf 200i, mobile and stationary phases are described in the general methods or the experimental procedures. Thin layer chromatography (TLC) was performed using aluminium-backed, F254-coated analytical TLC plates which were visualised under UV light at 254 nm or by potassium permanganate solution staining followed by heating.

General synthetic chemistry procedures

Azetidines were synthesised in four steps *via* procedures previously reported by co-authors of this report.[1,2] Conditions are described here for completeness, the synthesis of these intermediates will accompany the publication of the azetidines not carried forwards in this study, anticipated at later date.

General procedure A: Imine **S3** synthesis; Supplementary Scheme 1, step i

To solutions of aldehydes (**S1**, 1.02 equiv.) in methanol, amines (**S2**, 1.00 equiv., 0.20–0.25 M M) were added and the mixture was heated at reflux for three hours. The mixture was allowed to cool to room temperature and was concentrated *in vacuo* to the corresponding afford imines (**S3**). The loss of aldehydic (*H*C=O) proton and emergence of a signal consistent with imine (*H*C=N) in respective proton NMR spectrums confirmed formation of product and the materials were used in the next step without further purification or analysis.

General procedure B: Homoallylamine derivatives **S4** synthesis; Supplementary Scheme 1, step ii

Allyl bromide (2.5 equiv.) was added dropwise into a stirred suspension of freshly activated zinc powder (3.0 equiv.) in anhydrous tetrahydrofuran (0.20–0.25 M) at 0 ºC. After 30 minutes, imine (**S3**, 1 equiv.) was added to the suspension at room temperature. The resulting mixtures were stirred at room temperature (21–28 °C) for 14 hours, or until reactions were judged complete by TLC (silica, hexane/ethyl acetate 10:1). At which time sodium bicarbonate (saturated aqueous) was added and the resulting mixtures filtered through celite. The filtrates were extracted with ethyl acetate (3 × 30 mL), washed with water (2 × 20 mL),

dried over anhydrous magnesium sulfate and concentrated *in vacuo*. The residues thus obtained, were purified by flash chromatography (silica, ethyl acetate/hexane gradient elution 0:100–10:90), to afford the corresponding homoallyl amine (**S4**).

General procedure C: *cis*-Iodo-azetidines **S5** synthesis; Supplementary Scheme 1, steps iii & iv

To solutions of homoallyl amines (**S4**, 0.20–0.25 M, acetonitrile, 30 mL), iodine (3 equiv.) and sodium bicarbonate (5 equiv.) were added. The mixtures were stirred at 16 °C in order to suppress formation of pyrrolidine by-products [1,2]. After the reaction was judged to be complete (by TLC analysis silica, hexane/ethyl acetate 10:1), sodium thiosulfate solution (saturated aqueous) was added in order to facilitate removal of excess iodine. This mixture was extracted with ethyl acetate (3 × 30 mL), washed with water (2 × 20 mL), dried over anhydrous magnesium sulfate and concentrated *in vacuo*. The presence of iodo-azetidine derivatives (**S5**) were confirmed by proton NMR spectroscopy and used in the next step without further purification. Iodo-azetidine derivatives (**S5**) were dissolved in excess amine (pyrrolidine or propargyl amine, neat or 2.0 M methylamine in THF or DMSO) and stirred at room temperature for 48 h. Excess tetrahydrofuran or amine was removed *in vacuo* and the residues thus obtained were purified by flash column chromatography (ethyl acetate/hexane gradient elution 0:100–40:60), and if required, semi-preparative HPLC (methanol (10–15%)/ammonium hydroxide solution (0.1%))to afford *cis*-amino azetidine derivatives **BGAz-001–0016**.

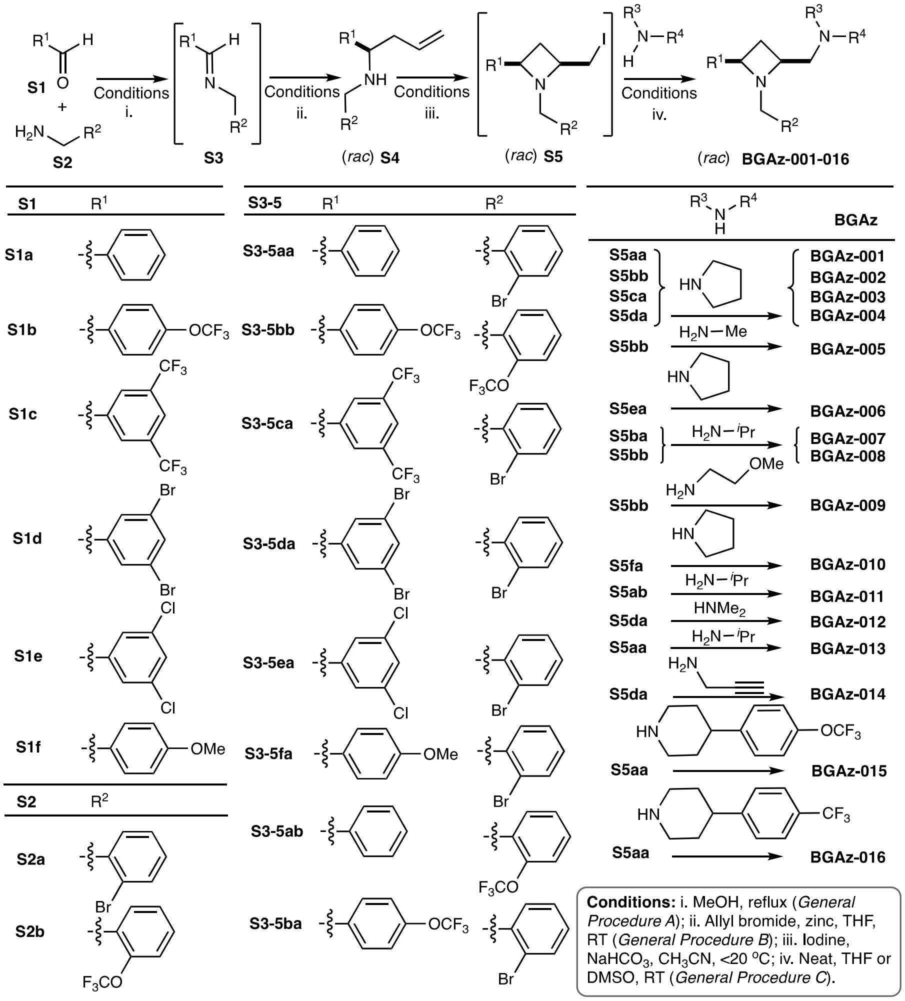

Supplementary Scheme 1. Synthesis of **BGAz001–016**.

<u>Synthesis of (*rac*)-1-(((2,4-*cis*)-1-(2-bromobenzyl)-4-phenylazetidin-2-yl)methyl)pyrrolidine, **BGAz-001**</u>

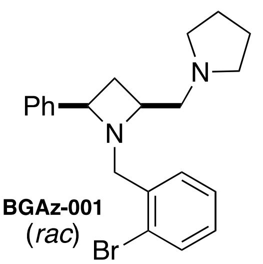

General procedures A to C were used employing **S4aa** as the starting homoallyl amine (0.6 mmol scale, **BGAz-001** was isolated as a brown oil, 40% yield). **δ_H (400 MHz, CDCl$_3$)** δ 7.48 (1H, dd, *J* 7.9 & 1.5, Ar*H*), 7.45–7.39 (3H, m, Ar*H*), 7.28 (2H, m, Ar*H*), 7.23–7.17 (1H, m, Ar*H*), 7.14 (1H, td, *J* 7.5 & 1.5, Ar*H*), 7.03 (1H, td, *J* 7.5 & 1.9, Ar*H*), 4.10 (1H, t, *J* 8.2, ArC*H*), 3.89 (1H, d, *J* 13.5, ArC*H*HN), 3.83 (1H, d, *J* 13.5, ArCH*H*N), 3.41 (1H, tdd, *J* 8.6, 6.8 & 3.6, CH_2C*H*CH_2), 2.70–2.40 (7H, m), 1.84 (1H, dt, *J* 10.3 & 8.5, ArCHC*H*H)1.77–1.68 (4H, m); **δ_C (126 MHz, CDCl$_3$)** δ 143.5, 137.9, 132.4, 131.6, 128.3, 128.0, 126.9, 126.8, 126.7, 124.3, 66.4, 62.2, 62.1, 60.7, 54.8, 35.0, 23.4; **IR** ν (cm^{-1}): 2783, 2787, 747 & 697; **TOF MS (ES+)** found 385.2 [M+H (^{79}Br)]$^+$ & 387.2 [M+H$^+$ (^{81}Br)]$^+$; **HRMS (ES+)** calcd for $C_{21}H_{26}N_2{}^{79}Br^+$: 385.1274, found 385.1277.

<u>Synthesis of (*rac*)-1-(((2,4-*cis*)-1-(2-(trifluoromethoxy)benzyl)-4-(4-(trifluoromethoxy)phenyl)azetidin-2-yl)methyl)pyrrolidine, **BGAz-002**</u>

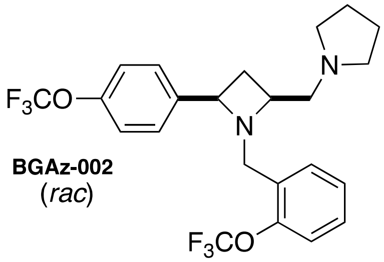

BGAz-002
(*rac*)

General procedures A to C were used employing **S4bb** as the starting homoallyl amine (0.5 mmol scale, **BGAz-002** was isolated as a brown oil, 23% yield). **δ_H (400 MHz, $CDCl_3$)** δ 7.37–7.32 (3H, m, Ar*H*), 7.19–7.10 (2H, m, Ar*H*), 7.09–7.03 (3H, m, Ar*H*), 3.98 (1H, t, *J* 8.1, Ar*H*), 3.78 (1H, d, *J* 13.3, ArC*H*HN), 3.72 (1H, d, *J* 13.3, ArCH*H*N), 3.31 (1H, tdd, *J* 8.5, 6.9 & 3.8, CH_2C*H*CH_2), 2.62–2.51 (2H, m), 2.47–2.38 (5H, m), 1.99–1.40 (5H, m); **δ_C (101 MHz, $CDCl_3$)** δ 148.1 (q, $^3J_{CF}$ 1.8), 147.6 (q, $^3J_{CF}$ 1.5), 142.2, 131.9, 130.8, 128.3, 128.0, 126.1, 120.6 (q, $^1J_{CF}$ 257.1), 120.5 (q, $^1J_{CF}$ 256.5), 120.5, 120.1, 65.4, 62.1, 62.1, 54.8, 54.7, 34.9, 23.4; **δ_F (377 MHz, $CDCl_3$)** δ -56.99 & -57.96; **IR** *ν* (cm^{-1}): 2960, 2790, 1250, 1213 & 1151; **TOF MS (ES+)** found 475.2 $[M+H]^+$ (100%) & 476.2; **HRMS (ES+)** calcd for $C_{23}H_{25}N_2O_2F_6^+$: 475.1815, found 475.1825.

<u>Synthesis of (*rac*)-1-(((2,4-*cis*)-4-(3,5-bis(trifluoromethyl)phenyl)-1-(2-bromobenzyl)azetidin-2-yl)methyl)-pyrrolidine, **BGAz-003**</u>

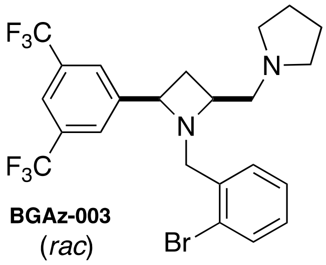

BGAz-003
(*rac*)

General procedures A to C were used employing **S4ca** as the starting homoallyl amine (0.4 mmol scale, **BGAz-003** was isolated as a brown oil, 50% yield). **δ_H (400 MHz, $CDCl_3$)** δ 7.73 (2H, s, Ar*H*), 7.59 (1H, s, Ar*H*), 7.39 (1H, dd, *J* 7.9 & 1.3, Ar*H*), 7.22 (1H, dd, *J* 7.5 & 1.7, Ar*H*), 7.04 (1H, td, *J* 7.5 & 1.3, Ar*H*), 6.95 (1H, td, *J* 7.9 & 1.7, Ar*H*), 4.17 (1H, t, *J* 8.1, ArC*H*), 3.95 (1H, d, *J* 12.4, ArC*H*HN), 3.71 (1H, d, *J* 12.4, ArCH*H*N), 3.49–3.42 (1H, m, CH_2C*H*CH_2), 2.79–2.60 (2H, m), 2.51-2.43 (5H, m), 1.96–1.62 (5H, m); **δ_C** (126 MHz, $CDCl_3$) δ 146.2, 136.7, 132.8, 131.8, 131.0 (q, $^2J_{CF}$ 32.8), 128.9, 126.9, 126.8 (q, $^3J_{CF}$ 3.6), 124.8, 123.5 (q, $^1J_{CF}$ 272.4), 120.6 (sept, $^3J_{CF}$ 3.8), 65.1, 62.0, 61.7, 60.6, 54.9, 35.0, 23.5; **δ_F** (377 MHz, $CDCl_3$) δ -62.81; **IR** *ν* (cm^{-1}): 2853, 2794, 1276, 1170 & 1130; **TOF MS** (ES+) found 521.1 $[M+H\ (^{79}Br)]^+$, 523.1 $[M+H\ (^{81}Br)]^+$ & 524.1; **HRMS** (ES+) calcd for $C_{23}H_{24}N_2F_6{}^{79}Br^+$: 521.1022, found 521.1026.

<u>Synthesis of (*rac*)-1-(((2,4-*cis*)-1-(2-bromobenzyl)-4-(3,5-dibromophenyl)azetidin-2-yl)methyl)pyrrolidine, **BGAz-004**</u>

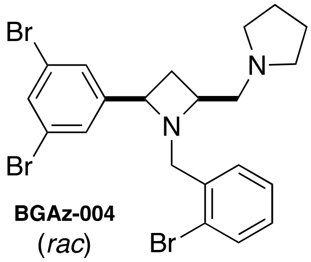

BGAz-004
(*rac*)

General procedures A to C were used employing **S4da** as the starting homoallyl amine (0.4 mmol scale, **BGAz-004** was isolated as a brown oil, 59% yield). **δ_H (400 MHz, $CDCl_3$)** δ 7.47 (1H, dd, *J* 7.8 & 1.3, Ar*H*), 7.42–7.38 (3H, m, Ar*H*), 7.28 (1H, dd, *J* 7.6 & 1.7, Ar*H*), 7.13 (1H, td, *J* 7.5 & 1.3, Ar*H*), 7.02 (1H, td, *J*

7.5 & 1.7, Ar*H*), 3.99 (1H, t, *J* 8.1, ArC*H*), 3.88 (1H, dd, *J* 12.9, ArC*H*HN), 3.71 (1H, dd, *J* 12.9, ArCH*H*N), 3.37 (1H, tdd, *J* 8.5, 7.0 & 3.5, CH_2CHCH_2), 2.68–2.55 (2H, m), 2.51–2.36 (5H, m), 1.80–1.66 (5H, m); **δ_C (101 MHz, $CDCl_3$)** δ 147.8, 137.1, 132.7, 132.2, 131.8, 128.8, 128.5, 126.9, 124.8, 122.5, 64.9, 62.1, 61.9, 60.5, 54.9, 35.0, 23.5; **IR** *ν* (cm^{-1}): 2956, 2790, 1584, 1556 & 740; **TOF MS (ES+)** found: 541.0 [$M+H^+$ (3 × ^{79}Br)], 543.0 [M+H (2 × ^{79}Br, ^{81}Br)]$^+$, 545.0 [M+H (^{79}Br, 2 × ^{81}Br)]$^+$ & 547.0 [$M+H^+$ (3 × ^{81}Br)]$^+$; **HRMS (ES+)** calcd for $C_{21}H_{24}N_2{}^{79}Br_3{}^+$: 540.9484, found 540.9489.

<u>Synthesis of (*rac*)-*N*-methyl-1-((2,4-*cis*)-1-(2-(trifluoromethoxy)benzyl)-4-(4-(trifluoromethoxy)phenyl)-azetidine-2-yl)-methanamine, **BGAz-005**</u>

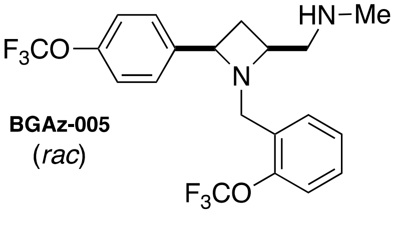

BGAz-005
(*rac*)

General procedures A to C were used employing **S4bb** as the starting homoallyl amine (0.5 mmol scale, **BGAz-005** was isolated as a brown oil, 25% yield). **δ_H ($CDCl_3$, 400 MHz)** δ 7.43–7.31 (3H, m, Ar*H*), 7.24–7.04 (5H, m, Ar*H*), 3.99 (1H, t, *J* 8.1, ArC*H*), 3.75 (1H, d, *J* 13.2, ArC*H*HN), 3.71 (1H, d, *J* 13.2, ArCH*H*N), 3.34 (1H, dddd, *J* 8.7, 7.1, 5.3 & 3.7, CH_2CHCH_2), 2.54 (1H, dd, *J* 12.2 & 3.8, NHC*H*HCH), 2.48–2.36 (2H, m), 2.32 (3H, s, Me), 2.10 (1H, s), 1.95 (1H, dt, *J* 10.2 & 8.6, ArCHCH*H*); **δ_C (101 MHz, $CDCl_3$)** δ 148.2 (q, $^3J_{CF}$ 1.8), 147.7 (q, $^3J_{CF}$ 1.5), 141.9, 131.7, 130.6, 128.6, 128.0, 126.3, 120.6 (q, $^1J_{CF}$ 257.3), 120.6, 120.5 (q, $^1J_{CF}$ 256.6), 120.1 65.0, 62.0, 55.6, 54.8, 36.6, 31.3; **δ_F (377 MHz, $CDCl_3$)** δ -57.03 & -57.96; **TOF MS** (ES+) found 435.2 [M+H]$^+$; **HRMS** (ES+) calcd for $C_{20}H_{21}N_2F_6O_2{}^+$: 435.1502, found 435.1505; **IR** *ν* (cm^{-1}): 2851, 1249, 1212, 1150 & 758.

<u>Synthesis of (*rac*)-1-(((2,4-*cis*)-1-(2-bromobenzyl)-4-(3,5-dichlorophenyl)azetidin-2-yl)methyl)pyrrolidine, **BGAz-006**</u>

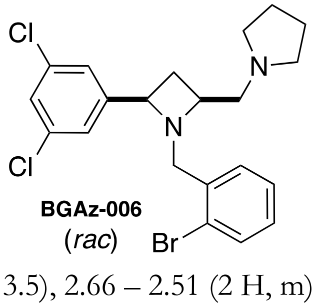

BGAz-006
(*rac*)

General procedures A to C were used employing **S4ea** as the starting homoallyl amine (4 mmol scale, **BGAz-006** was isolated as a yellow oil, 27%). **δ_H (400 MHz, $CDCl_3$)** 7.46 (1 H, dd, *J* 8.0, 1.4), 7.29 (1 H, dd, *J* 7.6, 1.9), 7.21 (2 H, d, *J* 1.9), 7.18 – 7.12 (1 H, m), 7.11 (1 H, t, *J* 2.0), 7.02 (1 H, td, *J* 7.6, 1.9), 4.01 (1 H, t, *J* 8.1), 3.88 (1 H, d, *J* 13.0), 3.72 (1 H, d, *J* 13.0), 3.37 (1 H, tdd, *J* 8.6, 6.9 & 3.5), 2.66 – 2.51 (2 H, m), 2.50 – 2.23 (5 H, m), 1.85 – 1.68 (5 H, m); **δ_C (126 MHz, $CDCl_3$)** 147.26, 137.15, 134.42, 132.68, 131.80, 128.76, 126.91, 126.81, 125.10, 124.72, 65.07, 62.02, 61.95, 60.49, 54.82, 34.82 & 23.45. **MS (ESI)** m/z 453 [M+H]$^+$.

Synthesis of *(rac)*-*N*-(((2,4-*cis*)-1-(2-bromobenzyl)-4-(4-(trifluoromethoxy)phenyl)azetidin-2-yl)methyl)propan-2-amine, **BGAz-007**

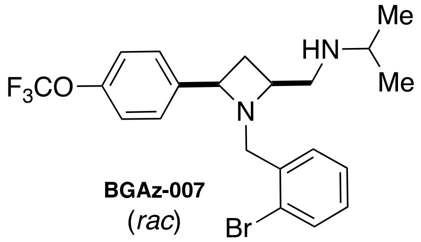

BGAz-007
(*rac*)

General procedures A to C were used employing **S4ba** as the starting homoallyl amine (1.2 mmol scale, **BGAz-007** was isolated as a pale brown-yellow oil, 21%). **δ_H (500 MHz, $CDCl_3$)** 7.45 (1 H, d, *J* 7.7), 7.39 (2 H, d, *J* 8.5), 7.29 (1 H, dd, *J* 7.7, 1.7), 7.15 – 7.03 (3 H, m), 7.00 (1 H, td, *J* 7.6, 2.0), 4.03 (1 H, t, *J* 8.1), 3.83 (1 H, d, *J* 12.9), 3.74 (1 H, d, *J* 12.9), 3.39 (1 H, tt, *J* 8.7, 4.5), 2.59 (1 H, dd, *J* 11.8, 4.1), 2.57 – 2.50 (1 H, m), 2.44 – 2.40 (2 H, m), 1.93 (1 H, q, *J* 8.7), 1.32 (1 H, brs), 0.99 (6 H, dd, *J* 31,3, 6.2), 0.93 (6 H, dd, *J* 31,3, 6.2); **δ_C (126 MHz, $CDCl_3$)** 148.12, 142.16, 137.55, 132.75, 131.59, 128.70, 128.05, 126.96, 124.58, 120.52 (q, J_{CF} 256.6), 120.48, 65.21, 62.64, 60.93, 51.68, 49.07, 31.46, 23.11 & 22.59; **δ_F (471 MHz, $CDCl_3$)** -57.87. **MS (ESI)** m/z 457 $[M+H]^+$.

Synthesis of *(rac)*-*N*-(((2,4-*cis*)-1-(2-(trifluoromethoxy)benzyl)-4-(4-(trifluoromethoxy)phenyl)azetidin-2-yl)methyl)propan-2-amine, **BGAz-008**

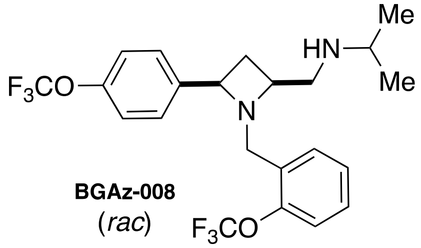

BGAz-008
(*rac*)

General procedures A to C were used employing **S4bb** as the starting homoallyl amine (1.1 mmol scale, **BGAz-008** was isolated as a pale brown oil, 27%). **δ_H (400 MHz, $CDCl_3$)** 7.46 – 7.34 (3 H, m), 7.25 – 7.02 (5 H, m), 4.00 (1 H, t, *J* 8.1), 3.77 – 3.70 (2 H, m), 3.32 (1 H, ddt, *J* 12.8, 8.8, 4.7), 2.64 – 2.50 (2 H, m), 2.49 – 2.33 (2 H, m), 1.89 (1 H, q, *J* 8.8), 1.27 (1 H, brs), 0.97 (3 H, d, 6.2), 0.92 (3 H, d, 6.2); **δ_C (126 MHz, $CDCl_3$)** 148.19, 147.73, 142.06, 131.72, 130.93, 128.50, 127.99, 126.29, 120.58, 120.6 (q, J_{CF} 257.3), 120.5 (q, J_{CF} 256.5), 120.16, 64.96, 62.79, 54.99, 51.78, 48.95, 31.53, 22.96, 22.54; **δ_F (471 MHz, $CDCl_3$)** -56.98, -57.94. **MS (ESI)** m/z 463 $[M+H]^+$.

Synthesis of *(rac)*-2-methoxy-*N*-(((2,4-*cis*)-1-(2-(trifluoromethoxy)benzyl)-4-(4-(trifluoromethoxy)phenyl)azetidin-2-yl)methyl)ethan-1-amine, **BGAz-009**

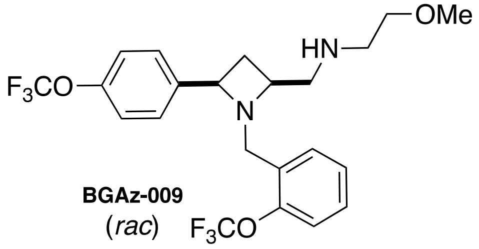

BGAz-009
(*rac*)

General procedures A to C were used employing **S4bb** as the starting homoallyl amine (2.2 mmol scale, **BGAz-009** was isolated as a yellow oil, 50%). **δ_H (500 MHz, $CDCl_3$)** 7.46 – 7.31 (3 H, m), 7.21 – 6.99 (5 H, m), 3.99 (1 H, t, *J* 8.3), 3.87 – 3.68 (2 H, m), 3.42 (2 H, t, *J* 5.4), 3.34 (3 H, s), 3.33 – 3.26 (1 H, m), 2.74 – 2.62 (2 H, m), 2.60 – 2.51 (2 H, m), 2.52 (1 H, dd, *J* 12.5, 6.3), 2.17 (2 H, dq, *J* 283, 9.1, 8.4); **δ_C (126 MHz, $CDCl_3$)** 148.30, 147.66, 141.37, 131.80, 130.36, 128.74, 127.98, 126.53, 120.72, 120.55 (q, J_{CF} 257.1), 120.49 (q, J_{CF}

256.9), 120.29, 70.64, 64.88, 61.22, 58.86, 54.39, 50.63, 49.25 & 31.10; **δ_F (471 MHz, $CDCl_3$)** -57.01, -57.91. **MS (ESI)** m/z 479[M+H]$^+$.

Synthesis of (*rac*)-1-(((2,4-*cis*)-1-(2-bromobenzyl)-4-(4-methoxyphenyl)azetidin-2-yl)methyl)pyrrolidine, **BGAz-010**

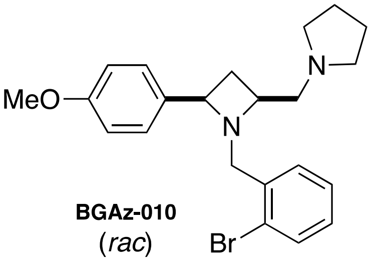

BGAz-010
(*rac*)

General procedures A to C were used employing **S4fa** as the starting homoallyl amine (2.2 mmol scale, **BGAz-010** was isolated as a yellow oil, 14%). **δ_H (400 MHz, $CDCl_3$)** 7.45 (1 H, d, *J* 7.9), 7.36 (1 H, d, *J* 7.5), 7.32 (2 H, d, *J* 8.6), 7.12 (1 H, t, *J* 7.4), 7.00 (1 H, t, *J* 8.2), 6.80 (2 H, d, *J* 8.6), 4.00 (1 H, t, *J* 8.1), 3.92 – 3.61 (5 H, m), 3.43 – 3.27 (1 H, m), 2.68 – 2.50 (2 H, m), 2.44 – 2.41 (5 H, m), 2.10 – 1.67 (5 H, m); **δ_C (126 MHz, $CDCl_3$)** 158.71, 137.96, 135.62, 132.45, 131.69, 128.36, 127.97, 126.84, 124.35, 113.41, 65.98, 61.92, 61.77, 60.59, 55.24, 54.73, 35.25 & 23.37. **MS (ESI)** m/z 415 [M+H]$^+$.

Synthesis of (*rac*)-*N*-(((2,4-*cis*)-4-phenyl-1-(2-(trifluoromethoxy)benzyl)azetidin-2-yl)methyl)propan-2-amine, **BGAz-011**

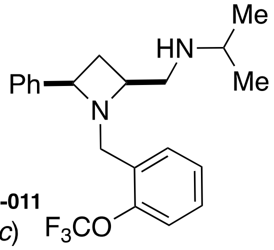

BGAz-011
(*rac*)

General procedures A to C were used employing **S4ab** as the starting homoallyl amine (2.2 mmol scale, **BGAz-011** was isolated as a pale brown oil, 38%). **δ_H (500 MHz, $CDCl_3$)** 7.49 – 7.37 (3 H, m), 7.32 – 7.11 (6 H, m), 4.01 (1 H, t, *J* 8.1), 3.77 (1 H, d, *J* 13.6), 3.73 (1 H, d, *J* 13.4), 3.42 – 3.11 (1 H, m), 2.65 – 2.47 (2 H, m), 2.47 – 2.34 (2 H, m), 1.92 (1 H, q, *J* 9.2), 1.07 – 1.00 (1 H, m), 0.95 (3 H, d, *J* 6.3), 0.90 (3 H, d, *J* 6.1) ;**δ_C (126 MHz, $CDCl_3$)** 147.66, 143.29, 131.72, 131.43, 128.31, 128.09, 127.02, 126.73, 126.31, 120.63 (q, J_{CF} 257.4), 120.20, 65.71, 62.98, 54.90, 51.97, 48.93, 31.53, 22.99 & 22.57; **δ_F (471 MHz, $CDCl_3$)** -56.94. **MS (ESI)** m/z 379 [M+H]$^+$.

Synthesis of (*rac*)-1-((2,4-*cis*)-1-(2-bromobenzyl)-4-(3,5-dibromophenyl)azetidin-2-yl)-*N*,*N*-dimethylmethanamine, **BGAz-012**

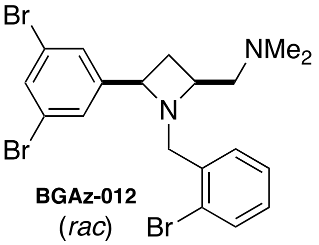

BGAz-012
(*rac*)

General procedures A to C were used employing **S4da** as the starting homoallyl amine (2.2 mmol scale, **BGAz-012**, 23%). **δ_H (500 MHz, $CDCl_3$)** 7.48 (1 H, dd, *J* 7.9, 1.3), 7.41 (1 H, d, *J* 1.9), 7.40 (2 H, d, *J* 1.7), 7.26 (1 H, d, *J* 1.6), 7.14 (1 H, td, *J* 7.5, 1.3), 7.04 (1 H, td, *J* 7.6, 1.7), 4.00 (1 H, t, *J* 8.1), 3.86 (1 H, d, *J* 12.8), 3.72 (1 H, d, *J* 12.8), 3.53 – 3.30 (1H, m), 2.68 – 2.54 (1 H, m), 2.32 (2 H, d, *J* 6.1), 2.17 (6 H, s), 1.72 – 1.62 (1 H, m); **δ_C (126 MHz, $CDCl_3$)** 147.54, 136.88, 132.79, 132.31, 131.88, 128.95, 128.43, 127.01, 124.82, 122.51, 65.07, 64.95, 60.81, 60.38, 46.13 & 34.99. **MS (ESI)** m/z 517 & 519.3 [M+H]$^+$.

Synthesis of (*rac*)-*N*-(((2,4-*cis*)-1-(2-bromobenzyl)-4-phenylazetidin-2-yl)methyl)propan-2-amine, **BGAz-013**

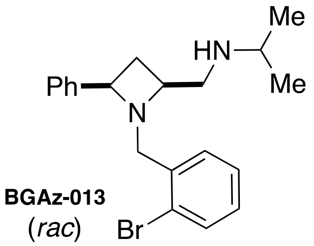

General procedures A to C were used employing **S4aa** as the starting homoallyl amine (2.2 mmol scale, **BGAz-013** was isolated as a pale brown-yellow oil, 33%). **δ_H (500 MHz, CDCl$_3$)** 7.47 (1 H, d, *J* 7.9), 7.41 (1 H, d, *J* 7.7), 7.37 (1 H, d, *J* 7.6), 7.31 – 7.23 (2 H, m), 7.18 (1 H, t, *J* 7.3), 7.14 (1 H, t, *J* 7.4), 7.02 (1 H, t, *J* 7.6), 4.05 (1 H, t, *J* 8.1), 3.82 (1 H, d, *J* 13.3), 3.78 (1 H, d, *J* 13.1), 3.39 – 3.36 (1 H, m), 2.57 (1 H, dd, *J* 11.8, 3.7), 2.53 – 2.47 (1 H, m), 2.47 – 2.35 (2 H, m), 1.95 (1 H, q, *J* 9.1), 1. 30 (1 H, brs), 0.98 (3 H, d, *J* 6.2), 0.91 (3 H, d, *J* 6.2); **δ_C (126 MHz, CDCl$_3$)** 143.35, 138.02, 132.66, 131.55, 128.54, 128.01, 126.95, 126.94, 126.81, 124.48, 65.96, 62.82, 60.94, 51.89, 49.04, 31.45, 23.10 & 22.58. **MS (ES+)** m/z 375 $[M+H]^+$.

Synthesis of (*rac*)-*N*-(((2,4-*cis*)-1-(2-bromobenzyl)-4-(3,5-dibromophenyl)azetidin-2-yl)methyl)prop-2-yn-1-amine, **BGAz-014**

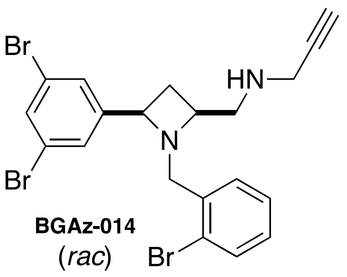

General procedures A to C were used employing **S4da** as the starting homoallyl amine (2.2 mmol scale, **BGAz-014**, 28%). **δ_H (400 MHz, CDCl$_3$)** 7.50 (1 H, dd, *J* 8.0, 1.3), 7.41 (1 H, t, *J* 1.9), 7.38 (2 H, d, *J* 1.9), 7.24 (1 H, dd, *J* 7.6, 1.8), 7.14 (1 H, td, *J* 7.4, 1.3), 7.04 (1 H, td, *J* 7.6, 1.8), 3.99 (1 H, t, *J* 8.1), 3.87 (1 H, d, *J* 12.5), 3.70 (1 H, d, *J* 12.6), 3.54 – 3.25 (3 H, m), 2.68 (1 H, dd, *J* 12.0, 4.9), 2.58 (1 H, dd, *J* 12.1, 3.7), 2.42 (1 H, dt, *J* 10.2, 7.5), 2.32 – 2.10 (1 H, m), 1.96 (1 H, dt, *J* 10.2, 8.5), 1.50 (1H, brs); **δ_C (126 MHz, CDCl$_3$)** 147.27, 136.74, 132.95, 132.32, 131.69, 129.08, 128.41, 127.16, 124.80, 122.49, 81.61, 71.72, 64.56, 61.54, 60.62, 51.73, 38.36 & 30.94. **MS (ES+)** m/z 529 $[M+H]^+$.

Synthesis of (*rac*)-1-(((2,4-*cis*)-1-(2-bromobenzyl)-4-phenylazetidin-2-yl)methyl)-4-(4-(trifluoromethoxy)-phenyl)piperidine, **BGAz-015**

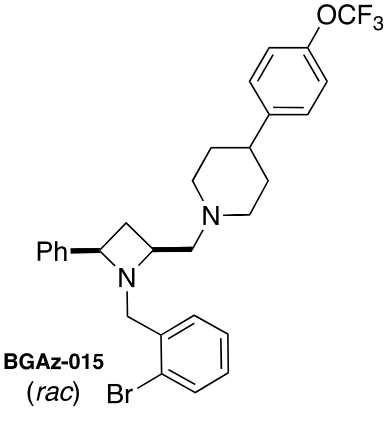

General procedures A to C were used employing **S4aa** as the starting homoallyl amine (3.2 mmol scale, **BGAz-015**, was isolated as a pale yellow oil, 13%). **δ_H (400 MHz, CDCl$_3$)** 7.49 – 7.42 (1 H, m), 7.42 – 7.35 (3 H, m), 7.29 – 7.16 (6 H, m), 7.14 – 7.09 (2 H, m), 7.00 (1 H, m), 4.08 (1 H, t, *J* 8.1), 3.91 (1 H, d, *J* 13.8), 3.79 (1 H, d, *J* 13.8), 3.42 (1 H, m), 2.95 (1 H, d, *J* 10.1), 2.88 (1 H, d, *J* 11.3), 2.59 (1 H, dt, *J* 10.2, 7.2), 2.43 (3 H, m), 2.02 (2 H, dtd, *J* 17.3, 11.5, 3.1), 1.88 – 1.61 (5 H, m); **δ_C (126 MHz, CDCl$_3$)** 147.47, 145.19, 143.52, 138.10, 132.42, 131.71, 128.30, 128.03,

127.98, 126.93, 126.74, 124.34, 120.85, 120.54 (q, J_{CF} 256.5), 66.61, 64.65, 61.43, 60.87, 55.21, 54.47, 41.94, 35.09, 33.44 & 33.39; **δ_F (471 MHz, $CDCl_3$)** -57.86. **MS (ES+)** m/z 562 $[M+H]^+$.

Synthesis of (*rac*)-1-(((2,4-*cis*)-1-(2-bromobenzyl)-4-phenylazetidin-2-yl)methyl)-4-(4-(trifluoromethyl)phenyl)piperidine, **BGAz-016**

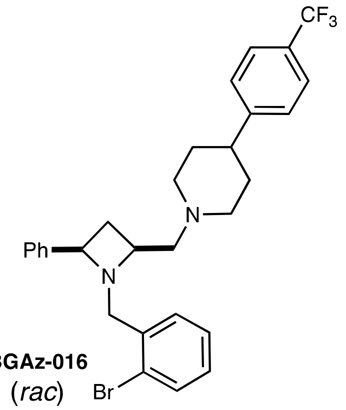

BGAz-016
(*rac*)

General procedures A to C were used employing using DMSO as solvent and **S4aa** as the starting homoallyl amine (3.2 mmol scale, **BGAz-016** was isolated as a yellow oil, 9%). **δ_H (400 MHz, $CDCl_3$)** 7.53 (2 H, d, *J* 8.3), 7.50 – 7.43 (1 H, m), 7.43 – 7.38 (3 H, m), 7.32 – 7.22 (4 H, m), 7.21 – 7.15 (1 H, m), 7.11 (1 H, td, *J* 7.5, 1.3), 7.00 (1 H, td, *J* 7.6, 1.9), 4.08 (1 H, t, *J* 8.1), 3.91 (1 H, d, *J* 13.6), 3.79 (1 H, d, *J* 13.6), 3.47 – 3.37 (1 H, m), 3.12 – 2.93 (1 H, m), 2.89 (1 H, d, *J* 11.2), 2.59 (1 H, dt, *J* 10.2, 7.2), 2.54 – 2.37 (2 H, m), 2.03 (2 H, m), 1.81 (2 H, m), 1.77 – 1.61 (4 H, m); **δ_C (126 MHz, $CDCl_3$)** 150.52, 143.50, 138.10, 132.41, 131.71 & 131.58 (q, J_{CF} 249.52) 128.30, 127.98, 127.17, 126.93, 126.76, 126.73, 125.30, 125.27, 124.33, 66.57, 64.61, 61.41, 60.86, 55.13, 54.38, 42.44, 35.02, 33.19 & 33.14; **δ_F (471 MHz, $CDCl_3$)** -62.26; **MS (ES+)** m/z 545 $[M+H]^+$.

Microbiology

Extraction of mycobacterial genomic DNA and whole genome sequencing

M. smegmatis and *M. bovis* BCG were plated onto agar containing 5, 10 and 20 × MIC concentrations of **BGAz-002–BGAz-005** in attempts to generate resistant mutants. Treatment of *M. bovis* BCG with 10 × MIC_{99} of **BGAz-002** resulted in the formation of three resistant colonies. Whole-genome sequencing analysis of these three colonies detected six putative non-synonymous single nucleotide polymorphisms (SNPs) compared to the *M. bovis* BCG reference sequence (Supplementary Table 1). Of these six, three were found across all the mutants. However, these three SNPs (in genes *infB, lipN* and BCG_3519) align with SNPs previously reported in the laboratory parental strain of BCG,[3] and are background mutations which have arisen during laboratory storage, and thus cannot be conferring the resistant phenotype. The stochastic nature of the other three SNPs identified means that the genes in which they occur can be assumed not to be the target of the BGAz compounds and are unlikely to confer specific resistance. Indeed, two of these SNPs occur in BCG_0727, a transcriptional regulator of the MmpL5 efflux pump involved in non-specific resistance [4]. Thus, none of the SNPs identified in the three resistant colonies provide insights into the specific gene target of the BGAz compounds tested.

Supplementary Table 1. Non-synonymous miscoding single nucleotide polymorphisms of apparent **BGAz-004** resistant mutants of BCG.

M. bovis **(BCG) chromosome**	**Codon Change**	**Amino Acid Change**	**Gene**	**Mutant 1**	**Mutant 2**	**Mutant 3**
810466	aTg/aGg	M23R	BCG_0727	-	-	G
810652	gTc/gGc	V85G	BCG_0727	-	G	-
3100829	gCc/gTc	A344V	infB	A	A	A
3278815	Ctg/Gtg	L351V	lipN	C	C	C
3856853	gGc/gAc	G294D	BCG_3519	A	A	A
3907860	Aac/Gac	N599D	PE_PGRS53	-	G	-

Transcriptomic supporting data

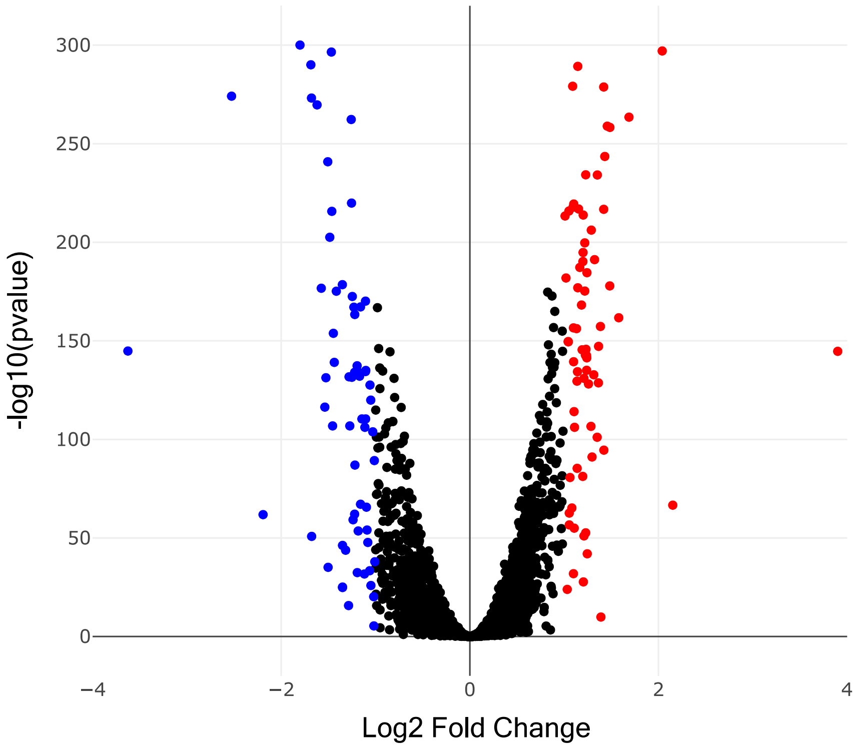

Supplementary Figure 1. Volcano plot of the *M. bovis* BCG transcriptional response to **BGAz-004** exposure, highlighting genes significantly differentially expressed relative to carrier control. Red colouring marking significantly induced genes, blue repressed genes.

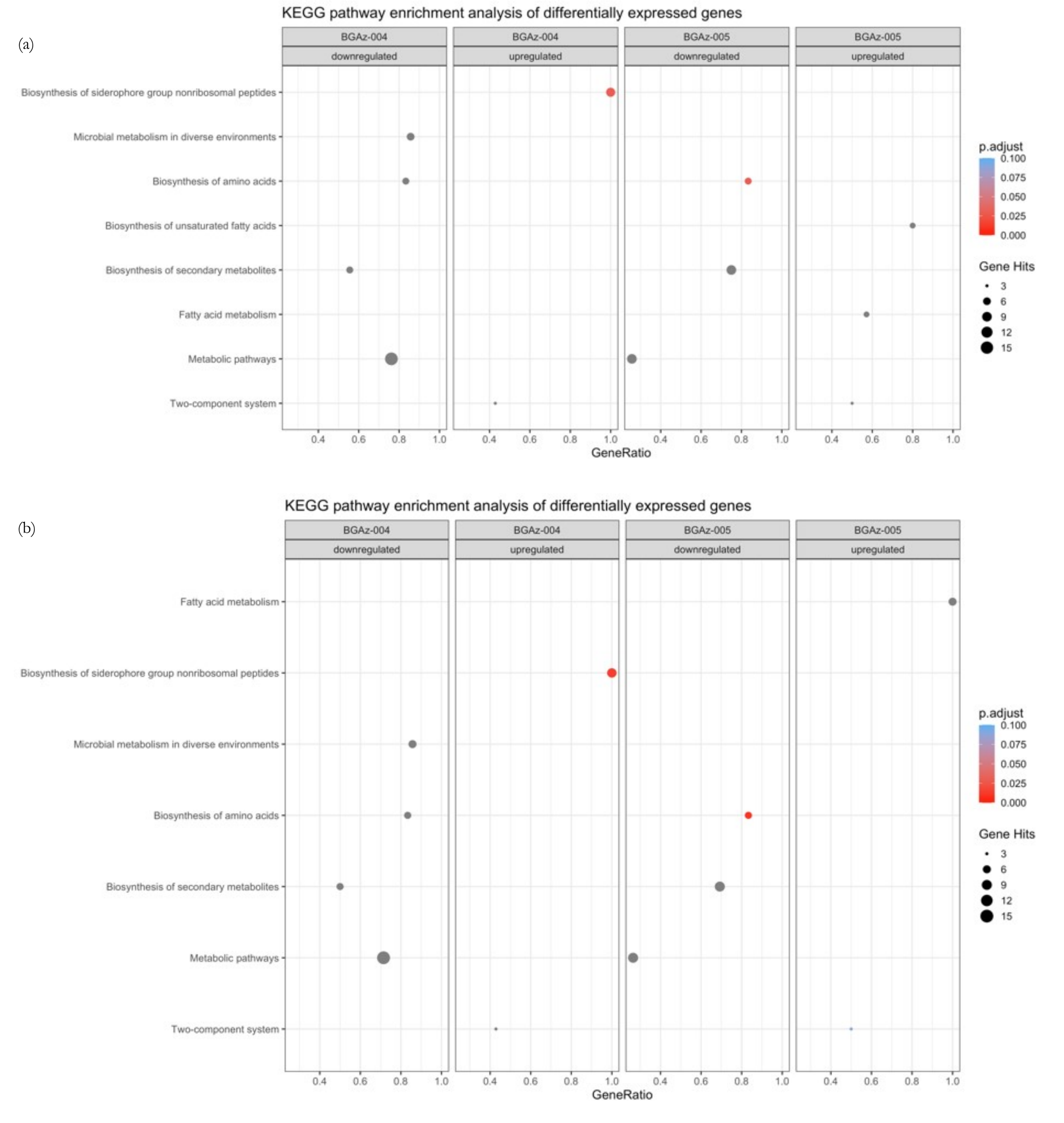

Supplementary Figure 2. KEGG Pathway Enrichment Analysis first selects genes with a differential expression of at least log2foldchange of 1. These genes are then clustered into functional groups (using information provided in the KEGG database), up to a P-value of 1. For this analysis, only genes with a 2-fold cutoff of 2 and an adjusted P-value of at least 0.05 were used. All other genes were disregarded. Analysis was completed with both (a) BCG identifiers using Pasteur genome and then (b) Rv identifiers using H37Rv genome.

BGAz-004 ***significantly alters mycobacterial cell envelope composition***

To investigate the mechanism by which mycobacterial cell envelope lipid composition is affected by **BGAz-004**, actively growing cultures of BCG were exposed to increasing concentrations of compound followed by metabolic labelling using [^{14}C]-acetic acid. Autoradiographs of cell envelope lipids separated by thin layer chromatography (TLC) reveals that treatment of BCG with **BGAz-004** at 0.5 × MIC causes a significant reduction in trehalose monomycolate (TMM) and trehalose dimycolate (TDM) and a complete loss of TMM and TDM at concentrations beyond the MIC (Supplementary Figure 3A). The formation of cytoplasmic membrane phospholipids (PIMs and CL) remain unaffected (Supplementary Figure 3A). The analysis of lipids loaded and separated by TLCs that had been normalised for total lipids extracted, revealed an altered lipid profile highlighting the accumulation of an unidentified lipid species that resolves to a relatively high Rf (Lipid species X, (Supplementary Figure 5B). The analysis of mycolic acid methyl esters (MAMES) reveals that both alpha and keto mycolates bound to the cell wall arabinogalactan (AG) are gradually depleted as BCG is exposed to increasing concentrations of **BGAz-004** during active cell culture (Supplementary Figure 3C). Quantification of the relative abundance of each lipid species highlights the significant depletion of mycolates (either conjugated to trehalose in the form of TMM/TDM or AG) when **BGAz-004** is used at a half MIC, whilst other lipids including PI and PIMS remain largely unaffected (Supplementary Figure 3D).

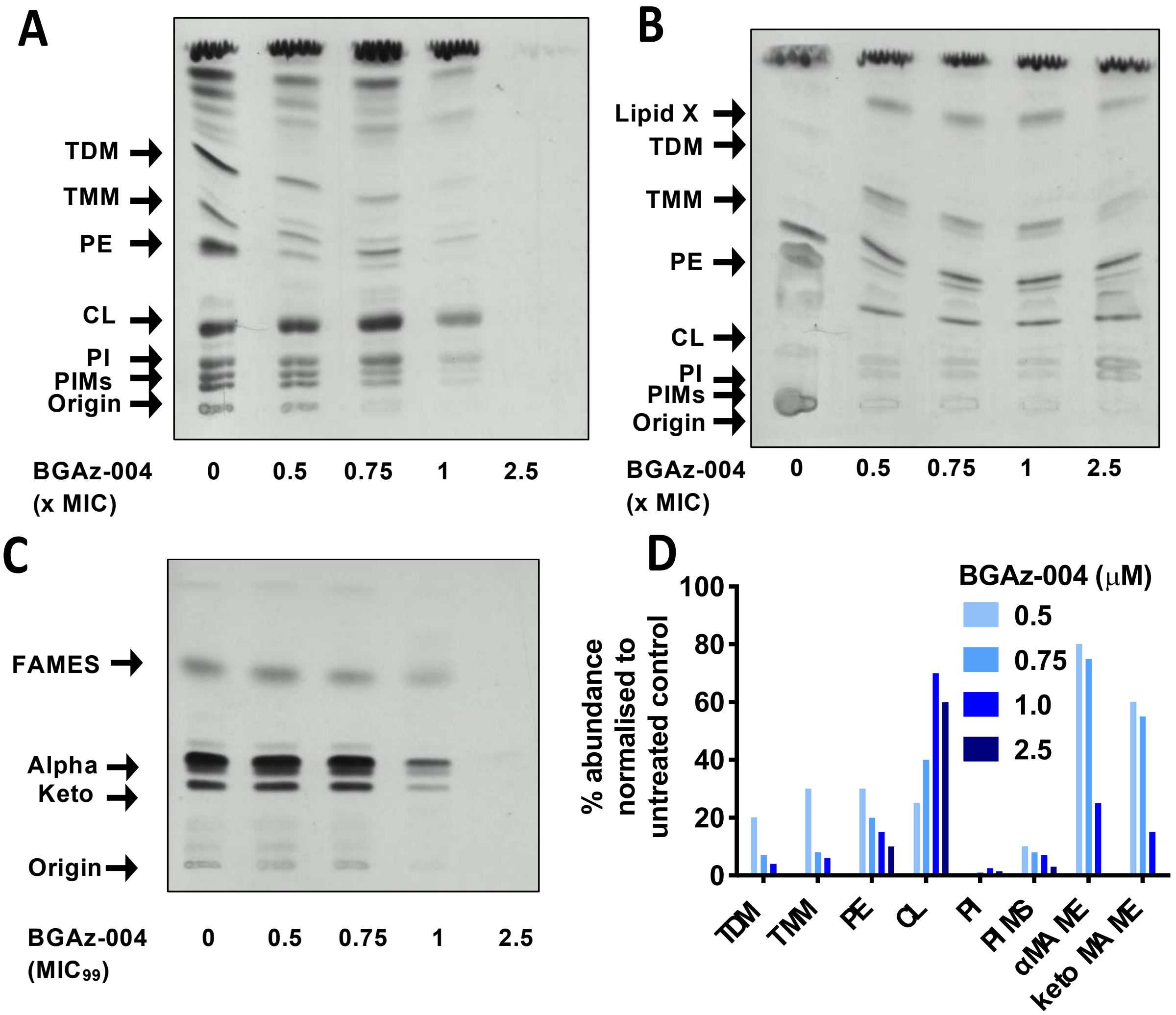

Supplementary Figure 3. BCG cell envelope lipid analysis upon exposure to **BGAz-004**. BCG were cultured in 7H9 broth and exposed to increasing concentrations of **BGAz-004**. Lipids were selectively labelled with [^{14}C]-acetic acid for 12 hours and cell envelope lipids were selectively removed by solvent extraction, separated by TLC (chloroform/methanol/water, 80:20:2, v/v/v), and visualised by autoradiography. A: equal volumes of lipids loaded adjusted for BCG growth. B: equal counts of lipids (25,000 cpm) loaded. C: Mycolic acid methyl ester (MAME) analysis of cell wall bound mycolates released by 5% TBAH and separated by TLC (petroleum ether/acetone, 95:5, v/v). D: quantification of BCG lipids from panels A-C by densitometry.

To investigate the effect of **BGAz-004** on mycobacterial cell envelope composition and to further identify the composition of lipid-X that appears in mycobacteria, actively growing cultures of *M. smegmatis* were exposed to **BGAz-004** concentrations, which resulted in a titratable-dependent reduction in the formation of TMM and TDM which can be observed by staining with MPA and α-napthol (Supplementary Figure 4A) consistent with [^{14}C]-labelling experiments performed when **BGAz-005** was exposed to BCG (Main Article). *M. smegmatis* exposed to the highest concentration of **BGAz-004**, resulted in a significant increase in the relative abundance of free mycolic acid (MA) within the cell envelope (Supplementary Figure 4C and D). This effect can also be observed when *M. smegmatis* is exposed to the known FbpC inhibitor ebselen (Supplementary Figure 5).

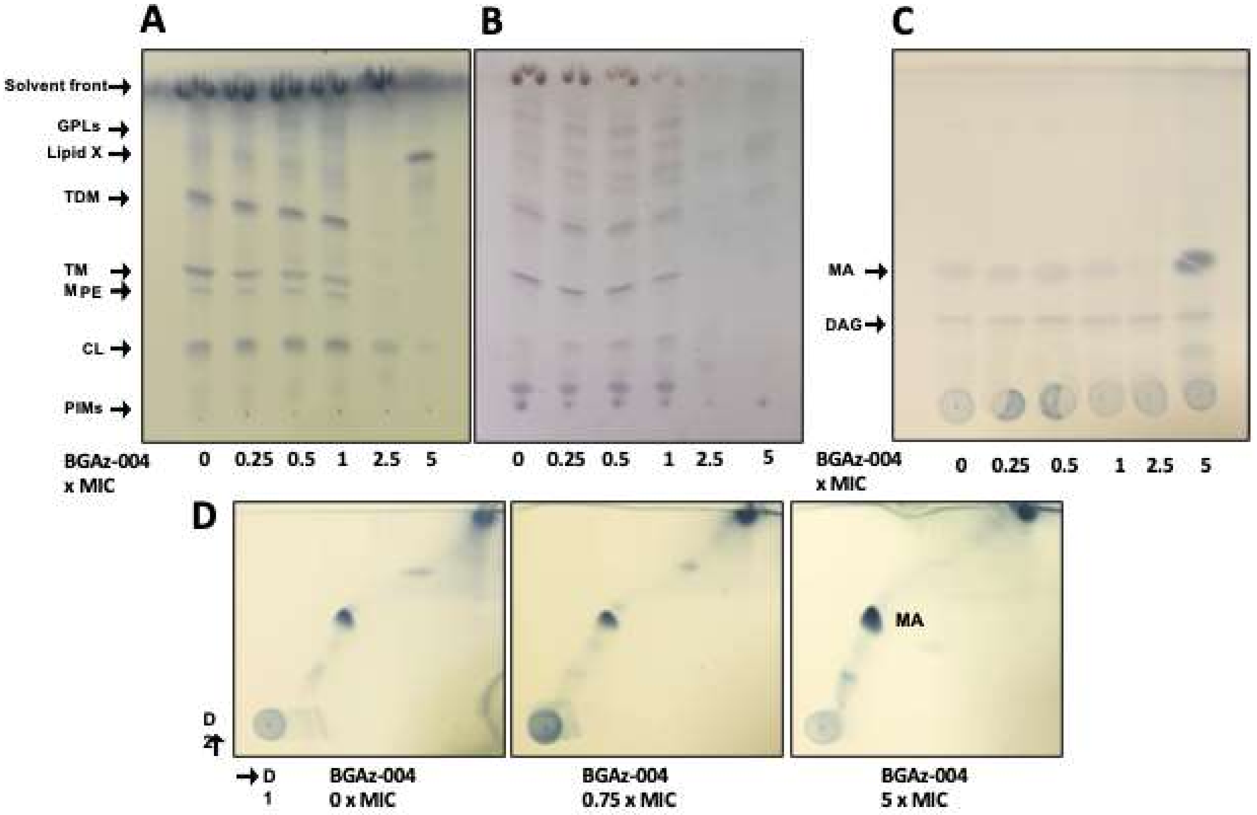

Supplementary Figure 4. *M. smegmatis* cell envelope lipid analysis upon exposure to **BGAz-004**. *M. smegmatis* were cultured in 7H9 broth, exposed to increasing concentrations of **BGAz-004** for 6 h and the cell envelope lipids selectively removed by solvent extraction. Equal volumes of lipid adjusted by bacterial growth were separated by TLC (chloroform/methanol/water, 80:20:2, v/v/v), and stained with MPA (A) or alpha-naphthol (B). Equal volumes of lipid adjusted by bacterial growth were separated by TLC (hexane/diethyl ether/acetic acid), 70:30:1, v/v/v and stained with MPA (C). Equal volumes of lipid adjusted by bacterial growth were separated by 2D-TLC (direction 1 chloroform/methanol 96:4, v/v, direction 2 toluene/acetone 80:20, v/v) and stained with MPA (D).

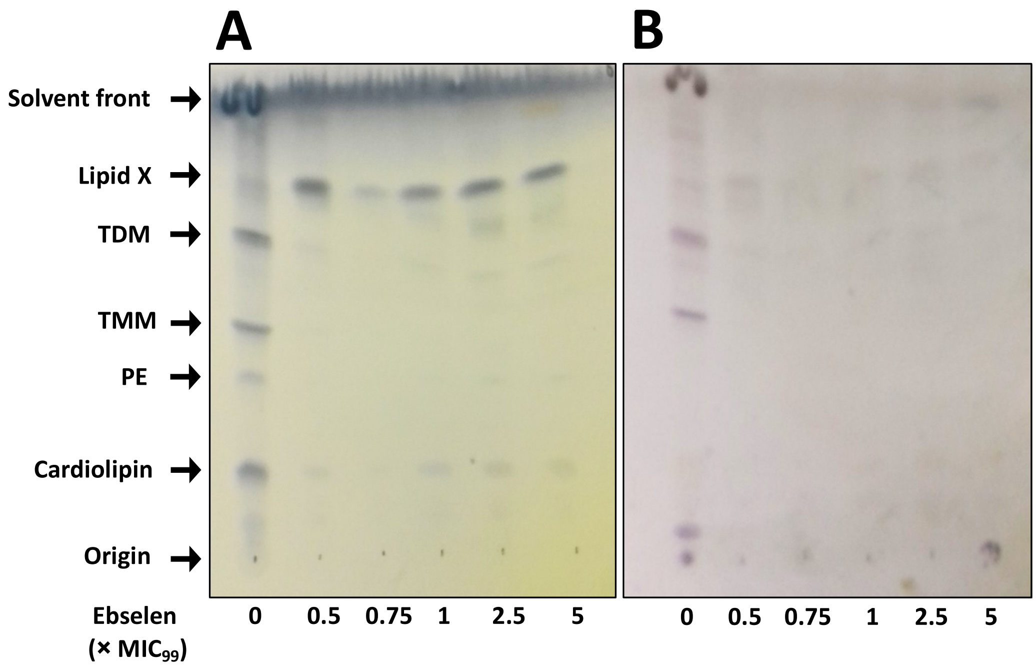

Supplementary Figure 5. M. smegmatis cell envelope lipid analysis upon exposure to Ebselen**.** *M. smegmatis* were cultured in 7H9 broth, exposed to increasing concentrations of Ebselen for 6 h and the cell envelope lipids selectively removed by solvent extraction. Equal volumes of lipid adjusted by bacterial growth were separated by TLC (chloroform/methanol/water, 80:20:2, v/v/v), and stained with MPA (A) or alpha-naphthol (B).

BGAz-005 ***targets late-stage mycolic acid biosynthesis in*** **C. glutamicum**

BGAz-005 inhibits corynemycolic acid biosynthesis in *C. glutamicum* similarly to mycobacteria (Supplementary Figure 6).

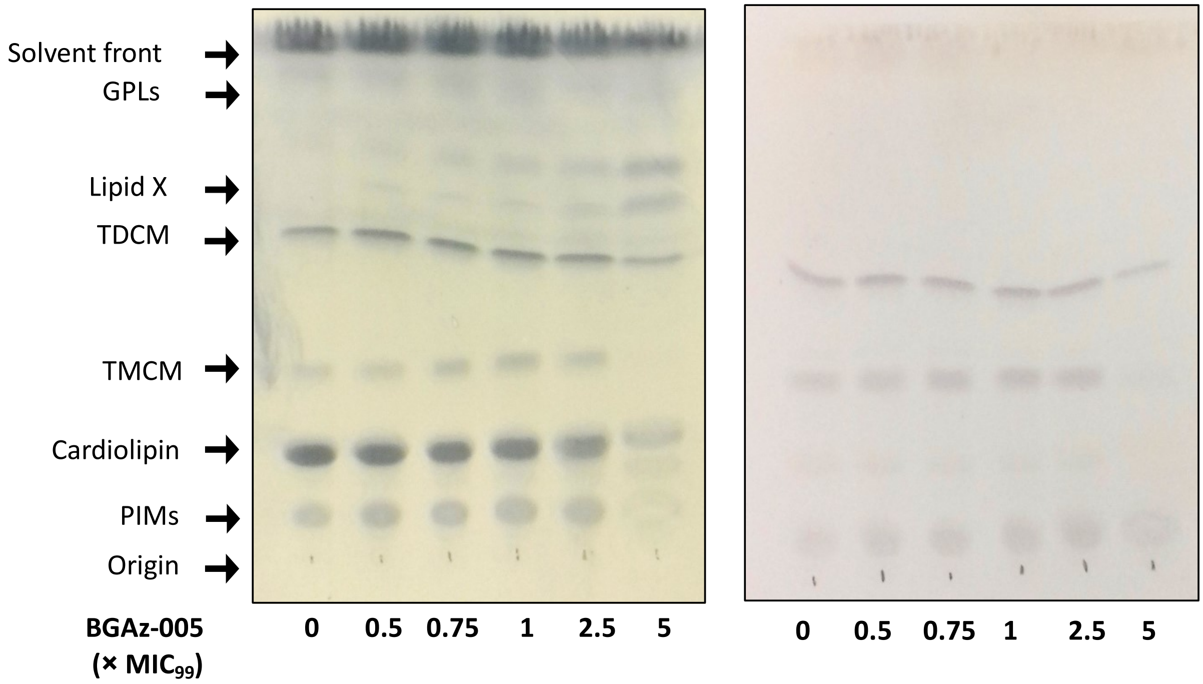

Supplementary Figure 6. C. glutamicum cell envelope lipid analysis upon exposure to **BGAz-005.** *M. smegmatis* were cultured in BHI broth, exposed to increasing concentrations of **BGAz-005** for 6 h and the cell envelope lipids selectively removed by solvent extraction. Equal volumes of lipid adjusted by bacterial growth were separated by TLC (chloroform/methanol/water, 60:16:2, v/v/v), and stained with MPA (A) or alpha-naphthol (B).

Supplementary Table 2. MIC values of the **BGAz-002–BGAz-005** and ebselen against mycobacterial and corynebacterial strains. NT = not tested.

Compound	*C. glutamicum*	*C. glutamicum*△*pks13*	BCG pVV16-*mmpL3*	BCG pTIC6-*fbpA*	BCG pTIC6-*fbpB*	BCG pTIC6-*fbpC*
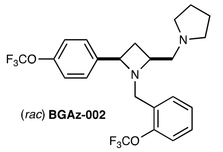 (*rac*) **BGAz-002**	50	50	25	NT	NT	NT
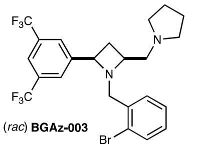 (*rac*) **BGAz-003**	14	10	40	NT	NT	NT
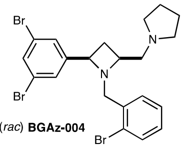 (*rac*) **BGAz-004**	20	10	40	20	28	34
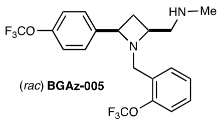 (*rac*) **BGAz-005**	12	10	NT	25	50	50
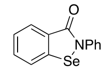 **Ebselen**	NT	NT	NT	28	90	98

[(a)] MIC as determined by a modified Gompertz function

Supplementary Table 3. Physiochemical and toxicological properties of **BGAz-002–BGAz-005**.

Entry	Compound	Kinetic solubility (μM) [(a)]	Mouse PPB (%) [(b)]	Mouse Microsomal CL_{int} clearance (μL/min/mg) [(c)]	Mouse Hepatocyte CL_{int} clearance (μL/min/mg) [(d)]	Caco-2 B-A Paap 10^6 cm/s [(e)]	Caco-2 Efflux ratio [(e)]
1	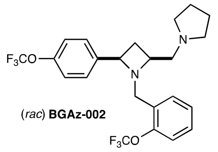 (*rac*) **BGAz-002**	57	>99	166	60	2.8	1.2
2	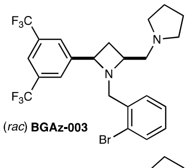 (*rac*) **BGAz-003**	27	>99	179	52	1.1	3.6
3	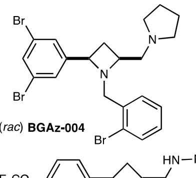 (*rac*) **BGAz-004**	9	>99	192	35	0.5	1.4
4	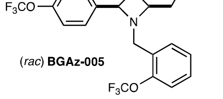 (*rac*) **BGAz-005**	117	>99	36	37	1.4	0.9

[(a)] Kinetic solubility measures turbidimetry providing a Log S which is then converted into a solubility value; [(b)] Rapid Equilibrium Dialysis (RED) measures percentage binding of compound to plasma proteins in human and preclinical species; [(c)] microsomal stability assay monitors the disappearance of a compound in the presence and absence of NADPH; [(d)] hepatocyte stability assay monitors the disappearance of a compound in the presence and absence of hepatocytes. [(e)] the human colon epithelial cancer cell line (Caco-2) is used as a model of human intestinal absorption of drugs.

NMR Spectrums

BGAz-001-**1H NMR**

NAME May06-2019
EXPNO 34
PROCNO 1
Date_ 20190506
Time 16.45
INSTRUM spect
PROBHD 5 mm DUL 13C-1
PULPROG zg30
TD 65536
SOLVENT CDCl3
NS 16
DS 2
SWH 8278.146 Hz
FIDRES 0.126314 Hz
AQ 3.9584243 sec
RG 71.8
DW 60.400 usec
DE 6.50 usec
TE 299.2 K
D1 1.00000000 sec
TD0 1

======== CHANNEL f1 ========
NUC1 1H
P1 12.58 usec
PL1 0.00 dB
PL1W 10.87646866 W
SFO1 400.1324710 MHz
SI 32768
SF 400.1300000 MHz
WDW EM
SSB 0
LB 0.30 Hz
GB 0
PC 1.00

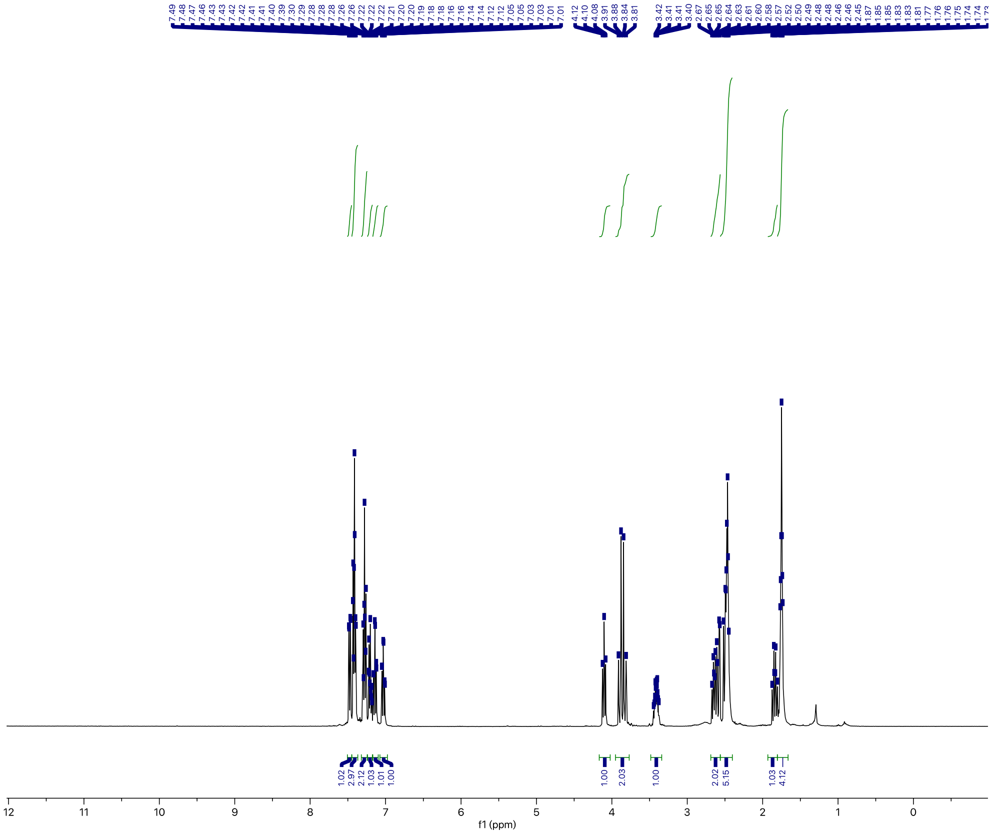

BGAz-001-**1H NMR zoom 1**

NAME May06-2019
EXPNO 34
PROCNO 1
Date_ 20190506
Time 16.45
INSTRUM spect
PROBHD 5 mm DUL 13C-1
PULPROG zg30
TD 65536
SOLVENT CDCl3
NS 16
DS 2
SWH 8278.146 Hz
FIDRES 0.126314 Hz
AQ 3.9584243 sec
RG 71.8
DW 60.400 usec
DE 6.50 usec
TE 299.2 K
D1 1.00000000 sec
TD0 1

======== CHANNEL f1 ========
NUC1 1H
P1 12.58 usec
PL1 0.00 dB
PL1W 10.87646866 W
SFO1 400.1324710 MHz
SI 32768
SF 400.1300000 MHz
WDW EM
SSB 0
LB 0.30 Hz
GB 0
PC 1.00

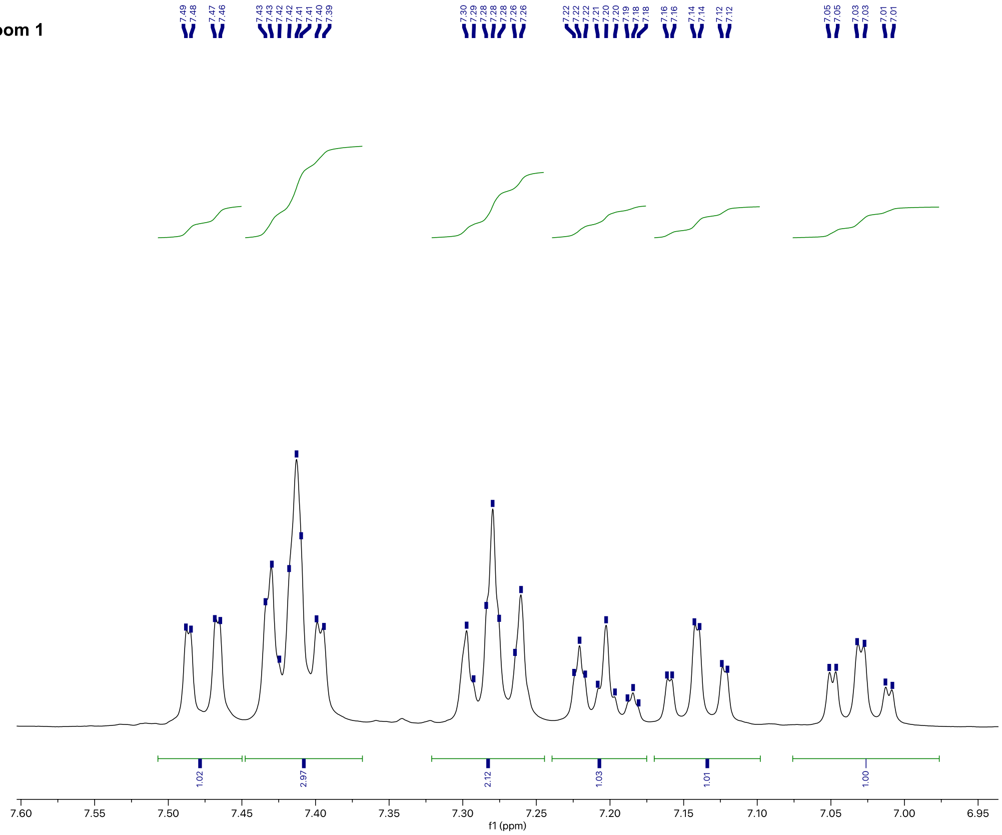

BGAz-001-**1H NMR zoom 2**

```
NAME May06-2019
EXPNO 34
PROCNO 1
Date_ 20190506
Time 16.45
INSTRUM spect
PROBHD 5 mm DUL 13C-1
PULPROG zg30
TD 65536
SOLVENT CDCl3
NS 16
DS 2
SWH 8278.146 Hz
FIDRES 0.126314 Hz
AQ 3.9584243 sec
RG 71.8
DW 60.400 usec
DE 6.50 usec
TE 299.2 K
D1 1.00000000 sec
TD0 1

======== CHANNEL f1 ========
NUC1 1H
P1 12.58 usec
PL1 0.00 dB
PL1W 10.87646866 W
SFO1 400.1324710 MHz
SI 32768
SF 400.1300000 MHz
WDW EM
SSB 0
LB 0.30 Hz
GB 0
PC 1.00
```

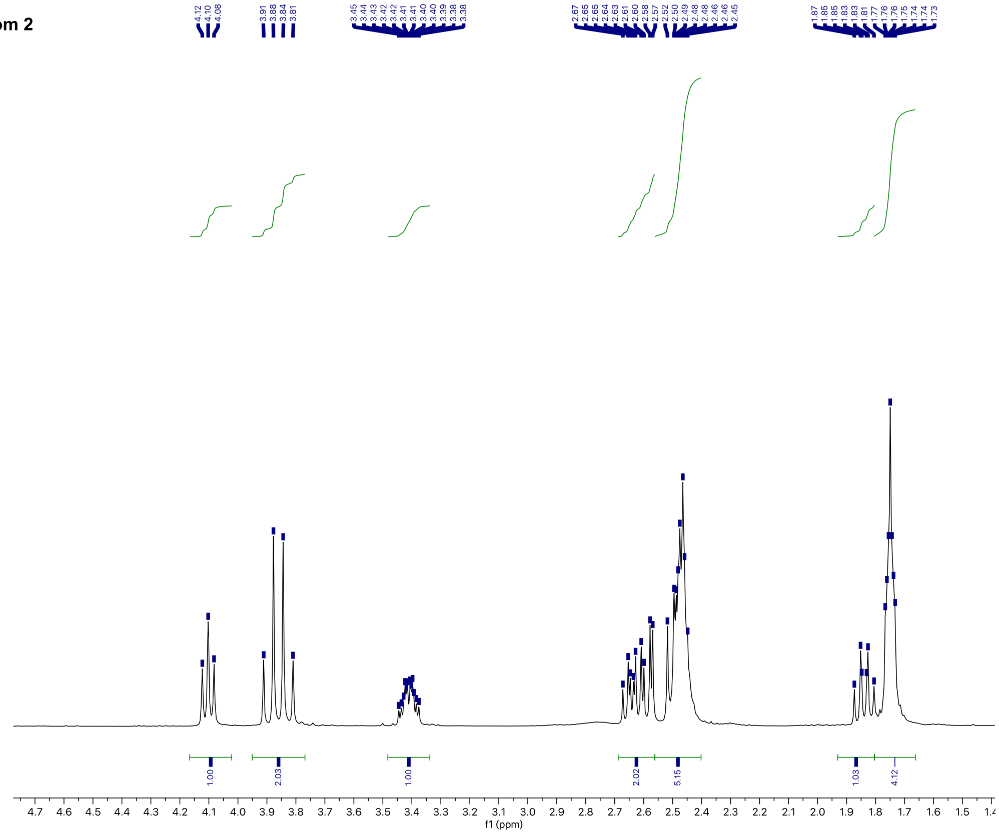

BGAz-001-**13C NMR**

```
Current Data Parameters
NAME May06-2019
EXPNO 7
PROCNO 1

F2 - Acquisition Parameters
Date_ 20190506
Time 20.49 h
INSTRUM Spect
PROBHD Z109128_0101 (
PULPROG zgpg30
TD 65536
SOLVENT CDCl3
NS 4096
DS 4
SWH 29761.904 Hz
FIDRES 0.908261 Hz
AQ 1.1010048 sec
RG 203
DW 16.800 usec
DE 6.50 usec
TE 297.7 K
D1 2.00000000 sec
D11 0.03000000 sec
TD0 1
SFO1 125.7703643 MHz
NUC1 13C
P1 13.50 usec
PLW1 76.00000000 W
SFO2 500.1320005 MHz
NUC2 1H
CPDPRG[2 waltz16
PCPD2 80.00 usec
PLW2 18.00000000 W
PLW12 0.43945000 W
PLW13 0.22104000 W

F2 - Processing parameters
SI 32768
SF 125.7577976 MHz
WDW EM
SSB 0
LB 1.00 Hz
GB 0
PC 1.40
```

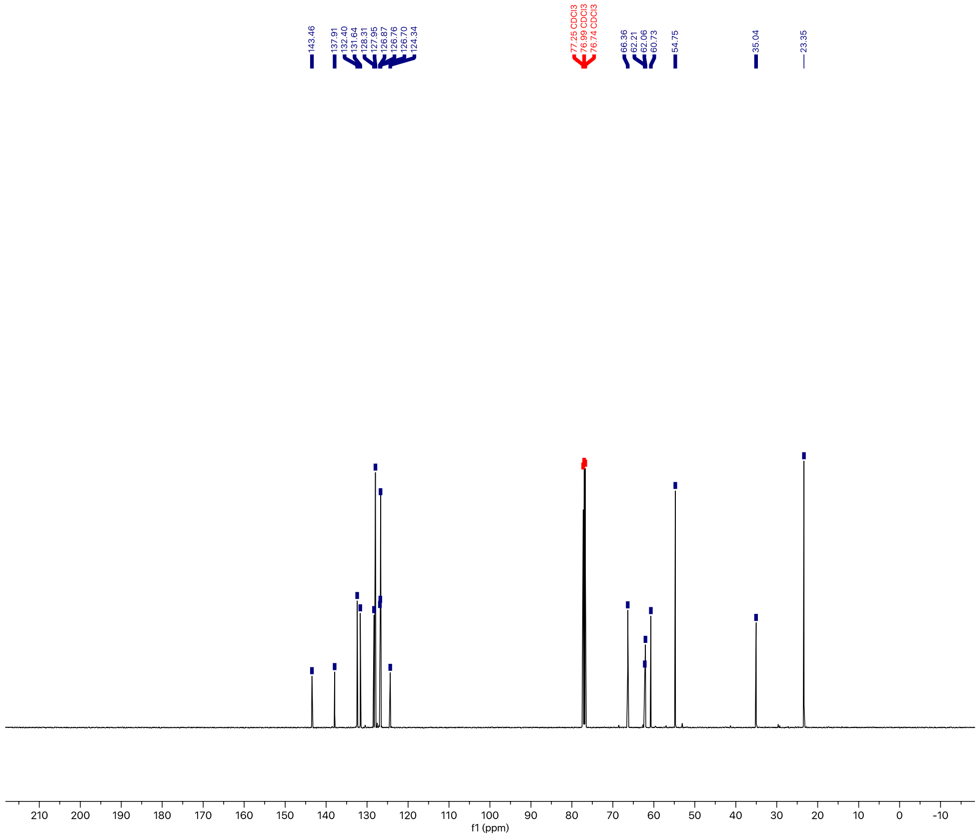

BGAz-002-**1H NMR zoom 1**

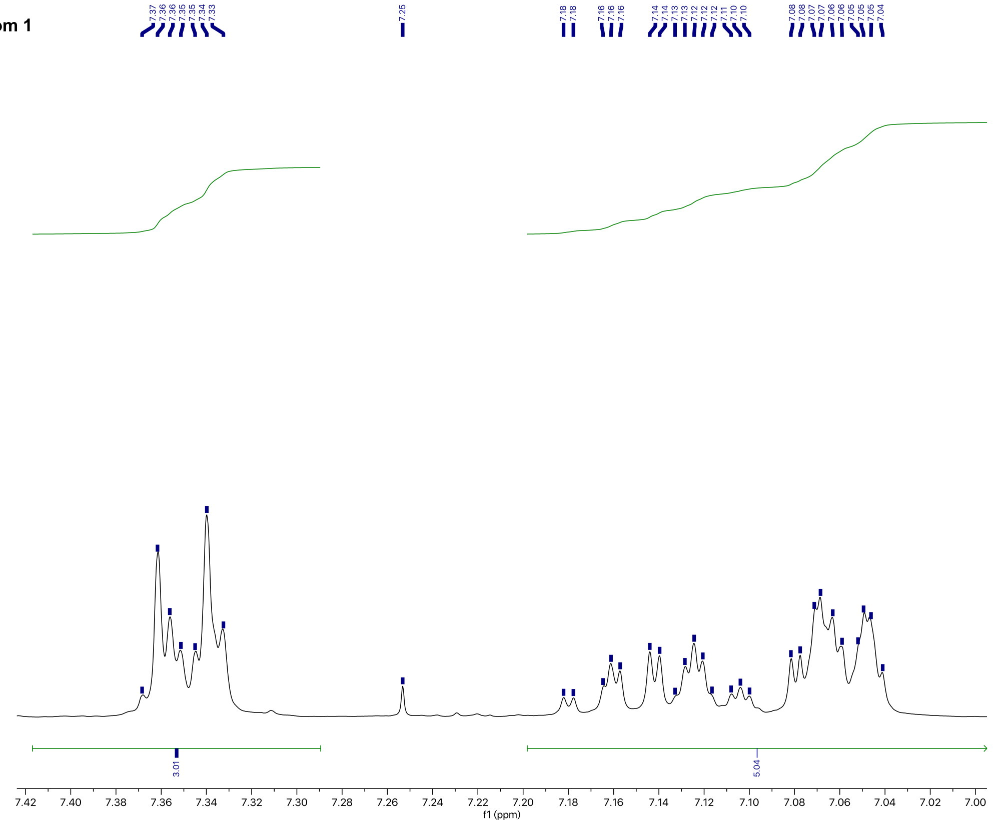

```
Current Data Parameters
NAME YC-GIBH-A08
EXPNO 10
PROCNO 1

F2 - Acquisition Parameters
Date_ 20190405
Time 8.20 h
INSTRUM AvanceNeo
PROBHD Z116098_0793 (
PULPROG zg30
TD 65536
SOLVENT CDCl3
NS 2
DS 0
SWH 7142.857 Hz
FIDRES 0.217983 Hz
AQ 4.5875201 sec
RG 32
DW 70.000 usec
DE 14.62 usec
TE 298.0 K
D1 2.00000000 sec
TD0 1
SFO1 400.1324008 MHz
NUC1 1H
P0 3.33 usec
P1 10.00 usec
PLW1 18.69700050 W

F2 - Processing parameters
SI 131072
SF 400.1300126 MHz
WDW EM
SSB 0
LB 0.10 Hz
GB 0
PC 1.00
```

BGAz-002-1H NMR zoom 2

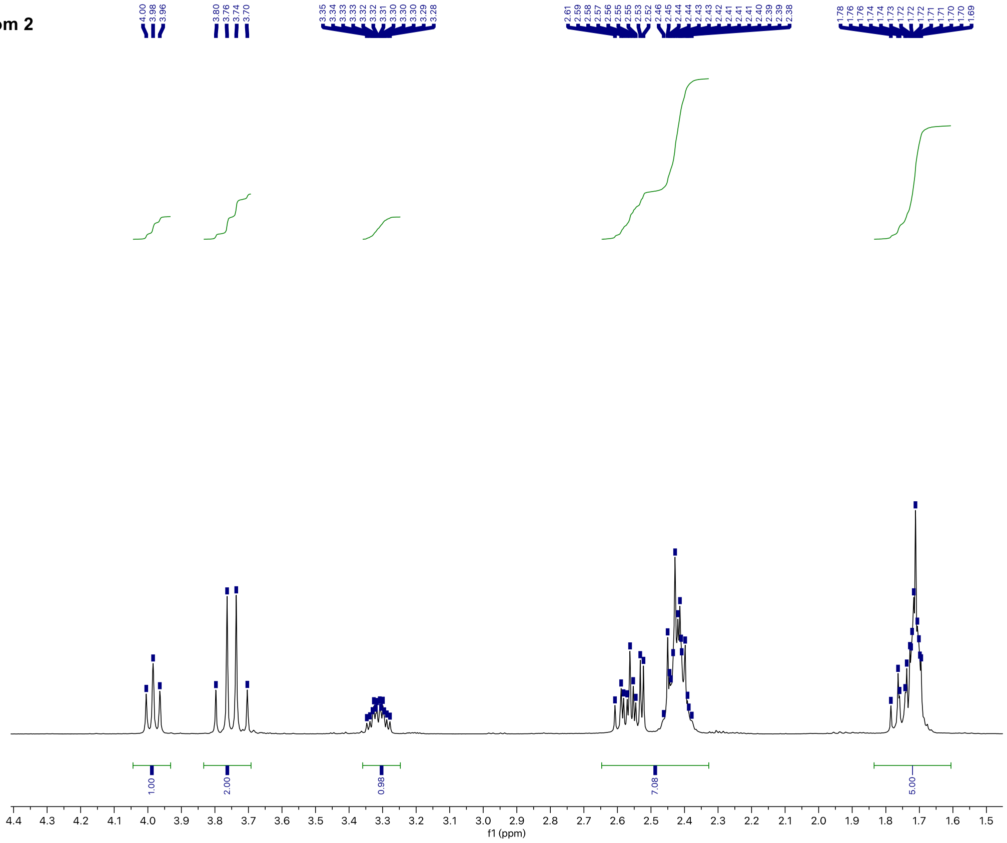


```
Current Data Parameters
NAME YC-GIBH-A08
EXPNO 10
PROCNO 1

F2 - Acquisition Parameters
Date_ 20190405
Time 8.20 h
INSTRUM AvanceNeo
PROBHD Z116098_0793 (
PULPROG zg30
TD 65536
SOLVENT CDCl3
NS 2
DS 0
SWH 7142.857 Hz
FIDRES 0.217983 Hz
AQ 4.5875201 sec
RG 32
DW 70.000 usec
DE 14.62 usec
TE 298.0 K
D1 2.00000000 sec
TD0 1
SFO1 400.1324008 MHz
NUC1 1H
P0 3.33 usec
P1 10.00 usec
PLW1 18.69700050 W

F2 - Processing parameters
SI 131072
SF 400.1300126 MHz
WDW EM
SSB 0
LB 0.10 Hz
GB 0
PC 1.00
```

BGAz-002-**1H NMR**

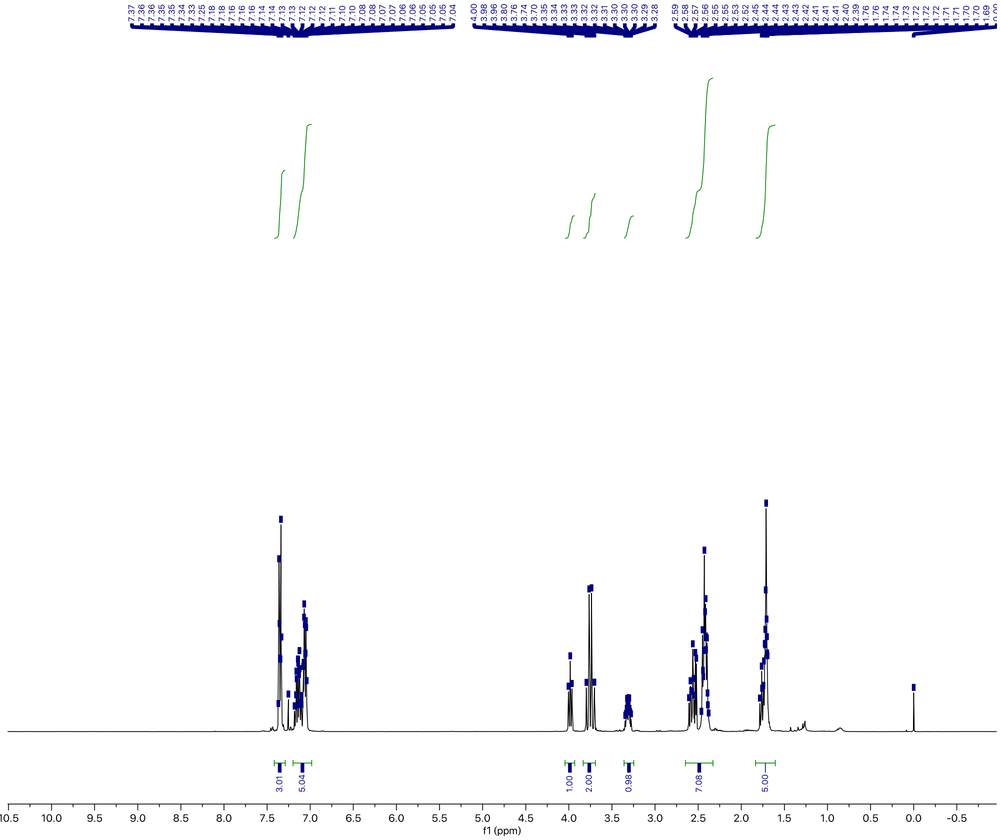

Current Data Parameters
NAME YC-GIBH-A08
EXPNO 10
PROCNO 1

F2 - Acquisition Parameters
Date_ 20190405
Time 8.20 h
INSTRUM AvanceNeo
PROBHD Z116098_0793 (
PULPROG zg30
TD 65536
SOLVENT CDCl3
NS 2
DS 0
SWH 7142.857 Hz
FIDRES 0.217983 Hz
AQ 4.5875201 sec
RG 32
DW 70.000 usec
DE 14.62 usec
TE 298.0 K
D1 2.00000000 sec
TD0 1
SFO1 400.1324008 MHz
NUC1 1H
P0 3.33 usec
P1 10.00 usec
PLW1 18.69700050 W

F2 - Processing parameters
SI 131072
SF 400.1300126 MHz
WDW EM
SSB 0
LB 0.10 Hz
GB 0
PC 1.00

BGAz-002-**13C NMR**

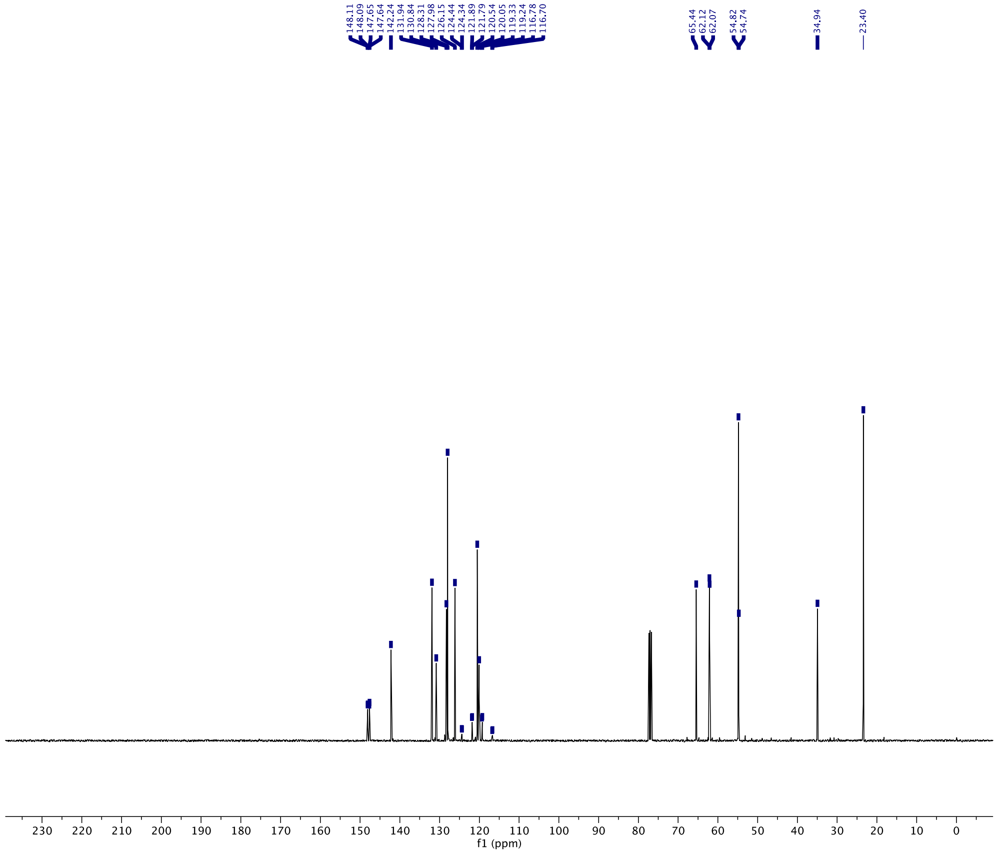

Current Data Parameters
NAME YC-GIBH-A08
EXPNO 11
PROCNO 1

F2 - Acquisition Parameters
Date_ 20190405
Time 8.50 h
INSTRUM AvanceNeo
PROBHD Z116098_0793 (
PULPROG zgpg30
TD 119044
SOLVENT CDCl3
NS 512
DS 0
SWH 25000.000 Hz
FIDRES 0.420013 Hz
AQ 2.3808801 sec
RG 31.9602
DW 20.000 usec
DE 7.12 usec
TE 298.0 K
D1 1.00000000 sec
D11 0.03000000 sec
TD0 1
SFO1 100.6243390 MHz
NUC1 13C
P0 3.33 usec
P1 10.00 usec
PLW1 83.92700195 W
SFO2 400.1318006 MHz
NUC2 1H
CPDPRG[2 waltz64
PCPD2 90.00 usec
PLW2 18.69700050 W
PLW12 0.23083000 W
PLW13 0.11611000 W

F2 - Processing parameters
SI 131072
SF 100.6127685 MHz
WDW EM
SSB 0
LB 1.00 Hz
GB 0
PC 1.40

BGAz-002-**19F NMR**

Current Data Parameters
NAME YC-GIBH-A08
EXPNO 13
PROCNO 1

F2 - Acquisition Parameters
Date_ 20190405
Time 9.12 h
INSTRUM AvanceNeo
PROBHD Z116098_0793 (
PULPROG zgig30
TD 261948
SOLVENT CDCl3
NS 16
DS 0
SWH 156250.000 Hz
FIDRES 1.192985 Hz
AQ 0.8382336 sec
RG 101
DW 3.200 usec
DE 6.82 usec
TE 298.0 K
D1 2.00000000 sec
D11 0.03000000 sec
TD0 1
SFO1 376.5021312 MHz
NUC1 19F
P0 6.00 usec
P1 18.00 usec
PLW1 18.94099998 W
SFO2 400.1318006 MHz
NUC2 1H
CPDPRG[2 waltz16
PCPD2 90.00 usec
PLW2 18.69700050 W
PLW12 0.23083000 W

F2 - Processing parameters
SI 262144
SF 376.4983662 MHz
WDW EM
SSB 0
LB 1.00 Hz
GB 0
PC 2.00

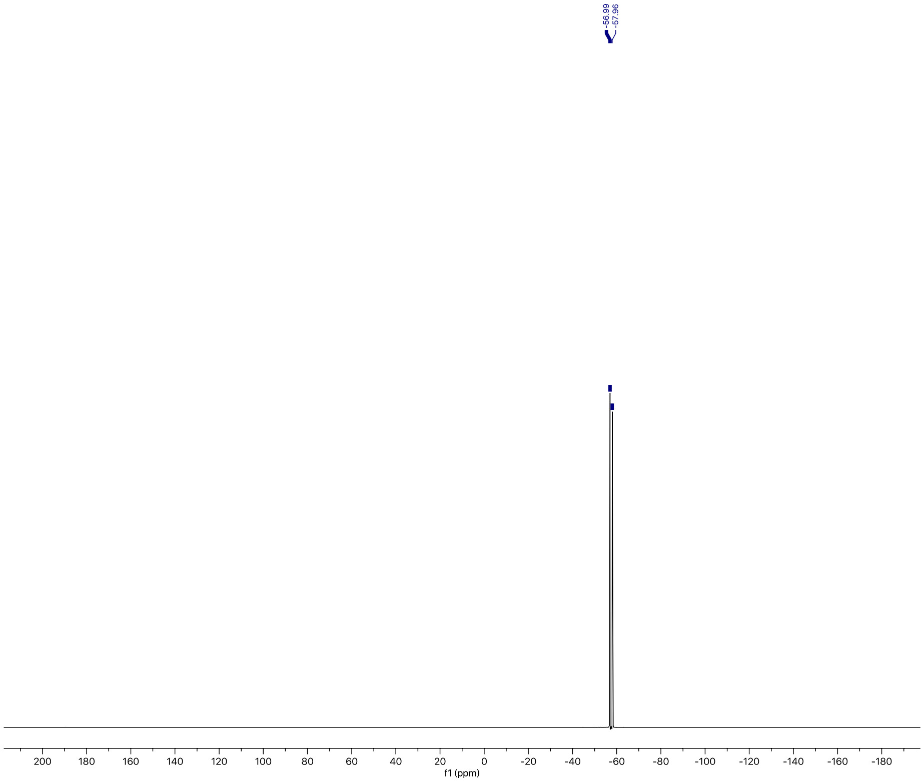

BGAz-003-**1H NMR**

Current Data Parameters
NAME CYX91
EXPNO 10
PROCNO 1

F2 - Acquisition Parameters
Date_ 20190404
Time 1.01 h
INSTRUM AvanceNeo
PROBHD Z116098_0793 (
PULPROG zg30
TD 65536
SOLVENT CDCl3
NS 2
DS 0
SWH 7142.857 Hz
FIDRES 0.217983 Hz
AQ 4.5875201 sec
RG 92.3077
DW 70.000 usec
DE 14.62 usec
TE 298.0 K
D1 2.00000000 sec
TD0 1
SFO1 400.1324008 MHz
NUC1 1H
P0 3.33 usec
P1 10.00 usec
PLW1 18.69700050 W

F2 - Processing parameters
SI 131072
SF 400.1300093 MHz
WDW EM
SSB 0
LB 0.10 Hz
GB 0
PC 1.00

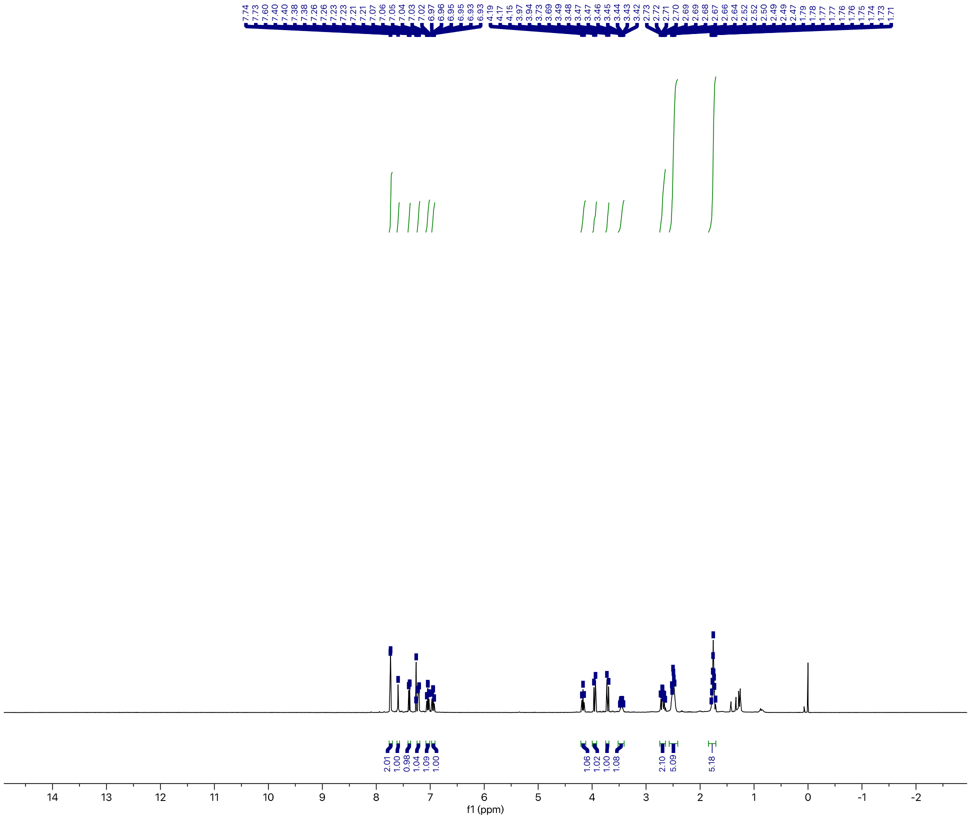

BGAz-003-**1H NMR zoom 1**

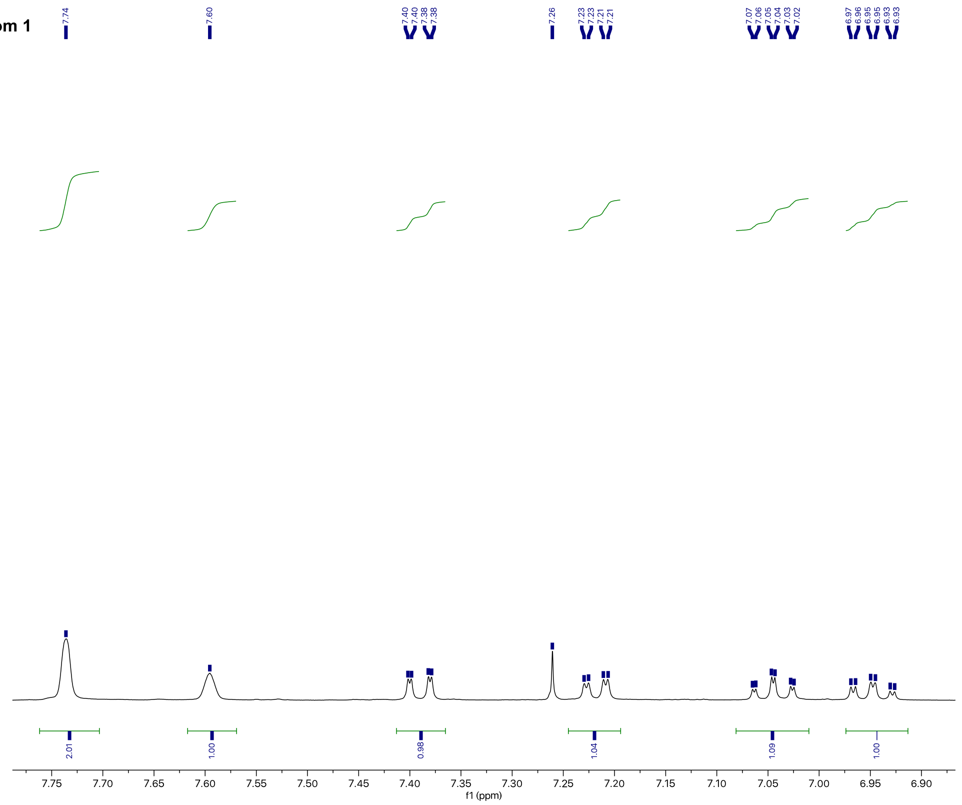


```
Current Data Parameters
NAME CYX91
EXPNO 10
PROCNO 1

F2 - Acquisition Parameters
Date_ 20190404
Time 1.01 h
INSTRUM AvanceNeo
PROBHD Z116098_0793 (
PULPROG zg30
TD 65536
SOLVENT CDCl3
NS 2
DS 0
SWH 7142.857 Hz
FIDRES 0.217983 Hz
AQ 4.5875201 sec
RG 92.3077
DW 70.000 usec
DE 14.62 usec
TE 298.0 K
D1 2.00000000 sec
TD0 1
SFO1 400.1324008 MHz
NUC1 1H
P0 3.33 usec
P1 10.00 usec
PLW1 18.69700050 W

F2 - Processing parameters
SI 131072
SF 400.1300093 MHz
WDW EM
SSB 0
LB 0.10 Hz
GB 0
PC 1.00
```

BGAz-003-1H NMR zoom 2

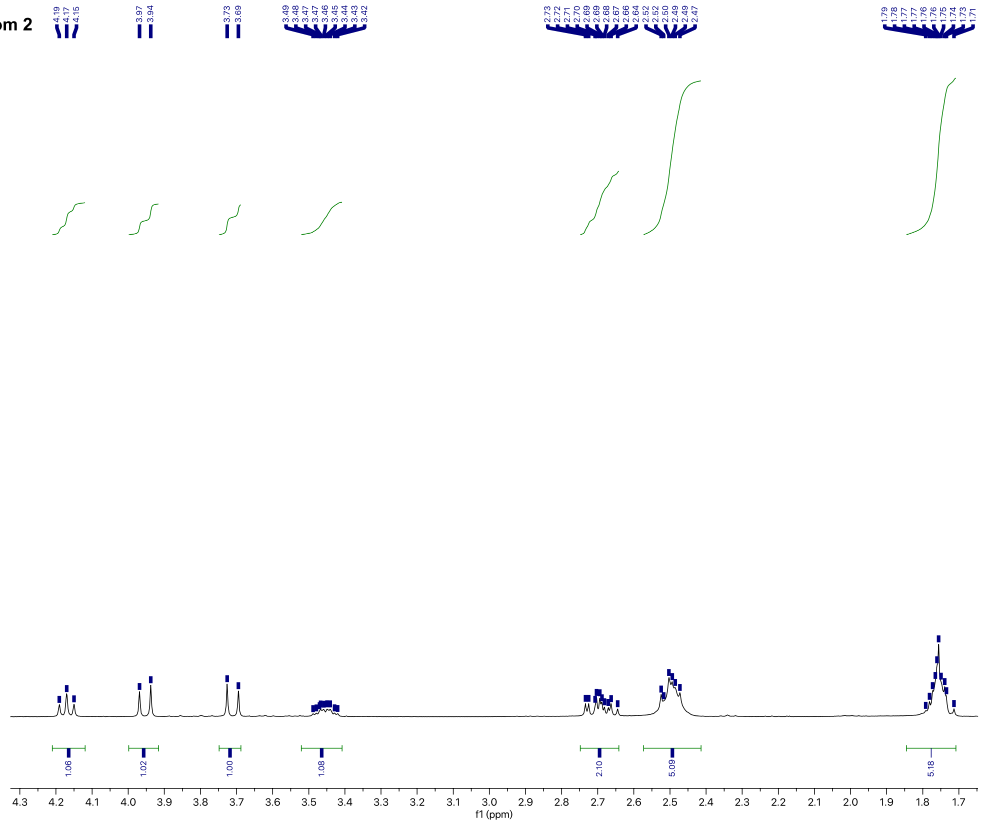


```
Current Data Parameters
NAME CYX91
EXPNO 10
PROCNO 1

F2 - Acquisition Parameters
Date_ 20190404
Time 1.01 h
INSTRUM AvanceNeo
PROBHD Z116098_0793 (
PULPROG zg30
TD 65536
SOLVENT CDCl3
NS 2
DS 0
SWH 7142.857 Hz
FIDRES 0.217983 Hz
AQ 4.5875201 sec
RG 92.3077
DW 70.000 usec
DE 14.62 usec
TE 298.0 K
D1 2.00000000 sec
TD0 1
SFO1 400.1324008 MHz
NUC1 1H
P0 3.33 usec
P1 10.00 usec
PLW1 18.69700050 W

F2 - Processing parameters
SI 131072
SF 400.1300093 MHz
WDW EM
SSB 0
LB 0.10 Hz
GB 0
PC 1.00
```

BGAz-003-**13C NMR**

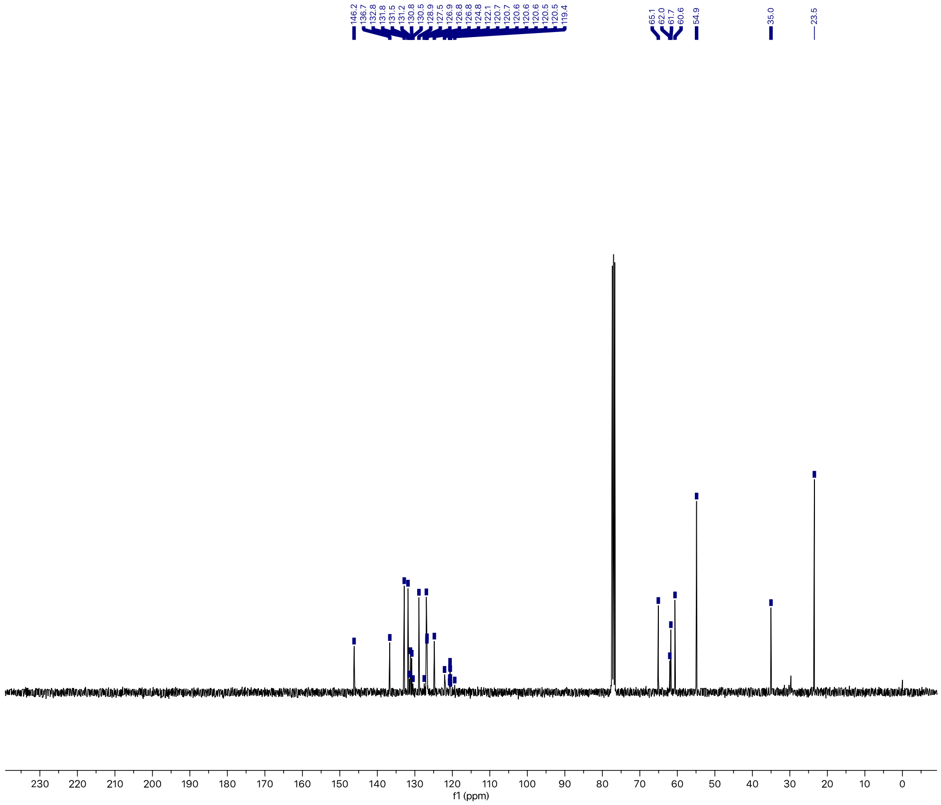


```
Current Data Parameters
NAME CYX91
EXPNO 12
PROCNO 1

F2 - Acquisition Parameters
Date_ 20190404
Time 1.33 h
INSTRUM AvanceNeo
PROBHD Z116098_0793 (
PULPROG zgpg30
TD 119044
SOLVENT CDCl3
NS 512
DS 0
SWH 25000.000 Hz
FIDRES 0.420013 Hz
AQ 2.3808801 sec
RG 31.9602
DW 20.000 usec
DE 7.12 usec
TE 298.0 K
D1 1.00000000 sec
D11 0.03000000 sec
TD0 1
SFO1 100.6243390 MHz
NUC1 13C
P0 3.33 usec
P1 10.00 usec
PLW1 83.92700195 W
SFO2 400.1318006 MHz
NUC2 1H
CPDPRG[2 waltz64
PCPD2 90.00 usec
PLW2 18.69700050 W
PLW12 0.23083000 W
PLW13 0.11611000 W

F2 - Processing parameters
SI 131072
SF 100.6127685 MHz
WDW EM
SSB 0
LB 1.00 Hz
GB 0
PC 1.40
```

BGAz-003-**19F NMR**

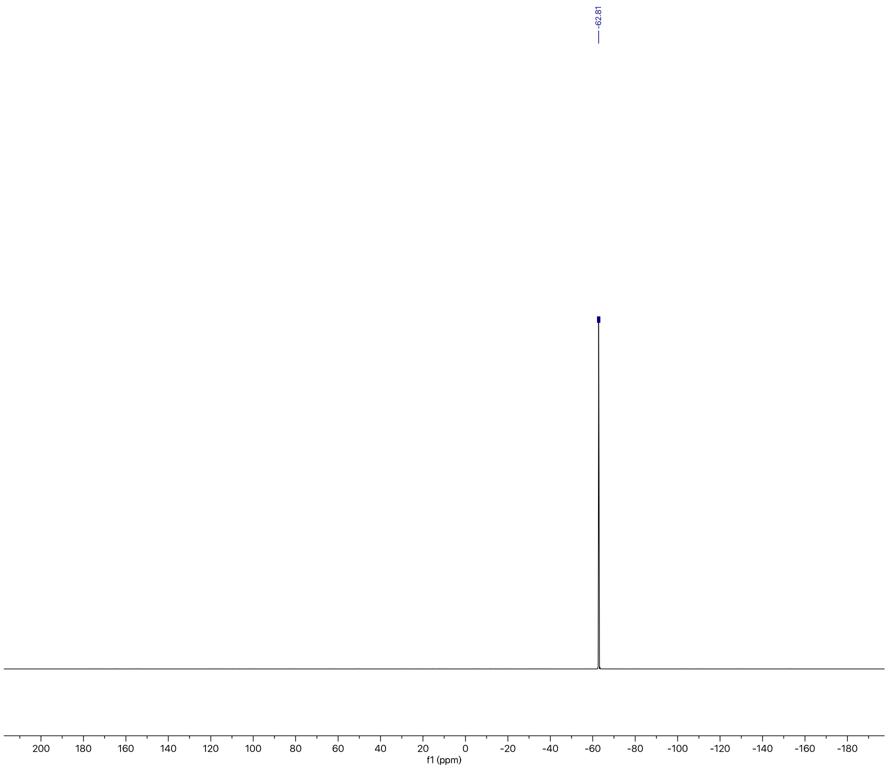


```
Current Data Parameters
NAME CYX91
EXPNO 11
PROCNO 1

F2 - Acquisition Parameters
Date_ 20190404
Time 1.02 h
INSTRUM AvanceNeo
PROBHD Z116098_0793 (
PULPROG zgig30
TD 261948
SOLVENT CDCl3
NS 16
DS 0
SWH 156250.000 Hz
FIDRES 1.192985 Hz
AQ 0.8382336 sec
RG 101
DW 3.200 usec
DE 6.82 usec
TE 298.0 K
D1 2.00000000 sec
D11 0.03000000 sec
TD0 1
SFO1 376.5021312 MHz
NUC1 19F
P0 6.00 usec
P1 18.00 usec
PLW1 18.94099998 W
SFO2 400.1318006 MHz
NUC2 1H
CPDPRG[2 waltz16
PCPD2 90.00 usec
PLW2 18.69700050 W
PLW12 0.23083000 W

F2 - Processing parameters
SI 262144
SF 376.4983662 MHz
WDW EM
SSB 0
LB 1.00 Hz
GB 0
PC 2.00
```

BGAz-004-**1H NMR**

Current Data Parameters
NAME CYX92
EXPNO 10
PROCNO 1

F2 - Acquisition Parameters
Date_ 20190404
Time 1.40 h
INSTRUM AvanceNeo
PROBHD Z116098_0793 (
PULPROG zg30
TD 65536
SOLVENT CDCl3
NS 2
DS 0
SWH 7142.857 Hz
FIDRES 0.217983 Hz
AQ 4.5875201 sec
RG 101
DW 70.000 usec
DE 14.62 usec
TE 298.0 K
D1 2.00000000 sec
TD0 1
SFO1 400.1324008 MHz
NUC1 1H
P0 3.33 usec
P1 10.00 usec
PLW1 18.69700050 W

F2 - Processing parameters
SI 131072
SF 400.1300098 MHz
WDW EM
SSB 0
LB 0.10 Hz
GB 0
PC 1.00

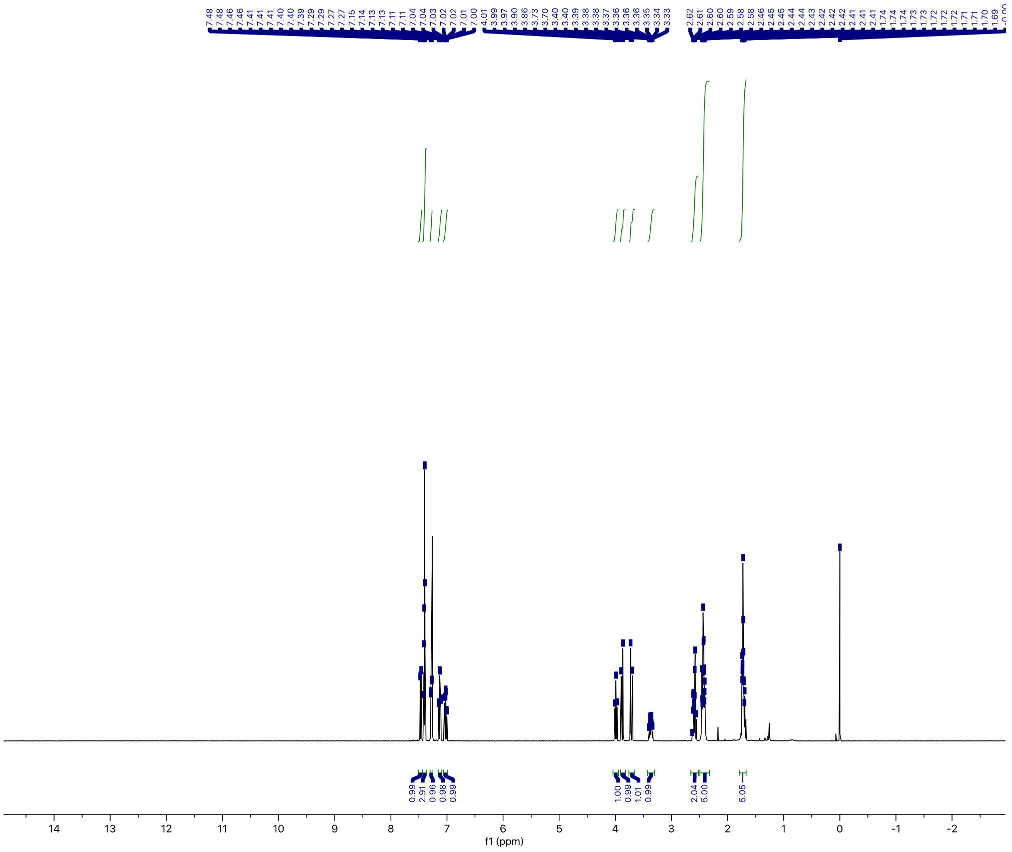

BGAz-004-**1H NMR zoom 1**

Current Data Parameters
NAME CYX92
EXPNO 10
PROCNO 1

F2 - Acquisition Parameters
Date_ 20190404
Time 1.40 h
INSTRUM AvanceNeo
PROBHD Z116098_0793 (
PULPROG zg30
TD 65536
SOLVENT CDCl3
NS 2
DS 0
SWH 7142.857 Hz
FIDRES 0.217983 Hz
AQ 4.5875201 sec
RG 101
DW 70.000 usec
DE 14.62 usec
TE 298.0 K
D1 2.00000000 sec
TD0 1
SFO1 400.1324008 MHz
NUC1 1H
P0 3.33 usec
P1 10.00 usec
PLW1 18.69700050 W

F2 - Processing parameters
SI 131072
SF 400.1300098 MHz
WDW EM
SSB 0
LB 0.10 Hz
GB 0
PC 1.00

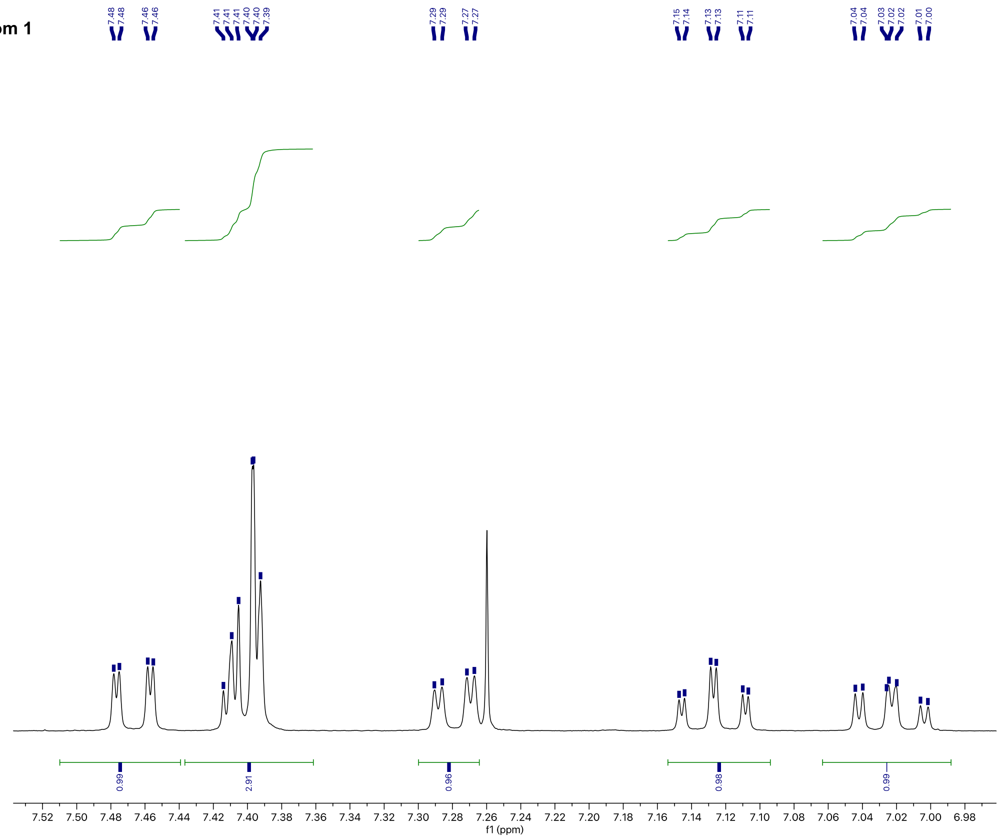

BGAz-004-**1H NMR zoom 2**

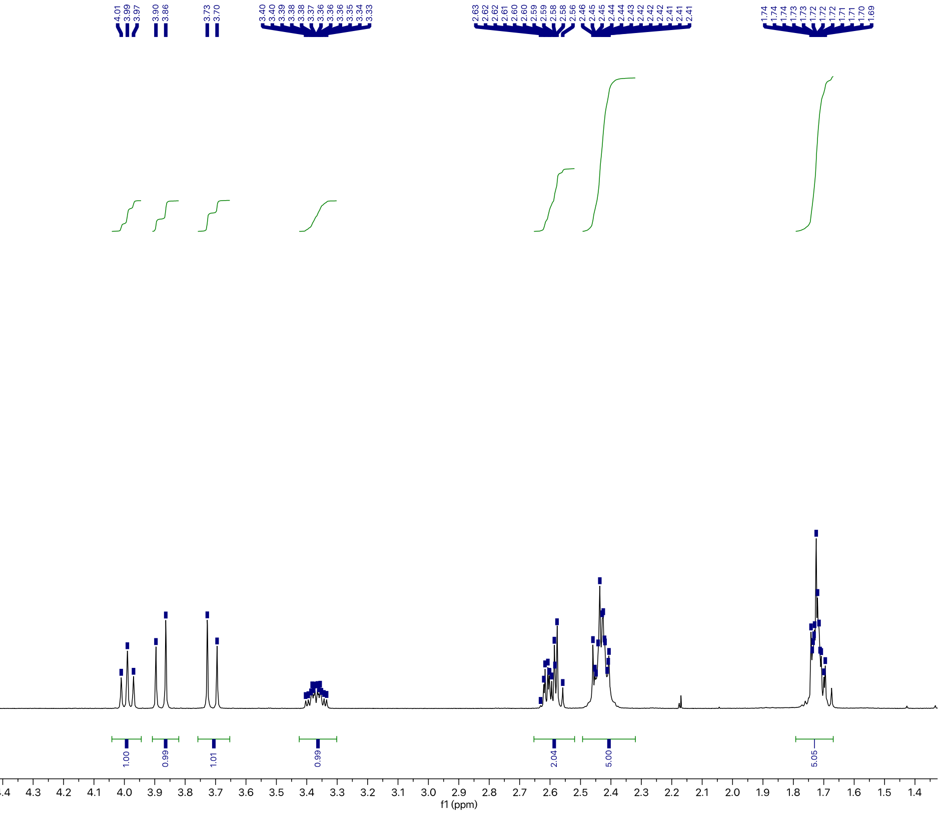

Current Data Parameters
NAME CYX92
EXPNO 10
PROCNO 1

F2 - Acquisition Parameters
Date_ 20190404
Time 1.40 h
INSTRUM AvanceNeo
PROBHD Z116098_0793 (
PULPROG zg30
TD 65536
SOLVENT CDCl3
NS 2
DS 0
SWH 7142.857 Hz
FIDRES 0.217983 Hz
AQ 4.5875201 sec
RG 101
DW 70.000 usec
DE 14.62 usec
TE 298.0 K
D1 2.00000000 sec
TD0 1
SFO1 400.1324008 MHz
NUC1 1H
P0 3.33 usec
P1 10.00 usec
PLW1 18.69700050 W

F2 - Processing parameters
SI 131072
SF 400.1300098 MHz
WDW EM
SSB 0
LB 0.10 Hz
GB 0
PC 1.00

BGAz-004-**13C NMR**

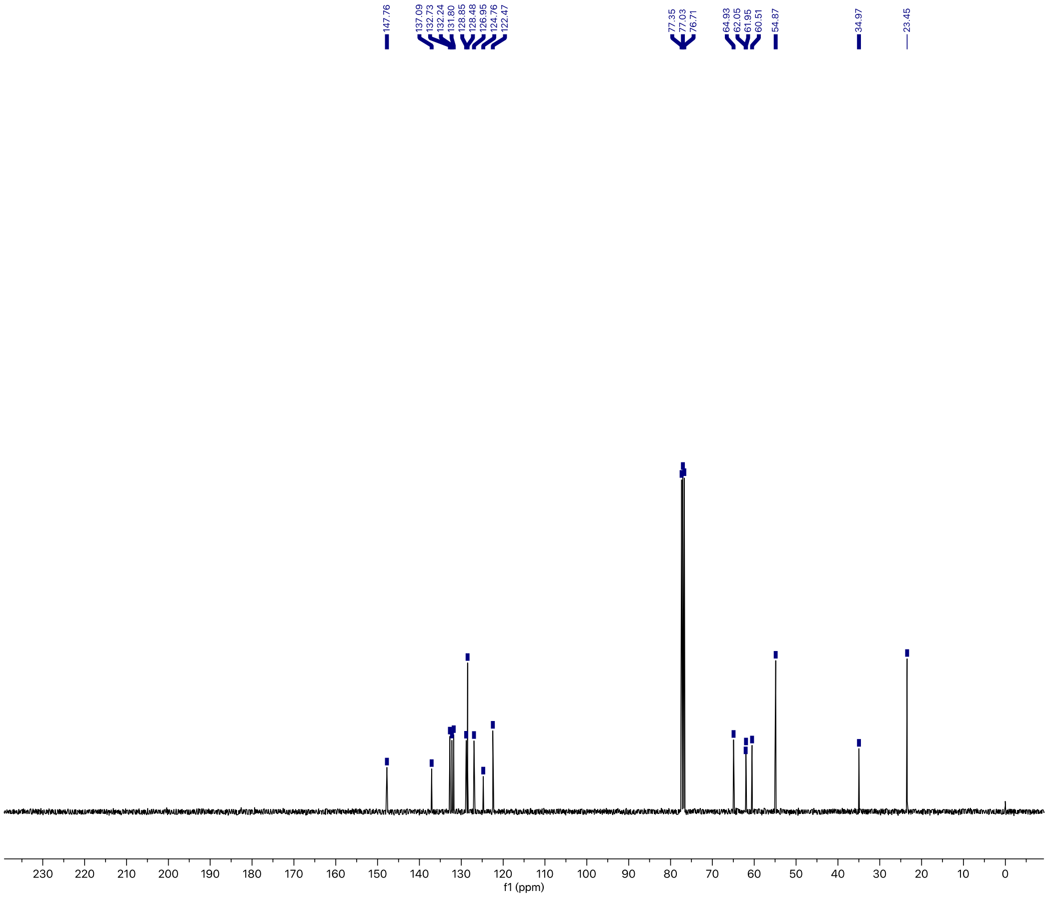

Current Data Parameters
NAME CYX92
EXPNO 11
PROCNO 1

F2 - Acquisition Parameters
Date_ 20190404
Time 2.11 h
INSTRUM AvanceNeo
PROBHD Z116098_0793 (
PULPROG zgpg30
TD 119044
SOLVENT CDCl3
NS 512
DS 0
SWH 25000.000 Hz
FIDRES 0.420013 Hz
AQ 2.3808801 sec
RG 31.9602
DW 20.000 usec
DE 7.12 usec
TE 298.0 K
D1 1.00000000 sec
D11 0.03000000 sec
TD0 1
SFO1 100.6243390 MHz
NUC1 13C
P0 3.33 usec
P1 10.00 usec
PLW1 83.92700195 W
SFO2 400.1318006 MHz
NUC2 1H
CPDPRG[2 waltz64
PCPD2 90.00 usec
PLW2 18.69700050 W
PLW12 0.23083000 W
PLW13 0.11611000 W

F2 - Processing parameters
SI 131072
SF 100.6127685 MHz
WDW EM
SSB 0
LB 1.00 Hz
GB 0
PC 1.40

BGAz-005-**1H NMR**

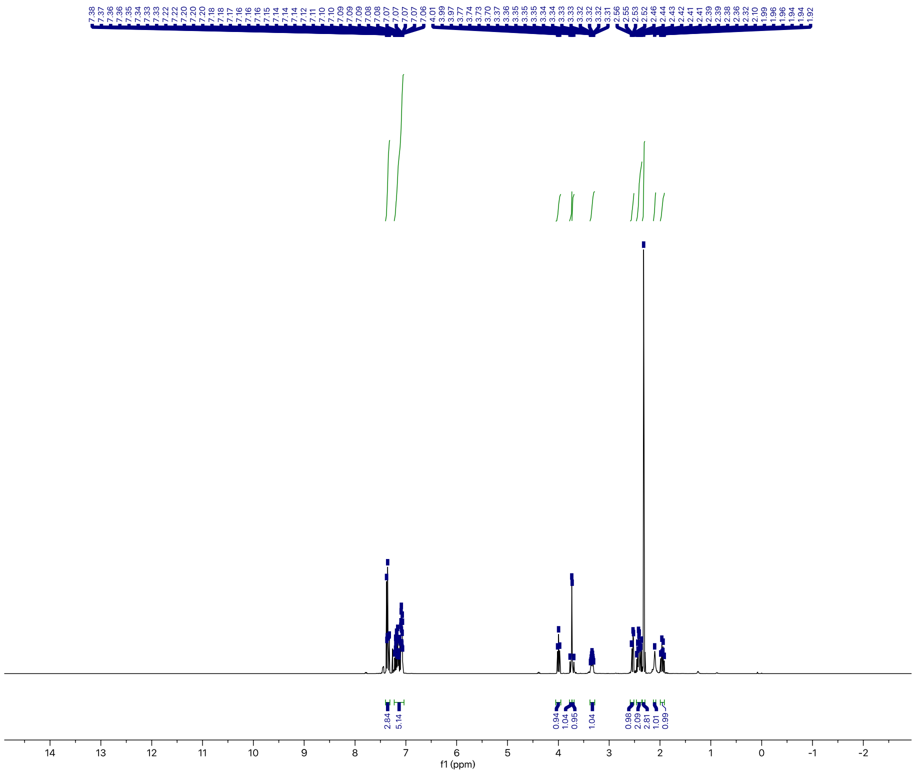

Current Data Parameters
NAME YC-GIBH-122
EXPNO 10
PROCNO 1

F2 - Acquisition Parameters
Date_ 20190615
Time 1.55 h
INSTRUM AvanceNeo
PROBHD Z116098_0793 (
PULPROG zg30
TD 65536
SOLVENT CDCl3
NS 2
DS 0
SWH 7142.857 Hz
FIDRES 0.217983 Hz
AQ 4.5875201 sec
RG 32
DW 70.000 usec
DE 14.62 usec
TE 298.0 K
D1 2.00000000 sec
TD0 1
SFO1 400.1324008 MHz
NUC1 1H
P0 3.33 usec
P1 10.00 usec
PLW1 18.69700050 W

F2 - Processing parameters
SI 131072
SF 400.1300419 MHz
WDW EM
SSB 0
LB 0.10 Hz
GB 0
PC 1.00

BGAz-005-**1H NMR zoom 1**

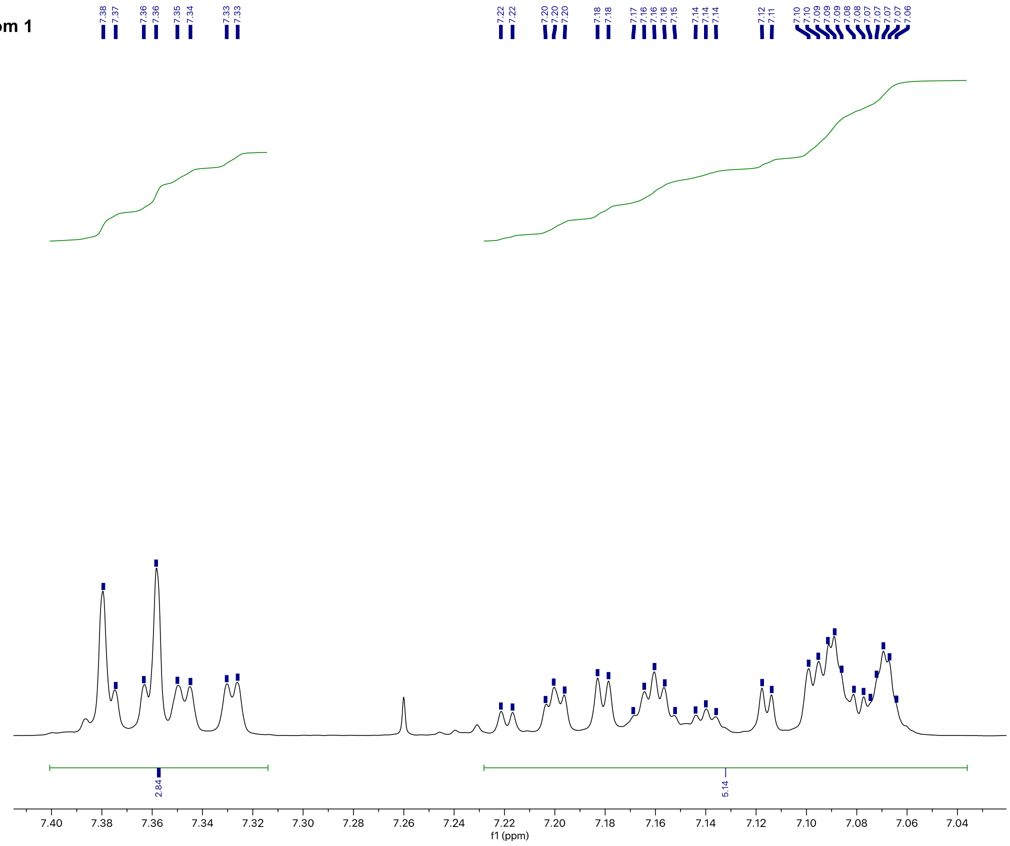

Current Data Parameters
NAME YC-GIBH-122
EXPNO 10
PROCNO 1

F2 - Acquisition Parameters
Date_ 20190615
Time 1.55 h
INSTRUM AvanceNeo
PROBHD Z116098_0793 (
PULPROG zg30
TD 65536
SOLVENT CDCl3
NS 2
DS 0
SWH 7142.857 Hz
FIDRES 0.217983 Hz
AQ 4.5875201 sec
RG 32
DW 70.000 usec
DE 14.62 usec
TE 298.0 K
D1 2.00000000 sec
TD0 1
SFO1 400.1324008 MHz
NUC1 1H
P0 3.33 usec
P1 10.00 usec
PLW1 18.69700050 W

F2 - Processing parameters
SI 131072
SF 400.1300419 MHz
WDW EM
SSB 0
LB 0.10 Hz
GB 0
PC 1.00

BGAz-005-**13C NMR**

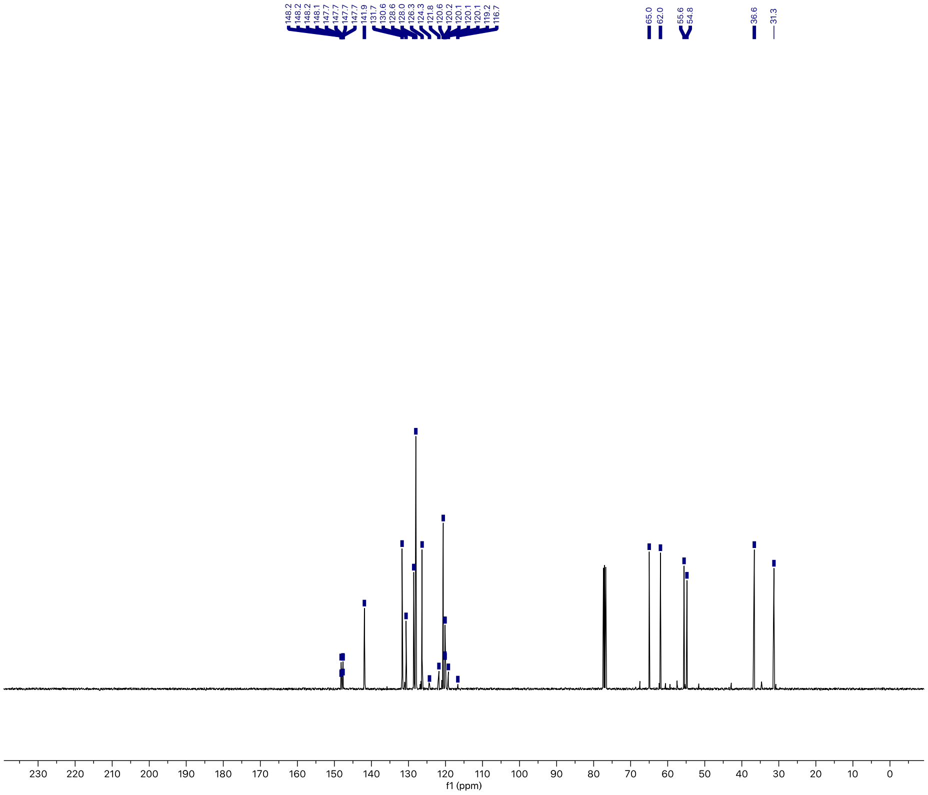

Current Data Parameters
NAME YC-GIBH-122
EXPNO 11
PROCNO 1

F2 - Acquisition Parameters
Date_ 20190615
Time 2.25 h
INSTRUM AvanceNeo
PROBHD Z116098_0793 (
PULPROG zgpg30
TD 119044
SOLVENT CDCl3
NS 512
DS 0
SWH 25000.000 Hz
FIDRES 0.420013 Hz
AQ 2.3808801 sec
RG 31.9602
DW 20.000 usec
DE 7.12 usec
TE 298.0 K
D1 1.00000000 sec
D11 0.03000000 sec
TD0 1
SFO1 100.6243390 MHz
NUC1 13C
P0 3.33 usec
P1 10.00 usec
PLW1 83.92700195 W
SFO2 400.1318006 MHz
NUC2 1H
CPDPRG[2 waltz64
PCPD2 90.00 usec
PLW2 18.69700050 W
PLW12 0.23083000 W
PLW13 0.11611000 W

F2 - Processing parameters
SI 131072
SF 100.6127685 MHz
WDW EM
SSB 0
LB 1.00 Hz
GB 0
PC 1.40

BGAz-005-**19F NMR**

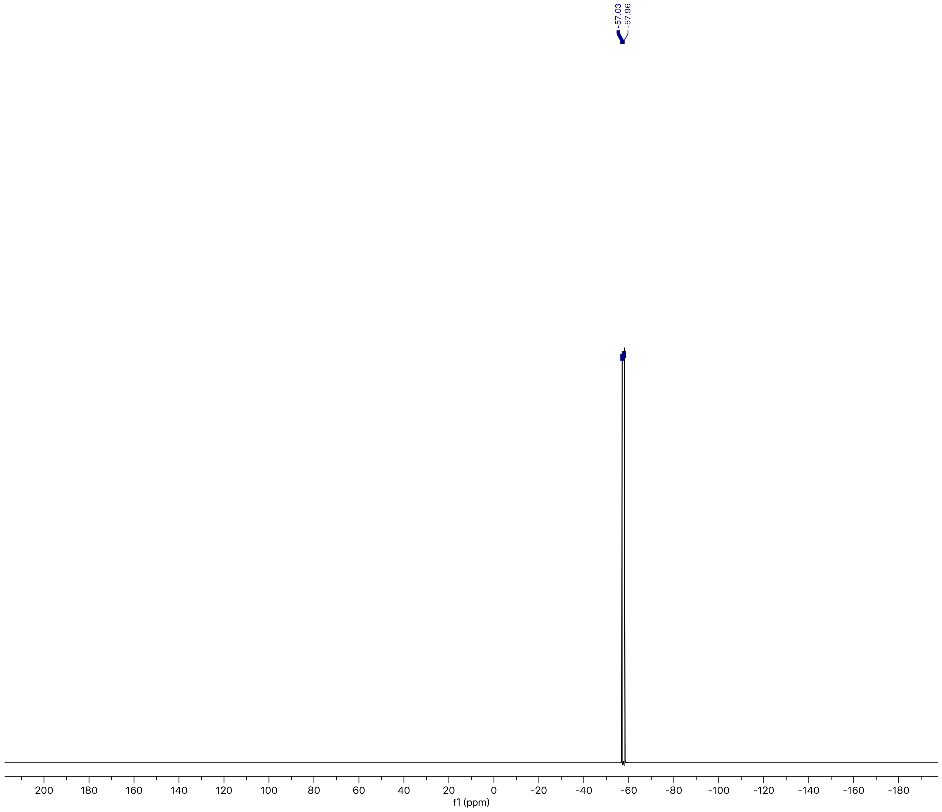

Current Data Parameters
NAME YC-GIBH-122
EXPNO 14
PROCNO 1

F2 - Acquisition Parameters
Date_ 20190615
Time 3.08 h
INSTRUM AvanceNeo
PROBHD Z116098_0793 (
PULPROG zgig30
TD 261948
SOLVENT CDCl3
NS 16
DS 0
SWH 156250.000 Hz
FIDRES 1.192985 Hz
AQ 0.8382336 sec
RG 101
DW 3.200 usec
DE 6.82 usec
TE 298.0 K
D1 2.00000000 sec
D11 0.03000000 sec
TD0 1
SFO1 376.5021312 MHz
NUC1 19F
P0 6.00 usec
P1 18.00 usec
PLW1 18.94099998 W
SFO2 400.1318006 MHz
NUC2 1H
CPDPRG[2 waltz16
PCPD2 90.00 usec
PLW2 18.69700050 W
PLW12 0.23083000 W

F2 - Processing parameters
SI 262144
SF 376.4983662 MHz
WDW EM
SSB 0
LB 1.00 Hz
GB 0
PC 2.00

BGAz-006

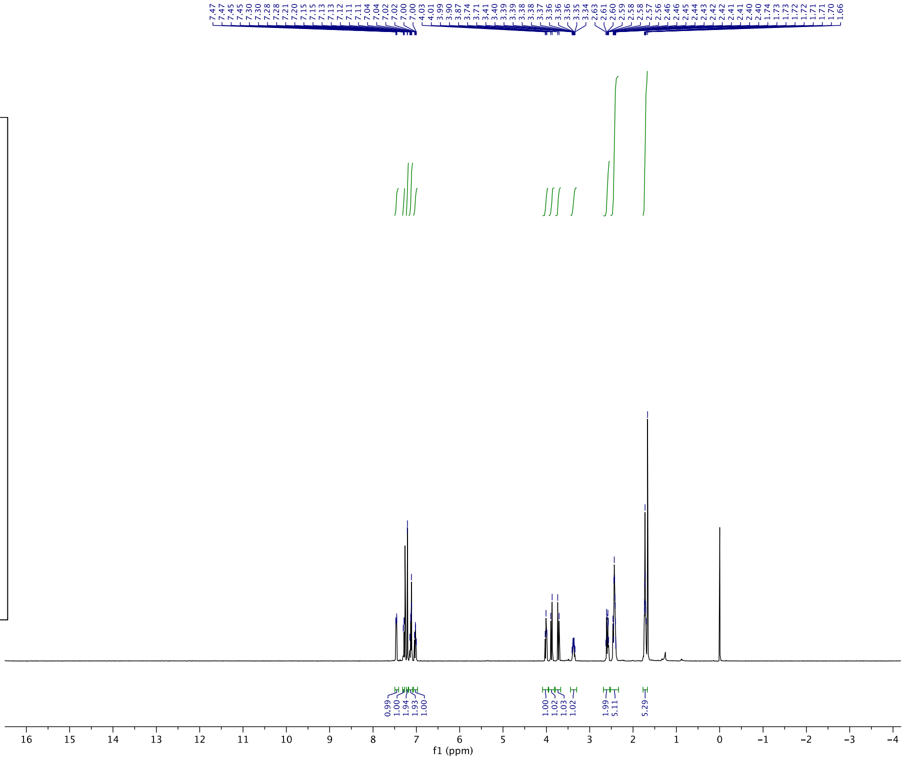

NAME May04-2017
EXPNO 31
PROCNO 1
Date_ 20170504
Time 15.58
INSTRUM spect
PROBHD 5 mm DUL 13C-1
PULPROG zg30
TD 65536
SOLVENT CDCl3
NS 16
DS 2
SWH 8278.146 Hz
FIDRES 0.126314 Hz
AQ 3.9584243 sec
RG 256
DW 60.400 usec
DE 6.50 usec
TE 298.6 K
D1 1.00000000 sec
TD0 1

======== CHANNEL f1 ========
NUC1 1H
P1 12.58 usec
PL1 0.00 dB
PL1W 10.87646866 W
SFO1 400.1324710 MHz
SI 32768
SF 400.1300082 MHz
WDW EM
SSB 0
LB 0.30 Hz
GB 0
PC 1.00

BGAz-006

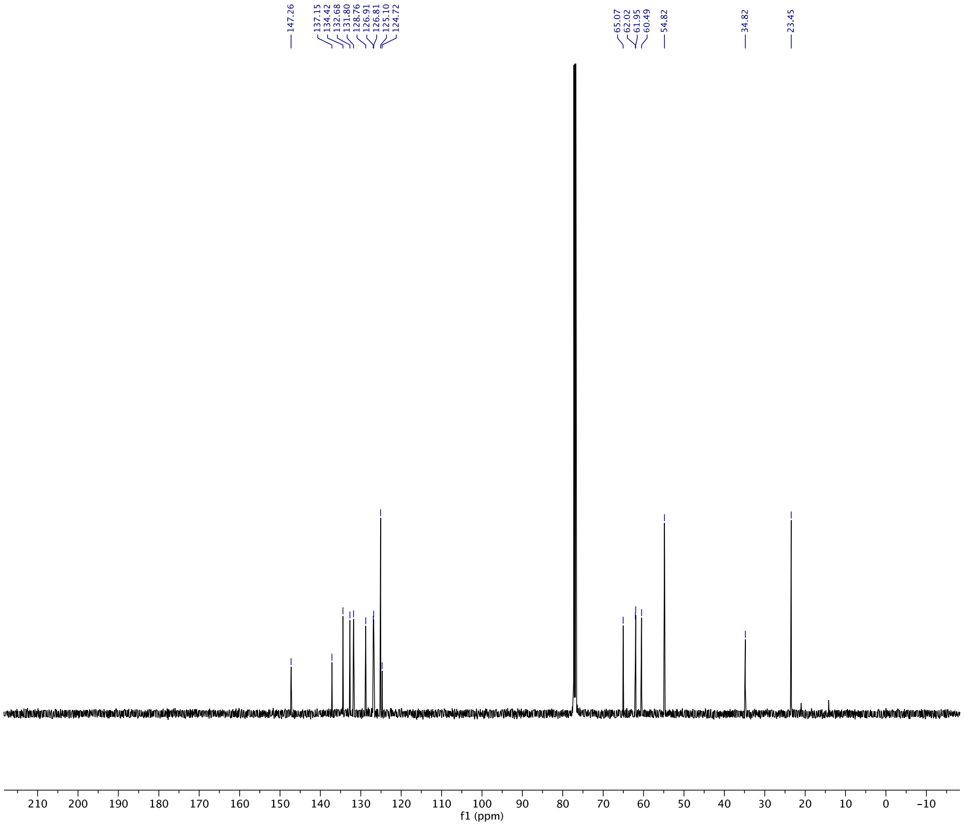

NAME May08-2017
EXPNO 2
PROCNO 1
Date_ 20170508
Time 15.14
INSTRUM Spect
PROBHD 5 mm PABBO BB-
PULPROG zgpg30
TD 65536
SOLVENT CDCl3
NS 1024
DS 4
SWH 29761.904 Hz
FIDRES 0.454131 Hz
AQ 1.1010548 sec
RG 203
DW 16.800 usec
DE 6.50 usec
TE 302.6 K
D1 2.00000000 sec
D11 0.03000000 sec
TD0 1

======== CHANNEL f1
========
NUC1 13C
P1 8.80 usec
PL1 -1.00 dB
PL1W 104.98761749 W
SFO1 125.7703643 MHz

======== CHANNEL f2
========
CPDPRG2 waltz16
NUC2 1H
PCPD2 80.00 usec
PL2 2.50 dB
PL12 17.46 dB
PL13 17.46 dB
PL2W 13.02359581 W
PL12W 0.41565308 W
PL13W 0.41565308 W
SFO2 500.1320005 MHz
SI 32768
SF 125.7577894 MHz
WDW EM
SSB 0
LB 1.00 Hz
GB 0
PC 1.40

BGAz-007

```
NAME Jun06-2017
EXPNO 9
PROCNO 1
Date_ 20170606
Time 16.00
INSTRUM Spect
PROBHD 5 mm PABBO BB-
PULPROG zg30
TD 65536
SOLVENT CDCl3
NS 16
DS 2
SWH 10330.578 Hz
FIDRES 0.157632 Hz
AQ 3.1719923 sec
RG 57
DW 48.400 usec
DE 6.50 usec
TE 302.2 K
D1 1.00000000 sec
TD0 1

======== CHANNEL f1 ========
NUC1 1H
P1 14.00 usec
PL1 2.50 dB
PL1W 13.02359581 W
SFO1 500.1330885 MHz
SI 32768
SF 500.1300154 MHz
WDW EM
SSB 0
LB 0.30 Hz
GB 0
PC 1.00
```

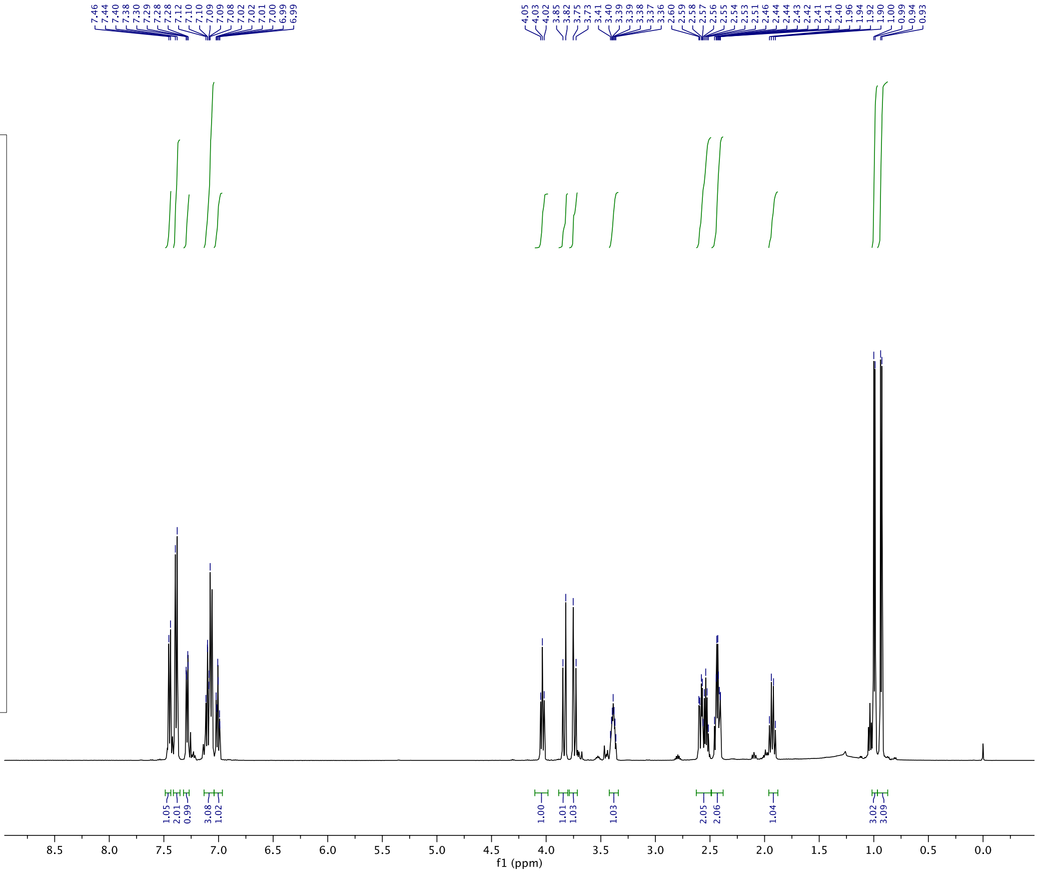

BGAz-007

```
NAME Jun06-2017
EXPNO 11
PROCNO 1
Date_ 20170606
Time 18.47
INSTRUM Spect
PROBHD 5 mm PABBO BB-
PULPROG zgpg30
TD 65536
SOLVENT CDCl3
NS 3072
DS 4
SWH 29761.904 Hz
FIDRES 0.454131 Hz
AQ 1.1010548 sec
RG 203
DW 16.800 usec
DE 6.50 usec
TE 304.8 K
D1 2.00000000 sec
D11 0.03000000 sec
TD0 1

======== CHANNEL f1
========
NUC1 13C
P1 8.80 usec
PL1 -1.00 dB
PL1W 104.98761749 W
SFO1 125.7703643 MHz

======== CHANNEL f2
========
CPDPRG2 waltz16
NUC2 1H
PCPD2 80.00 usec
PL2 2.50 dB
PL12 17.46 dB
PL13 17.46 dB
PL2W 13.02359581 W
PL12W 0.41565308 W
PL13W 0.41565308 W
SFO2 500.1320005 MHz
SI 32768
SF 125.7577890 MHz
WDW EM
SSB 0
LB 1.00 Hz
GB 0
PC 1.40
```

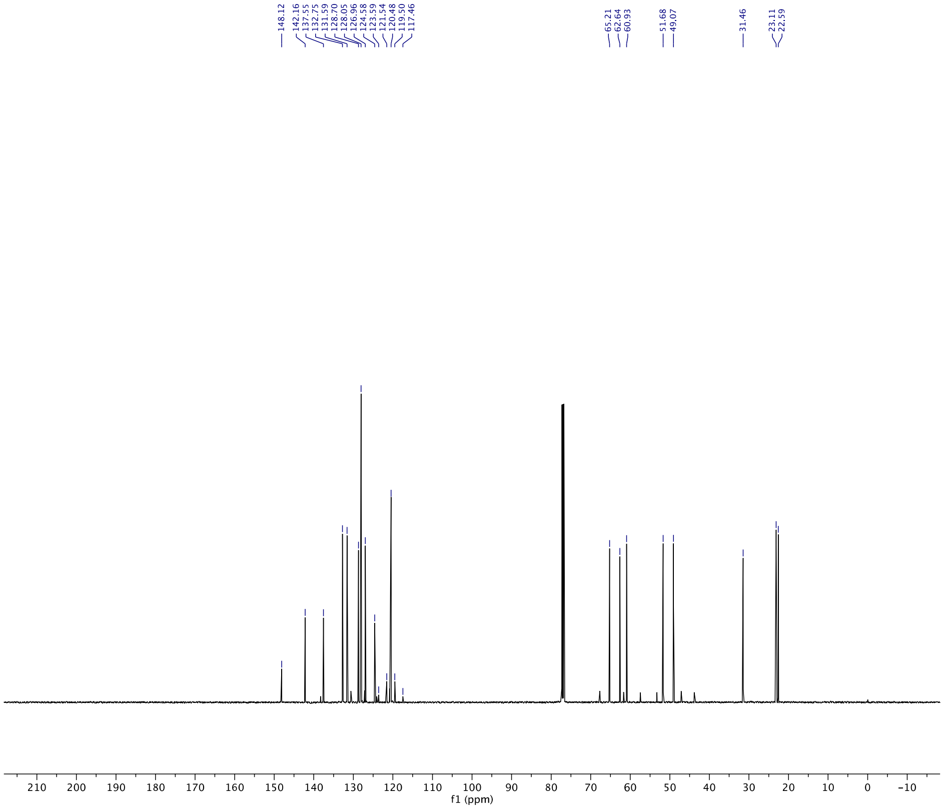

BGAz-007

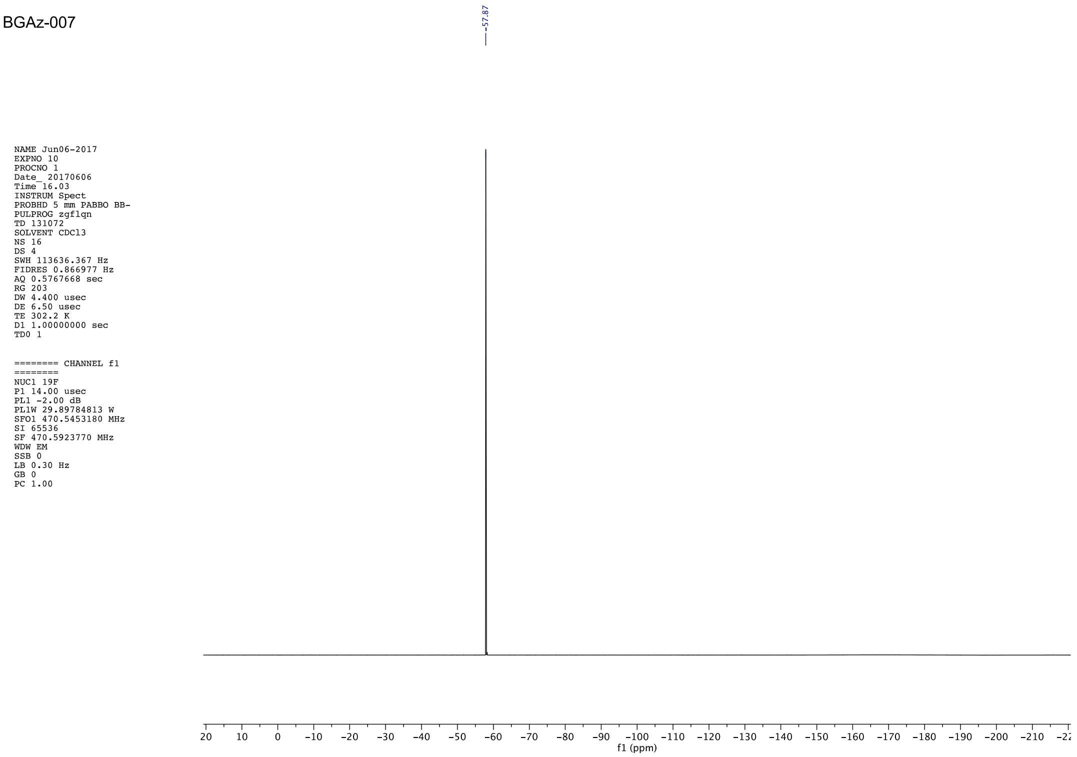

BGAz-008

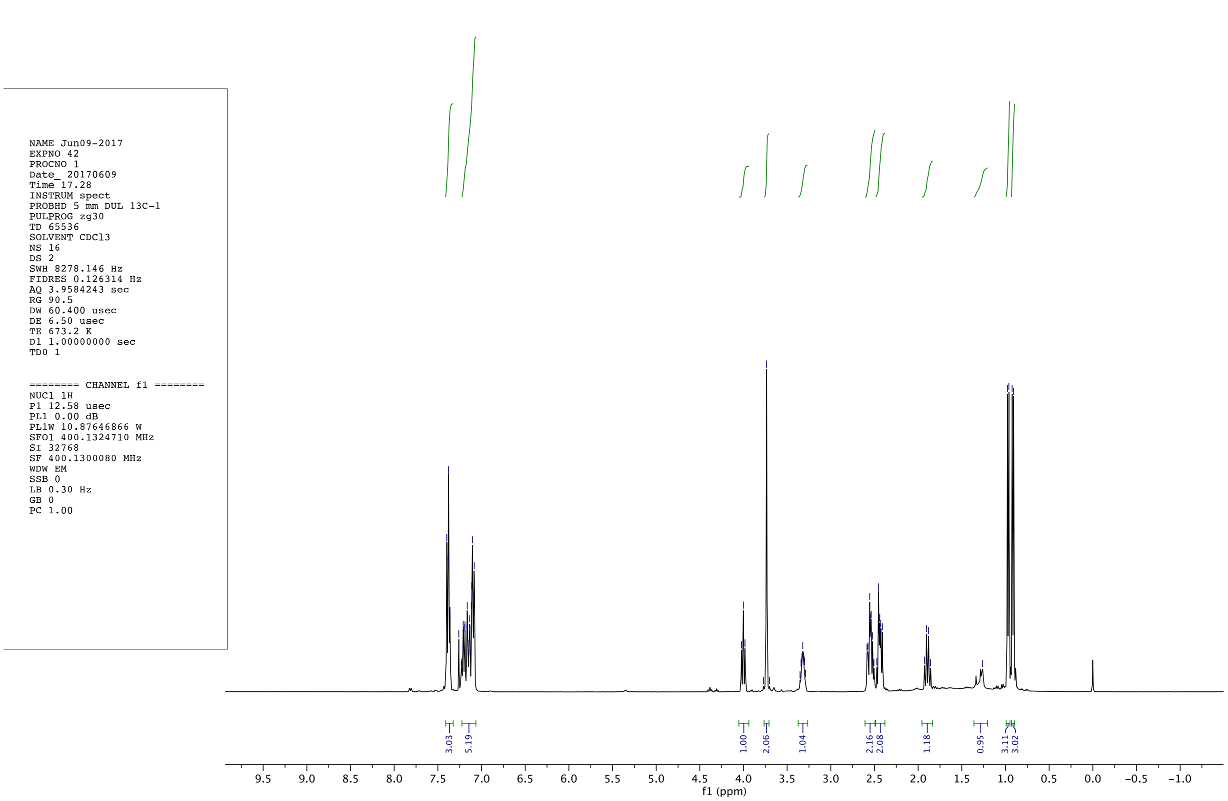

BGAz-008

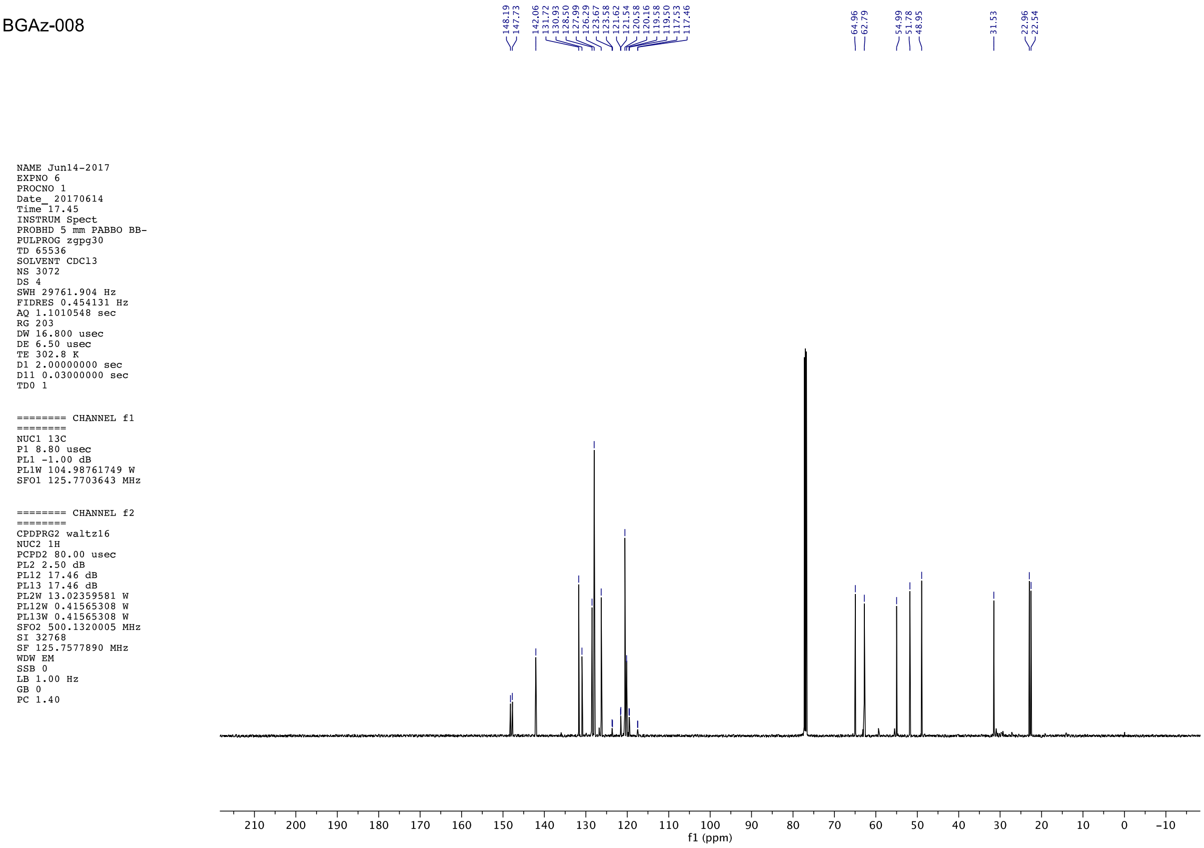

BGAz-008

δ F (471 MHz, $CDCl_3$) -56.98, -57.94.

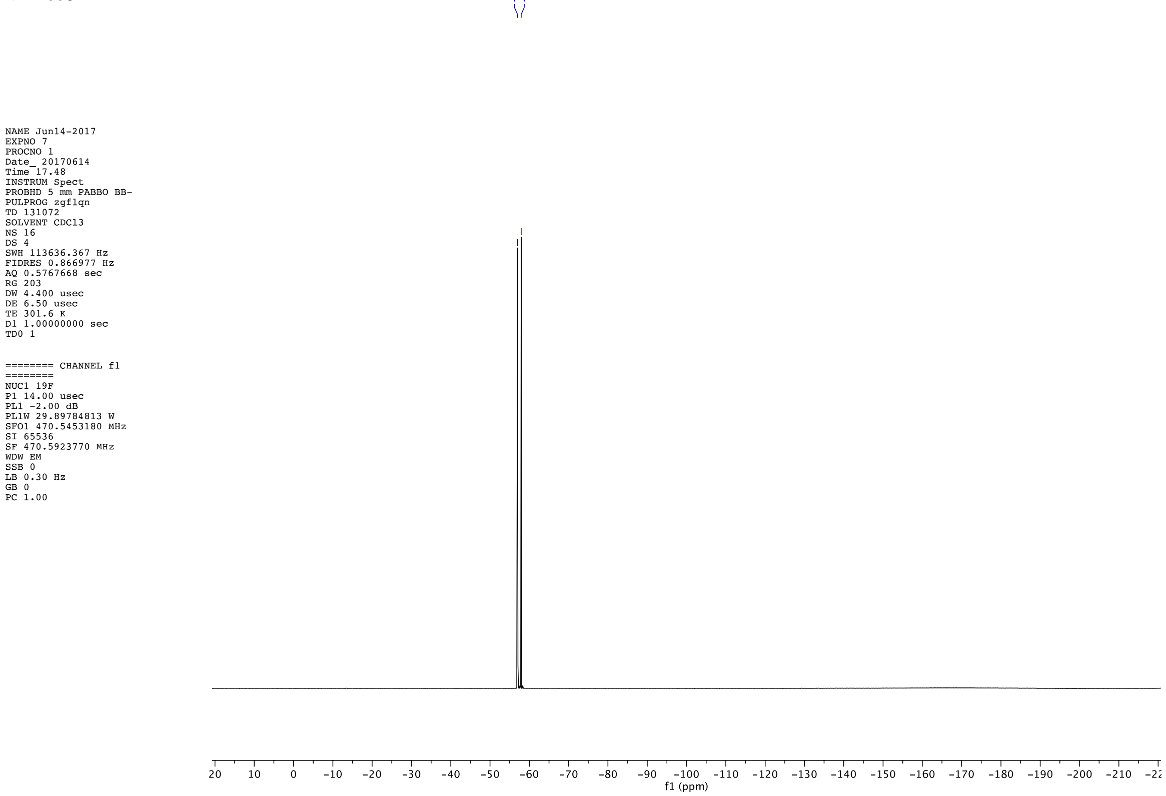

BGAz-009

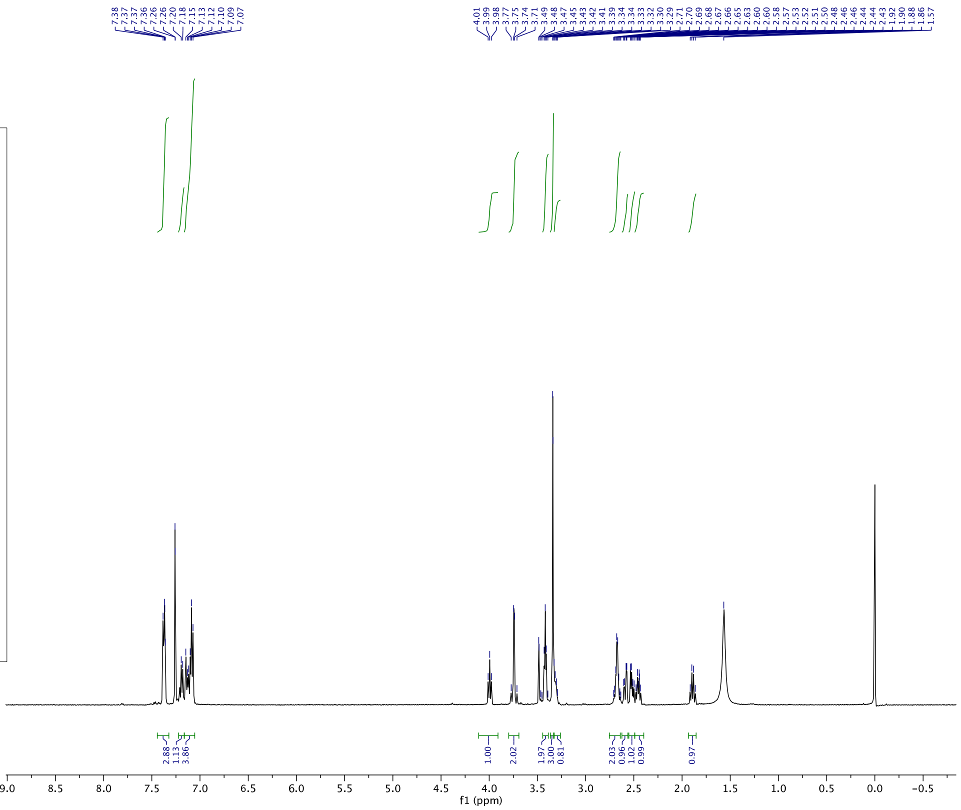

NAME Aug03-2017
EXPNO 8
PROCNO 1
Date_ 20170803
Time 13.07
INSTRUM Spect
PROBHD 5 mm PABBO BB-
PULPROG zg30
TD 65536
SOLVENT CDCl3
NS 16
DS 2
SWH 10330.578 Hz
FIDRES 0.157632 Hz
AQ 3.1719923 sec
RG 203
DW 48.400 usec
DE 6.50 usec
TE 301.9 K
D1 1.00000000 sec
TD0 1

======== CHANNEL f1 ========
NUC1 1H
P1 14.00 usec
PL1 2.50 dB
PL1W 13.02359581 W
SFO1 500.1330885 MHz
SI 32768
SF 500.1300140 MHz
WDW EM
SSB 0
LB 0.30 Hz
GB 0
PC 1.00

BGAz-009

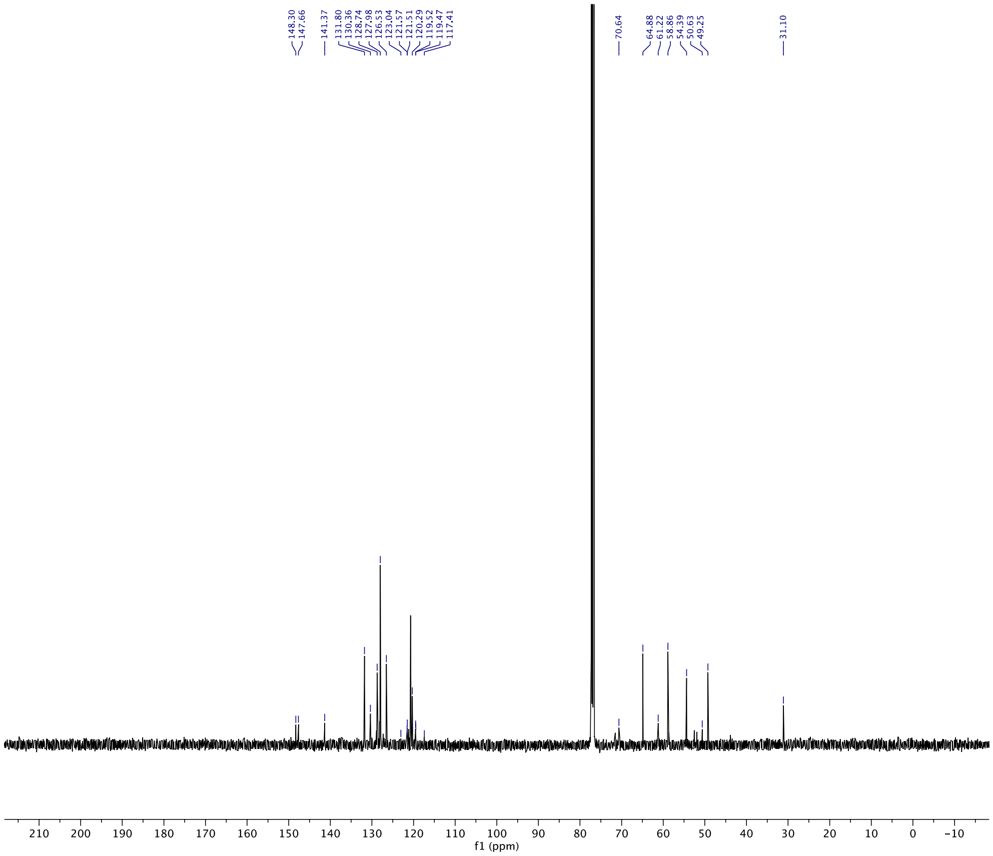

Current Data Parameters
NAME Jul26-2019
EXPNO 20
PROCNO 1

F2 - Acquisition Parameters
Date_ 20190726
Time 23.12 h
INSTRUM Spect
PROBHD Z109128_0101 (
PULPROG zgpg30
TD 65536
SOLVENT CDCl3
NS 4096
DS 4
SWH 29761.904 Hz
FIDRES 0.908261 Hz
AQ 1.1010048 sec
RG 203
DW 16.800 usec
DE 6.50 usec
TE 300.2 K
D1 2.00000000 sec
D11 0.03000000 sec
TD0 1
SFO1 125.7703643 MHz
NUC1 13C
P1 13.50 usec
PLW1 76.00000000 W
SFO2 500.1320005 MHz
NUC2 1H
CPDPRG[2 waltz16
PCPD2 80.00 usec
PLW2 18.00000000 W
PLW12 0.43945000 W
PLW13 0.22104000 W

F2 - Processing parameters
SI 32768
SF 125.7577899 MHz
WDW EM
SSB 0
LB 1.00 Hz
GB 0
PC 1.40

BGAz-009

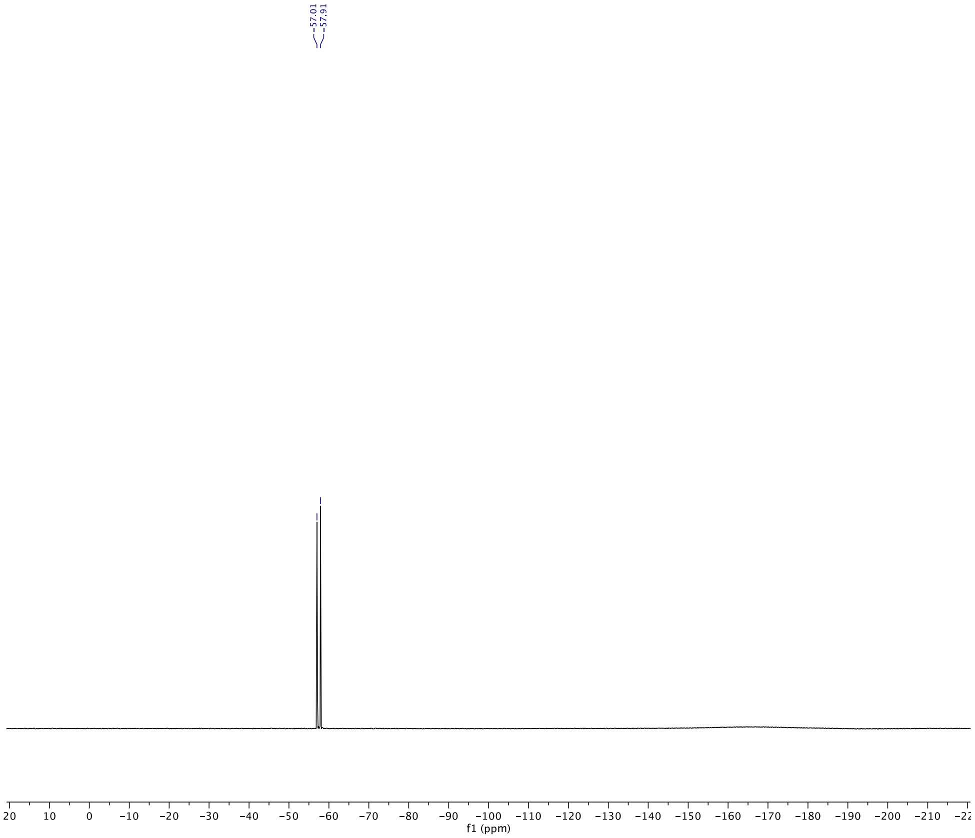

Current Data Parameters
NAME Jul25-2019
EXPNO 3
PROCNO 1

F2 - Acquisition Parameters
Date_ 20190725
Time 17.35 h
INSTRUM Spect
PROBHD Z109128_0101 (
PULPROG zgflqn
TD 131072
SOLVENT CDCl3
NS 16
DS 4
SWH 113636.367 Hz
FIDRES 1.733953 Hz
AQ 0.5767168 sec
RG 203
DW 4.400 usec
DE 6.50 usec
TE 298.5 K
D1 1.00000000 sec
TD0 1
SFO1 470.5453180 MHz
NUC1 19F
P1 18.00 usec
PLW1 20.00000000 W

F2 - Processing parameters
SI 65536
SF 470.5923770 MHz
WDW EM
SSB 0
LB 0.30 Hz
GB 0
PC 1.00

BGAz-010

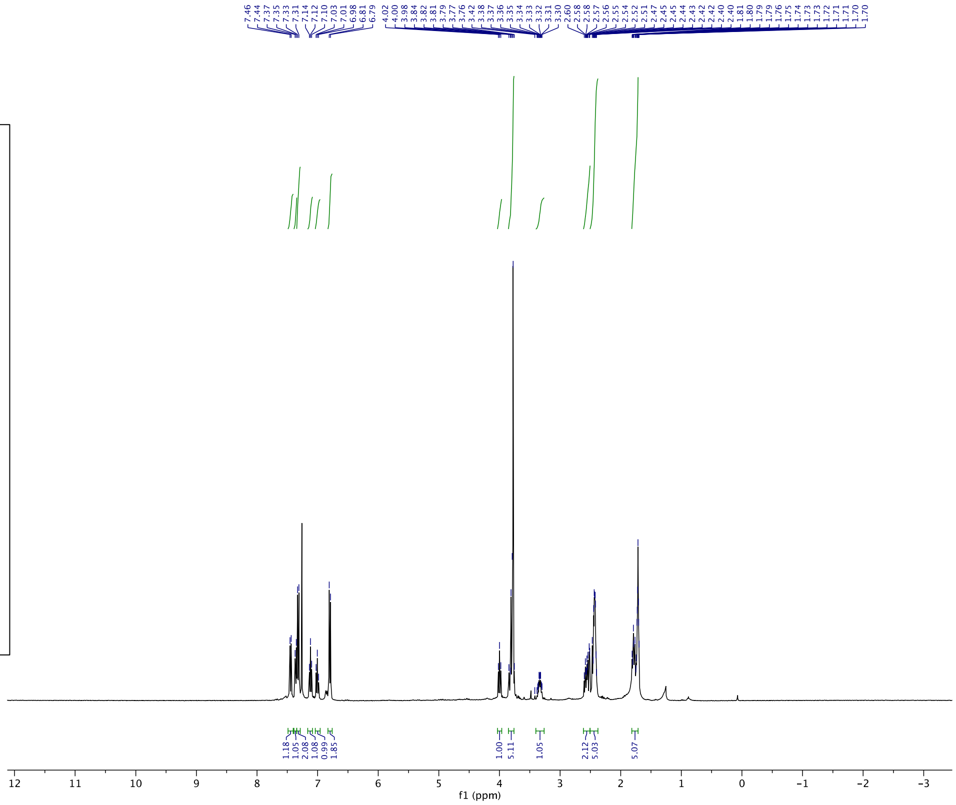

NAME Dec05-2016
EXPNO 49
PROCNO 1
Date_ 20161205
Time 18.14
INSTRUM spect
PROBHD 5 mm DUL 13C-1
PULPROG zg30
TD 65536
SOLVENT CDCl3
NS 16
DS 2
SWH 8278.146 Hz
FIDRES 0.126314 Hz
AQ 3.9584243 sec
RG 256
DW 60.400 usec
DE 6.50 usec
TE 296.6 K
D1 1.00000000 sec
TD0 1

======== CHANNEL f1 ========
NUC1 1H
P1 12.58 usec
PL1 0.00 dB
PL1W 10.87646866 W
SFO1 400.1324710 MHz
SI 32768
SF 400.1300093 MHz
WDW EM
SSB 0
LB 0.30 Hz
GB 0
PC 1.00

BGAz-010

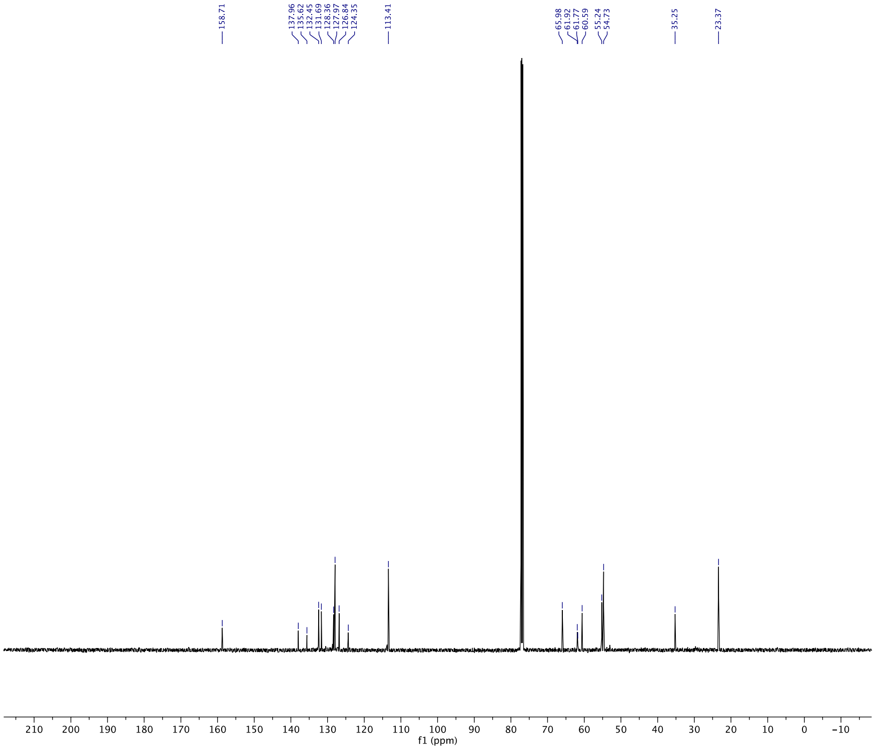

NAME Dec06-2016
EXPNO 16
PROCNO 1
Date_ 20161208
Time 3.11
INSTRUM Spect
PROBHD 5 mm PABBO BB-
PULPROG zgpg30
TD 65536
SOLVENT CDCl3
NS 2048
DS 4
SWH 29761.904 Hz
FIDRES 0.454131 Hz
AQ 1.1010548 sec
RG 203
DW 16.800 usec
DE 6.50 usec
TE 298.0 K
D1 2.00000000 sec
D11 0.03000000 sec
TD0 1

======== CHANNEL f1 ========
NUC1 13C
P1 13.84 usec
PL1 2.50 dB
PL1W 46.89624786 W
SFO1 125.7703643 MHz

======== CHANNEL f2 ========
CPDPRG2 waltz16
NUC2 1H
PCPD2 80.00 usec
PL2 2.50 dB
PL12 17.40 dB
PL13 17.40 dB
PL2W 13.02359581 W
PL12W 0.42143536 W
PL13W 0.42143536 W
SFO2 500.1320005 MHz
SI 32768
SF 125.7577940 MHz
WDW EM
SSB 0
LB 1.00 Hz
GB 0
PC 1.40

BGAz-011

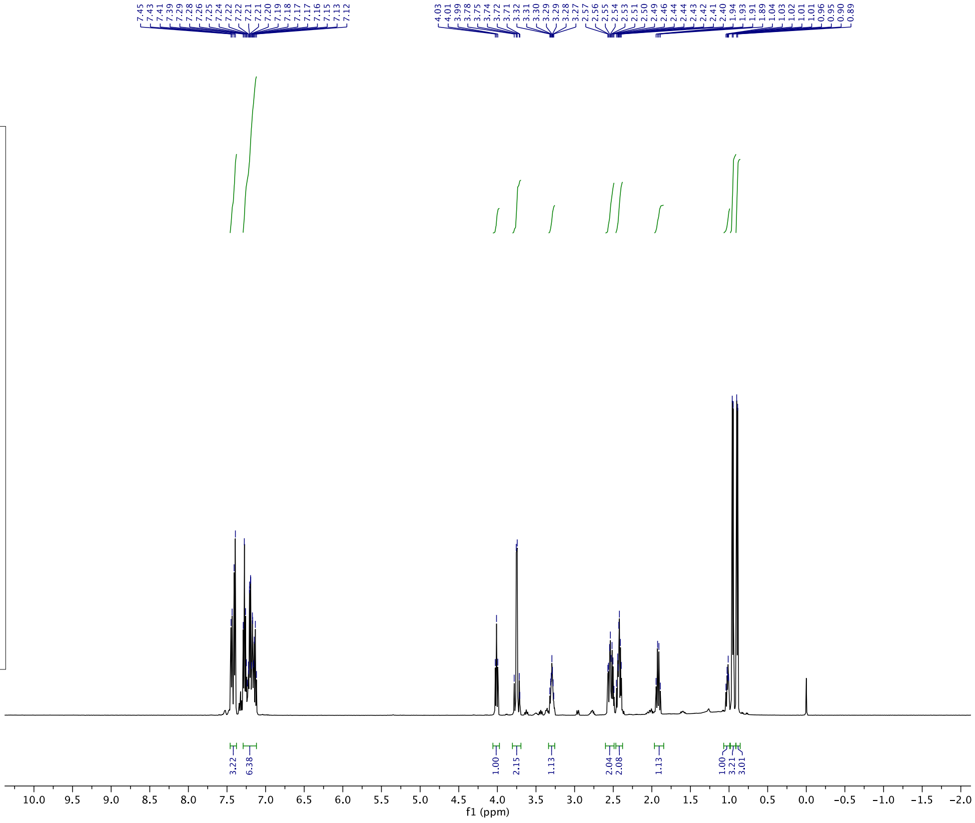

NAME May31-2017
EXPNO 15
PROCNO 1
Date_ 20170531
Time 21.21
INSTRUM Spect
PROBHD 5 mm PABBO BB-
PULPROG zg30
TD 65536
SOLVENT CDCl3
NS 16
DS 2
SWH 10330.578 Hz
FIDRES 0.157632 Hz
AQ 3.1719923 sec
RG 80.6
DW 48.400 usec
DE 6.50 usec
TE 301.9 K
D1 1.00000000 sec
TD0 1

======== CHANNEL f1 ========
NUC1 1H
P1 14.00 usec
PL1 2.50 dB
PL1W 13.02359581 W
SFO1 500.1330885 MHz
SI 32768
SF 500.1300192 MHz
WDW EM
SSB 0
LB 0.30 Hz
GB 0
PC 1.00

BGAz-011

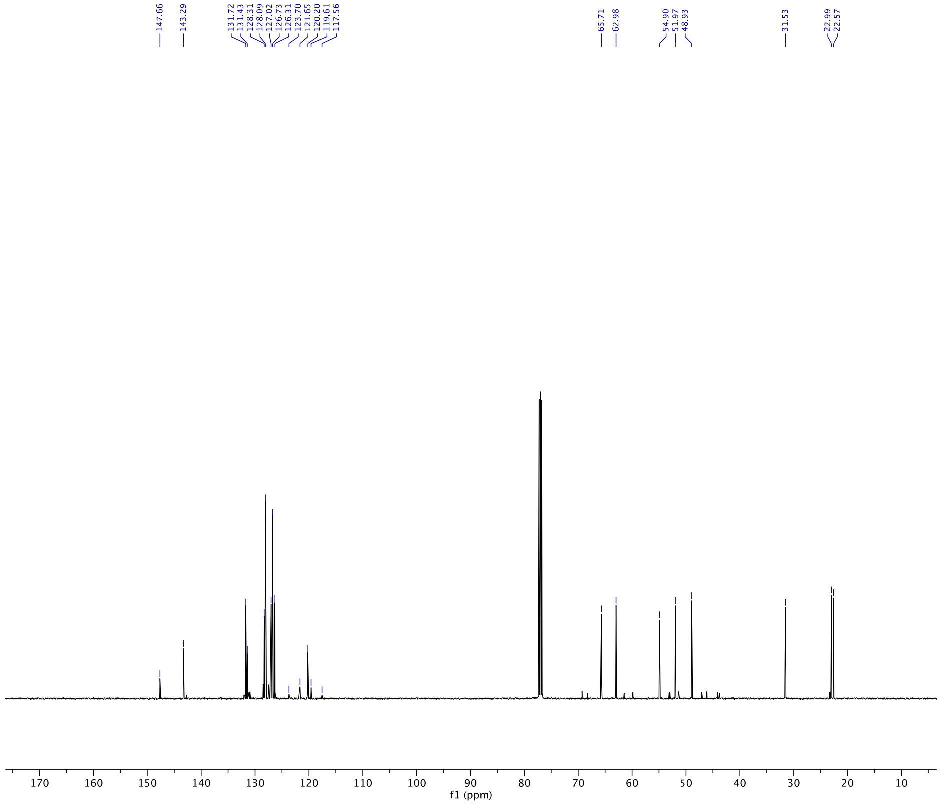

NAME May31-2017
EXPNO 17
PROCNO 1
Date_ 20170601
Time 0.08
INSTRUM Spect
PROBHD 5 mm PABBO BB-
PULPROG zgpg30
TD 65536
SOLVENT CDCl3
NS 3072
DS 4
SWH 29761.904 Hz
FIDRES 0.454131 Hz
AQ 1.1010548 sec
RG 203
DW 16.800 usec
DE 6.50 usec
TE 303.4 K
D1 2.00000000 sec
D11 0.03000000 sec
TD0 1

======== CHANNEL f1
========
NUC1 13C
P1 8.80 usec
PL1 -1.00 dB
PL1W 104.98761749 W
SFO1 125.7703643 MHz

======== CHANNEL f2
========
CPDPRG2 waltz16
NUC2 1H
PCPD2 80.00 usec
PL2 2.50 dB
PL12 17.46 dB
PL13 17.46 dB
PL2W 13.02359581 W
PL12W 0.41565308 W
PL13W 0.41565308 W
SFO2 500.1320005 MHz
SI 32768
SF 125.7577898 MHz
WDW EM
SSB 0
LB 1.00 Hz
GB 0
PC 1.40

BGAz-011

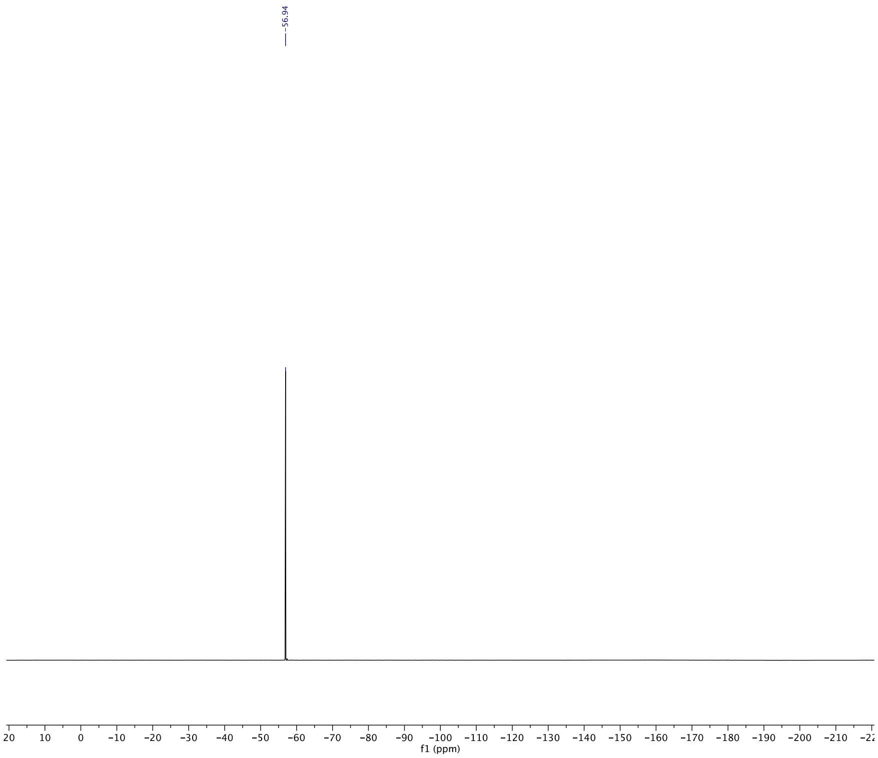

NAME May31-2017
EXPNO 16
PROCNO 1
Date_ 20170531
Time 21.23
INSTRUM Spect
PROBHD 5 mm PABBO BB-
PULPROG zgflqn
TD 131072
SOLVENT CDCl3
NS 16
DS 4
SWH 113636.367 Hz
FIDRES 0.866977 Hz
AQ 0.5767668 sec
RG 203
DW 4.400 usec
DE 6.50 usec
TE 301.8 K
D1 1.00000000 sec
TD0 1

======== CHANNEL f1
========
NUC1 19F
P1 14.00 usec
PL1 -2.00 dB
PL1W 29.89784813 W
SFO1 470.5453180 MHz
SI 65536
SF 470.5923770 MHz
WDW EM
SSB 0
LB 0.30 Hz
GB 0
PC 1.00

BGAz-012

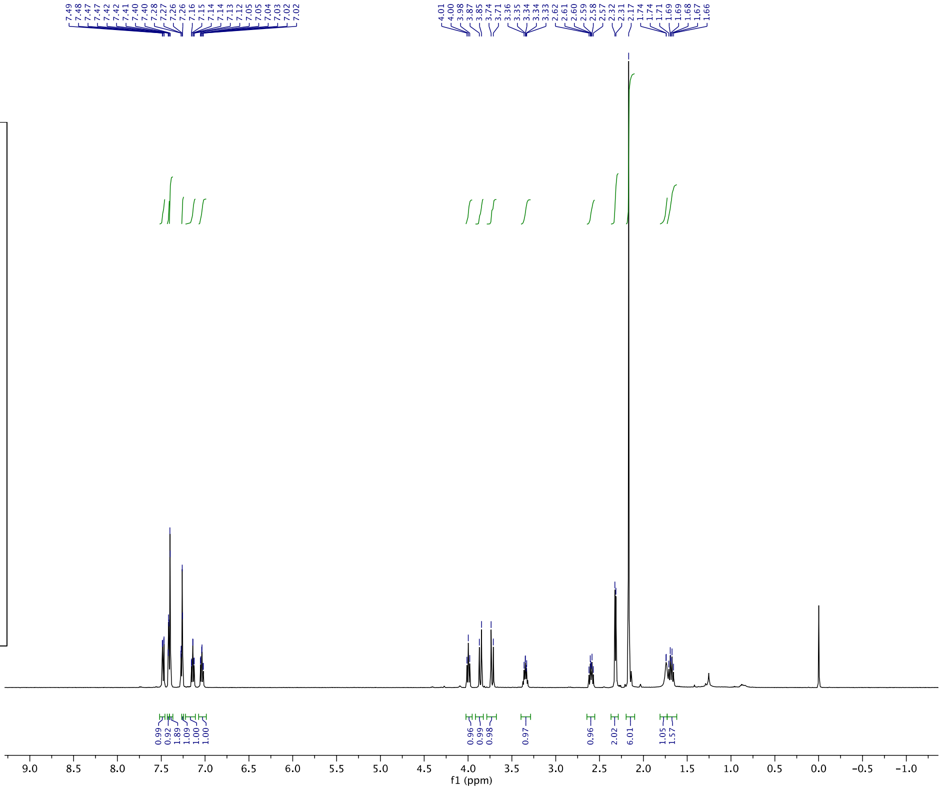

NAME Mar27-2018
EXPNO 6
PROCNO 1
Date_ 20180327
Time 16.32
INSTRUM Spect
PROBHD 5 mm PABBO BB-
PULPROG zg30
TD 65536
SOLVENT CDCl3
NS 16
DS 2
SWH 10330.578 Hz
FIDRES 0.157632 Hz
AQ 3.1719923 sec
RG 203
DW 48.400 usec
DE 6.50 usec
TE 296.2 K
D1 1.00000000 sec
TD0 1

======== CHANNEL f1 ========
NUC1 1H
P1 14.00 usec
PL1 2.50 dB
PL1W 13.02359581 W
SFO1 500.1330885 MHz
SI 32768
SF 500.1300132 MHz
WDW EM
SSB 0
LB 0.30 Hz
GB 0
PC 1.00

BGAz-012

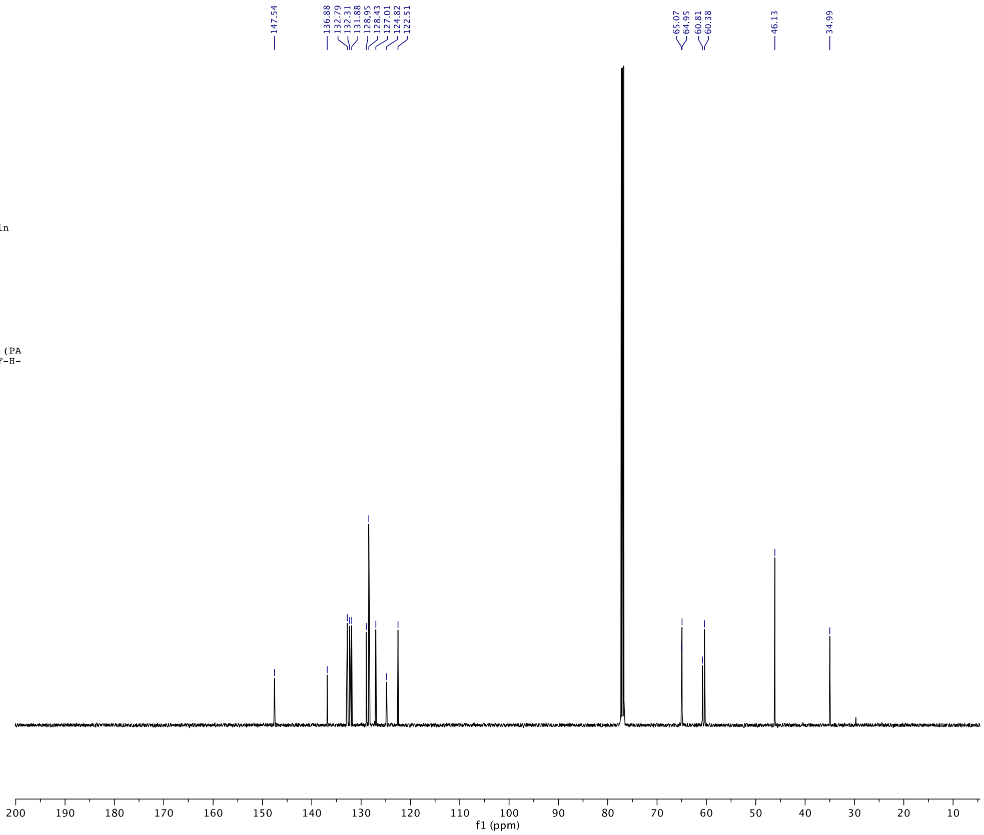

Origin	Bruker BioSpin GmbH
Owner	500MHz
Instrument	Spect
Solvent	CDCl3
Temperature	300.6
Pulse Sequence	zgpg30
Experiment	1D
Probe	Z109128_0101 (PA BBO 500S2 BBF-H-D-05 Z)
Number of Scans	2048
Receiver Gain	203.0
Relaxation Delay	2.0000
Pulse Width	13.5000
Presaturation Frequency	
Acquisition Time	1.1010
Class	
Spectrometer Frequency	125.77
Spectral Width	29761.9
Lowest Frequency	-2305.6
Nucleus	13C
Acquired Size	32768
Spectral Size	65536

NAME May25-2017
EXPNO 2
PROCNO 1
Date_ 20170525
Time 17.56
INSTRUM Spect
PROBHD 5 mm PABBO BB-
PULPROG zgpg30
TD 65536
SOLVENT CDCl3
NS 2048
DS 4
SWH 29761.904 Hz
FIDRES 0.454131 Hz
AQ 1.1010548 sec
RG 203
DW 16.800 usec
DE 6.50 usec
TE 302.4 K
D1 2.00000000 sec
D11 0.03000000 sec
TD0 1

======== CHANNEL f1 ========
NUC1 13C
P1 8.80 usec
PL1 -1.00 dB
PL1W 104.98761749 W
SFO1 125.7703643 MHz

======== CHANNEL f2 ========
CPDPRG2 waltz16
NUC2 1H
PCPD2 80.00 usec
PL2 2.50 dB
PL12 17.46 dB
PL13 17.46 dB
PL2W 13.02359581 W
PL12W 0.41565308 W
PL13W 0.41565308 W
SFO2 500.1320005 MHz
SI 32768
SF 125.7577919 MHz
WDW EM
SSB 0
LB 1.00 Hz
GB 0
PC 1.40

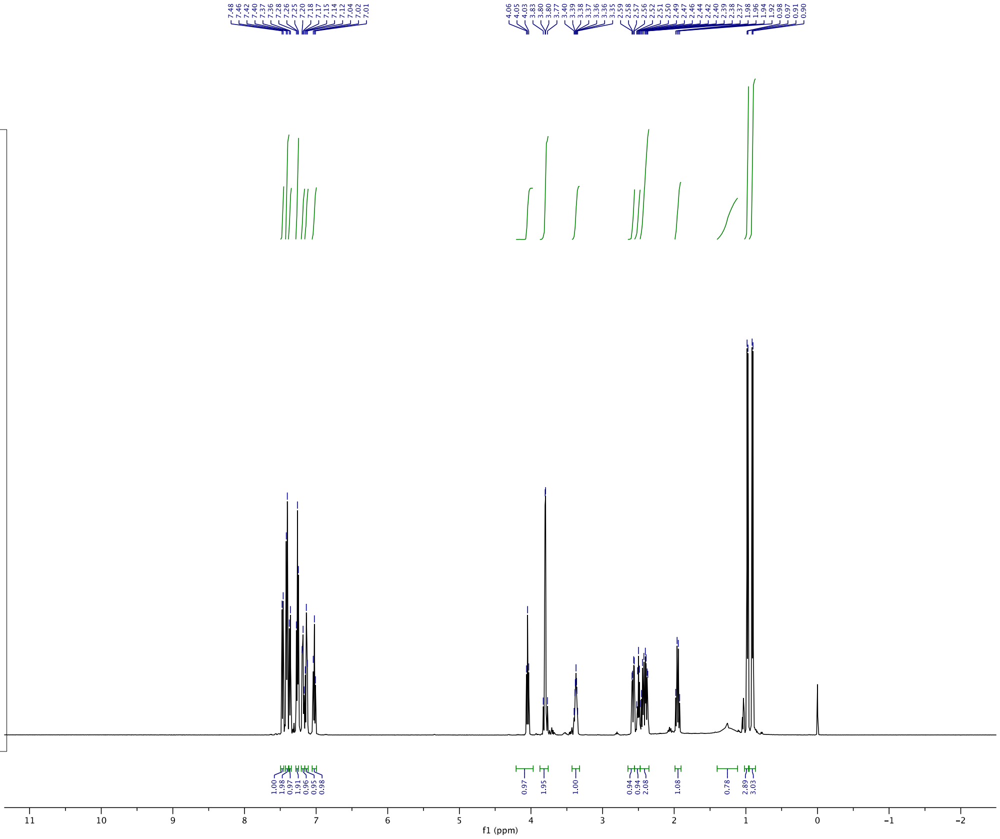

BGAz-013

NAME May25-2017
EXPNO 2
PROCNO 1
Date_ 20170525
Time 17.56
INSTRUM Spect
PROBHD 5 mm PABBO BB-
PULPROG zgpg30
TD 65536
SOLVENT CDCl3
NS 2048
DS 4
SWH 29761.904 Hz
FIDRES 0.454131 Hz
AQ 1.1010548 sec
RG 203
DW 16.800 usec
DE 6.50 usec
TE 302.4 K
D1 2.00000000 sec
D11 0.03000000 sec
TD0 1

======== CHANNEL f1
========
NUC1 13C
P1 8.80 usec
PL1 -1.00 dB
PL1W 104.98761749 W
SFO1 125.7703643 MHz

======== CHANNEL f2
========
CPDPRG2 waltz16
NUC2 1H
PCPD2 80.00 usec
PL2 2.50 dB
PL12 17.46 dB
PL13 17.46 dB
PL2W 13.02359581 W
PL12W 0.41565308 W
PL13W 0.41565308 W
SFO2 500.1320005 MHz
SI 32768
SF 125.7577919 MHz
WDW EM
SSB 0
LB 1.00 Hz
GB 0
PC 1.40

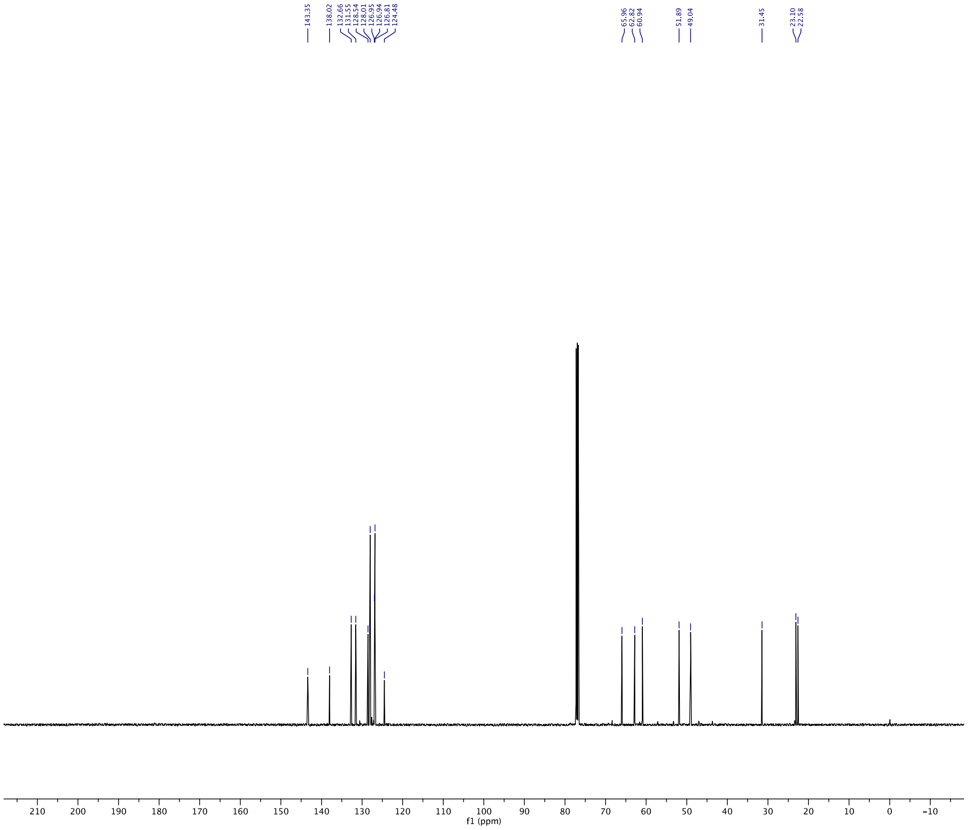

BGAz-014

NAME May25-2017
EXPNO 2
PROCNO 1
Date_ 20170525
Time 17.56
INSTRUM Spect
PROBHD 5 mm PABBO BB-
PULPROG zgpg30
TD 65536
SOLVENT CDCl3
NS 2048
DS 4
SWH 29761.904 Hz
FIDRES 0.454131 Hz
AQ 1.1010548 sec
RG 203
DW 16.800 usec
DE 6.50 usec
TE 302.4 K
D1 2.00000000 sec
D11 0.03000000 sec
TD0 1

======== CHANNEL f1 ========
NUC1 13C
P1 8.80 usec
PL1 -1.00 dB
PL1W 104.98761749 W
SFO1 125.7703643 MHz

======== CHANNEL f2 ========
CPDPRG2 waltz16
NUC2 1H
PCPD2 80.00 usec
PL2 2.50 dB
PL12 17.46 dB
PL13 17.46 dB
PL2W 13.02359581 W
PL12W 0.41565308 W
PL13W 0.41565308 W
SFO2 500.1320005 MHz
SI 32768
SF 125.7577919 MHz
WDW EM
SSB 0
LB 1.00 Hz
GB 0
PC 1.40

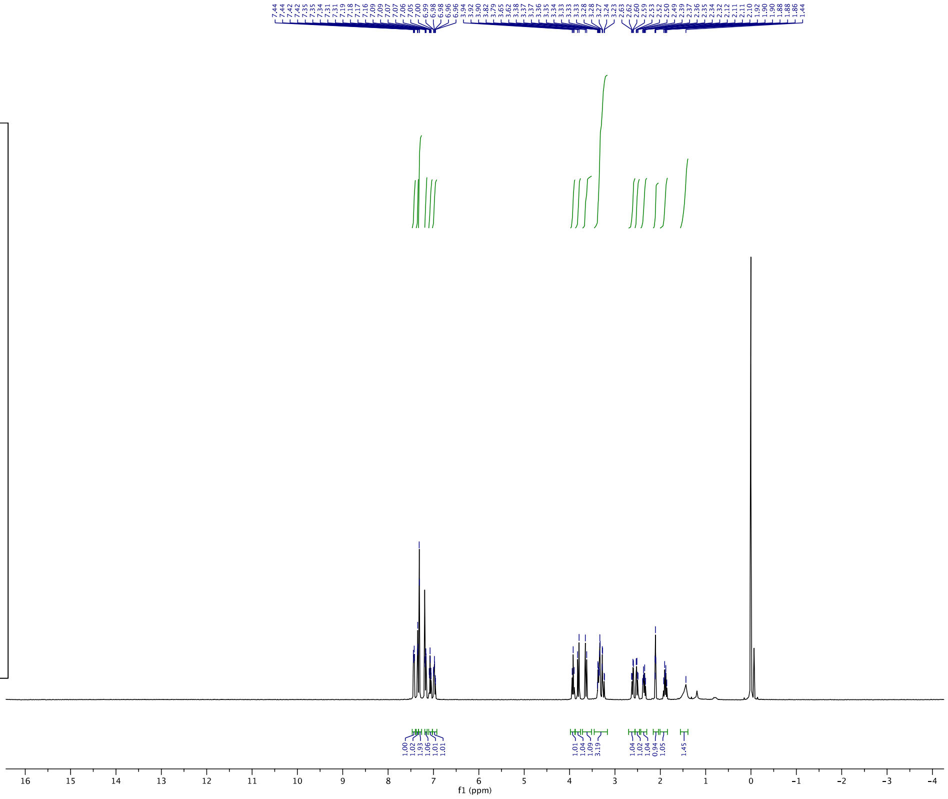

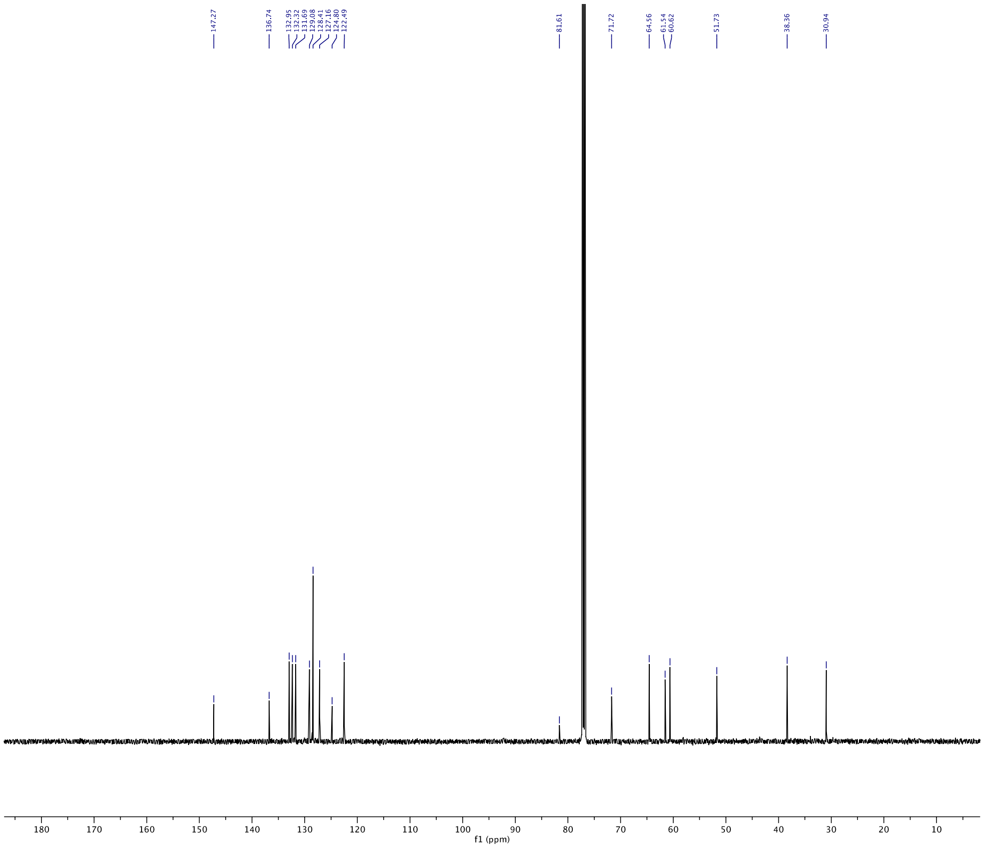

NAME May25-2017
EXPNO 2
PROCNO 1
Date_ 20170525
Time 17.56
INSTRUM Spect
PROBHD 5 mm PABBO BB-
PULPROG zgpg30
TD 65536
SOLVENT CDCl3
NS 2048
DS 4
SWH 29761.904 Hz
FIDRES 0.454131 Hz
AQ 1.1010548 sec
RG 203
DW 16.800 usec
DE 6.50 usec
TE 302.4 K
D1 2.00000000 sec
D11 0.03000000 sec
TD0 1

======== CHANNEL f1 ========
NUC1 13C
P1 8.80 usec
PL1 -1.00 dB
PL1W 104.98761749 W
SFO1 125.7703643 MHz

======== CHANNEL f2 ========
CPDPRG2 waltz16
NUC2 1H
PCPD2 80.00 usec
PL2 2.50 dB
PL12 17.46 dB
PL13 17.46 dB
PL2W 13.02359581 W
PL12W 0.41565308 W
PL13W 0.41565308 W
SFO2 500.1320005 MHz
SI 32768
SF 125.7577919 MHz
WDW EM
SSB 0
LB 1.00 Hz
GB 0
PC 1.40

BGAz-015

```
NAME May25-2017
EXPNO 2
PROCNO 1
Date_ 20170525
Time 17.56
INSTRUM Spect
PROBHD 5 mm PABBO BB-
PULPROG zgpg30
TD 65536
SOLVENT CDCl3
NS 2048
DS 4
SWH 29761.904 Hz
FIDRES 0.454131 Hz
AQ 1.1010548 sec
RG 203
DW 16.800 usec
DE 6.50 usec
TE 302.4 K
D1 2.00000000 sec
D11 0.03000000 sec
TD0 1

======== CHANNEL f1 ========
NUC1 13C
P1 8.80 usec
PL1 -1.00 dB
PL1W 104.98761749 W
SFO1 125.7703643 MHz

======== CHANNEL f2 ========
CPDPRG2 waltz16
NUC2 1H
PCPD2 80.00 usec
PL2 2.50 dB
PL12 17.46 dB
PL13 17.46 dB
PL2W 13.02359581 W
PL12W 0.41565308 W
PL13W 0.41565308 W
SFO2 500.1320005 MHz
SI 32768
SF 125.7577919 MHz
WDW EM
SSB 0
LB 1.00 Hz
GB 0
PC 1.40
```

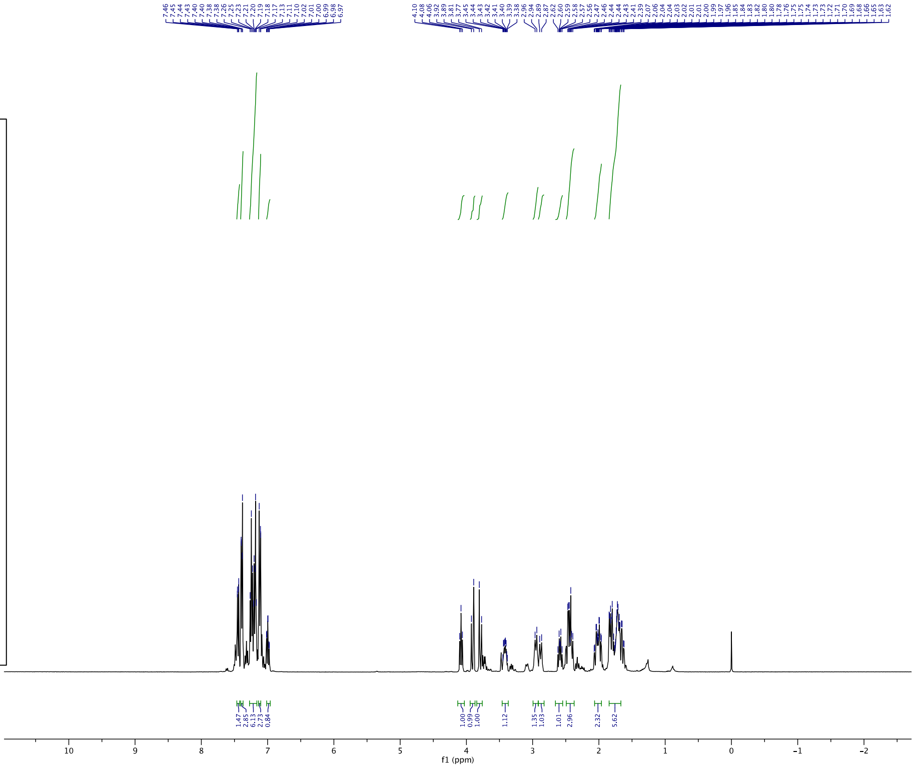

BGAz-015

```
NAME May25-2017
EXPNO 2
PROCNO 1
Date_ 20170525
Time 17.56
INSTRUM Spect
PROBHD 5 mm PABBO BB-
PULPROG zgpg30
TD 65536
SOLVENT CDCl3
NS 2048
DS 4
SWH 29761.904 Hz
FIDRES 0.454131 Hz
AQ 1.1010548 sec
RG 203
DW 16.800 usec
DE 6.50 usec
TE 302.4 K
D1 2.00000000 sec
D11 0.03000000 sec
TD0 1

======== CHANNEL f1
========
NUC1 13C
P1 8.80 usec
PL1 -1.00 dB
PL1W 104.98761749 W
SFO1 125.7703643 MHz

======== CHANNEL f2
========
CPDPRG2 waltz16
NUC2 1H
PCPD2 80.00 usec
PL2 2.50 dB
PL12 17.46 dB
PL13 17.46 dB
PL2W 13.02359581 W
PL12W 0.41565308 W
PL13W 0.41565308 W
SFO2 500.1320005 MHz
SI 32768
SF 125.7577919 MHz
WDW EM
SSB 0
LB 1.00 Hz
GB 0
PC 1.40
```

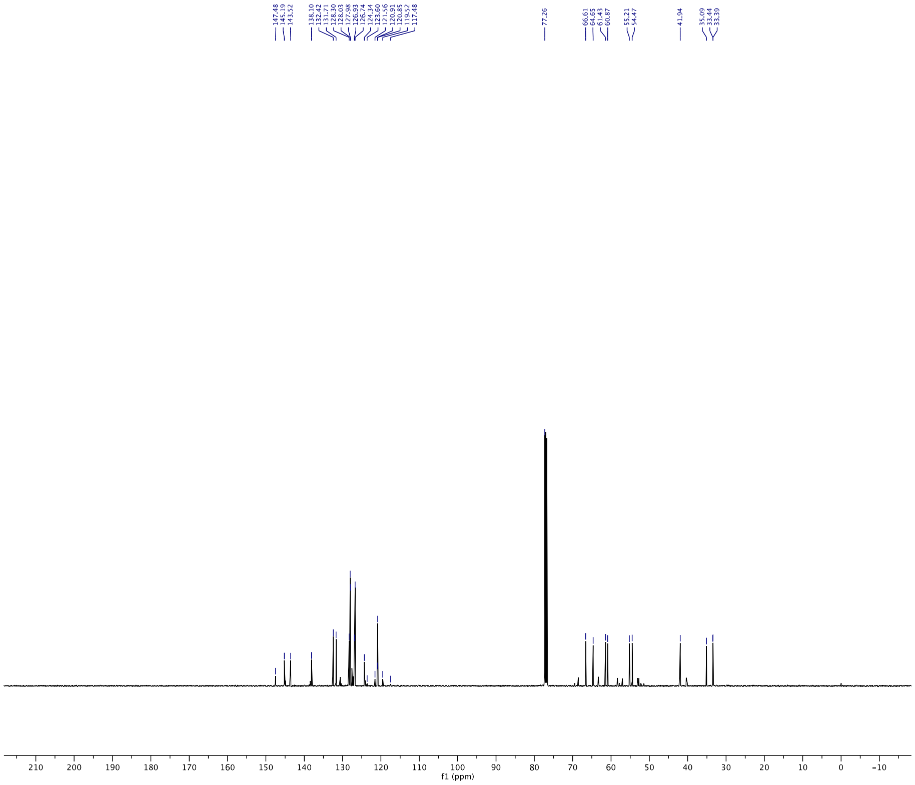

BGAz-015

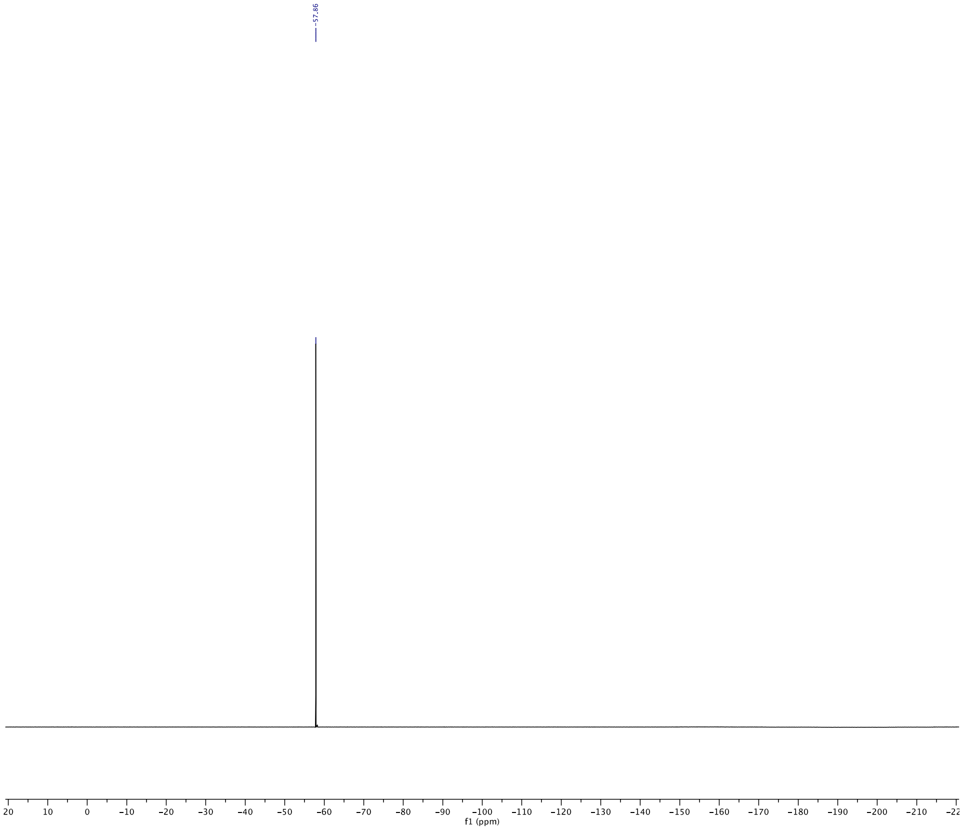

NAME May25-2017
EXPNO 2
PROCNO 1
Date_ 20170525
Time 17.56
INSTRUM Spect
PROBHD 5 mm PABBO BB-
PULPROG zgpg30
TD 65536
SOLVENT CDCl3
NS 2048
DS 4
SWH 29761.904 Hz
FIDRES 0.454131 Hz
AQ 1.1010548 sec
RG 203
DW 16.800 usec
DE 6.50 usec
TE 302.4 K
D1 2.00000000 sec
D11 0.03000000 sec
TD0 1

======== CHANNEL f1 ========
NUC1 13C
P1 8.80 usec
PL1 -1.00 dB
PL1W 104.98761749 W
SFO1 125.7703643 MHz

======== CHANNEL f2 ========
CPDPRG2 waltz16
NUC2 1H
PCPD2 80.00 usec
PL2 2.50 dB
PL12 17.46 dB
PL13 17.46 dB
PL2W 13.02359581 W
PL12W 0.41565308 W
PL13W 0.41565308 W
SFO2 500.1320005 MHz
SI 32768
SF 125.7577919 MHz
WDW EM
SSB 0
LB 1.00 Hz
GB 0
PC 1.40

BGAz-016

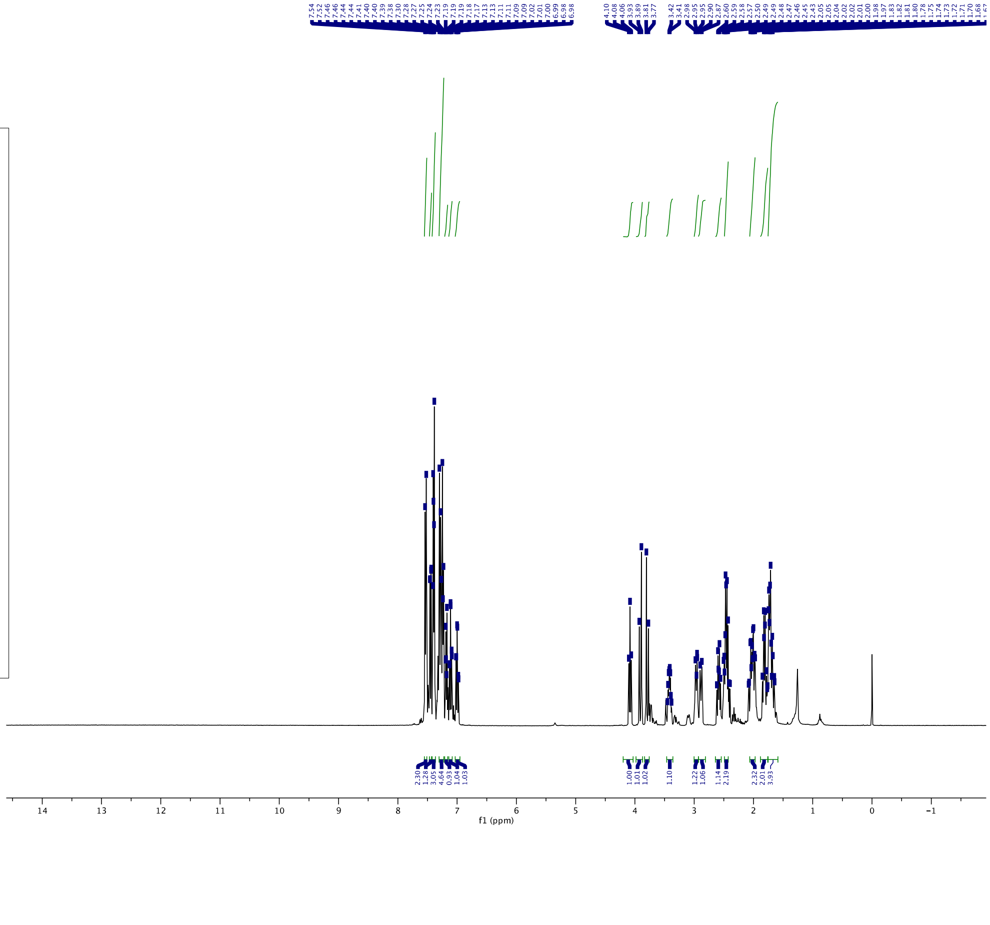

NAME May25-2017
EXPNO 2
PROCNO 1
Date_ 20170525
Time 17.56
INSTRUM Spect
PROBHD 5 mm PABBO BB-
PULPROG zgpg30
TD 65536
SOLVENT CDCl3
NS 2048
DS 4
SWH 29761.904 Hz
FIDRES 0.454131 Hz
AQ 1.1010548 sec
RG 203
DW 16.800 usec
DE 6.50 usec
TE 302.4 K
D1 2.00000000 sec
D11 0.03000000 sec
TD0 1

======== CHANNEL f1 ========
NUC1 13C
P1 8.80 usec
PL1 -1.00 dB
PL1W 104.98761749 W
SFO1 125.7703643 MHz

======== CHANNEL f2 ========
CPDPRG2 waltz16
NUC2 1H
PCPD2 80.00 usec
PL2 2.50 dB
PL12 17.46 dB
PL13 17.46 dB
PL2W 13.02359581 W
PL12W 0.41565308 W
PL13W 0.41565308 W
SFO2 500.1320005 MHz
SI 32768
SF 125.7577919 MHz
WDW EM
SSB 0
LB 1.00 Hz

BGAz-016

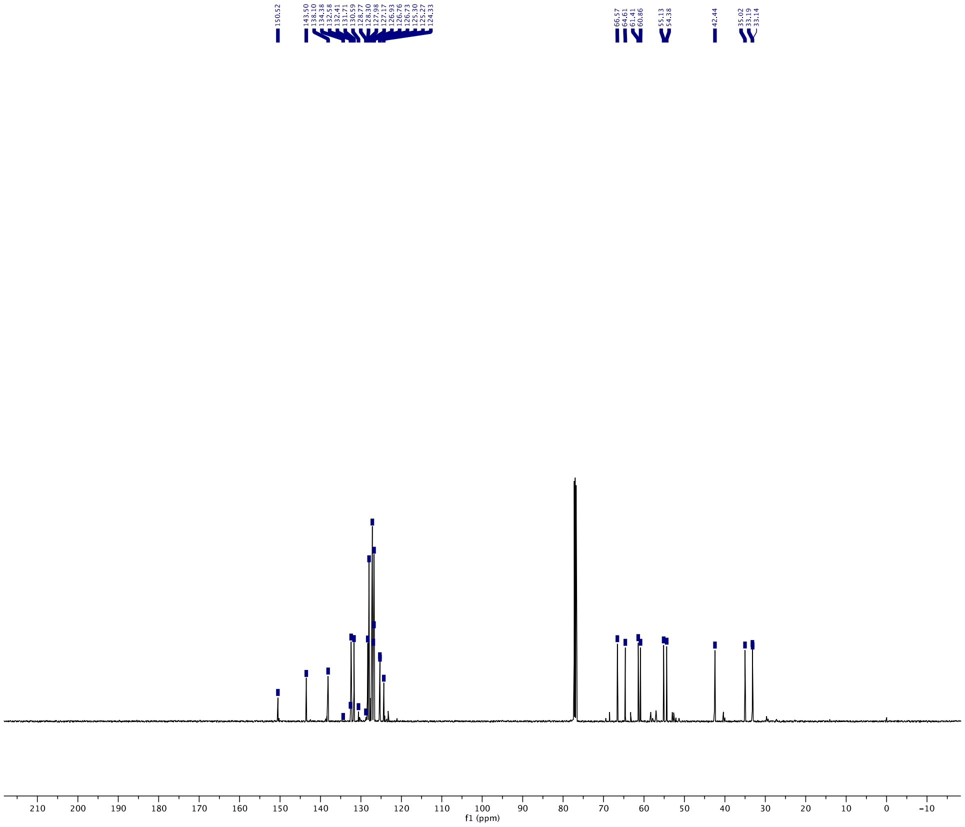

NAME May25-2017
EXPNO 2
PROCNO 1
Date_ 20170525
Time 17.56
INSTRUM Spect
PROBHD 5 mm PABBO BB-
PULPROG zgpg30
TD 65536
SOLVENT CDCl3
NS 2048
DS 4
SWH 29761.904 Hz
FIDRES 0.454131 Hz
AQ 1.1010548 sec
RG 203
DW 16.800 usec
DE 6.50 usec
TE 302.4 K
D1 2.00000000 sec
D11 0.03000000 sec
TD0 1

======== CHANNEL f1
========
NUC1 13C
P1 8.80 usec
PL1 -1.00 dB
PL1W 104.98761749 W
SFO1 125.7703643 MHz

======== CHANNEL f2
========
CPDPRG2 waltz16
NUC2 1H
PCPD2 80.00 usec
PL2 2.50 dB
PL12 17.46 dB
PL13 17.46 dB
PL2W 13.02359581 W
PL12W 0.41565308 W
PL13W 0.41565308 W
SFO2 500.1320005 MHz
SI 32768
SF 125.7577919 MHz
WDW EM
SSB 0
LB 1.00 Hz
GB 0
PC 1.40

BGAz-016

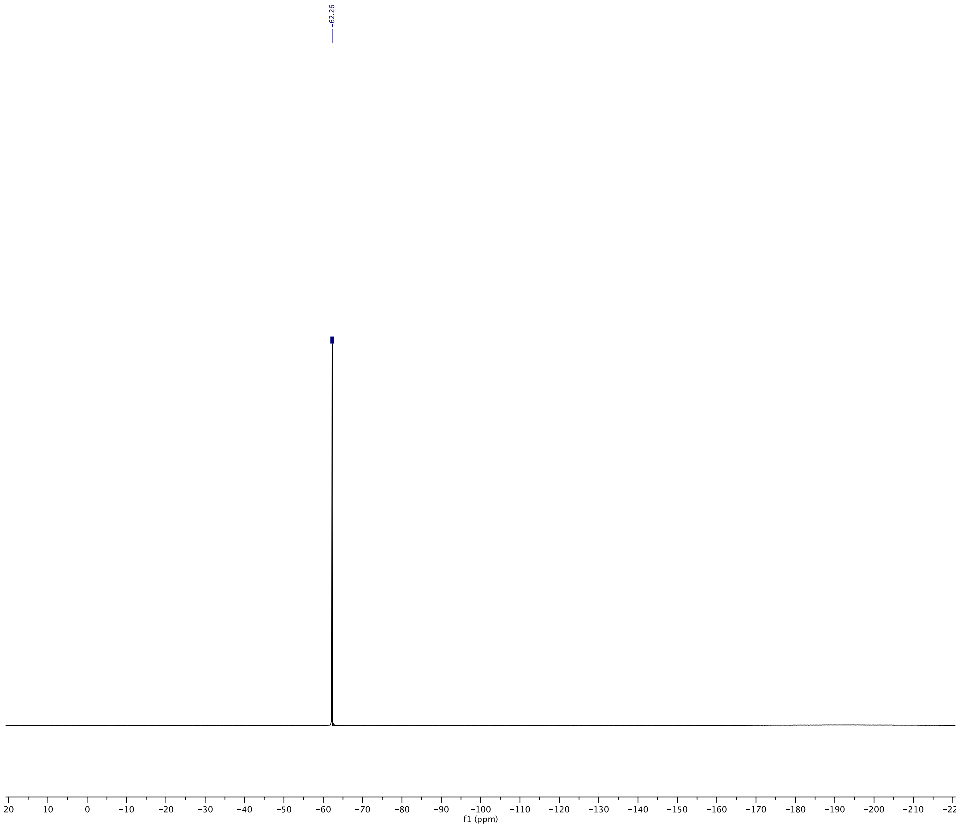

NAME May25-2017
EXPNO 2
PROCNO 1
Date_ 20170525
Time 17.56
INSTRUM Spect
PROBHD 5 mm PABBO BB-
PULPROG zgpg30
TD 65536
SOLVENT CDCl3
NS 2048
DS 4
SWH 29761.904 Hz
FIDRES 0.454131 Hz
AQ 1.1010548 sec
RG 203
DW 16.800 usec
DE 6.50 usec
TE 302.4 K
D1 2.00000000 sec
D11 0.03000000 sec
TD0 1

======== CHANNEL f1
========
NUC1 13C
P1 8.80 usec
PL1 -1.00 dB
PL1W 104.98761749 W
SFO1 125.7703643 MHz

======== CHANNEL f2
========
CPDPRG2 waltz16
NUC2 1H
PCPD2 80.00 usec
PL2 2.50 dB
PL12 17.46 dB
PL13 17.46 dB
PL2W 13.02359581 W
PL12W 0.41565308 W
PL13W 0.41565308 W
SFO2 500.1320005 MHz
SI 32768
SF 125.7577919 MHz
WDW EM
SSB 0
LB 1.00 Hz
GB 0
PC 1.40

Mass Spectrometry BGAz001-005

BGAz-001-MS

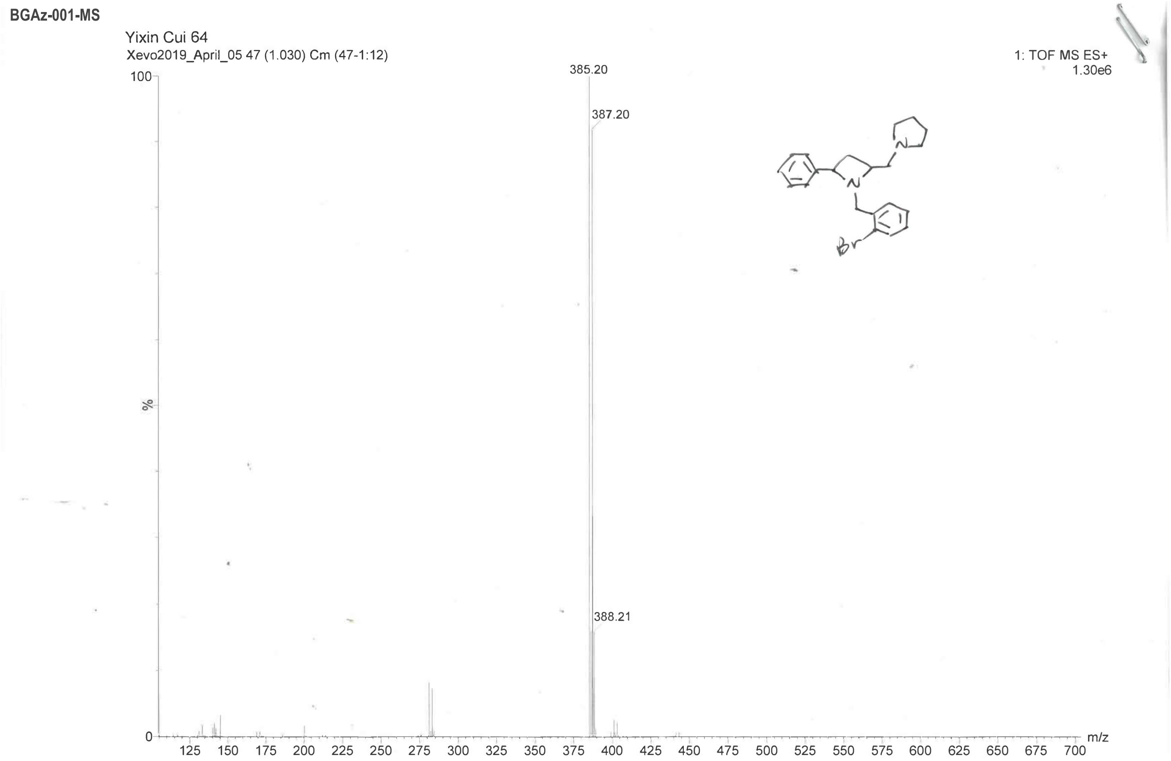

BGAz-001-HRMS

Elemental Composition Report **Yixin Cui 64**

Single Mass Analysis

Tolerance = 5.0 PPM / DBE: min = -1.5, max = 50.0

Element prediction: Off

Monoisotopic Mass, Even Electron Ions

27 formula(e) evaluated with 1 results within limits (up to 50 closest results for each mass)

Elements Used:

C: 0-100 H: 0-100 N: 0-5 79Br: 1-1

Minimum:			-1.5		
Maximum:	5.0	5.0	50.0		
Mass	Calc. Mass	mDa	PPM	DBE	Formula
**385.1277**	385.1279	-0.2	**-0.5**	9.5	**C21 H26 N2 79Br**

BGAz-002-MS-1

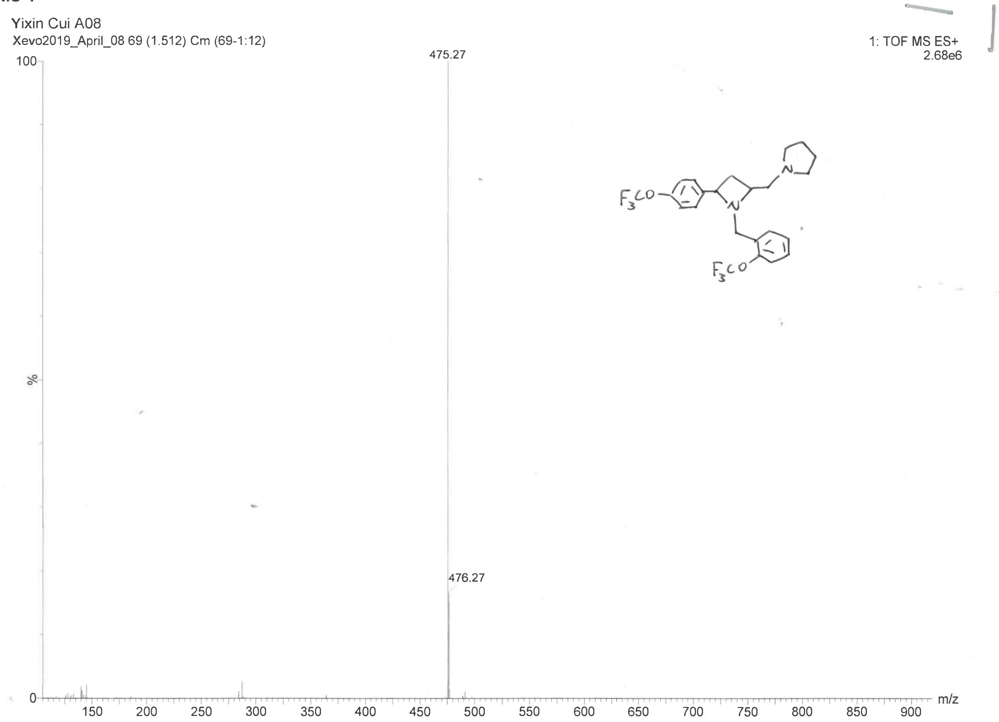

BGAz-002-HRMS-1

Elemental Composition Report **Yixin Cui A08**

Single Mass Analysis

Tolerance = 5.0 PPM / DBE: min = -1.5, max = 50.0

Element prediction: Off

Monoisotopic Mass, Odd and Even Electron Ions

100 formula(e) evaluated with 1 results within limits (up to 50 closest results for each mass)

Elements Used:

C: 0-100 H: 0-100 N: 0-3 O: 2-2 19F: 2-6

Minimum:				-1.5	
Maximum:		5.0	5.0	50.0	
Mass	Calc. Mass	mDa	PPM	DBE	Formula
**475.1825**	475.1820	0.5	**1.1**	9.5	**C23 H25 N2 O2 19F6**

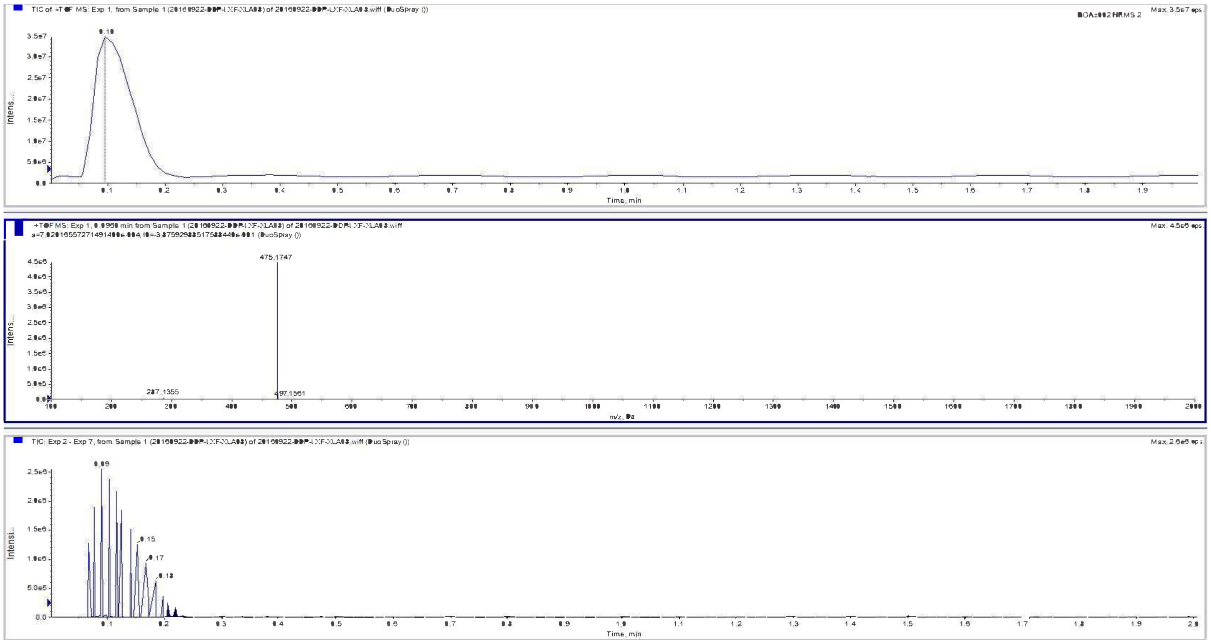

BGAz-003-MS-1
Yixin Cui 91
Xevo2019_April_75 32 (0.704) Cm (32:34-(1:15+192:231))
1: TOF MS ES+
2.67e6
523.10
521.10
524.11
BGAz-003

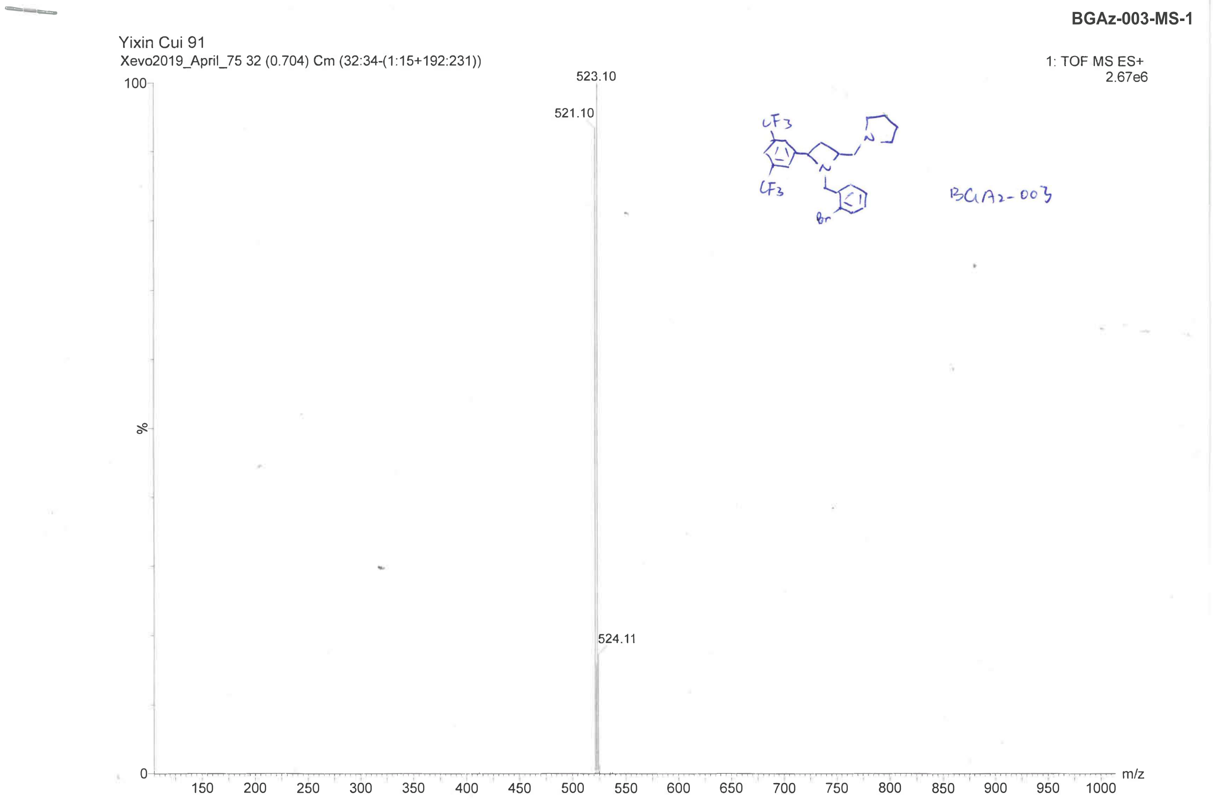

Elemental Composition Report **Yixin Cui YC91**

Single Mass Analysis

Tolerance = 5.0 PPM / DBE: min = -1.5, max = 50.0

Element prediction: Off

Monoisotopic Mass, Even Electron Ions

189 formula(e) evaluated with 1 results within limits (up to 50 closest results for each mass)

Elements Used:

C: 0-100 H: 0-100 N: 0-5 19F: 2-8 79Br: 1-1

Minimum:			-1.5		
Maximum:	5.0	5.0	50.0		
Mass	Calc. Mass	mDa	PPM	DBE	Formula
**521.1026**	521.1027	-0.1	**-0.2**	9.5	**C23 H24 N2 19F6 79Br**

BGAz-004 MS

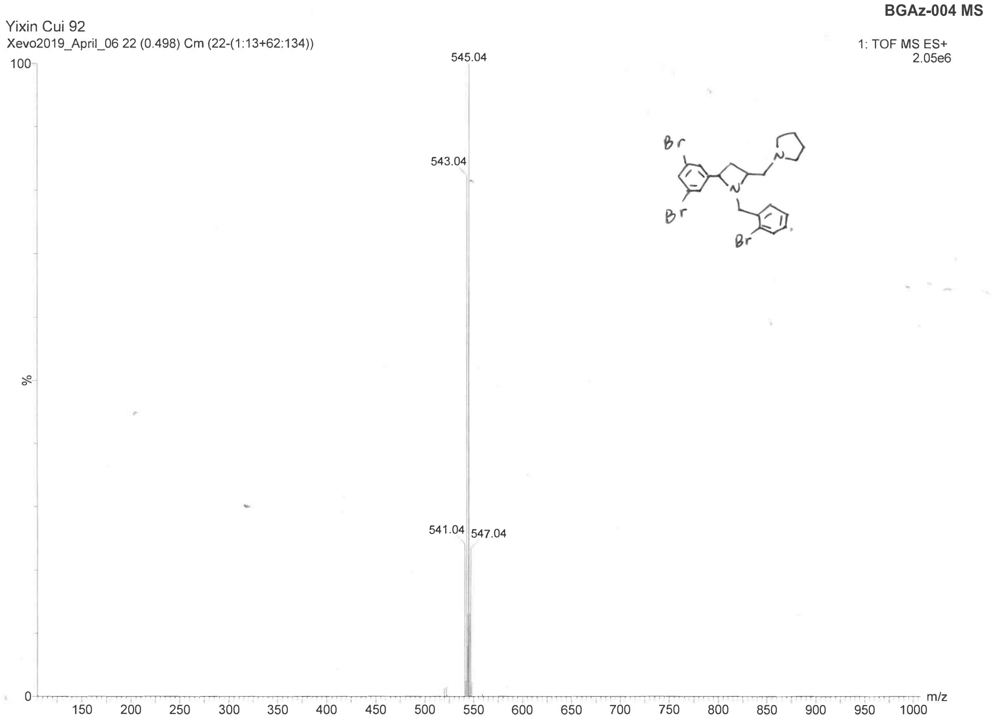

BGAz-004-HRMS

Elemental Composition Report **Yixin Cui 92**

Single Mass Analysis

Tolerance = 5.0 PPM / DBE: min = -1.5, max = 50.0

Element prediction: Off

Monoisotopic Mass, Odd and Even Electron Ions

104 formula(e) evaluated with 1 results within limits (up to 50 closest results for each mass)

Elements Used:

C: 0-100 H: 0-100 N: 1-5 79Br: 1-5

Minimum:			-1.5		
Maximum:	5.0	5.0	50.0		
Mass	Calc. Mass	mDa	PPM	DBE	Formula
**540.9489**	540.9490	-0.1	**-0.2**	9.5	**C21 H24 N2 79Br3**

BGAz-005 MS

D:\Data\Chemistry Mass Spectrometry Project\Yixin Cui YC-GIBH-122_GE2_01_300.d 7/17/2019 4:49:49 PM

Yixin Cui YC-GIBH-122

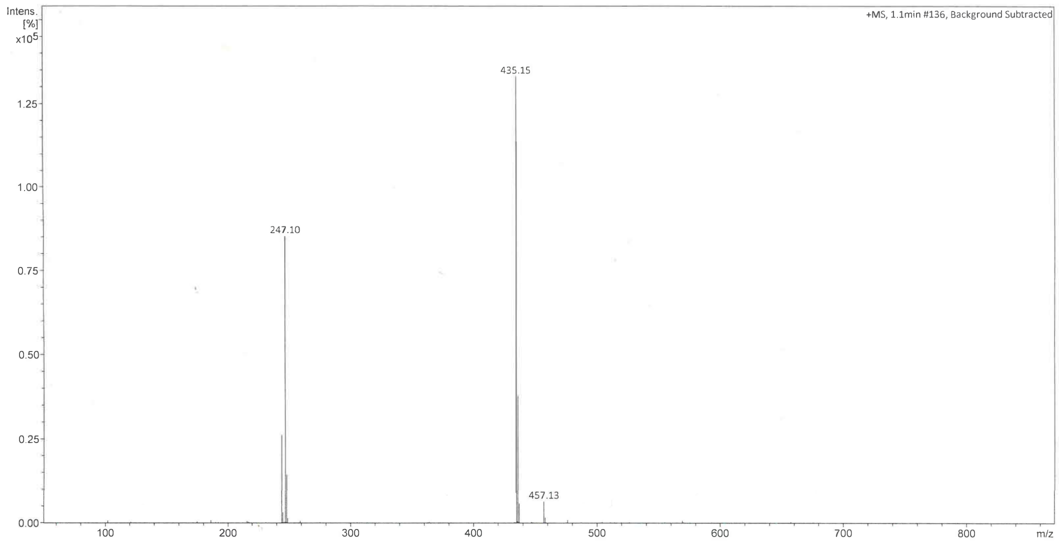

BGAz-005 HRMS

D:\Data\Chemistry Mass Spectrometry Project\Yixin Cui YC-GIBH-122_GE2_01_300.d 7/17/2019 4:49:49 PM

Yixin Cui YC-GIBH-122

Meas. m/z	#	Ion Formula	m/z	err [ppm]	mSigma	# mSigma	Score	rdb	e⁻ Conf	N-Rule
435.1505	1	C20H21F6N2O2	435.1502	-0.8	18.7	1	100.00	8.5	even	ok

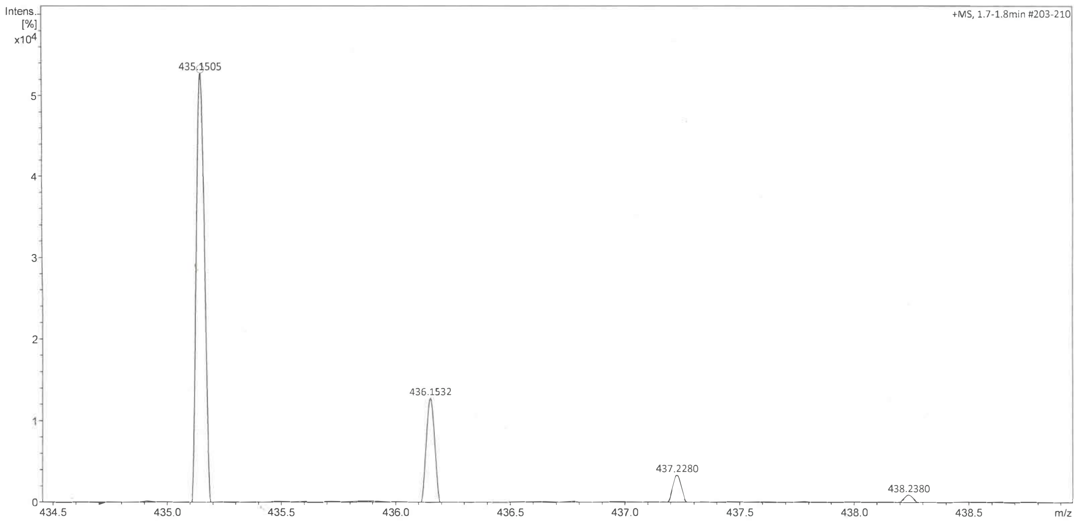

HPLC analysis of BGAz001–BGAz005

[illegible]

BGAz-001-HPLC

[illegible]

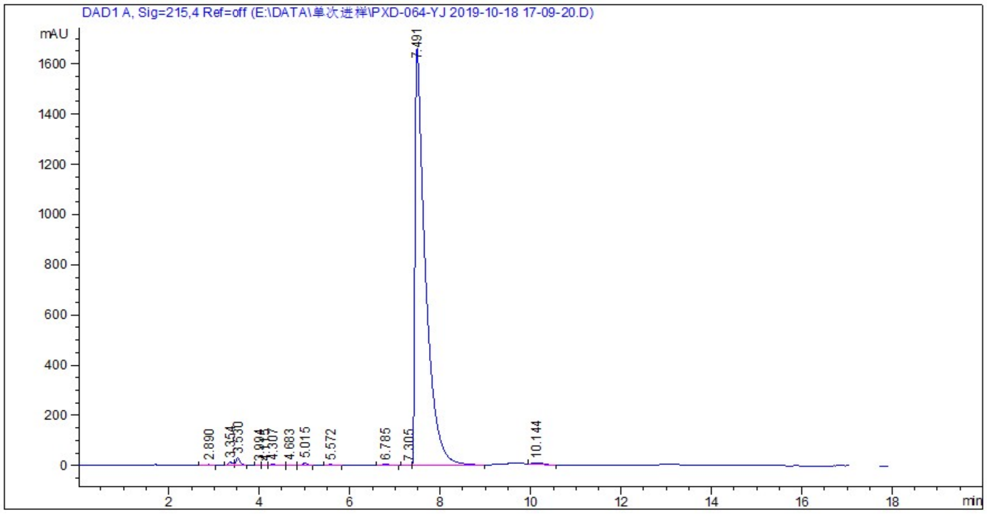

1: DAD1 A, Sig=215,4 Ref=off

#	[min]		[min]	[mAU*s]	[mAU]	%
1	2.890	BB	0.0830	21.44737	3.62478	0.0792
2	3.354	BV	0.0843	77.17467	13.18584	0.2850
3	3.530	VB	0.0873	175.25566	29.49627	0.6472
4	3.994	BV	0.0872	7.29503	1.30526	0.0269
5	4.115	VV	0.0839	7.49826	1.36981	0.0277
6	4.307	VB	0.1102	45.70713	6.04725	0.1688

BGAz-002-HPLC

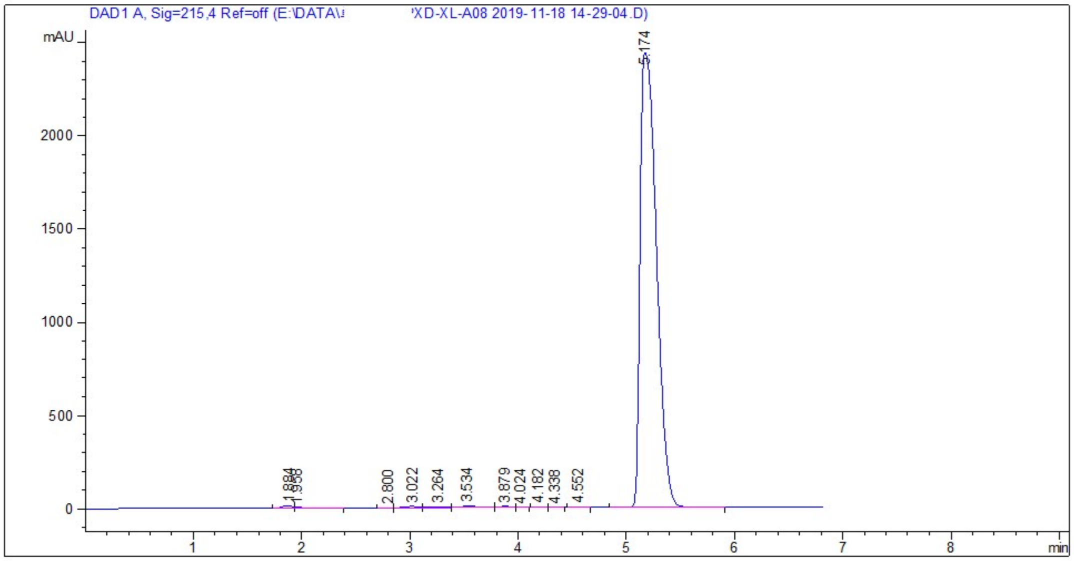

===

1: DAD1 A, Sig=215,4 Ref=off

	[min]		[min]	[mAU*s]	[mAU]	%
1	1.884	BV	0.0953	94.87852	13.28846	0.3642
2	1.958	VB	0.0719	39.90796	7.52465	0.1532
3	2.800	BB	0.0540	6.93734	2.02328	0.0266
4	3.022	BV	0.0805	55.11722	9.97407	0.2115
5	3.264	VB	0.1003	35.72900	5.32175	0.1371
6	3.534	BV	0.1537	111.60405	10.03849	0.4283
7	3.879	VV	0.1092	51.24211	7.18935	0.1967

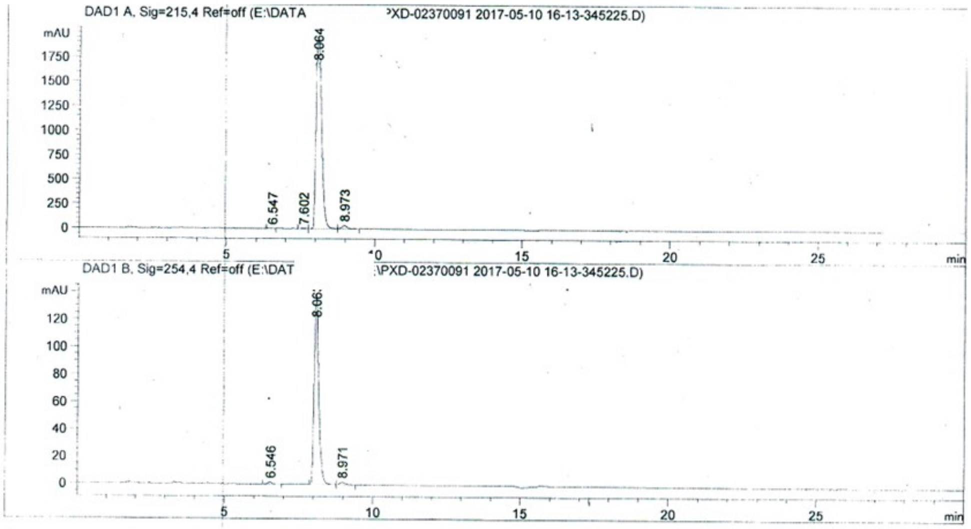

1: DAD1 A, Sig=215,4 Ref=off

#	[min]		[min]	[mAU*s]	[mAU]	%
1	6.547	BB	0.1058	16.01622	2.34190	0.0606
2	7.602	BB	0.1330	11.39994	1.26682	0.0431
3	8.064	BV	0.2110	2.59805e4	1956.12817	98.2938
4	8.973	VB	0.1916	423.54568	33.47721	1.6024
				2.64314e4	1993.21410	

BGAz-004-HPLC

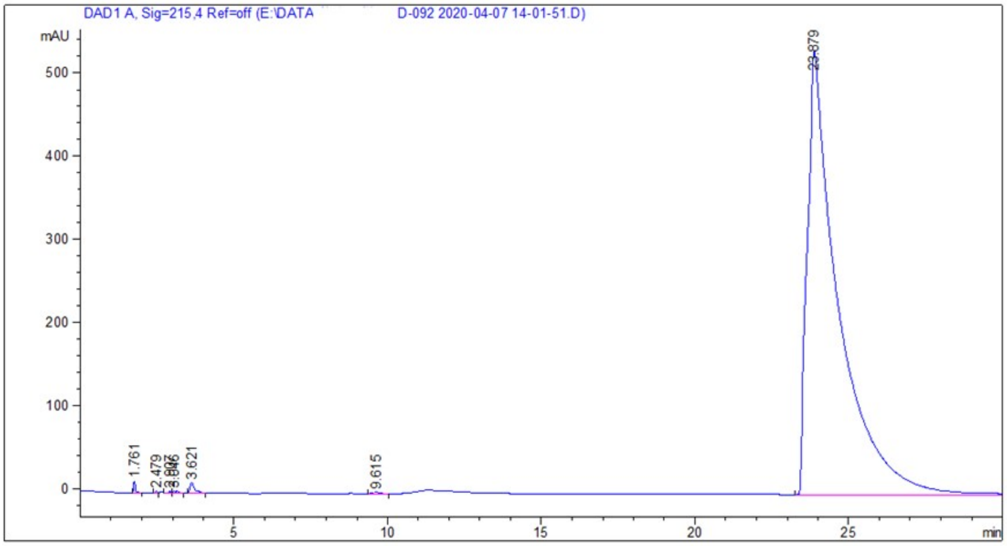

1: DAD1 A, Sig=215,4 Ref=off

#	[min]		[min]	[mAU*s]	[mAU]	%
1	1.761	BB	0.0589	54.39043	14.12255	0.1452
2	2.479	BB	0.0708	7.95832	1.90398	0.0212
3	2.907	BV	0.0814	8.05163	1.53018	0.0215
4	3.046	VB	0.1642	21.37819	1.70590	0.0571
5	3.621	BB	0.1570	132.90109	12.40872	0.3548
6	9.615	BB	0.2310	28.47347	1.91995	0.0760
7	23.879	BBA	0.8826	3.72078e4	532.88928	99.3242

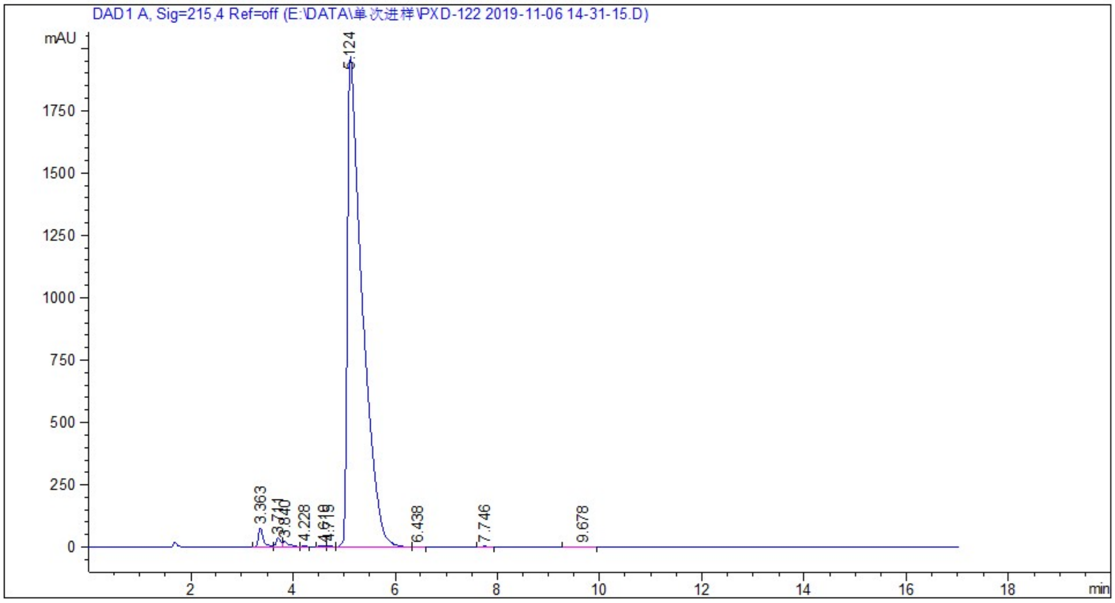

1: DAD1 A, Sig=215,4 Ref=off

#	[min]		[min]	[mAU*s]	[mAU]	%
1	3.363	BV	0.0930	475.38925	75.93010	1.1842
2	3.711	VV	0.0900	207.26859	34.52227	0.5163
3	3.840	VB	0.1084	160.22266	20.23956	0.3991
4	4.228	BB	0.0737	13.40368	2.90957	0.0334
5	4.610	BV	0.0908	15.34426	2.45905	0.0382
6	4.715	VB	0.0960	19.88466	3.13596	0.0495

References

(1) Feula, A.; Dhillon, S. S.; Byravan, R.; Sangha, M.; Ebanks, R.; Hama Salih, M. A.; Spencer, N.; Male, L.; Magyary, I.; Deng, W.-P.; Müller, F.; Fossey, J. S. *Org. Biomol. Chem.* **2013**, *11*, 5083.

(2) Feula, A.; Male, L.; Fossey, J. S. *Org. Lett.* **2010**, *12*, 5044.

(3) Abrahams, K. A.; Cox, J. A. G.; Spivey, V. L.; Loman, N. J.; Pallen, M. J.; Constantinidou, C.; Fernández, R.; Alemparte, C.; Remuiñán, M. J.; Barros, D.; Ballell, L.; Besra, G. S. *PLoS One* **2013**, *7*, e52951.

(4) Briffotaux, J.; Huang, W.; Wang, X.; Gicquel, B. *Tuberculosis* **2017**, *107*, 13.

In vivo pharmacokinetics parameters report BGAz compounds

(combination of three reports)

Table of Contents

In vivo pharmacokinetics parameters report BGAz compounds (combination of three reports) 1
Report on PK Results of Combined Dosing with BGAz-001–005 2
1 Materials and instrument combined dosing 2
2 Animal 2
3 Pharmacokinetic studies 2
4 Plasma sample and brain sample analysis 2
4.1 Standard curve sample preparation 2
4.2 Plasma and preparation 2
5 LC/MS/MS analysis 2
6 Results 3
6.1 Results for BGAz-001 4
6.2 Results for BGAz-002 9
6.3 Results for BGAz-003 13
6.4 Results for BGAz-004 18
6.5 Results for BGAz-005 22
Report on PK results of BGAz-004 by multiple oral dosing 27
7 Materials and instrument combined dosing 27
8 Animal 27
9 Pharmacokinetic studies 27
10 Plasma sample analysis 27
10.1 Standard curve sample preparation 27
10.2 Plasma and preparation 27
11 LC/MS/MS analysis 27
12 Results 28
Report on PK results of BGAz-005 by multiple oral dosing 30
13 Materials and instrument combined dosing 30
14 Animal 30
15 Pharmacokinetic studies 30
16 Plasma sample analysis 30
16.1 Standard curve sample preparation 30
16.2 Plasma and preparation 30
17 LC/MS/MS analysis 30
18 Results 31

Report on PK Results of Combined Dosing with BGAz-001–005

2019.12.27

1 Materials and instrument combined dosing

An MS2 Turnover type oscillator was purchased from IKA Work's Guangzhou (China), and a 5415R Chromatographic analyses were conducted using an Agilent 1290 Infinity II high performance liquid chromatography and Agilent Technologies 6470Triple Quad LC/MS. Propranolol (≥90% in purity, internal standard, IS) was purchased from the Sigma Chemical Co. (China). Formic acid (HPLC grade) and methanol (HPLC grade) were purchased from DIKMA Co. (China). All chemicals and solvents were analytical grade. Water was purified using a Millipore (AK, USA) laboratory ultra-pure water system (0.2 μm filter).

2 Animal

KM mice, weighing 25–35 g (Beijing Vital River Laboratory Animal Technology Co., Ltd., China) were utilised for the studies. The protocols were approved by the Animal Care and Use Committee, GIBH. Animals were maintained on standard animal chow and water *ad libitum*, in a climate-controlled room (23±1 °C 30–70% relative humidity, a minimum of ten exchanges of room air per hour and a 12 h light/dark cycle) for one week prior to experiments.

3 Pharmacokinetic studies

Compounds **BGAz-001**, **BGAz-002**, **BGAz-003**, **BGAz-004** and **BGAz-005** were dissolved mixed in a solution containing DMSO (2%), ethanol (4%), Cremophor EL (4%) and ddH_2O (90%); Pharmacokinetic properties of KM (male) were determined following IV, PO and IP administration. Animals were randomly distributed into three experimental groups (n = 4). The PO group was given mixed solution with 5 mg/kg by gastric gavage. The IV group was dosed with mixed solution 1mg/kg by injection into the tail vein. The IP group was dosed with mixed solution 1mg/kg by intraperitoneal injection. After single administration, whole blood samples (100 μL) were obtained from the orbital venous plexus at the following time points after dosing: 5, 15, 30 min and 1, 3, 5, 8, 24 hour (PO and IP groups) and at the following time points after dosing 2, 10, 30 min and 1, 3, 5, 8, 24 hour (IV group). Whole blood samples were collected in heparinised tubes. The plasma fraction was immediately separated by centrifugation. The mice were humanely euthanasia by carbon dioxide 24 hours after experiment without pain.

4 Plasma sample and brain sample analysis

4.1 Standard curve sample preparation

The compounds were dissolved in DMSO (2mg/mL) and diluted with to series concentration (methanol:H_2O, 1:1, 10 μL) and blank plasma (50 μL) were added to 1.5 mL tubes and vortexed for 3 min, then acetonitrile-containing internal standard (150 μL) was added and vortexed for a further 5 min, and subsequently spun in a centrifuge at 13000 ×*g* for 40 min at 4 °C, the final concentrations were as follow: 5, 10, 20, 50, 100, 200, 500, 1000 ng/mL.

4.2 Plasma and preparation

Plasma samples were prepared using a protein precipitation method. Solution (10 μL, methanol H_2O, 1:1) and plasma samples (50 μL) were added to 1.5 mL tubes and vortex for 3 min, then acetonitrile-containing internal standard (150 μL) was added and vortex for 5 min, and subsequently spun in a centrifuge at 13000 ×*g* for 40 min at 4 °C.

5 LC/MS/MS analysis

After centrifugation, supernatant (100 μL) was transfer to 96 well plates and analysed by LC-MS/MS using an Agilent 1290 Infinity II HPLC and an Agilent Technologies 6470Triple Quad LC/MS.

6 Results

Table 1. Pharmacokinetic parameters for BGAz-001.

	BGAz-001		
	**PO**	**IV**	**IP**
**Animal Number KM mice**	♂4	♂4	♂4
**Dose level mg/kg**	5	1	1
**AUC(0-∞) μg*h**	147.069	143.307	21.656
**$T_{1/2}$ (h)**	1.482	1.003	2.128
**T_{max} (h)**	0.312	0.033	0.125
**C_{max}（μg/L）**	54.708	157.627	21.923
**BA (%)**		20.53	

Table 2. Pharmacokinetic parameters for BGAz-002.

	BGAz-002		
	**PO**	**IV**	**IP**
**Animal Number KM mice**	♂4	♂4	♂4
**Dose level mg/kg**	5	1	1
**AUC(0-∞) μg*h**	1268.03	440.39	629.982
**$T_{1/2}$ (h)**	24.921	8.349	16.122
**T_{max} (h)**	1.875	0.033	0.167
**C_{max}（μg/L）**	53.858	188.69	39.955
**BA (%)**		57.59	

Table 3. Pharmacokinetic parameters for BGAz-003.

	BGAz-003		
	**PO**	**IV**	**IP**
**Animal Number KM mice**	♂4	♂4	♂4
**Dose level mg/kg**	5	1	1
**AUC(0-∞) μg*h**	1403.716	563.438	849.246
**$T_{1/2}$ (h)**	27.991	10.834	21.3
**T_{max} (h)**	1.375	0.033	0.167
**C_{max}（μg/L）**	56.034	254.543	49.137
**BA (%)**		49.83	

Table 4. Pharmacokinetic parameters for BGAz-004.

	BGAz-004		
	**PO**	**IV**	**IP**
**Animal Number KM mice**	♂4	♂4	♂4
**Dose level mg/kg**	5	1	1
**AUC(0-∞) μg*h**	1406.796	789.43	581.359
**$T_{1/2}$ (h)**	11.368	8.289	9.823
**T_{max} (h)**	0.875	0.033	0.271
**C_{max}（μg/L）**	87.649	278.395	61.021
**BA (%)**		35.64	

Table 5. Pharmacokinetic parameters for BGAz-005.

	BGAz-005		
	**PO**	**IV**	**IP**
**Animal Number KM mice**	♂4	♂4	♂4
**Dose level mg/kg**	5	1	1
**AUC(0-∞) μg*h**	1542.381	424.729	464.151
**$T_{1/2}$ (h)**	35.733	8.052	9.339
**T_{max} (h)**	6	0.033	0.646
**C_{max}（μg/L）**	43.127	320.487	41.897
**BA (%)**		72.63	

6.1 *Results for BGAz-001*

Table 6. Concentration of **BGAz-001** for PO (μg/L).

Time (h)	No. 1 (μg/L)	No. 2 (μg/L)	No. 3 (μg/L)	No. 4 (μg/L)	Mean (μg/L)	SD
0.083	144.18	20.10	6.67	30.82	50.44	63.27
0.25	29.52	1.90	4.72	6.73	10.72	12.69
0.5	31.49	4.96	13.11	9.48	14.76	11.64
1	22.70	10.80	23.73	17.83	18.76	5.90
3	15.54	9.49	18.32	19.20	15.64	4.39
5	13.54	6.93	death	12.38	10.95	3.53
8	9.03	9.67	death	7.54	8.75	1.09
24	0.00	0.00	death	0.00	0.00	0.00

Table 7. Concentration of **BGAz-001** for PO (μM).

Time (h)	No. 1 (μM)	No. 2 (μM)	No. 3 (μM)	No. 4 (μM)	Mean (μg/L)	SD
0.083	0.38	0.05	0.02	0.08	0.13	0.16
0.25	0.08	0.00	0.01	0.02	0.03	0.03
0.5	0.08	0.01	0.03	0.02	0.04	0.03
1	0.06	0.03	0.06	0.05	0.05	0.02
3	0.04	0.02	0.05	0.05	0.04	0.01
5	0.04	0.02	death	0.03	0.03	0.01
8	0.02	0.03	death	0.02	0.02	0.00
24	0.00	0.00	death	0.00	0.00	0.00

Table 8. Pharmacokinetic parameters of **BGAz-001** for PO.

Parameter	Unit	No. 1	No. 2	No. 3	No. 4	Mean	SD	RSD/%
**AUC(0-t)**	µg*	215.047	146.456	54.715	172.051	147.067	67.761	46.1
**AUC(0-∞)**	µg*	215.049	146.458	54.715	172.053	147.069	67.762	46.1
**R_AUC(t/∞)**	%	100	100	100	100	100	0	0
**AUMC(0-t)**	**µg	985.855	893.394	87.392	867.533	708.544	417.204	58.9
**AUMC(0-∞)**	**µg	985.912	893.454	87.392	867.588	708.587	417.233	58.9
**MRT(0-t)**		4.584	6.1	1.597	5.042	4.331	1.93	44.6
**MRT(0-∞)**		4.585	6.1	1.597	5.043	4.331	1.93	44.6
**VRT(0-t)**	^2	9.89	7.162	1.028	7.853	6.483	3.817	58.9
**VRT(0-∞)**	^2	9.894	7.168	1.028	7.858	6.487	3.819	58.9
**λz**	1/	0.476	0.448	0	0.48	0.351	0.234	66.7
**λz**		134	134		134	--	--	--
**C_last**	µg	0.001	0.001	1	0.001	0.251	0.5	199.2
**t1/2z**		1.456	1.547		1.443	1.482	0.057	3.8
**Tmax**		0.083	0.083	1	0.083	0.312	0.459	147.1
**Vz/F**	L/kg	48.84	76.229		60.517	61.862	13.744	22.2
**CLz/F**	L//kg	23.251	34.139	91.383	29.061	44.459	31.598	71.1
**Cmax**	µg	144.1835178	20.09557627	23.73036304	30.82118547	54.708	59.817	109.3

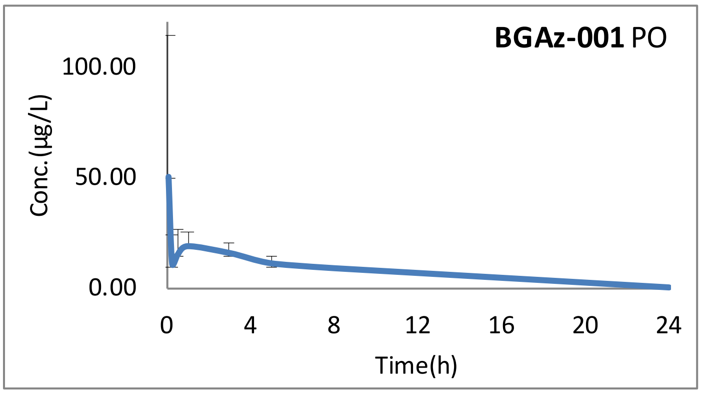

Figure 1

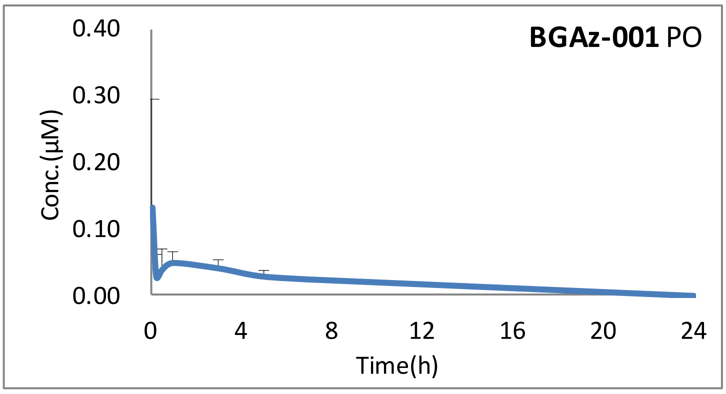

Figure 2

Table 9. Concentration of **BGAz-001** for IV (µg/L).

Time (h)	No. 1 (µg/L)	No. 2 (µg/L)	No. 3 (µg/L)	No. 4 (µg/L)	Mean (µg/L)	SD
0.033	180.84	161.14	222.24	66.28	157.63	66.00
0.167	85.06	102.64	124.42	54.81	91.73	29.41
0.5	35.31	53.80	58.88	25.46	43.36	15.65
1	21.78	66.59	47.23	18.05	38.41	22.82
3	4.22	13.07	12.54	5.18	8.75	4.70
5	0.79	15.12	0.00	0.00	3.98	7.44
8	0.00	5.14	0.00	0.00	1.29	2.57
24	0.00	0.00	0.00	0.00	0.00	0.00

Table 10. Concentration of **BGAz-001** for IV (µM).

Time (h)	No. 1 (µM)	No. 2 (µM)	No. 3 (µM)	No. 4 (µM)	Mean (µg/L)	SD
0.033	0.47	0.42	0.58	0.17	0.41	0.17
0.167	0.22	0.27	0.32	0.14	0.24	0.08
0.5	0.09	0.14	0.15	0.07	0.11	0.04
1	0.06	0.17	0.12	0.05	0.10	0.06
3	0.01	0.03	0.03	0.01	0.02	0.01
5	0.00	0.04	0.00	0.00	0.01	0.02
8	0.00	0.01	0.00	0.00	0.00	0.01
24	0.00	0.00	0.00	0.00	0.00	0.00

Table 11. Pharmacokinetic parameters of **BGAz-001** for IV.

Parameter	Unit	No. 1	No. 2	No. 3	No. 4	Mean	SD	RSD/%
**AUC(0-t)**	µg*	90.889	258.845	160.483	63.007	143.306	87.254	60.9
**AUC(0-∞)**	µg*	90.892	258.847	160.483	63.007	143.307	87.254	60.9
**R_AUC(t/∞)**	%	100	100	100	100	100	0	0
**AUMC(0-t)**	**µg	73.552	757.268	152.002	61.262	261.021	333.264	127.7
**AUMC(0-∞)**	**µg	73.619	757.321	152.002	61.262	261.051	333.278	127.7
**MRT(0-t)**		0.809	2.926	0.947	0.972	1.414	1.011	71.5
**MRT(0-∞)**		0.81	2.926	0.947	0.972	1.414	1.011	71.5
**VRT(0-t)**	^2	1.069	8.43	0.921	0.94	2.84	3.727	131.2
**VRT(0-∞)**	^2	1.088	8.434	0.921	0.94	2.846	3.726	130.9
**λz**	1/	0.379	0.516	1.738	1.575	1.052	0.703	66.8
**λz**		134	123	234	234	--	--	--
**C_last**	µg	0.001	0.001	0	0	0.001	0.001	100
**t1/2z**		1.829	1.344	0.399	0.44	1.003	0.702	70
**Tmax**		0.033	0.033	0.033	0.033	0.033	0	0
**Vz/F**	L/kg	29.038	7.491	3.585	10.074	12.547	11.313	90.2
**CLz/F**	L//kg	11.002	3.863	6.231	15.871	9.242	5.324	57.6
**Cmax**	µg	180.8432007	161.1415149	222.239702	66.28256575	157.627	66.005	41.9
**C0**	µg	217.759	180.074	256.369	69.46	180.916	80.568	44.5

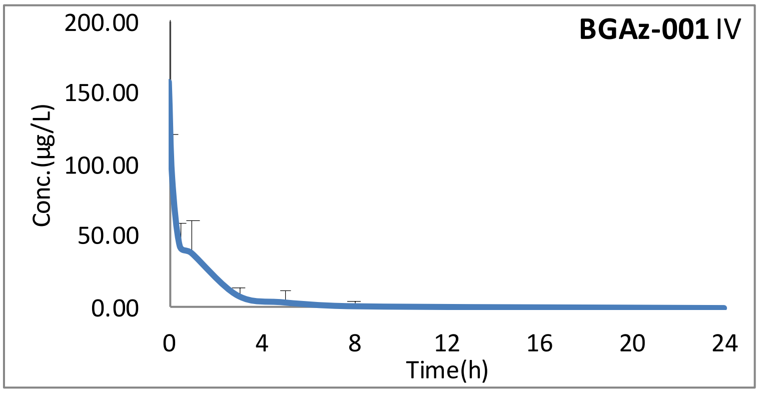

Figure 3

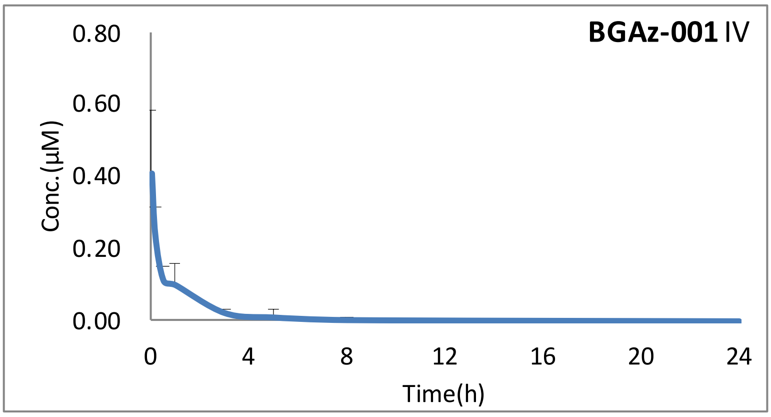

Figure 4

Table 12. Concentration of **BGAz-001** for IP (μg/L).

Time (h)	No. 1 (μg/L)	No. 2 (μg/L)	No. 3 (μg/L)	No. 4 (μg/L)	Mean (μg/L)	SD
0.083	36.79	16.68	11.71	12.46	19.41	11.79
0.25	17.42	8.00	9.69	22.50	14.40	6.78
0.5	12.01	5.99	9.68	12.89	10.14	3.08
1	3.14	3.25	7.60	7.17	5.29	2.43
3	0.36	0.00	4.54	1.45	1.59	2.06
5	0.00	0.00	/	0.00	0.00	0.00
8	0.00	0.39	0.00	0.00	0.10	0.19
24	0.00	0.00	0.00	0.00	0.00	0.00

Table 13. Concentration of **BGAz-001** for IP (μM).

Time (h)	No. 1 (μM)	No. 2 (μM)	No. 3 (μM)	No. 4 (μM)	Mean (μg/L)	SD
0.083	0.10	0.04	0.03	0.03	0.05	0.03
0.25	0.05	0.02	0.03	0.06	0.04	0.02
0.5	0.03	0.02	0.03	0.03	0.03	0.01
1	0.01	0.01	0.02	0.02	0.01	0.01
3	0.00	0.00	0.01	0.00	0.00	0.01
5	0.00	0.00	/	0.00	0.00	0.00
8	0.00	0.00	0.00	0.00	0.00	0.00
24	0.00	0.00	0.00	0.00	0.00	0.00

Table 14. Pharmacokinetic parameters of **BGAz-00x** for IP.

Parameter	Unit	No. 1	No. 2	No. 3	No. 4	Mean	SD	RSD/%
**AUC(0-t)**	μg*	17.377	13.769	32.506	22.953	21.651	8.163	37.7
**AUC(0-∞)**	μg*	17.377	13.783	32.509	22.953	21.656	8.16	37.7
**R_AUC(t/∞)**	%	100	99.9	100	100	99.975	0.05	0.1
**AUMC(0-t)**	**μg	9.629	35.382	59.605	21.387	31.501	21.491	68.2
**AUMC(0-∞)**	**μg	9.629	35.827	59.673	21.387	31.629	21.548	68.1
**MRT(0-t)**		0.554	2.57	1.834	0.932	1.473	0.908	61.6
**MRT(0-∞)**		0.554	2.599	1.836	0.932	1.48	0.92	62.2
**VRT(0-t)**	^2	0.373	10.94	1.361	0.725	3.35	5.077	151.6
**VRT(0-∞)**	^2	0.373	11.871	1.409	0.725	3.595	5.534	153.9
**λz**	1/	1.085	0.124	0.394	1.341	0.736	0.571	77.6
**λz**		234	123	134	234	--	--	--
**C_last**	μg	0	0.002	0.001	0	0.001	0.001	100
**t1/2z**		0.639	5.595	1.761	0.517	2.128	2.378	111.7
**Tmax**		0.083	0.083	0.083	0.25	0.125	0.084	67.2
**Vz/F**	L/kg	53.038	585.72	78.146	32.491	187.349	266.236	142.1
**CLz/F**	L//kg	57.547	72.553	30.761	43.568	51.107	18.002	35.2
**Cmax**	μg	36.79392484	16.67918911	11.7147724	22.5021479	21.923	10.85	49.5

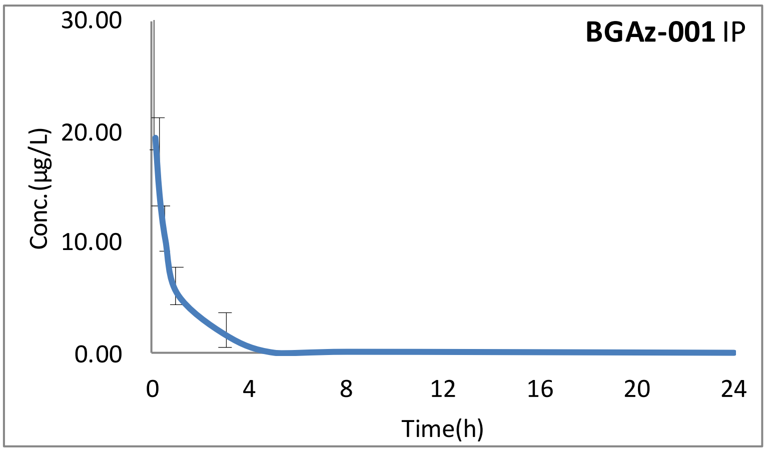

Figure 5

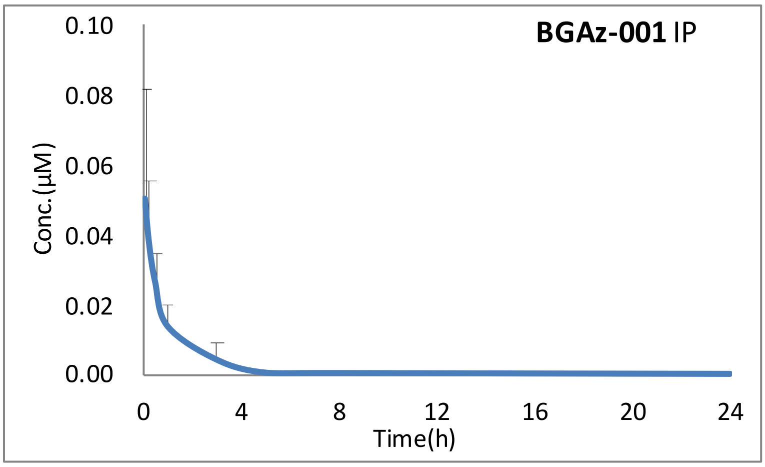

Figure 6

6.2 *Results for* BGAz-002

Table 15. Concentration of **BGAz-002** for PO (μg/L).

Time (h)	No. 1 (μg/L)	No. 2 (μg/L)	No. 3 (μg/L)	No. 4 (μg/L)	Mean (μg/L)	SD
0.083	22.70	18.44	12.42	12.17	16.43	5.09
0.25	30.92	22.35	26.54	20.79	25.15	4.55
0.5	45.43	34.31	37.96	33.08	37.69	5.56
1	38.47	53.36	47.84	57.44	49.28	8.21
3	35.15	56.60	55.96	49.78	49.37	9.97
5	29.27	42.52	death	48.54	40.11	9.86
8	30.25	42.15	death	37.76	36.72	6.02
24	24.18	22.29	death	22.40	22.95	1.06

Table 16. Concentration of **BGAz-002** for PO (μM).

Time (h)	No. 1 (μM)	No. 2 (μM)	No. 3 (μM)	No. 4 (μM)	Mean (μg/L)	SD
0.083	0.05	0.04	0.03	0.03	0.03	0.01
0.25	0.07	0.05	0.06	0.04	0.05	0.01
0.5	0.10	0.07	0.08	0.07	0.08	0.01
1	0.08	0.11	0.10	0.12	0.10	0.02
3	0.07	0.12	0.12	0.10	0.10	0.02
5	0.06	0.09	death	0.10	0.08	0.02
8	0.06	0.09	death	0.08	0.08	0.01
24	0.05	0.05	death	0.05	0.05	0.00

Table 17. Pharmacokinetic parameters of **BGAz-002** for PO.

Parameter	Unit	No. 1	No. 2	No. 3	No. 4	Mean	SD	RSD/%
**AUC(0-t)**	μg*	698.688	884.816	137.083	848.886	642.368	346.37	53.9
**AUC(0-∞)**	μg*	2117.266	1394.629	137.083	1423.146	1268.031	824.688	65
**R_AUC(t/∞)**	%	33	63.4	100	59.6	64	27.548	43
**AUMC(0-t)**	**μg	7576.309	8429.064	236.316	8154.748	6099.109	3924.657	64.3
**AUMC(0-∞)**	**μg	125599.193	32448.81	236.316	36621.833	48726.538	53765.525	110.3
**MRT(0-t)**		10.844	9.526	1.724	9.606	7.925	4.178	52.7
**MRT(0-∞)**		59.321	23.267	1.724	25.733	27.511	23.791	86.5
**VRT(0-t)**	^2	71.792	58.534	1.167	61.059	48.138	31.837	66.1
**VRT(0-∞)**	^2	3529.144	560.135	1.167	684.723	1193.792	1585.023	132.8
**λz**	1/	0.017	0.043	0	0.039	0.025	0.02	80
**λz**		124	124		134	--	--	--
**C_last**	μg	23.963	22.056	1	22.457	17.369	10.944	63
**t1/2z**		41.024	16.019		17.721	24.921	13.971	56.1
**Tmax**		0.5	3	3	1	1.875	1.315	70.1
**Vz/F**	L/kg	139.798	82.871		89.842	104.17	31.051	29.8
**CLz/F**	L//kg	2.362	3.585	36.474	3.513	11.484	16.67	145.2
**Cmax**	μg	45.4286889	56.59584787	55.96190526	57.44444395	53.858	5.652	10.5

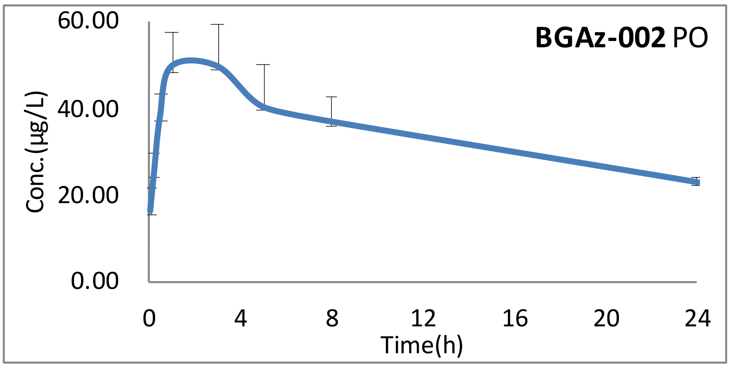

Figure 7

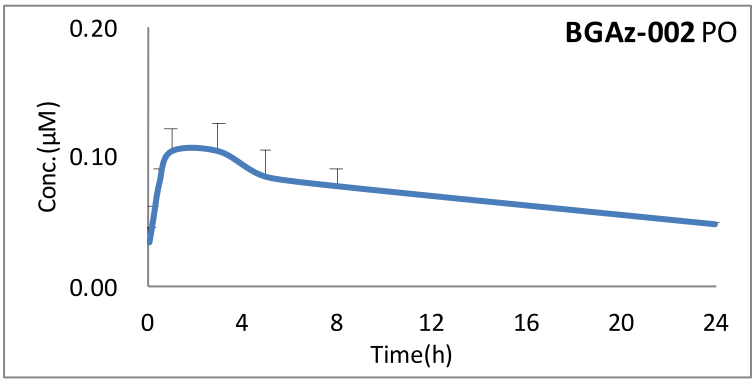

Figure 8

Table 18. Concentration of **BGAz-002** for IV (μg/L).

Time (h)	No. 1 (μg/L)	No. 2 (μg/L)	No. 3 (μg/L)	No. 4 (μg/L)	Mean (μg/L)	SD
0.033	220.68	204.88	235.95	93.24	188.69	64.89
0.167	113.21	103.79	107.54	71.07	98.90	18.95
0.5	63.20	73.71	74.67	38.53	62.53	16.82
1	43.14	54.66	50.75	33.90	45.61	9.16
3	26.15	32.42	30.42	24.06	28.26	3.83
5	14.69	24.54	18.33	15.70	18.32	4.42
8	10.73	13.38	13.37	12.48	12.49	1.25
24	4.80	12.59	3.91	3.33	6.16	4.33

Table 19. Concentration of **BGAz-002** for IV (μM).

Time (h)	No. 1 (μM)	No. 2 (μM)	No. 3 (μM)	No. 4 (μM)	Mean (μg/L)	SD
0.033	0.47	0.43	0.50	0.20	0.40	0.14
0.167	0.24	0.22	0.23	0.15	0.21	0.04
0.5	0.13	0.16	0.16	0.08	0.13	0.04
1	0.09	0.12	0.11	0.07	0.10	0.02
3	0.06	0.07	0.06	0.05	0.06	0.01
5	0.03	0.05	0.04	0.03	0.04	0.01
8	0.02	0.03	0.03	0.03	0.03	0.00
24	0.01	0.03	0.01	0.01	0.01	0.01

Table 20. Pharmacokinetic parameters of **BGAz-002** for IV.

Parameter	Unit	No. 1	No. 2	No. 3	No. 4	Mean	SD	RSD/%
**AUC(0-t)**	µg*	358.743	498.345	409.005	317.008	395.775	78.041	19.7
**AUC(0-∞)**	µg*	443.504	502.691	457.565	357.798	440.39	60.574	13.8
**R_AUC(t/∞)**	%	80.9	99.1	89.4	88.6	89.5	7.46	8.3
**AUMC(0-t)**	**µg	2150.014	4023.36	2261.527	1982.397	2604.325	952.954	36.6
**AUMC(0-∞)**	**µg	5698.919	4151.911	4034.703	3459.685	4336.305	957.452	22.1
**MRT(0-t)**		5.993	8.073	5.529	6.253	6.462	1.115	17.3
**MRT(0-∞)**		12.85	8.259	8.818	9.669	9.899	2.051	20.7
**VRT(0-t)**	^2	47.999	71.952	37.619	37.901	48.868	16.129	33
**VRT(0-∞)**	^2	298.828	75.566	141.33	141.277	164.25	94.92	57.8
**λz**	1/	0.056	0.179	0.08	0.082	0.099	0.054	54.5
**λz**		123	234	123	123	--	--	--
**C_last**	µg	4.743	0.778	3.88	3.339	3.185	1.706	53.6
**t1/2z**		12.384	3.871	8.673	8.466	8.349	3.486	41.8
**Tmax**		0.033	0.033	0.033	0.033	0.033	0	0
**Vz/F**	L/kg	40.291	11.111	27.351	34.144	28.224	12.573	44.5
**CLz/F**	L//kg	2.255	1.989	2.185	2.795	2.306	0.345	15
**Cmax**	µg	220.6847261	204.88032	235.9536049	93.24005526	188.69	64.885	34.4
**C0**	µg	260.116	242.233	286.328	99.688	222.091	83.587	37.6

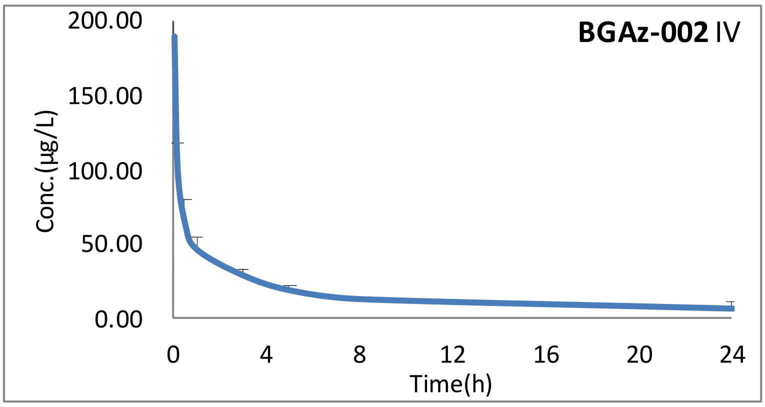

Figure 9

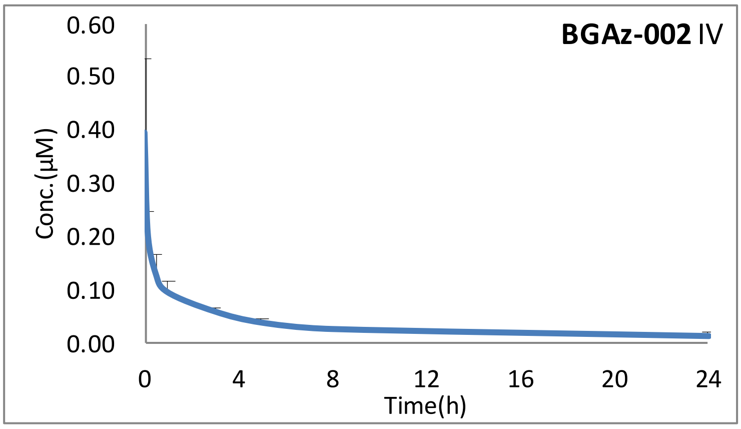

Figure 10

Table 21. Concentration of **BGAz-002** for IP (μg/L).

Time (h)	No. 1 (μg/L)	No. 2 (μg/L)	No. 3 (μg/L)	No. 4 (μg/L)	Mean (μg/L)	SD
0.083	52.67	33.27	27.07	36.75	37.44	10.91
0.25	54.59	28.96	26.71	44.89	38.79	13.29
0.5	41.62	25.98	25.65	37.33	32.65	8.08
1	30.90	22.07	21.44	28.77	25.79	4.75
3	29.46	15.73	25.12	25.84	24.04	5.85
5	15.26	13.45	/	22.15	16.96	4.59
8	19.65	11.65	11.32	17.23	14.96	4.14
24	15.40	14.27	10.09	9.93	12.42	2.82

Table 22. Concentration of **BGAz-002** for IP (μM).

Time (h)	No. 1 (μM)	No. 2 (μM)	No. 3 (μM)	No. 4 (μM)	Mean (μg/L)	SD
0.083	0.11	0.07	0.06	0.08	0.08	0.02
0.25	0.12	0.06	0.06	0.09	0.08	0.03
0.5	0.09	0.05	0.05	0.08	0.07	0.02
1	0.07	0.05	0.05	0.06	0.05	0.01
3	0.06	0.03	0.05	0.05	0.05	0.01
5	0.03	0.03	/	0.05	0.04	0.01
8	0.04	0.02	0.02	0.04	0.03	0.01
24	0.03	0.03	0.02	0.02	0.03	0.01

Table 23. Pharmacokinetic parameters of **BGAz-002** for IP.

Parameter	Unit	No. 1	No. 2	No. 3	No. 4	Mean	SD	RSD/%
**AUC(0-t)**	μg*	479.126	337.45	332.867	414.063	390.877	69.63	17.8
**AUC(0-∞)**	μg*	1027.494	413.093	607.489	471.851	629.982	277.229	44
**R_AUC(t/∞)**	%	46.6	81.7	54.8	87.8	67.725	20.097	29.7
**AUMC(0-t)**	**μg	4867.315	3922.348	3185.311	3693.675	3917.162	704.36	18
**AUMC(0-∞)**	**μg	38280.33	7016.073	17170.99	5791.21	17064.651	15035.3	88.1
**MRT(0-t)**		10.159	11.623	9.569	8.921	10.068	1.153	11.5
**MRT(0-∞)**		37.256	16.984	28.266	12.273	23.695	11.259	47.5
**VRT(0-t)**	^2	73.026	84.194	73.443	60.812	72.869	9.557	13.1
**VRT(0-∞)**	^2	1403.536	249.274	791.7	152.432	649.236	576.208	88.8
**λz**	1/	0.027	0.059	0.037	0.081	0.051	0.024	47.1
**λz**		124	234	134	234	--	--	--
**C_last**	μg	14.848	4.476	10.199	4.699	8.556	4.96	58
**t1/2z**		25.594	11.711	18.66	8.522	16.122	7.602	47.2
**Tmax**		0.25	0.083	0.083	0.25	0.167	0.096	57.5
**Vz/F**	L/kg	35.944	40.909	44.325	26.062	36.81	7.949	21.6
**CLz/F**	L//kg	0.973	2.421	1.646	2.119	1.79	0.631	35.3
**Cmax**	μg	54.59298631	33.26648936	27.07007106	44.88882257	39.955	12.239	30.6

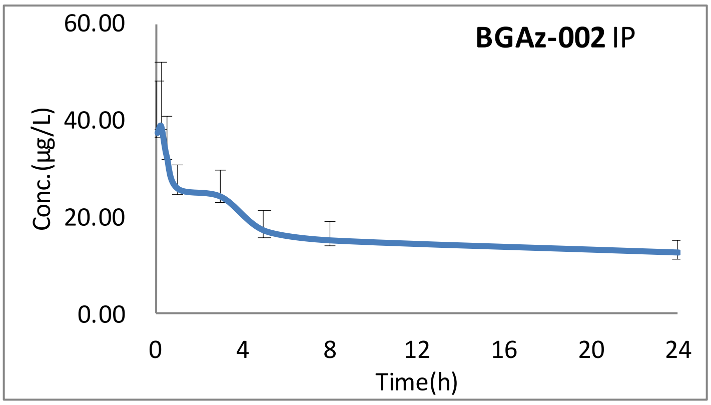

Figure 11

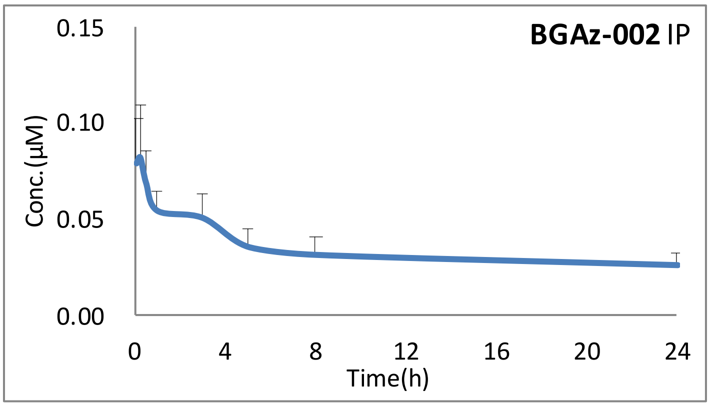

Figure 12

6.3 Results for BGAz-003

Table 24. Concentration of **BGAz-003** for PO (µg/L).

Time (h)	No. 1 (µg/L)	No. 2 (µg/L)	No. 3 (µg/L)	No. 4 (µg/L)	Mean (µg/L)	SD
0.083	15.47	17.39	11.81	10.58	13.81	3.17
0.25	34.32	27.35	30.32	23.35	28.83	4.64
0.5	51.22	39.33	41.56	36.72	42.21	6.32
1	43.03	59.13	53.16	59.58	53.72	7.71
3	36.20	60.18	50.03	50.81	49.30	9.88
5	30.57	43.70	death	45.91	40.06	8.29
8	31.31	40.51	death	35.15	35.65	4.62
24	24.56	25.92	death	23.91	24.80	1.03

Table 25. Concentration of **BGAz-003** for PO (µM).

Time (h)	No. 1 (µM)	No. 2 (µM)	No. 3 (µM)	No. 4 (µM)	Mean (µg/L)	SD
0.083	0.03	0.03	0.02	0.02	0.03	0.01
0.25	0.07	0.05	0.06	0.04	0.06	0.01
0.5	0.10	0.08	0.08	0.07	0.08	0.01
1	0.08	0.11	0.10	0.11	0.10	0.01
3	0.07	0.12	0.10	0.10	0.09	0.02
5	0.06	0.08	death	0.09	0.08	0.02
8	0.06	0.08	death	0.07	0.07	0.01
24	0.05	0.05	death	0.05	0.05	0.00

Table 26. Pharmacokinetic parameters of **BGAz-003** for PO.

Parameter	Unit	No. 1	No. 2	No. 3	No. 4	Mean	SD	RSD/%
**AUC(0-t)**	µg*	724.799	918.348	139.859	835.99	654.749	352.303	53.8
**AUC(0-∞)**	µg*	2104.452	1857.679	139.859	1512.875	1403.716	876.805	62.5
**R_AUC(t/∞)**	%	34.4	49.4	100	55.3	59.775	28.223	47.2
**AUMC(0-t)**	**µg	7759.603	9046.21	226.023	8223.172	6313.752	4093.211	64.8
**AUMC(0-∞)**	**µg	118968.77	65604.625	226.023	43660.783	57115.05	49380.611	86.5
**MRT(0-t)**		10.706	9.851	1.616	9.836	8.002	4.277	53.4
**MRT(0-∞)**		56.532	35.315	1.616	28.859	30.581	22.641	74
**VRT(0-t)**	^2	71.411	64.105	1.113	65.216	50.461	33.055	65.5
**VRT(0-∞)**	^2	3228.55	1328.702	1.113	842.672	1350.259	1367.026	101.2
**λz**	1/	0.018	0.028	0	0.035	0.02	0.015	75
**λz**		124	123		134	--	--	--
**C_last**	µg	24.373	25.94	1	23.873	18.797	11.897	63.3
**t1/2z**		39.228	25.095		19.649	27.991	10.106	36.1
**Tmax**		0.5	3	1	1	1.375	1.109	80.7
**Vz/F**	L/kg	134.493	97.464		93.709	108.555	22.541	20.8
**CLz/F**	L//kg	2.376	2.692	35.75	3.305	11.031	16.484	149.4
**Cmax**	µg	51.21708015	60.17758845	53.16155932	59.58024983	56.034	4.517	8.1

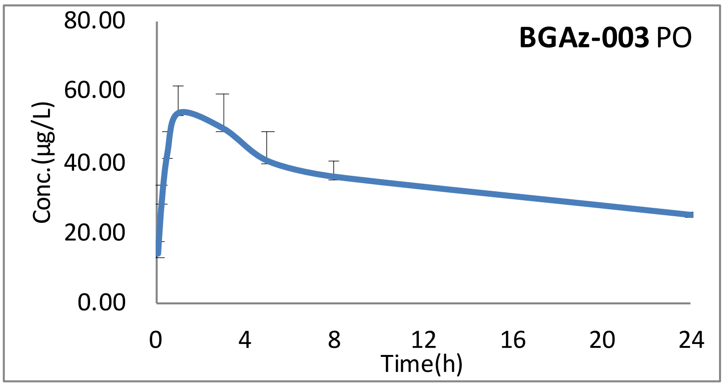

Figure 13

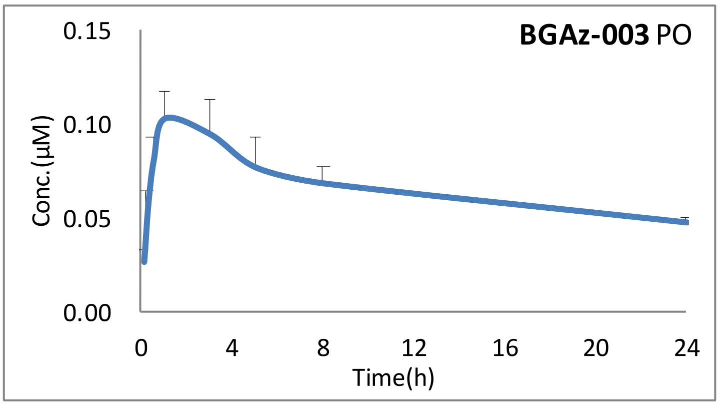

Figure 14

Table 27. Concentration of **BGAz-003** for IV (µg/L).

Time (h)	No. 1 (µg/L)	No. 2 (µg/L)	No. 3 (µg/L)	No. 4 (µg/L)	Mean (µg/L)	SD
0.033	296.33	275.38	314.33	132.14	254.54	83.14
0.167	141.72	127.01	126.87	87.93	120.88	23.05
0.5	73.46	85.29	87.09	44.34	72.54	19.75
1	50.44	67.89	58.02	39.82	54.04	11.87
3	28.70	37.64	35.03	27.22	32.15	4.99
5	16.34	27.05	21.69	18.20	20.82	4.71
8	12.34	15.90	15.08	13.80	14.28	1.56
24	7.04	15.48	6.10	5.63	8.56	4.65

Table 28. Concentration of **BGAz-003** for IV (µM).

Time (h)	No. 1 (µM)	No. 2 (µM)	No. 3 (µM)	No. 4 (µM)	Mean (µg/L)	SD
0.033	0.57	0.53	0.60	0.25	0.49	0.16
0.167	0.27	0.24	0.24	0.17	0.23	0.04
0.5	0.14	0.16	0.17	0.09	0.14	0.04
1	0.10	0.13	0.11	0.08	0.10	0.02
3	0.06	0.07	0.07	0.05	0.06	0.01
5	0.03	0.05	0.04	0.03	0.04	0.01
8	0.02	0.03	0.03	0.03	0.03	0.00
24	0.01	0.03	0.01	0.01	0.02	0.01

Table 29. Pharmacokinetic parameters of **BGAz-003** for IV.

Parameter	Unit	No. 1	No. 2	No. 3	No. 4	Mean	SD	RSD/%
**AUC(0-t)**	µg*	429.177	596.372	487.47	378.272	472.823	93.673	19.8
**AUC(0-∞)**	µg*	597.789	602.205	582.291	471.465	563.438	61.907	11
**R_AUC(t/∞)**	%	71.8	99	83.7	80.2	83.675	11.372	13.6
**AUMC(0-t)**	**µg	2751.396	4853.432	2894.712	2582.813	3270.588	1062.901	32.5
**AUMC(0-∞)**	**µg	10889.033	5027.179	6666.617	6375.757	7239.647	2535.593	35
**MRT(0-t)**		6.411	8.138	5.938	6.828	6.829	0.946	13.9
**MRT(0-∞)**		18.216	8.348	11.449	13.523	12.884	4.142	32.1
**VRT(0-t)**	^2	55.76	73.735	45.442	48.554	55.873	12.668	22.7
**VRT(0-∞)**	^2	560.765	77.842	234.705	276.043	287.339	201.286	70.1
**λz**	1/	0.041	0.173	0.063	0.06	0.084	0.06	71.4
**λz**		123	234	123	123	--	--	--
**C_last**	µg	6.95	1.007	6.009	5.58	4.887	2.649	54.2
**t1/2z**		16.814	4.013	10.935	11.573	10.834	5.255	48.5
**Tmax**		0.033	0.033	0.033	0.033	0.033	0	0
**Vz/F**	L/kg	40.587	9.616	27.098	35.422	28.181	13.567	48.1
**CLz/F**	L//kg	1.673	1.661	1.717	2.121	1.793	0.22	12.3
**Cmax**	µg	296.3267152	275.3785806	314.3285331	132.1385403	254.543	83.141	32.7
**C0**	µg	355.355	333.194	393.026	146.08	306.914	110.03	35.9

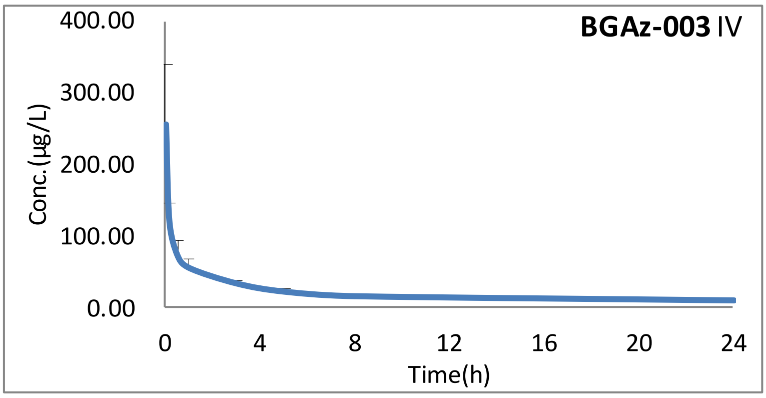

Figure 15

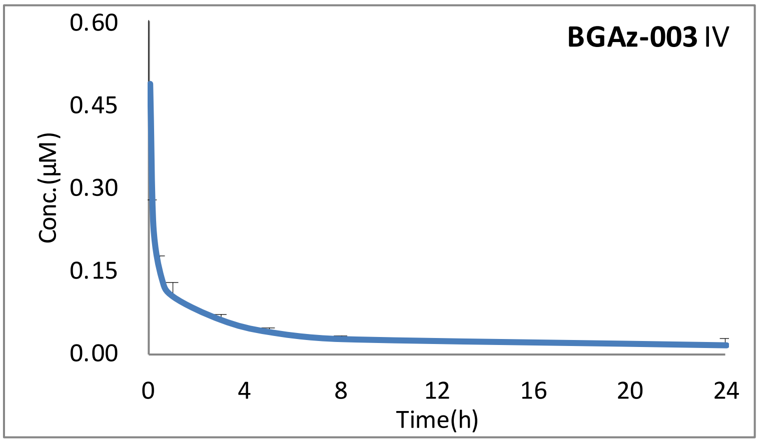

Figure 16

Table 30. Concentration of **BGAz-003** for IP (μg/L).

Time (h)	No. 1 (μg/L)	No. 2 (μg/L)	No. 3 (μg/L)	No. 4 (μg/L)	Mean (μg/L)	SD
0.083	59.71	39.66	35.20	43.85	44.61	10.67
0.25	66.33	35.29	33.21	55.35	47.55	16.02
0.5	48.44	29.41	29.71	47.54	38.78	10.65
1	31.43	22.57	22.02	31.88	26.97	5.41
3	29.94	16.81	24.92	26.99	24.66	5.63
5	16.39	14.81	/	23.47	18.22	4.61
8	20.87	12.79	13.23	18.01	16.23	3.90
24	19.42	16.41	11.70	12.44	14.99	3.60

Table 31. Concentration of **BGAz-003** for IP (μM).

Time (h)	No. 1 (μM)	No. 2 (μM)	No. 3 (μM)	No. 4 (μM)	Mean (μg/L)	SD
0.083	0.11	0.08	0.07	0.08	0.09	0.02
0.25	0.13	0.07	0.06	0.11	0.09	0.03
0.5	0.09	0.06	0.06	0.09	0.07	0.02
1	0.06	0.04	0.04	0.06	0.05	0.01
3	0.06	0.03	0.05	0.05	0.05	0.01
5	0.03	0.03	/	0.05	0.04	0.01
8	0.04	0.02	0.03	0.03	0.03	0.01
24	0.04	0.03	0.02	0.02	0.03	0.01

Table 32. Pharmacokinetic parameters of **BGAz-003** for IP.

Parameter	Unit	No. 1	No. 2	No. 3	No. 4	Mean	SD	RSD/%
**AUC(0-t)**	µg*	533.268	374.944	369.663	457.978	433.963	77.58	17.9
**AUC(0-∞)**	µg*	1654.392	473.609	750.59	518.391	849.246	550.322	64.8
**R_AUC(t/∞)**	%	32.2	79.2	49.2	88.3	62.225	26.072	41.9
**AUMC(0-t)**	**µg	5752.205	4443.772	3654.106	4264.861	4528.736	882.931	19.5
**AUMC(0-∞)**	**µg	99849.438	8631.896	25094.826	6456.42	35008.145	44021.111	125.7
**MRT(0-t)**		10.787	11.852	9.885	9.312	10.459	1.11	10.6
**MRT(0-∞)**		60.354	18.226	33.433	12.455	31.117	21.406	68.8
**VRT(0-t)**	^2	78.281	85.251	74.376	66.726	76.159	7.731	10.2
**VRT(0-∞)**	^2	3627.883	292.778	1103.766	151.371	1293.95	1611.546	124.5
**λz**	1/	0.017	0.054	0.031	0.081	0.046	0.028	60.9
**λz**		124	234	134	234	--	--	--
**C_last**	µg	18.707	5.348	11.799	4.921	10.194	6.489	63.7
**t1/2z**		41.532	12.784	22.374	8.508	21.3	14.682	68.9
**Tmax**		0.25	0.083	0.083	0.25	0.167	0.096	57.5
**Vz/F**	L/kg	36.225	38.952	43.014	23.682	35.468	8.338	23.5
**CLz/F**	L//kg	0.604	2.111	1.332	1.929	1.494	0.68	45.5
**Cmax**	µg	66.33443614	39.65805608	35.20115477	55.3538559	49.137	14.358	29.2

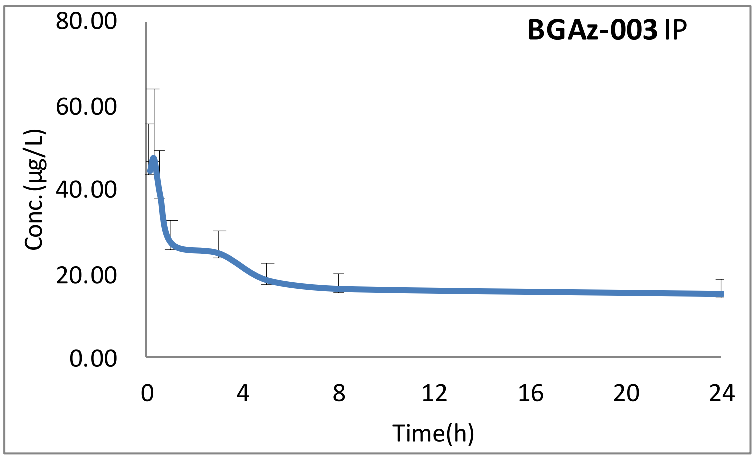

Figure 17

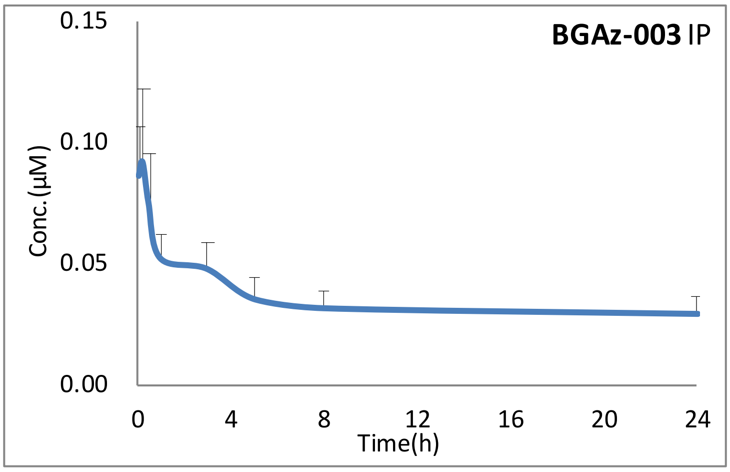

Figure 18

6.4 *Results for* BGAz-004

Table 33. Concentration of **BGAz-004** for PO (μg/L).

Time (h)	No. 1 (μg/L)	No. 2 (μg/L)	No. 3 (μg/L)	No. 4 (μg/L)	Mean (μg/L)	SD
0.0833	18.93	20.62	17.03	12.67	17.31	3.42
0.25	47.52	41.91	46.72	34.36	42.63	6.05
0.5	71.61	60.84	70.38	57.60	65.11	6.94
1	58.47	88.31	96.10	94.58	84.36	17.59
3	53.58	79.62	60.20	80.36	68.44	13.61
5	55.17	62.93	death	71.81	63.31	8.33
8	53.73	69.78	death	57.99	60.50	8.32
24	23.54	17.68	death	25.27	22.16	3.98

Table 34. Concentration of **BGAz-004** for PO (μM).

Time (h)	No. 1 (μM)	No. 2 (μM)	No. 3 (μM)	No. 4 (μM)	Mean (μg/L)	SD
0.083	0.04	0.04	0.03	0.02	0.03	0.01
0.25	0.09	0.08	0.09	0.06	0.08	0.01
0.5	0.13	0.11	0.13	0.11	0.12	0.01
1	0.11	0.16	0.18	0.18	0.16	0.03
3	0.10	0.15	0.11	0.15	0.13	0.03
5	0.10	0.12	death	0.13	0.12	0.02
8	0.10	0.13	death	0.11	0.11	0.02
24	0.04	0.03	death	0.05	0.04	0.01

Table 35. Pharmacokinetic parameters of **BGAz-004** for PO.

Parameter	Unit	No. 1	No. 2	No. 3	No. 4	Mean	SD	RSD/%
**AUC(0-t)**	μg*	1056.005	1265.482	218.572	1241.851	945.478	493.575	52.2
**AUC(0-∞)**	μg*	1627.205	1517.952	781.063	1700.962	1406.796	423.875	30.1
**R_AUC(t/∞)**	%	64.9	83.4	28	73	62.325	24.104	38.7
**AUMC(0-t)**	**μg	9702.775	10087.367	316.518	10769.797	7719.114	4954.749	64.2
**AUMC(0-∞)**	**μg	37195.654	19768.43	7049.909	30130.148	23536.035	13115.572	55.7
**MRT(0-t)**		9.188	7.971	1.448	8.672	6.82	3.616	53
**MRT(0-∞)**		22.859	13.023	9.026	17.714	15.656	5.972	38.1
**VRT(0-t)**	^2	53.474	38.713	0.964	51.831	36.246	24.431	67.4
**VRT(0-∞)**	^2	584.618	194.427	80.537	348.053	301.909	218.03	72.2
**λz**	1/	0.041	0.07	0.111	0.055	0.069	0.03	43.5
**λz**		134	134	123	134	--	--	--
**C_last**	μg	23.67	17.6	62.703	25.269	32.311	20.529	63.5
**t1/2z**		16.723	9.941	6.217	12.591	11.368	4.425	38.9
**Tmax**		0.5	1	1	1	0.875	0.25	28.6
**Vz/F**	L/kg	74.151	47.252	57.426	53.409	58.06	11.515	19.8
**CLz/F**	L//kg	3.073	3.294	6.402	2.94	3.927	1.656	42.2
**Cmax**	μg	71.61139092	88.30540751	96.09674961	94.58256818	87.649	11.211	12.8

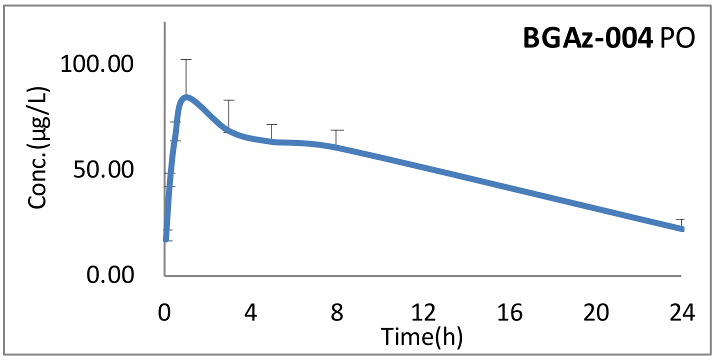

Figure 19

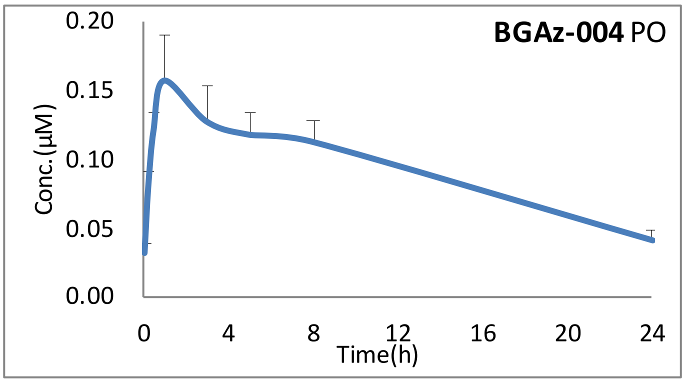

Figure 20

Table 36. Concentration of **BGAz-004** for IV (μg/L).

Time (h)	No. 1 (μg/L)	No. 2 (μg/L)	No. 3 (μg/L)	No. 4 (μg/L)	Mean (μg/L)	SD
0.033	312.46	299.55	348.67	152.89	278.39	86.21
0.167	154.96	167.65	170.71	97.46	147.69	34.18
0.5	76.49	105.49	101.34	55.23	84.64	23.42
1	69.82	153.90	87.34	47.23	89.57	45.92
3	34.42	53.91	43.69	30.09	40.53	10.57
5	20.54	44.93	26.66	21.15	28.32	11.41
8	13.46	40.83	17.31	14.90	21.62	12.90
24	2.96	50.95	3.09	3.23	15.06	23.93

Table 37. Concentration of **BGAz-004** for IV (μM).

Time (h)	No. 1 (μM)	No. 2 (μM)	No. 3 (μM)	No. 4 (μM)	Mean (μg/L)	SD
0.033	0.58	0.55	0.65	0.28	0.52	0.16
0.167	0.29	0.31	0.32	0.18	0.27	0.06
0.5	0.14	0.20	0.19	0.10	0.16	0.04
1	0.13	0.29	0.16	0.09	0.17	0.09
3	0.06	0.10	0.08	0.06	0.08	0.02
5	0.04	0.08	0.05	0.04	0.05	0.02
8	0.02	0.08	0.03	0.03	0.04	0.02
24	0.01	0.09	0.01	0.01	0.03	0.04

Table 38. Pharmacokinetic parameters of **BGAz-004** for IV.

Parameter	Unit	No. 1	No. 2	No. 3	No. 4	Mean	SD	RSD/%
**AUC(0-t)**	μg*	459.31	1321.748	570.433	400.795	688.072	428.27	62.2
**AUC(0-∞)**	μg*	488.727	1637.476	597.917	433.6	789.43	569.472	72.1
**R_AUC(t/∞)**	%	94	80.7	95.4	92.4	90.625	6.729	7.4
**AUMC(0-t)**	**μg	2164.698	13991.502	2642.241	2271.474	5267.479	5819.615	110.5
**AUMC(0-∞)**	**μg	3166.206	27445.659	3548.323	3394.024	9388.553	12039.094	128.2
**MRT(0-t)**		4.713	10.586	4.632	5.667	6.4	2.83	44.2
**MRT(0-∞)**		6.478	16.761	5.934	7.828	9.25	5.07	54.8
**VRT(0-t)**	^2	29.652	87.357	26.472	32.386	43.967	29.028	66
**VRT(0-∞)**	^2	82.613	296.959	64.162	94.844	134.645	108.942	80.9
**λz**	1/	0.1	0.054	0.112	0.098	0.091	0.025	27.5
**λz**		123	234	123	123	--	--	--
**C_last**	μg	2.928	16.963	3.065	3.21	6.542	6.949	106.2
**t1/2z**		6.962	12.899	6.215	7.081	8.289	3.097	37.4
**Tmax**		0.033333333	0.033333333	0.033333333	0.033333333	0.033	0	0
**Vz/F**	L/kg	20.555	11.367	14.998	23.567	17.622	5.476	31.1
**CLz/F**	L//kg	2.046	0.611	1.672	2.306	1.659	0.745	44.9
**Cmax**	μg	312.4616133	299.5545268	348.6708335	152.8928136	278.395	86.212	31
**C0**	μg	372.341	346.335	416.829	171.112	326.654	107.703	33

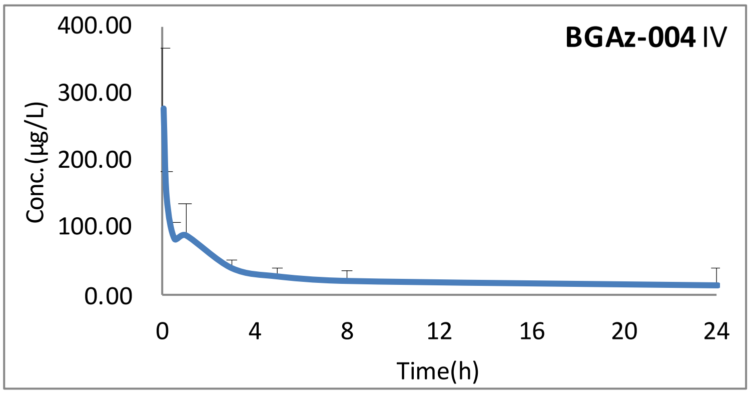

Figure 21

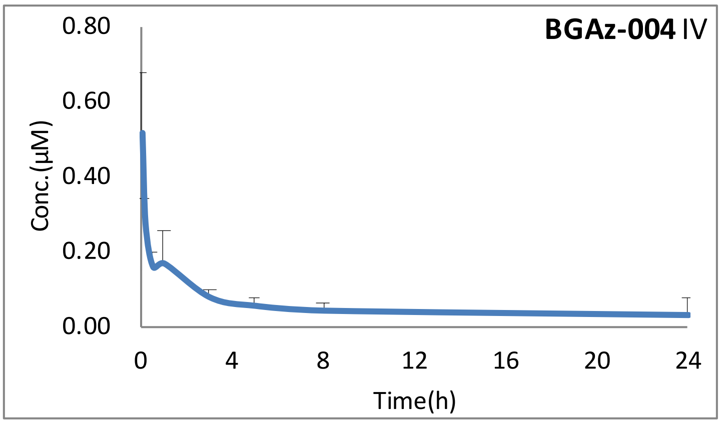

Figure 22

Table 39. Concentration of **BGAz-004** for IP (µg/L).

Time (h)	No. 1 (µg/L)	No. 2 (µg/L)	No. 3 (µg/L)	No. 4 (µg/L)	Mean (µg/L)	SD
0.083	65.88	48.41	45.50	61.29	55.27	9.85
0.25	71.52	46.22	45.43	77.07	60.06	16.59
0.5	52.15	41.72	47.09	60.20	50.29	7.86
1	37.03	38.73	37.78	44.09	39.41	3.20
3	31.92	28.16	33.48	30.35	30.98	2.27
5	21.17	27.36	/	24.54	24.36	3.10
8	24.39	22.52	23.22	17.09	21.81	3.24
24	7.45	8.48	7.51	5.18	7.15	1.40

Table 40. Concentration of **BGAz-004** for IP (µM).

Time (h)	No. 1 (µM)	No. 2 (µM)	No. 3 (µM)	No. 4 (µM)	Mean (µg/L)	SD
0.083	0.12	0.09	0.08	0.11	0.10	0.02
0.25	0.13	0.09	0.08	0.14	0.11	0.03
0.5	0.10	0.08	0.09	0.11	0.09	0.01
1	0.07	0.07	0.07	0.08	0.07	0.01
3	0.06	0.05	0.06	0.06	0.06	0.00
5	0.04	0.05	/	0.05	0.05	0.01
8	0.05	0.04	0.04	0.03	0.04	0.01
24	0.01	0.02	0.01	0.01	0.01	0.00

Table 41. Pharmacokinetic parameters of **BGAz-004** for IP.

Parameter	Unit	No. 1	No. 2	No. 3	No. 4	Mean	SD	RSD/%
**AUC(0-t)**	µg*	497.01	486.239	501.186	427.24	477.919	34.368	7.2
**AUC(0-∞)**	µg*	603.895	624.016	608.203	489.32	581.359	61.966	10.7
**R_AUC(t/∞)**	%	82.3	77.9	82.4	87.3	82.475	3.84	4.7
**AUMC(0-t)**	**µg	3800.137	3909.467	3803.862	2853.255	3591.68	494.886	13.8
**AUMC(0-∞)**	**µg	7882.021	9457.334	7895.112	5088.798	7580.816	1818.514	24
**MRT(0-t)**		7.646	8.04	7.59	6.678	7.489	0.576	7.7
**MRT(0-∞)**		13.052	15.156	12.981	10.4	12.897	1.947	15.1
**VRT(0-t)**	^2	44.353	48.522	44.823	40.606	44.576	3.237	7.3
**VRT(0-∞)**	^2	208.032	274.912	208.691	149.064	210.175	51.421	24.5
**λz**	1/	0.07	0.061	0.07	0.083	0.071	0.009	12.7
**λz**		124	123	124	134	--	--	--
**C_last**	µg	7.533	8.47	7.521	5.169	7.173	1.408	19.6
**t1/2z**		9.833	11.273	9.861	8.323	9.823	1.205	12.3
**Tmax**		0.25	0.083333333	0.5	0.25	0.271	0.172	63.5
**Vz/F**	L/kg	23.497	26.068	23.397	24.545	24.377	1.241	5.1
**CLz/F**	L//kg	1.656	1.603	1.644	2.044	1.737	0.206	11.9
**Cmax**	µg	71.52326579	48.41040301	47.08688711	77.06535763	61.021	15.502	25.4

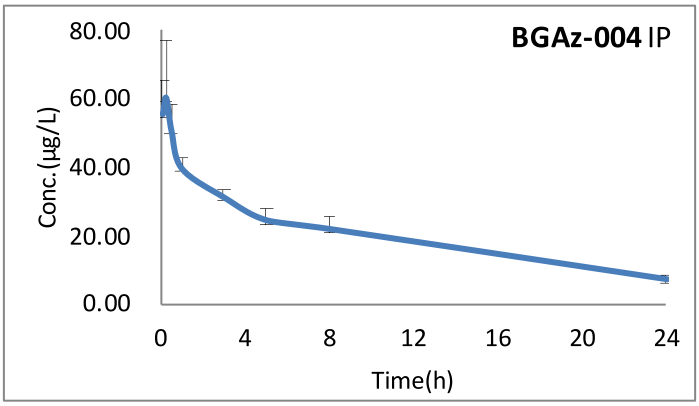

Figure 23

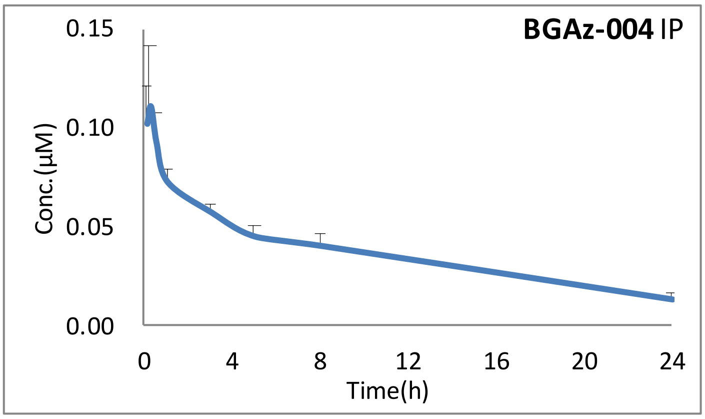

Figure 24

6.5 Results for BGAz-005

Table 42. Concentration of **BGAz-005** for PO (µg/L).

Time (h)	No. 1 (µg/L)	No. 2 (µg/L)	No. 3 (µg/L)	No. 4 (µg/L)	Mean (µg/L)	SD
0.083	11.85	9.55	3.03	3.87	7.08	4.30
0.25	15.24	8.96	11.40	9.61	11.30	2.82
0.5	26.93	17.11	19.69	18.05	20.45	4.45
1	24.17	35.42	31.56	39.03	32.55	6.36
3	25.26	46.01	46.93	38.34	39.13	10.02
5	22.52	43.17	death	47.25	37.65	13.26
8	28.02	50.31	death	42.42	40.25	11.30
24	18.13	31.32	death	23.47	24.31	6.63

Table 43. Concentration of **BGAz-005** for PO (µM).

Time (h)	No. 1 (µM)	No. 2 (µM)	No. 3 (µM)	No. 4 (µM)	Mean (µg/L)	SD
0.083	0.03	0.02	0.01	0.01	0.02	0.01
0.25	0.04	0.02	0.03	0.02	0.03	0.01
0.5	0.06	0.04	0.05	0.04	0.05	0.01
1	0.06	0.08	0.07	0.09	0.07	0.01
3	0.06	0.11	0.11	0.09	0.09	0.02
5	0.05	0.10	death	0.11	0.09	0.03
8	0.06	0.12	death	0.10	0.09	0.03
24	0.04	0.07	death	0.05	0.06	0.02

Table 44. Pharmacokinetic parameters of **BGAz-005** for PO.

Parameter	Unit	No. 1	No. 2	No. 3	No. 4	Mean	SD	RSD/%
**AUC(0-t)**	µg*	563.054	982.143	96.511	843.549	621.314	390.896	62.9
**AUC(0-∞)**	µg*	1848.857	2744.193	96.511	1479.962	1542.381	1100.411	71.3
**R_AUC(t/∞)**	%	30.5	35.8	100	57	55.825	31.597	56.6
**AUMC(0-t)**	**µg	6079.921	10699.775	184.544	8603.037	6391.819	4548.828	71.2
**AUMC(0-∞)**	**µg	128529.145	152271.287	184.544	41130.722	80528.925	71783.066	89.1
**MRT(0-t)**		10.798	10.894	1.912	10.199	8.451	4.37	51.7
**MRT(0-∞)**		69.518	55.489	1.912	27.792	38.678	30.022	77.6
**VRT(0-t)**	^2	65.399	63.023	1.149	59.142	47.178	30.794	65.3
**VRT(0-∞)**	^2	5058.543	3169.501	1.149	760.034	2247.307	2310.173	102.8
**λz**	1/	0.014	0.018	0	0.037	0.017	0.015	88.2
**λz**		134	134		123	--	--	--
**C_last**	µg	18.051	31.273	1	23.474	18.45	12.837	69.6
**t1/2z**		49.364	39.047		18.788	35.733	15.555	43.5
**Tmax**		8	8	3	5	6	2.449	40.8
**Vz/F**	L/kg	192.637	102.662		91.593	128.964	55.419	43
**CLz/F**	L//kg	2.704	1.822	51.808	3.378	14.928	24.595	164.8
**Cmax**	µg	28.02378861	50.30584346	46.92633289	47.25369321	43.127	10.183	23.6

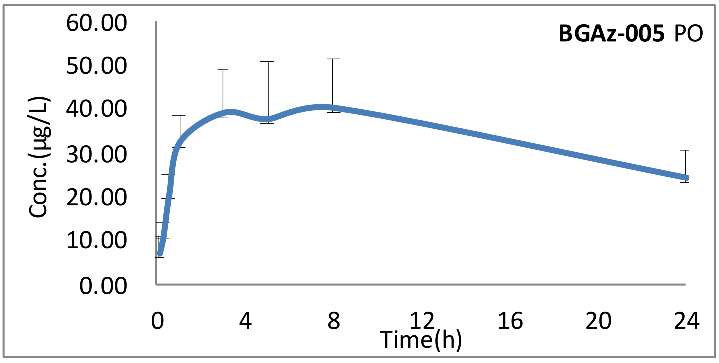

Figure 25

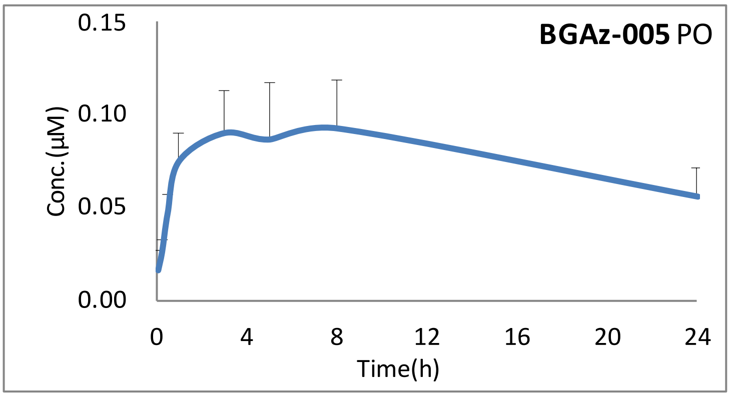

Figure 26

Table 45. Concentration of **BGAz-005** for IV (μg/L).

Time (h)	No. 1 (μg/L)	No. 2 (μg/L)	No. 3 (μg/L)	No. 4 (μg/L)	Mean (μg/L)	SD
0.033	333.20	356.84	403.52	188.39	320.49	92.78
0.167	144.37	160.92	156.16	119.07	145.13	18.71
0.5	63.00	81.74	78.03	78.03	75.20	8.32
1	38.37	56.47	52.87	52.87	50.15	8.03
3	20.15	28.18	26.64	26.64	25.40	3.58
5	12.02	21.68	15.37	15.37	16.11	4.03
8	8.24	12.87	10.08	10.08	10.32	1.91
24	3.70	11.57	2.76	2.76	5.20	4.27

Table 46. Concentration of **BGAz-005** for IV (μM).

Time (h)	No. 1 (μM)	No. 2 (μM)	No. 3 (μM)	No. 4 (μM)	Mean (μg/L)	SD
0.033	0.77	0.82	0.93	0.43	0.74	0.21
0.167	0.33	0.37	0.36	0.27	0.33	0.04
0.5	0.15	0.19	0.18	0.18	0.17	0.02
1	0.09	0.13	0.12	0.12	0.12	0.02
3	0.05	0.06	0.06	0.06	0.06	0.01
5	0.03	0.05	0.04	0.04	0.04	0.01
8	0.02	0.03	0.02	0.02	0.02	0.00
24	0.01	0.03	0.01	0.01	0.01	0.01

Table 47. Pharmacokinetic parameters of **BGAz-005** for IV.

Parameter	Unit	No. 1	No. 2	No. 3	No. 4	Mean	SD	RSD/%
**AUC(0-t)**	μg*	320.753	504.628	386.76	355.171	391.828	79.885	20.4
**AUC(0-∞)**	μg*	383.317	511.214	417.987	386.399	424.729	59.747	14.1
**R_AUC(t/∞)**	%	83.7	98.7	92.5	91.9	91.7	6.156	6.7
**AUMC(0-t)**	**μg	1675.205	3735.11	1737.886	1735.846	2221.012	1009.818	45.5
**AUMC(0-∞)**	**μg	4253.915	3934.839	2845.377	2843.337	3469.367	733.361	21.1
**MRT(0-t)**		5.223	7.402	4.493	4.887	5.501	1.302	23.7
**MRT(0-∞)**		11.098	7.697	6.807	7.359	8.24	1.94	23.5
**VRT(0-t)**	^2	45.113	70.24	32.475	33.463	45.323	17.575	38.8
**VRT(0-∞)**	^2	263.087	76.534	106.18	110.84	139.16	84.003	60.4
**λz**	1/	0.058	0.158	0.087	0.087	0.098	0.043	43.9
**λz**		123	234	123	123	--	--	--
**C_last**	μg	3.634	1.041	2.724	2.724	2.531	1.082	42.7
**t1/2z**		11.932	4.385	7.945	7.945	8.052	3.084	38.3
**Tmax**		0.033333333	0.033333333	0.033333333	0.033333333	0.033	0	0
**Vz/F**	L/kg	44.917	12.377	27.43	29.672	28.599	13.316	46.6
**CLz/F**	L//kg	2.609	1.956	2.392	2.588	2.386	0.303	12.7
**Cmax**	μg	333.1965586	356.8355463	403.522902	188.3926665	320.487	92.784	29
**C0**	μg	410.685	435.447	511.611	211.29	392.258	128.062	32.6

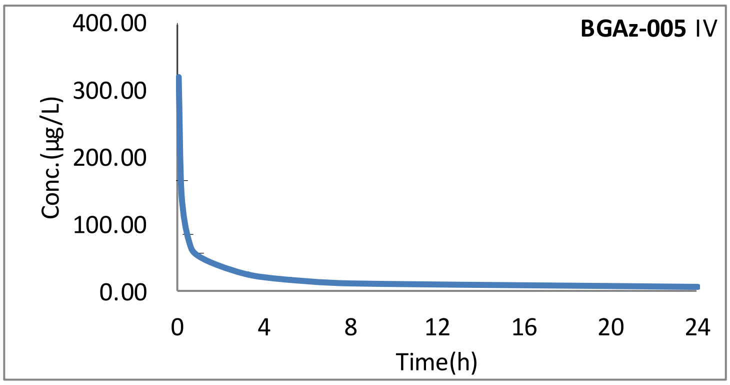

Figure 27

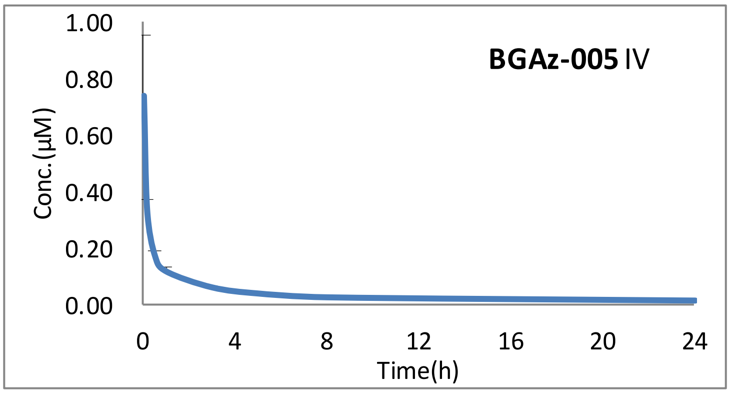

Figure 28

Table 48. Concentration of **BGAz-005** for IP (μg/L).

Time (h)	No. 1 (μg/L)	No. 2 (μg/L)	No. 3 (μg/L)	No. 4 (μg/L)	Mean (μg/L)	SD
0.083	20.53	14.50	19.62	53.21	26.96	17.70
0.25	39.41	20.97	31.29	27.59	29.82	7.69
0.5	42.67	28.16	40.95	33.32	36.27	6.76
1	43.88	29.55	39.49	46.14	39.76	7.35
3	34.52	22.40	39.02	37.48	33.35	7.54
5	18.30	19.40	/	26.18	21.29	4.27
8	16.77	15.15	12.75	17.08	15.44	1.03
24	5.58	6.67	5.89	3.64	5.44	1.53

Table 49. Concentration of **BGAz-005** for IP (μM).

Time (h)	No. 1 (μM)	No. 2 (μM)	No. 3 (μM)	No. 4 (μM)	Mean (μg/L)	SD
0.083	0.05	0.03	0.05	0.12	0.06	0.04
0.25	0.09	0.05	0.07	0.06	0.07	0.02
0.5	0.10	0.06	0.09	0.08	0.08	0.02
1	0.10	0.07	0.09	0.11	0.09	0.02
3	0.08	0.05	0.09	0.09	0.08	0.02
5	0.04	0.04	/	0.06	0.05	0.01
8	0.04	0.03	0.03	0.04	0.04	0.00
24	0.01	0.02	0.01	0.01	0.01	0.00

Table 50. Pharmacokinetic parameters of **BGAz-005** for IP.

Parameter	Unit	No. 1	No. 2	No. 3	No. 4	Mean	SD	RSD/%
**AUC(0-t)**	μg*	400.345	344.276	391.262	414.319	387.551	30.368	7.8
**AUC(0-∞)**	μg*	487.462	460.9	460.52	447.721	464.151	16.704	3.6
**R_AUC(t/∞)**	%	82.1	74.7	85	92.5	83.575	7.363	8.8
**AUMC(0-t)**	**μg	2846.397	2852.293	2670.84	2614.567	2746.024	121.52	4.4
**AUMC(0-∞)**	**μg	6285.492	7693.972	5141.16	3725.026	5711.413	1686.32	29.5
**MRT(0-t)**		7.11	8.285	6.826	6.311	7.133	0.836	11.7
**MRT(0-∞)**		12.894	16.693	11.164	8.32	12.268	3.501	28.5
**VRT(0-t)**	^2	43.674	52.184	47.976	31.434	43.817	8.957	20.4
**VRT(0-∞)**	^2	232.444	325.316	167.527	85.551	202.71	101.457	50.1
**λz**	1/	0.065	0.057	0.086	0.108	0.079	0.023	29.1
**λz**		123	134	134	134	--	--	--
**C_last**	μg	5.629	6.659	5.936	3.613	5.459	1.304	23.9
**t1/2z**		10.726	12.138	8.086	6.407	9.339	2.577	27.6
**Tmax**		1	1	0.5	0.083333333	0.646	0.443	68.6
**Vz/F**	L/kg	31.75	38.002	25.337	20.649	28.935	7.566	26.1
**CLz/F**	L//kg	2.051	2.17	2.171	2.234	2.157	0.076	3.5
**Cmax**	μg	43.87528447	29.55363799	40.94640377	53.21341977	41.897	9.751	23.3

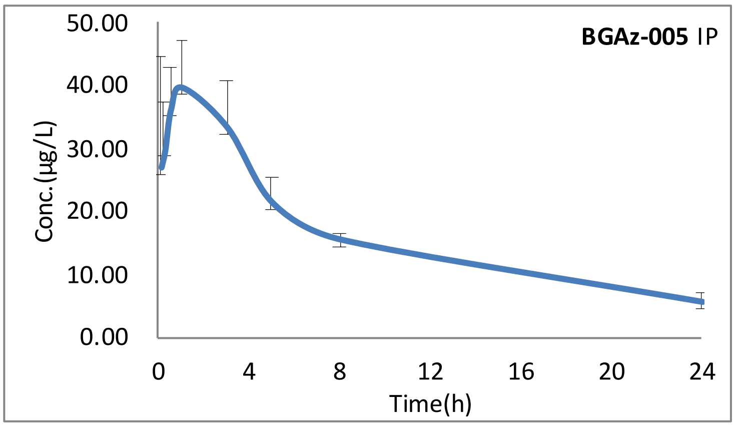

Figure 29

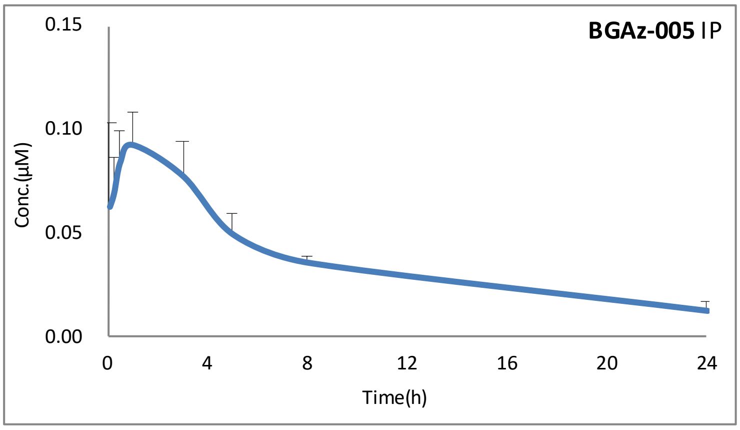

Figure 30

Report on PK results of BGAz-004 by multiple oral dosing

2020.04.08

7 Materials and instrument combined dosing

An MS2 Turnover type oscillator was purchased from IKA Work's Guangzhou (China), and a 5415R Chromatographic analyses were conducted using an Agilent 1290 Infinity II high performance liquid chromatography and Agilent Technologies 6470Triple Quad LC/MS. Propranolol (≥90% in purity, internal standard, IS) was purchased from the Sigma Chemical Co. (China). Formic acid (HPLC grade) and methanol (HPLC grade) were purchased from DIKMA Co. (China). All chemicals and solvents were analytical grade. Water was purified using a Millipore (AK, USA) laboratory ultra-pure water system (0.2 μm filter).

8 Animal

KM mice, weighing 25–35 g (Beijing Vital River Laboratory Animal Technology Co., Ltd., China) were utilised for the studies. The protocols were approved by the Animal Care and Use Committee, GIBH. Animals were maintained on standard animal chow and water *ad libitum*, in a climate-controlled room (23±1 °C 30–70% relative humidity, a minimum of ten exchanges of room air per hour and a 12 h light/dark cycle) for one week prior to experiments.

9 Pharmacokinetic studies

Compound **BGAz-004** were dissolved in the solution containing DMSO (2%), ethanol (4%), Cremophor EL (4%) and ddH_2O (90%). The mice were given solution with 30 mg/kg by gastric gavage for 3 days. Whole blood samples (100 μL) were obtained from the orbital venous plexus at the following time points after the third day dosing: 5, 15, 30 min and 1, 3, 5, 8, 24 hour. Whole blood samples were collected in heparinised tubes. The plasma fraction was immediately separated by centrifugation. The mice were humanely euthanasia by carbon dioxide 24 hours after experiment without pain.

10 Plasma sample analysis

10.1 Standard curve sample preparation

The compounds were dissolved in DMSO (2mg/mL) and diluted with to series concentration (methanol:H_2O, 1:1, 10 μL) and blank plasma (50 μL) were added to 1.5 mL tubes and vortexed for 3 min, then acetonitrile-containing internal standard (150 μL) was added and vortexed for a further 5 min, and subsequently spun in a centrifuge at 13000 ×*g* for 40 min at 4 °C, the final concentrations were as follow: 5, 10, 20, 50, 100, 200, 500 ng/mL.

10.2 Plasma and preparation

Plasma samples were prepared using a protein precipitation method. Solution (10 μL, methanol H_2O, 1:1) and plasma samples (50 μL) were added to 1.5 mL tubes and vortex for 3 min, then acetonitrile-containing internal standard (150 μL) was added and vortex for 5 min, and subsequently spun in a centrifuge at 13000 ×*g* for 40 min at 4 °C.

11 LC/MS/MS analysis

After centrifugation, supernatant (100 μL) was transfer to 96 well plates and analysed by LC-MS/MS using an Agilent 1290 Infinity II HPLC and an Agilent Technologies 6470Triple Quad LC/MS.

12 Results

Table 51. Pharmacokinetic parameters of multiple oral dosing for **BGAz-004**.

**BGAz-004**	
**Dosing method**	Multiple PO
**Times**	4
**Animal Number KM mice**	♂4
**Dose level mg/kg**	30
**AUC(0-∞) μg*h**	5420.987
**$T_{1/2}$ (h)**	8.072
**T_{max} (h)**	1
**C_{max}(μg/L)**	363.85

Table 52. Concentration of **BGAz -004** for PO (μg/L).

Time (h)	No. 1 (μg/L)	No. 2 (μg/L)	No. 3 (μg/L)	No. 4 (μg/L)	Mean (μg/L)	SD (μg/L)
0.083	96.71	101.68	138.93	123.99	115.33	19.71
0.25	176.35	170.28	252.32	180.64	194.90	38.52
0.5	236.08	351.51	392.71	293.87	318.54	68.30
1	265.84	421.49	413.82	354.25	363.85	71.92
3	245.29	393.56	354.44	290.26	320.88	65.98
5	195.41	337.53	302.89	236.49	268.08	64.07
8	155.47	404.46	251.36	161.49	243.20	116.11
24	66.06	11.72	82.40	94.62	63.70	36.57

Table 53. Concentration of **BGAz -004** for PO (μM).

Time (h)	No. 1 (μM)	No. 2 (μM)	No. 3 (μM)	No. 4 (μM)	Mean (μM)	SD (μM)
0.083	0.179	0.188	0.257	0.230	0.214	0.036
0.25	0.327	0.315	0.467	0.335	0.361	0.071
0.5	0.437	0.651	0.727	0.544	0.590	0.127
1	0.492	0.781	0.766	0.656	0.674	0.133
3	0.454	0.729	0.656	0.538	0.594	0.122
5	0.362	0.625	0.561	0.438	0.496	0.119
8	0.288	0.749	0.466	0.299	0.450	0.215
24	0.122	0.022	0.153	0.175	0.118	0.068

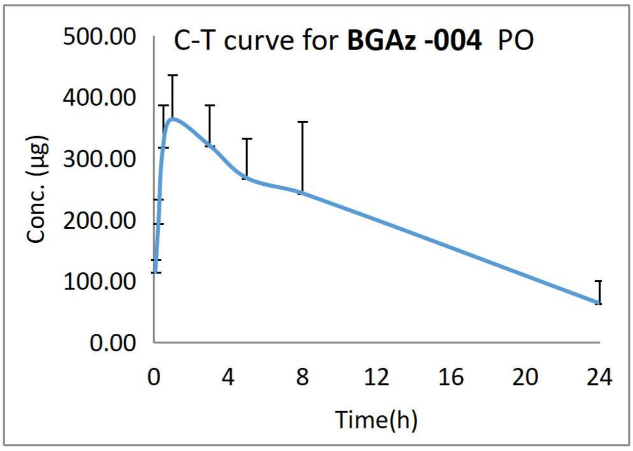

Figure 31

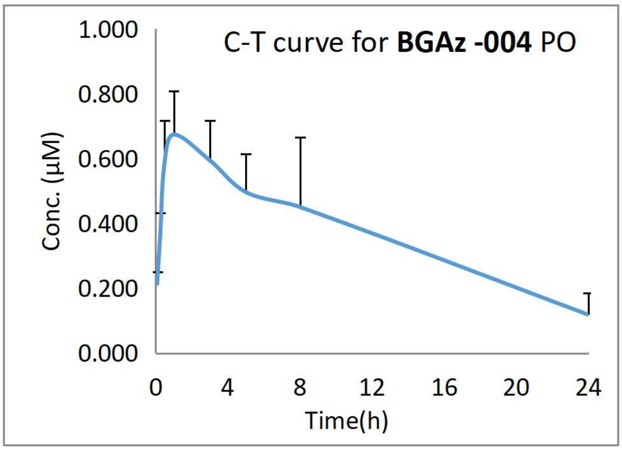

Figure 32

Table 54. Pharmacokinetic parameters of **BGAz-004** for PO.

Parameter	Unit	No. 1	No.2	No.3	No.4	Mean	SD	RSD/%
**AUC(0-t)**	μg*h	3454.221	6273.905	5247.724	4069.043	4761.223	1253.297	26.3
**AUC(0-∞)**	μg*h	4629.924	6343.027	6433.361	4277.637	5420.987	1126.66	20.8
**R_AUC(t/∞)**	%	74.6	98.9	81.6	95.1	87.55	11.386	13
**AUMC(0-t)**	h*h*μg	28799.836	40171.862	41441.784	35646.495	36514.994	5713.256	15.6
**AUMC(0-∞)**	h*h*μg	78051.986	42234.646	86948.542	42419.904	62413.77	23476.659	37.6
**MRT(0-t)**	h	8.338	6.403	7.897	8.76	7.85	1.027	13.1
**MRT(0-∞)**	h	16.858	6.658	13.515	9.917	11.737	4.416	37.6
**VRT(0-t)**	h^2	50.888	11.386	44.039	59.569	41.471	21.039	50.7
**VRT(0-∞)**	h^2	332.554	17.556	213.745	86.244	162.525	139.485	85.8
**λz**	1/h	0.056	0.171	0.07	0.118	0.104	0.052	50
**λz**		123	134	124	234	--	--	--
**C_last**	μg	65.712	11.83	82.441	24.622	46.151	33.372	72.3
**t1/2z**	h	12.399	4.049	9.967	5.871	8.072	3.801	47.1
**Tmax**	h	1	1	1	1	1	0	0
**Vz/F**	L/kg	115.931	27.634	67.065	59.414	67.511	36.517	54.1
**CLz/F**	L/h/kg	6.48	4.73	4.663	7.013	5.722	1.204	21
**Cmax**	μg	265.8381411	421.4879417	413.8210272	354.252085	363.85	71.921	19.8

Report on PK results of BGAz-005 by multiple oral dosing

2020.05.22

13 Materials and instrument combined dosing

An MS2 Turnover type oscillator was purchased from IKA Work's Guangzhou (China), and a 5415R Chromatographic analyses were conducted using an Agilent 1290 Infinity II high performance liquid chromatography and Agilent Technologies 6470Triple Quad LC/MS. Propranolol (≥90% in purity, internal standard, IS) was purchased from the Sigma Chemical Co. (China). Formic acid (HPLC grade) and methanol (HPLC grade) were purchased from DIKMA Co. (China). All chemicals and solvents were analytical grade. Water was purified using a Millipore (AK, USA) laboratory ultra-pure water system (0.2 μm filter).

14 Animal

KM mice, weighing 25–35 g (Beijing Vital River Laboratory Animal Technology Co., Ltd., China) were utilised for the studies. The protocols were approved by the Animal Care and Use Committee, GIBH. Animals were maintained on standard animal chow and water *ad libitum*, in a climate-controlled room (23±1 °C 30–70% relative humidity, a minimum of ten exchanges of room air per hour and a 12 h light/dark cycle) for one week prior to experiments.

15 Pharmacokinetic studies

Compound **BGAz-005** were dissolved in the solution containing DMSO (2%), ethanol (4%), Cremophor EL (4%) and ddH$_2$O (90%). The mice were given solution with 30 mg/kg by gastric gavage for 3 days. Whole blood samples (100 μL) were obtained from the orbital venous plexus at the following time points after the third day dosing: 5, 15, 30 min and 1, 3, 5, 8, 24 hour. Whole blood samples were collected in heparinised tubes. The plasma fraction was immediately separated by centrifugation. The mice were humanely euthanasia by carbon dioxide 24 hours after experiment without pain.

16 Plasma sample analysis

16.1 Standard curve sample preparation

The compounds were dissolved in DMSO (2mg/mL) and diluted with to series concentration (methanol:H$_2$O, 1:1, 10 μL) and blank plasma (50 μL) were added to 1.5 mL tubes and vortexed for 3 min, then acetonitrile-containing internal standard (150 μL) was added and vortexed for a further 5 min, and subsequently spun in a centrifuge at 13000 ×*g* for 40 min at 4 °C, the final concentrations were as follow: 5, 10, 20, 50, 100, 200, 500, 2000 ng/mL.

16.2 Plasma and preparation

Plasma samples were prepared using a protein precipitation method. Solution (10 μL, methanol H$_2$O, 1:1) and plasma samples (50 μL) were added to 1.5 mL tubes and vortex for 3 min, then acetonitrile-containing internal standard (150 μL) was added and vortex for 5 min, and subsequently spun in a centrifuge at 13000 ×*g* for 40 min at 4 °C.

17 LC/MS/MS analysis

After centrifugation, supernatant (100 μL) was transfer to 96 well plates and analysed by LC-MS/MS using an Agilent 1290 Infinity II HPLC and an Agilent Technologies 6470Triple Quad LC/MS.

Table 55. Pharmacokinetic parameters of multiple oral dosing for **BGAz-005**.

BGAz-005	
**Dosing method**	Multiple PO
**Times**	3
**Animal Number KM mice**	♂4
**Dose level mg/kg**	30
**AUC(0-∞) μg/L*h**	82247.729
**$T_{1/2}$ (h)**	80.437
**T_{max} (h)**	0.875
**C_{max}(μg/L)**	1712.54

Table 56. Concentration of **BGAz-005** for PO (μg/L).

Time (h)	No. 1 (μg/L)	No. 2 (μg/L)	No. 3 (μg/L)	No. 4 (μg/L)	Mean (μg/L)	SD (μg/L)
0.083	513.99	1123.99	826.27	968.72	858.24	259.72
0.25	656.54	935.75	1196.01	1887.19	1168.87	527.11
0.5	702.36	1539.13	1591.63	2314.39	1536.88	659.28
1	979.43	1928.73	1276.73	2350.26	1633.79	620.75
3	535.60	1377.36	631.57	875.45	854.99	376.48
5	614.08	831.63	654.72	754.98	713.85	98.34
8	507.07	594.94	570.05	759.27	607.83	107.52
24	383.27	744.63	605.57	546.05	569.88	149.67

Table 57. Concentration of **BGAz-005** for PO (μM).

Time (h)	No. 1 (μM)	No. 2 (μM)	No. 3 (μM)	No. 4 (μM)	Mean (μM)	SD (μM)
0.083	1.181	2.582	1.898	2.225	1.971	0.597
0.25	1.508	2.149	2.747	4.334	2.685	1.211
0.5	1.613	3.535	3.656	5.316	3.530	1.514
1	2.250	4.430	2.932	5.398	3.752	1.426
3	1.230	3.163	1.451	2.011	1.964	0.865
5	1.410	1.910	1.504	1.734	1.640	0.226
8	1.165	1.366	1.309	1.744	1.396	0.247
24	0.880	1.710	1.391	1.254	1.309	0.344

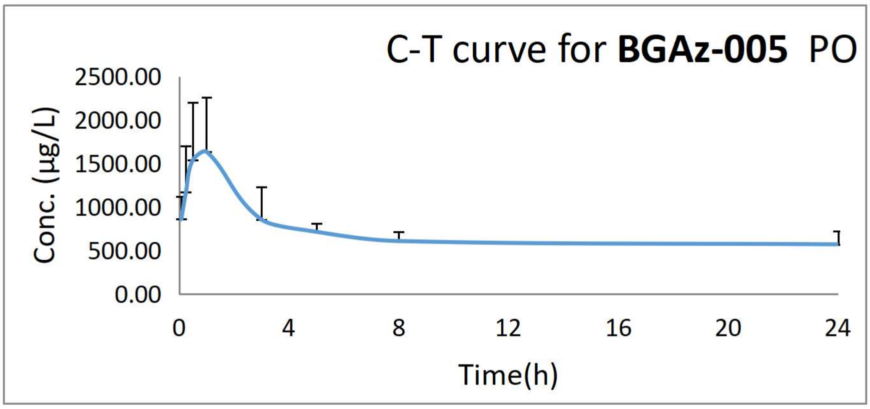

Figure 33

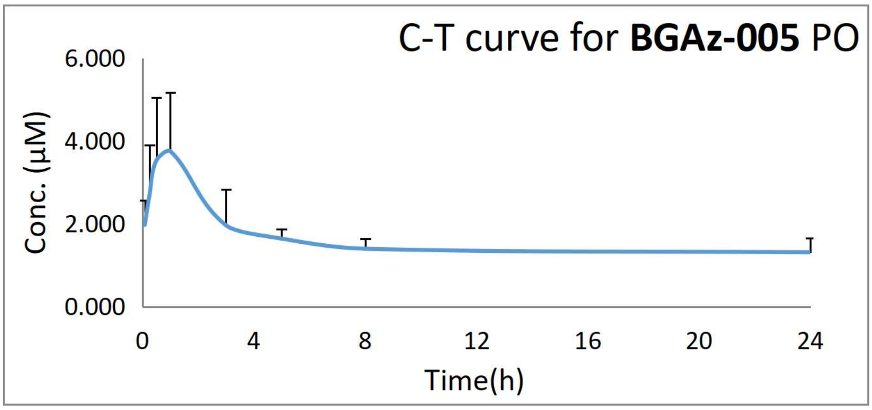

Figure 34

Table 58. Pharmacokinetic parameters of **BGAz-005** for PO.

Parameter	Unit	No. 1	No.2	No.3	No.4	Mean	SD	RSD/%
**AUCss**	μg/L*h	12178.507	19766.468	15705.449	19540.058	16797.621	3599.262	21.4
**AUC(0-t)**	μg/L*h	12178.507	19766.468	15705.449	19540.058	16797.621	3599.262	21.4
**AUC(0-∞)**	μg/L*h	35753.919	20021.166	228986.832	44229	82247.729	98338.883	119.6
**R_AUC(t/∞)**	%	34.1	98.7	6.9	44.2	45.975	38.518	83.8
**AUMC(0-t)**	h*h*μg/L	124409.562	209604.037	173532.842	180715.464	172065.476	35389.288	20.6
**AUMC(0-∞)**	h*h*μg/L	2135136.115	217275.027	80252935.26	1894415.043	21124940.36	39427892.07	186.6
**MRT(0-t)**	h	10.216	10.604	11.049	9.248	10.279	0.767	7.5
**MRT(0-∞)**	h	59.718	10.852	350.47	42.832	115.968	157.643	135.9
**VRT(0-t)**	h^2	70.025	83.458	81.101	70.246	76.208	7.078	9.3
**VRT(0-∞)**	h^2	3766.565	87.655	123543.426	2074.815	32368.115	60802.133	187.8
**λz**	1/h	0.016	0.163	0.003	0.022	0.051	0.075	147.1
**λz**		124	234	134	124	--	--	--
**C_last**	μg/L	384.659	41.631	606.838	543.67	394.2	252.951	64.2
**t1/2z**	h	42.473	4.24	243.564	31.47	80.437	109.932	136.7
**Tmax**	h	1	1	0.5	1	0.875	0.25	28.6
**Vz/F**	L/kg	51.426	9.167	46.046	30.802	34.36	18.931	55.1
**Vss/F**	L/kg	50.107	16.261	45.916	29.052			
**CLz/F**	L/h/kg	0.839	1.498	0.131	0.678	0.787	0.563	71.5
**Cmax**	μg/L	979.4331092	1928.734817	1591.628366	2350.260294	1712.514	578.934	33.8
**Cav**	μg/L	507.438	823.603	654.394	814.169	699.901	149.969	21.4
**DF**		1.93	2.342	2.432	2.887	2.398	0.393	16.4
